# The proneural gene *ASCL1* governs the transcriptional subgroup affiliation in glioblastoma stem cells by directly repressing the mesenchymal gene *NDRG1*

**DOI:** 10.1038/s41418-018-0248-7

**Published:** 2018-12-11

**Authors:** Ashwin Narayanan, Filippo Gagliardi, Alberto L. Gallotti, Stefania Mazzoleni, Manuela Cominelli, Luca Fagnocchi, Mauro Pala, Ignazio S. Piras, Paola Zordan, Nicole Moretta, Elisa Tratta, Gianluca Brugnara, Luisa Altabella, Giuseppina Bozzuto, Petra Gorombei, Agnese Molinari, Rose-Ann Padua, Alessandro Bulfone, Letterio S. Politi, Andrea Falini, Antonella Castellano, Pietro Mortini, Alessio Zippo, Pietro L. Poliani, Rossella Galli

**Affiliations:** 1 Neural Stem Cell Biology Unit, Division of Regenerative Medicine, Stem Cells and Gene Therapy, San Raffaele Scientific Institute, Via Olgettina 58, 20132 Milan, Italy; 2 Department of Neurosurgery and Gamma Knife Radiosurgery, San Raffaele Scientific Institute, Via Olgettina 60, 20132 Milan, Italy; 3 Department of Molecular and Translational Medicine, Pathology Unit, University of Brescia, 25124 Brescia, Italy; 4 Istituto Nazionale di Genetica Molecolare (INGM), Via Francesco Sforza 35, 20122 Milan, Italy; 5 CRS4, Biomedicine, Scientific Park of Sardinia, Pula, Cagliari, Italy; 6 Neurogenomics Division, Translational Genomics Research Institute (TGen), Phoenix, AZ USA; 7 Neuroradiology Unit and CERMAC, Vita-Salute San Raffaele University and San Raffaele Scientific Institute, 20132 Milan, Italy; 8 Center for Research and Drug Evaluation, Istituto Superiore di Sanità, Viale Regina Elena 299, 00161 Rome, Italy; 9 Institut Univérsitaire d’Hématologie, Hôpital Saint-Louis, 75010 Paris, France

**Keywords:** Cancer stem cells, CNS cancer

## Abstract

Achaete-scute homolog 1 gene *(ASCL1)* is a gene classifier for the proneural (PN) transcriptional subgroup of glioblastoma (GBM) that has a relevant role in the neuronal-like differentiation of GBM cancer stem cells (CSCs) through the activation of a PN gene signature. Besides prototypical *ASCL1* PN target genes, the molecular effectors mediating *ASCL1* function in regulating GBM differentiation and, most relevantly, subgroup specification are currently unknown. Here we report that ASCL1 not only promotes the acquisition of a PN phenotype in CSCs by inducing a glial-to-neuronal lineage switch but also concomitantly represses mesenchymal (MES) features by directly downregulating the expression of N-Myc downstream-regulated gene 1 (*NDRG1*), which we propose as a novel gene classifier of MES GBMs. Increasing the expression of *ASCL1* in PN CSCs results in suppression of self-renewal, promotion of differentiation and, most significantly, decrease in tumorigenesis, which is also reproduced by *NDRG1* silencing. Conversely, both abrogation of *ASCL1* expression in PN CSCs and enforcement of *NDRG1* expression in either PN or MES CSCs induce proneural-to-mesenchymal transition (PMT) and enhanced mesenchymal features. Surprisingly, *ASCL1* overexpression in MES CSCs increases malignant features and gives rise to a neuroendocrine-like secretory phenotype. Altogether, our results propose that the fine interplay between *ASCL1* and its target *NDRG1* might serve as potential subgroup-specific targetable vulnerability in GBM; enhancing *ASCL1* expression in PN GBMs might reduce tumorigenesis, whereas repressing NDRG1 expression might be actionable to hamper the malignancy of GBM belonging to the MES subgroup.

## Introduction

Predicting clinical course and delivering therapeutics based on the genetic makeup of tumors is of utmost significance to increase efficacy and prevent overtreatment in patients. This notion applies also to glioblastoma (GBM), which is the most malignant brain tumor of adults and accounts for 50% of the newly diagnosed glioma [1]. For many years, gliomas have been classified on the basis of tumor histology, which, together with tumor grading, resulted in a classification that reflected tumor malignancy and, to a certain extent, predicted disease course. Only recently, a genetic classification of GBM based on the isocitrate dehydrogenase 1 (IDH1) status has been included into the histologic criteria-based diagnosis by the 2016 WHO classification of brain tumors [2].

Among the many multidimensional molecular data available for GBM, transcriptional signatures classified GBMs into distinct subgroups, i.e., proneural (PN), mesenchymal (MES), classical (CL)/proliferative (P), and neural (NL) [3–5]. The PN signature correlates with a slightly less aggressive disease and increased response to anti-angiogenic treatment in patients with IDH-wild-type GBM, whereas the MES subgroup has been associated with radio-resistance and poorer prognosis [6, 7].

Given that 20% of GBMs are not transcriptionally homogeneous and comprise multiple populations of cells belonging to different subclasses [8, 9], the clinical significance and usefulness of gene expression-based GBM patient stratification is still under debate. However, an in-depth understanding of the transcriptional signatures of GBM might help identifying molecular mediators that could not only be useful to increase our knowledge of GBM pathogenesis but may also serve as diagnostic, prognostic and/or predictive markers to be incorporated into the clinical routine [10].

Notably, GBM progression and recurrence are very often accompanied by a shift into a predominant MES molecular phenotype [3, 11]. Unfortunately, the mechanisms that underlie PN to MES transition (PMT) are very poorly understood, with only few effectors being identified [3, 12–15].

Core transcription factors (TFs), which act as oncogenes or tumor suppressors, are now considered as potential therapeutic targets in cancer [16]. Given the importance of understanding the dynamics of TFs in modulating the stem cell state [17] and knowing that GBM cancer stem cells (CSCs) are enriched for helix-loop-helix (HLH) and sex-determining region Y (SRY)-containing TFs when compared to traditional glioma cell lines (GCLs) [18], we set out to investigate the subgroup-specific function of the PN TF Achaete-scute homolog 1 (ASCL1).

As other proneural factors, Ascl1 expression not only endows mouse progenitors with a neuronal fate, but also drives progenitors out of the cell cycle and initiates their differentiation [19]. Similarly, *ASCL1* expression in a subset of GBM CSCs activates neuronal target genes and promotes responsiveness to Notch inhibitors, thus resulting in impaired tumorigenicity [20].

In the present study, we extend the latter findings by reporting that *ASCL1* regulates the phenotypic switch between GBM subgroups by directly repressing the expression of N-Myc downstream-regulated gene 1 (*NDRG1*) that we functionally identified as a novel MES subgroup gene classifier. Remarkably, *ASCL1* overexpression efficiently reduces tumorigenesis in PN CSC-derived preclinical models of GBM. However, enforcing *ASCL1* expression in MES GBM CSCs promotes the development of xenografts, which acquire highly malignant neuroendocrine-like features. The possibility of hampering the progression of PN GBM by up-regulating the expression of ASCL1 highlights new therapeutics opportunities, but, at the same time, underscores the necessity for the accurate molecular stratification of GBM patients and for the identification of MES-restricted actionable molecular targets.

## Materials and methods

### In vitro culture of GBM CSCs

GBM CSC lines were cultured in standard serum-free medium containing EGF and FGF2 [21] (undifferentiated conditions). CSC differentiation was obtained by culturing them on Matrigel, after withdrawal of mitogens from the culture medium and addition of 2% FBS for 7 days (differentiated conditions) [22].

### Microarray-based gene expression profiling and gene set enrichment analysis

Total RNA was isolated from GBM CSCs and GCLs using the RNeasy Mini Kit (Qiagen, Chatsworth, CA, USA) with DNase digestion. Biotinylated cRNA probes were synthesized using the GeneChip Whole Transcript Sense Target Labeling Assay Kit (Affymetrix) following the manufacturer’s instructions. Following fragmentation, biotinylated cRNA probes (25 ng/µL in 100 µL hybridization cocktail) were hybridized for 17 h at 45 °C on GeneChip® Human Gene 1.0 ST Array (Affymetrix). Gene Set Enrichment Analysis (GSEA) [23] was used to assess the degree of association between GBM CSC/GCL signatures and the molecular classification as in the NCBI GEO GSE4271 GBM patient cohort. Details of bioinformatics analysis are provided as Supplementary Methods.

### Quantitative real-time PCR

One μg of total RNA was reverse-transcribed by using first strand synthesis kit Superscript III RNaseH^-^ Reverse Transcriptase (Invitrogen, Carlsbad, CA) and OligodT primers. Each cDNA was diluted 1:3. Quantitative real-time PCR was performed by the IQ SybrGreen technology (Biorad, Hercules, CA, USA) following manufacturer’s instructions. Human-specific primers for *ASCL1*, *NDRG1*, *DLL3*, *HES6*, *CD44, NMYC, EGFR*, *CEBPD*, and *TIMP1* were purchased from Sigma (KiCqStart™ Primers**)**. ΔCt of the gene on each sample was calculated on its matched beta-actin. Data analysis was performed by the ΔΔ*C*_t_ method.

### Bright-field immunohistochemistry

Two µm sections were cut from paraffin blocks containing subgroup-classified human GBM samples as well as brain from mice transplanted with CSCs. Sections were stained with primary antibodies (provided as Supplementary Methods). Sections were then incubated with the secondary antibody (ChemMATE Envision Rabbit/Mouse, Dako Cytomation) or with the NovolinkTM Polymer Detection System (NovocastraTM). The retrospective study on human samples was conducted in compliance with the Declaration of Helsinki and with policies approved by the Ethics Board of Spedali Civili di Brescia, University of Brescia. Specifically, for the retrospective and exclusively observational study on archival material obtained for diagnostic purpose, patient consent was not needed (Delibera del Garante n. 52 del 24/7/2008 and DL 193/2003).

### Western blotting and nanopro assay

Lysates from GBM CSCs and GCLs were homogenized in 10× volume of RIPA lysis buffer. Proteins were separated by electrophoresis on 8–10% polyacrilamide (Sigma, St. Louis, MO) gels and transferred onto trans-blot nitrocellulose membranes (Amersham). Primary antibodies were diluted in 3% bovine serum albumine (Sigma) in TBS-T, and incubated with the membranes overnight at 4 °C. The primary antibody was removed, and the blots were washed in TBS-T and then incubated for 1 h at room temperature in horseradish peroxidase-conjugated secondary antibodies (Amersham). Reactive proteins were visualized using LiteBlot (Euroclone, Padriciano, Italy) or SuperSignal West Femto chemiluminescence reagent (Pierce Biotechnology, Rockford, IL) and exposure to X-ray film (BioMax MR; Kodak, Rochester, NY). For NanoPro assay, all isoelectric separations were performed on the NanoPro 1000 (ProteinSimple, Santa Clara, CA, USA) with the Premix Generation 2 pH 3–10 separation mix. Details can be found in Supplementary Methods.

### Generation of lentiviral vectors for gene overexpression

cDNAs for human *ASCL1* (Upstate, Lake Placid, NY, USA) and *NDRG1* (Origene, Rockville, MD, USA) were cloned into the pC.sin.cPPT.PGK.GFP.WPRE11 monocistronic transfer lentiviral vector (LV) in place of the GFP sequence. GBM CSCs were transduced with 1 × 10^7^ TU/mL of LVs for 16 h. Sister cultures were infected with pCCL.sin.cPPT.PGK.GFP.WPRE11, as mock condition.

### Immunocytochemistry

ICC was performed on undifferentiated GBM CSCs, plated at 3.5 × 10^5^ cells/cm^2^ on Matrigel (Becton and Dickinson, San Jose, CA)-coated glass coverslips for 24 h, and on their differentiated progeny.

For intracellular epitopes detection, the cells were permeabilized for 10 min with 0.1% Triton X-100 in PBS. Cells were then incubated with primary antibodies diluted at the appropriate concentration in PBS-10% NGS over night at 4 °C. Secondary antibodies were then added for 1 h at room temperature. Nuclei were counterstained with TOPROIII (Invitrogen), 1:2000 in PBS or DAPI (Fluka, Buchs, Switzerland).

### Invasion assays

Invasion assays were performed in Matrigel-coated 8µm-pore Transwell chambers (Corning Costar, Cambridge, MA). Overall, 2 × 10^5^ GBM CSCs were seeded in sister cultures on the upper side of the chambers in complete medium and allowed to migrate for 7 and 10 days. Non-invaded cells on the upper side of filters were or were not scraped off, and those migrated onto the lower side were fixed and stained by using DiffQuick (Dade Behring, IL, USA).

The invasive behavior of GBM CSCs was analyzed by FEG-SEM and LSCM. The samples were fixed with 2.5% glutaraldehyde at 4 °C for 1.5 h, post-fixed in 1% OsO_4_ for 2 h, and dehydrated using a graded ethanol series. Critical point-dried samples were sputtered with gold. Surface images were then acquired by a FEI FEG-SEM 200 microscope.

### Gene silencing

LV particles coding for shRNAs targeted against human *ASCL1* and *NDRG1* were purchased from Sigma (Mission™ RNAi). Infection of GBM CSCs was performed according to the manufacturer’s instruction at MOI 10.

### Orthotopic implantation of GBM CSCs

Tumorigenicity was determined by injecting CSCs orthotopically into the brain of *nu/nu* mice. Two x10^5^ GBM CSCs were suspended in 2 µL of DMEM supplemented with DNase (Sigma) and delivered into the right striatum (0.2 μL/min) by stereotactic injection with a micro-syringe (Hamilton). All animal experiments were approved by and performed in accordance with the guidelines of the International Animal Care and Use Committee.

### Chromatin immuno precipitation assay (ChIP)

Each ChIP experiment was performed in at least three independent biological samples and performed as previously described [24]. Briefly, 1 × 10^6^ GBM CSCs, over-expressing either ASCL1 or GFP as control, were cross-linked with 1% formaldehyde for 10 min at r/t and the reaction was quenched by glycine at a final concentration of 0.125 M. Cells were lysed in lysis buffer and chromatin was sonicated. Fifty μg of each sonicated chromatin sample were incubated o/n at 4 °C with the following antibodies: anti-IgG (Santa Cruz) and anti-MASH1 (Ascl1; BD Pharmingen). Immunoprecipitated DNA was analyzed by qPCR by using SYBR GreenER kit (Invitrogen). Values were normalized to those obtained with a non-immune serum and divided by input. The data shown represent triplicate qPCR measurements of the immunoprecipitated DNA. The data are expressed as (‰) express 1/1000 of the DNA inputs. All ChIP experimental values were normalized to those obtained with the relative input sample.

### RNA sequencing

RNA from GCLs and CSCs was extracted by using the RNeasy mini kit (Qiagen) according to manufacturer’s protocol. The cDNA was synthesized starting from total RNA using SMART technology. After barcoding, the RNA libraries were pooled, denatured and diluted to 2.4 pM final concentration. RNA sequencing was performed by using NextSeq 550 (Illumina) set for 76 cycles in single end (SE), yielding an average of 15 × 10^6^ clusters for each sample. Sequences were aligned using STAR (version 2.5.3a) on the reference genome GRCh38; association between reads and genes has been performed by feature counts, using gencode (version 28) basic annotation as reference. Analysis of count data was performed using the DESeq2 (differential gene expression analysis based on the negative binomial distribution) pipeline (version 1.6.3). The independent filtering of genes with low counts was set to a mean of 9 raw counts between all samples. The cut off imposed for differential gene expression was the one suggested by the SEquencing Quality Control consortium, which defines a gene as differentially expressed when it has an associated raw *p*-value lower than 0.01 and, at the same time, the absolute value of its log2FC is greater than 1 (log2FC > 1 or log2FC < −1).

### MRI acquisition and analysis

MR imaging was performed on a small animal-dedicated 7T scanner (30/70 BioSpec; Bruker, Ettlingen, Germany). The animal protocol included high-resolution T2 and T1 sequences as well as dynamic contrast-enhanced (DCE) perfusion MR imaging performed by using a dynamic gradient-echo T1-weighted sequence during the injection of a 1:9 dilution of gadobutrol (Gadavist; Bayer Schering Pharma, Berlin, Germany; 100 μL of contrast in 900 μL of physiological solution). The total injected volume was of 0.08 mL, at a rate of 600 μL/s. DCE-MR imaging was preceded by a saturation recovery sequence for T1 mapping and followed by a contrast-enhanced T1 sequence for anatomic reference. The complete details of the MRI sequences and analysis are provided as Supplementary Methods.

### Statistics

For experiments involving patients’ samples or in vitro CSC/GCL cultures, *n* represents the number of single patient-derived samples and CSC lines. For experiments involving transplanted CSC lines, *n* represents the number of individual animals that were transplanted with a single CSC line. Results for continuous variables were expressed as mean ± standard error mean (s.e.m.). Two-group comparisons were performed with the independent samples’ one-tailed Student *t-*test. *p* values < 0.05 were considered statistically significant. *p < 0.05; **p < 0.01; ***p < 0.005; ****p < 0.001.

## Results

### *ASCL1* is a specific marker of proneural (PN) GBM CSCs

To identify gene signatures that might predict the transcriptional subtypes of GBM CSCs, we subjected patient-derived GBM CSC lines (*n* = 6, i.e., L0605, L0306, L0627, L0104, L0512 and L0125) to microarray-based transcriptome profiling [22]. The transcriptional profile of the CSC lines was analyzed and compared with that of several patients’ GBM tissue specimens (*n* = 28). As non-stem controls, we analyzed standard human glioma cell lines (GCLs) (*n* = 4, i.e., U87, U373, T98G, and LN18), as well as serum-cultured primary GBM lines (*n* = 2) [21, 25].

**Fig. 1:**
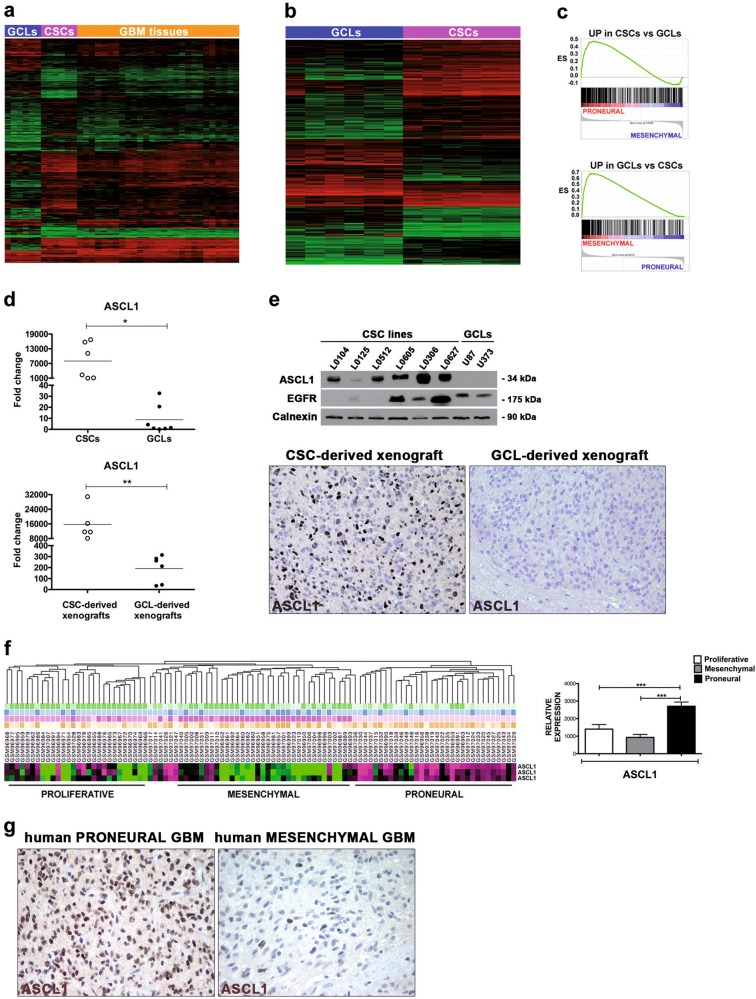
*ASCL1* is a specific marker of proneural (PN) GBM CSCs. **a** Unsupervised hierarchical whole-transcript expression analysis of human GCLs, CSCs and tumor samples highlights a high extent of similarity between CSCs and GBM tissues. **b** Supervised clustering analysis of transcriptional profiles sharply distinguishes CSCs from GCLs. **c** GSEA indicates that genes upregulated in CSCs vs. GCLs are enriched in the PN subgroup of human GBM, whereas those upregulated in GCLs vs. CSCs are enriched in the MES subgroup. **d** Validation of the upregulation of *ASCL1* in CSCs, GCLs, and corresponding xenografts by qRT-PCR. **e** Validation of the upregulation of *ASCL1* in CSCs, GCLs and corresponding xenografts by WB and IHC. **f** Cluster heatmap, generated by applying the NCBI GEO DataSet Cluster Analysis tool to the GDS1816 (GSE4271) dataset, shows enhanced expression of *ASCL1* in the PN subgroup of human GBM. Quantification of the expression of *ASCL1* in the same dataset is shown in the right panel. **g** IHC analysis indicates that ASCL1 expression is restricted to PN GBM specimens

Unsupervised hierarchical clustering analysis revealed that the gene expression profile of CSCs, rather than that of serum-grown GCLs/GBM cultures, closely mirrored that of GBM specimens (Fig. 1a).

To identify molecular mediators regulating GBM molecular subgrouping, we compared the global gene expression profile of CSCs vs. that of GCLs by supervised clustering analysis (Fig. 1b). This analysis returned an output list containing 1651 differentially expressed genes (DEGs), ranked based on log2 fold change in expression between the two sample sets with a *p* < 0.001 significance. To investigate whether this gene signature was enriched in molecular subtypes of human GBM, we selected 557 genes that were upregulated at least twofold in CSCs vs. GCLs and 424 genes that were upregulated at least twofold in the reciprocal comparison. Then, we performed Gene Set Enrichment Analysis (GSEA) on the publicly available GEO dataset GSE4271, which contained expression data from 100 human GBM samples classified as proneural (PN), mesenchymal (MES) and proliferative (P) [3]. Interestingly, the expression of genes upregulated in CSCs vs. GCLs was more strongly associated with the PN subgroup than with either the MES (Fig. 1c, Supplementary List 1 and Supplementary Table 1) or the P (Supplementary Table 2) subgroups. Notably, genes overexpressed in GCLs vs. CSCs were highly enriched in the MES subgroup (Fig. 1c, Supplementary List 2 and Supplementary Table 1).

Next, from the list of genes that were upregulated in CSCs, we selected the top-ranking proneural subgroup gene classifier *ASCL1* and confirmed that it was more highly expressed in CSCs and in their xenografts than in GCL and in their corresponding tumors (Fig. 1d), also at the protein level (Fig. 1e).

To assess whether *ASCL1* was expressed in human GBM specimens, we queried GEO-available datasets, which confirmed the enhanced expression in the PN subgroup (Fig. 1f). Most remarkably, we tested several GBM samples that were previously classified based on both transcriptional profiling and immunohistochemistry (IHC) for subgroup-restricted markers and found that ASCL1 protein was specifically retrieved in human PN samples (*n* = 6 and *n* = 10 patients for PN and MES, respectively) (Fig. 1g). As such, ASCL1 is a CSC-specific mediator that might potentially have a significant role in GBM subgroup phenotypic specification.

### ASCL1 overexpression in GBM CSCs promotes neuronal differentiation through a glial-to-neuronal lineage switch

**Fig. 2:**
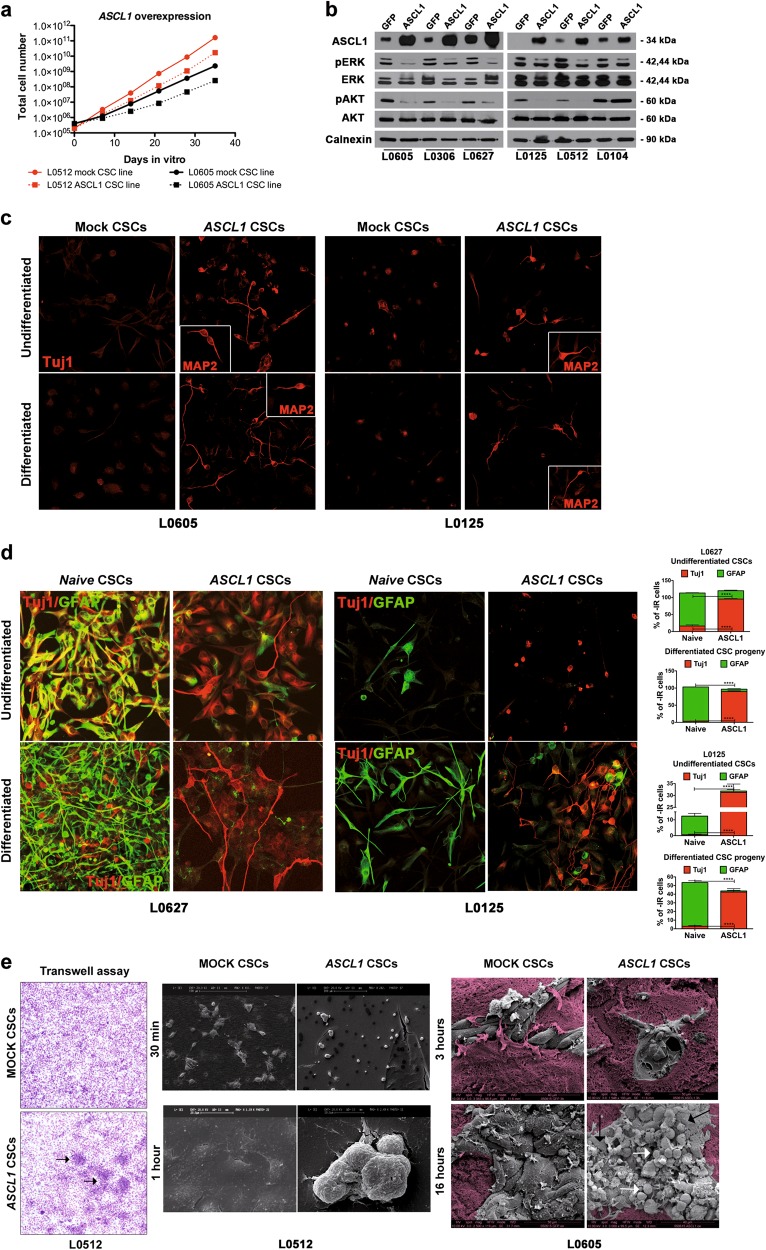
ASCL1 overexpression in GBM CSCs promotes neuronal differentiation and lineage switch. **a** *ASCL1* overexpression in CSCs significantly reduces their long-term self-renewal (population analysis; representative analysis of *n* = 3 experiments; *p* value of the comparison EGFR^pos^ mock vs. ASCL1-overexpressing CSCs < 0.05; *p* value of the comparison EGFR^neg^ mock vs. ASCL1-overexpressing CSCs < 0.05). **b** ERK and AKT pathways are hypoactivated in *ASCL1*-transduced GBM CSCs (WB). **c** *ASCL1* overexpression in CSCs induces neuronal differentiation under proliferative (upper panels) and differentiative (lower panels) conditions, as assessed by early (Tuj1) and late (MAP2) neuronal markers (Tuj1, red, ×400; MAP2, red, inset ×400). **d** *ASCL1-*induced neuronal differentiation of CSCs is accompanied by the concurrent repression of the astroglial phenotype under both proliferative (upper left panels) and differentiative (lower left panels) conditions (Tuj1, red; GFAP, green; ×400). Quantification of the frequency of Tuj1- and GFAP-IR cells after *ASCL1* overexpression (right panels). **e** *ASCL1* overexpression induces CSCs to invade as clusters of cells (black arrows), whereas mock CSCs move as single cells (left panels) (standard Matrigel Transwell invasion assay). *ASCL1* overexpression increases invasion (30 min), triggers a shift from single-cell to collective migration (1 h; representative CSC line L0512), and induces the formation of heterogeneous ‘niche’-like structures (3–16 h; epithelioid cells: black arrows; ‘satellite cells’: white arrows; the pseudocolor magenta identifies the Matrigel layer; representative CSC line L0605) (scanning electron microscopy, SEM, Matrigel Transwell invasion assay, right panels)

To understand the putative role of *ASCL1* in regulating CSC properties, we either silenced or overexpressed *ASCL1* in CSCs exhibiting a PN molecular phenotype (Supplementary Fig. 1a–d). Notably, whereas *ASCL1* silencing in two distinct CSC clones (C) (i.e., C50 and C51) did not result in any significant difference in long-term self-renewal, proliferation rate, differentiation potential and invasive ability in vitro (data not shown), *ASCL1* overexpression in CSC lines negatively affected their self-renewal ability as compared to GFP-transduced mock controls (Fig. 2a). In agreement with these findings, activation of the pro-proliferative pathways pERK_Thr202/Tyr204_ and pAKT_Ser473_ was strongly diminished in most CSCs upon *ASCL1* overexpression (Fig. 2b). Interestingly, even when maintained under proliferative culture conditions (i.e., in the presence of EGF and FGF2), *ASCL1*-overexpressing CSCs showed a significant increase in the frequency of cells displaying a typical neuronal-like morphology and immunoreactive (-IR) for early (Tuj1) and late (MAP2) neuronal markers (Fig. 2c and Supplementary Fig. 2) (*n* = 6 CSC lines tested). The same enhanced neuronal differentiation was also evident when CSCs were challenged to differentiate by mitogen removal and exposure to serum (Fig. 2c and Supplementary Fig. 2). Moreover, the increased neuronal commitment promoted by *ASCL1* was accompanied by a decrease in the number of GFAP-IR astroglial cells, indicating that *ASCL1* expression promotes a lineage switch in CSCs by activating the neuronal fate and repressing the glial one (Fig. 2d).

The overall migration and invasion properties of GBM CSCs in vitro were also affected by *ASCL1* overexpression, with CSCs progressing from single cell-driven to homotypic collective invasion and migrating as compact clusters of tumor cells (Fig. 2e). Notably, *ASCL1*-overexpressing CSC-derived clusters were composed by large epithelioid cells, which were covered by small and rounded cells (‘satellite’ cells), thus suggesting that phenotypic cellular diversification was occurring upon *ASCL1* overexpression.

### ASCL1 directly represses the expression of the MES gene NDRG1

To identify novel transcriptional targets that might mediate the ASCL1-dependent phenotype, we analyzed different genes, whose sequence is predicted to contain ASCL1 binding sites [26–28], might have a role in GBM-relevant signaling pathways and are downregulated in PN CSCs (data not shown). By this biased approach, we selected *N-Myc downstream-regulated gene 1* (*NDRG1*) and *EGFR* as potential ASCL1 targets. NDRG1 is a well-known marker of mTORC2 pathway activation, whereas EGFR is a GBM-specific marker [10] and a pivotal regulator of CSC malignancy [22].

**Fig. 3:**
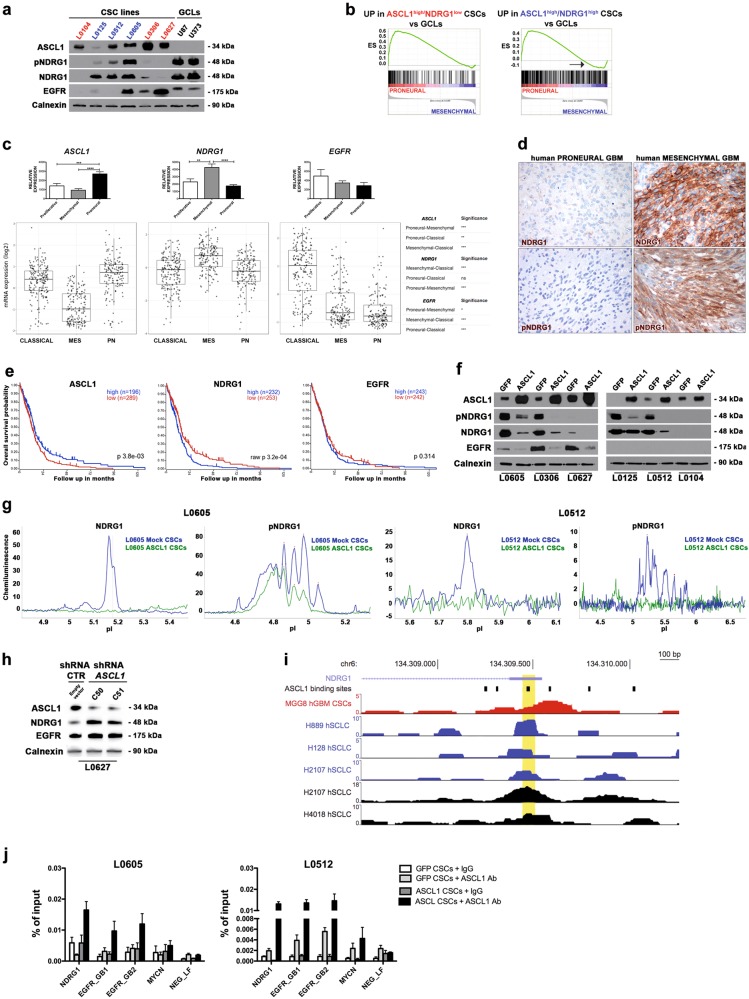
ASCL1 directly represses the expression of the MES gene NDRG1. **a** CSCs and GCLs show distinct patterns of endogenous ASCL1, NDRG1, and EGFR expression (WB). **b** The gene signature associated with high NDRG1 expression in PN CSC lines is enriched in the PN subgroup but also comprises a subset of genes (black arrow) correlated with the MES subgroup (GSEA). **c** *ASCL1*, *NDRG1*, and *EGFR* expression is upregulated in the human PN, MES, and P/CL GBM subgroups, respectively (upper histograms, expression data from Phillips dataset; lower histograms, expression data from the TCGA). **d** The expression and activation of NDRG1 by IHC are restricted to GBM specimens classified as MES (NDRG1 and pNDRG1, brown). **e** High *ASCL1* expression in GBM positively correlates with a slightly more favorable prognosis than low expression, whereas high NDRG1 expression is associated with a worse outcome (Kaplan–Meier survival curves; data from TCGA). No differences in survival are retrieved in patients stratified based on EGFR expression. **f** *ASCL1* overexpression in CSCs downregulates NDRG1 and EGFR expression (size-based WB). **g** The same results were confirmed by charge-based WB (Representative CSC lines: L0605 and L0512). **h** *ASCL1* silencing de-represses NDRG1 and EGFR expression (R7epresentative CSC line: L0627). **i** Genomic snapshot depicting *NDRG1* transcription start site and its promoter region. ASCL1 binding sites are indicated by solid black boxes. Representative ASCL1 ChIP-seq data produced in human GBM CSCs (red profile) [29] and in multiple lung cancer cell lines from independent laboratories (blue and black profiles) [28, 30] are reported. The yellow highlight indicates the site screened by ChIP-qPCR in L0512 and L0605 CSC lines. Coverage data are represented as normalized RPM. (**j**) ChIP analysis indicates binding of ASCL1 to promoter/intragenic binding sites of *NDRG1* and *EGFR* genes

NDRG1, in particular, showed a distinguished pattern of expression in CSCs and GCLs (Fig. 3a). Three out of 6 CSC lines expressed high levels of ASCL1 with very low to null NDRG1 expression (ASCL1^high^/NDRG1^low^), whereas the other three expressed both proteins at similar level (ASCL1^high^/NDRG1^high^). On the contrary, GCLs never expressed ASCL1, while displaying very high expression of NDRG1 (Fig. 3a).

To understand whether the pattern of expression of ASCL1 and NDRG1 in CSCs might impact on their association with specific molecular subgroups, GSEA was applied to the gene expression profiles of CSC lines, clustered based on the relative ASCL1 and NDRG1 protein expression, after comparing them with GCLs. Interestingly, the 205 genes upregulated in ASCL1^high^/NDRG1^low^ CSCs vs. GCLs (Supplementary List 3) were more highly enriched in the PN subgroup than the 470 genes upregulated in ASCL1^high^/NDRG1^high^ CSCs vs. GCL (Supplementary List 4), as shown by high NES values (Supplementary Table 3). Very interestingly, 24 out of the 470 genes upregulated in ASCL1^high^/NDRG1^high^ CSCs correlated positively with those specific for the MES subgroup (i.e., they showed a decreasing negative running ES), suggesting that NDRG1 expression might correlate with the acquisition of mesenchymal traits (Fig. 3b and Supplementary List 4).

To prove this hypothesis, we performed an in silico analysis of the expression of *NDRG1*, together with that of *ASCL1* and *EGFR*, in the three known molecular subgroups of human GBM as available in the GEO dataset GSE4271 (GEO Data Analysis Tools) and in the TCGA dataset (Tumor Glioblastoma-TCGA-540-MAS5.0; R2, Genomics Analysis and Visualization Platform, http://r2.amc.nl) (Fig. 3c). Expression of *ASCL1* was retrieved at significantly higher levels in the PN subgroup than in the MES, whereas *NDRG1* expression was enhanced in the MES subgroup (Fig. 3c). *EGFR* was expressed in all the three groups with a positive trend of expression both in the P and in the CL subgroups (Fig. 3c).

IHC for NDRG1 on GBM specimens confirmed high positivity for NDRG1 in MES GBMs (Fig. 3d). Remarkably, as reported in the TCGA dataset, the level of expression of each of the three genes associates with a different patients’ prognosis, with patients showing upregulation of *ASCL1* having a better survival than those with low *ASCL1* expression and patients with high *NDRG1* expression having a worse outcome (Fig. 3e). *EGFR* expression did not significantly impact on the overall survival (Fig. 3e). In line with these findings, R2 analysis of *ASCL1* and *NDRG1* expression in the Tumor Glioma French dataset indicated that *NDRG1* expression was significantly increased in (a) high-grade as compared to low-grade gliomas and (b) IDH1 wild type vs. IDH1 mutant gliomas, while *ASCL1* expression showed the opposite trend (Supplementary Fig. 3a, b). Thus, the reciprocal pattern of expression of *ASCL1* with respect to *NDRG1* may define human GBM molecular subgroups and the molecular interplay among these genes might have relevant clinical implications.

To understand how the expression of *NDRG1* and *EGFR* was regulated by ASCL1, we tested NDRG1 and EGFR protein expression in *ASCL1*-overexpressing-PN CSCs. Notably, the expression of total NDRG1 and its phosphorylated form pNDRG1_Thr346_ was strongly decreased by *ASCL1* overexpression, as shown by both size-based WB (Fig. 3f) and charge-based Nanoblot immunoassay (Fig. 3g). In the same way, *EGFR* overexpression was diminished by ASCL1 in *EGFR*-expressing CSC lines (i.e., L0605, L0627, and L0306) (Fig. 3f). Notably, knockdown of *ASCL1* expression in CSCs elicited a significant increase in both NDRG1 total and phosphorylated forms as well as in EGFR (Fig. 3h), suggesting that ASCL1 was either directly or indirectly repressing the expression of the two genes.

To identify the mechanism(s) through which the ASCL1-dependent modulation of NDRG1 expression may take place, we assessed whether NDRG1 regulation by ASCL1 was mediated by mTORC2 (Supplementary Fig. 3c–e). To this end, we checked the activation of the NDRG1 upstream activator pSGK1 and observed that its pattern of activation did not correlate with that of pNDRG1 (Supplementary Fig. 3c). Accordingly, mTORC2 inhibition by long-term treatment with rapamycin in *ASCL1*-overexpressing CSCs only partially rescued NDRG1 expression (Supplementary Fig. 3d). Thus, NDRG1 regulation by ASCL1 may be achieved predominantly through mTORC2-independent mechanisms.

Thus, to determine if the change in both NDRG1 and EGFR expression was a function of variation in the transcription at the *NDRG1* and *EGFR* gene loci as a consequence of ASCL1 binding to intrapromoter/intragenic E-box sites, we performed a qPCR analysis that indicated that the expression pattern of *NDRG1* and *EGFR* primary transcripts mirrored that of the corresponding proteins (Supplementary Fig. 3e). Thus, the regulation of *NDRG1* and *EGFR* expression by ASCL1 seemed to entail an active transcriptional process.

As such, to assess whether *NDRG1* and *EGFR* were direct transcriptional targets of ASCL1 in CSCs or, in the case of *NDRG1*, to clarify if its expression was regulated indirectly through *MYCN*, which is also a predicted transcriptional target of ASCL1 [27], we performed ChIP analysis for all three genes on CSCs expressing ASCL1, either endogenously or ectopically. To assess ASCL1 binding on *NDRG1* promoter, we designed ChIP primers based both on ASCL1 putative binding sites and publicly available ASCL1 ChIP-seq datasets of GBM and other tumors [27, 29, 30]. We identified ten putative ASCL1 binding sites nearby the transcription starting site of *NDRG1* (±500 bp), by querying the JASPAR2018 database with a relative profile score of 80%. Through ChIP-qPCR, we evaluated the binding of ASCL1 by testing a region comprising a putative ASCL1 binding site and a ChIP-seq peak conserved among the public available datasets (Fig. 3i). Core *MYCN* promoter (−429/−340) fragments as well as promoter and intragenic regions of *EGFR* (gene body, GB, positions + 44685/+44747 and +44712/+44772) were also co-precipitated using the anti-ASCL1 antibody. As controls, we tested *DLL3* and *DKK1* genes, whose expression was up- and downregulated in PN CSCs vs. GCLs, respectively (Supplementary List 1 and data not shown), and that are known to be direct transcriptional targets of ASCL1 [20, 29]. Both *NDRG1* and *EGFR* resulted as direct targets of ASCL1, with *NDRG1* bound by ASCL1 on the promoter region and *EGFR* on intragenic regions (Fig. 3j). No significant binding to *MYCN* promoter was observed (Fig. 3j). Binding of ASCL1 to the promoter/enhancer of both *DLL3* and *DKK1* was observed also in our CSC lines (Supplementary Fig. 3f). Altogether, these data indicate that ASCL1 expression represses *NDRG1* and *EGFR* expression in a direct manner and that their expression is inversely related.

In support of these experimental findings, in silico Pearson correlation analysis of human GBM specimens by R2 indicated that *NDRG1* expression was anti-correlated to that of *ASCL1* in the majority of GBM samples as well as in other different types of cancer (TCGA and PANCANCER datasets; Supplementary Fig. 4a–c).

### Modulation of the expression of *ASCL1* in GBM CSCs affects their subgroup affiliation and tumorigenic behavior

**Fig. 4:**
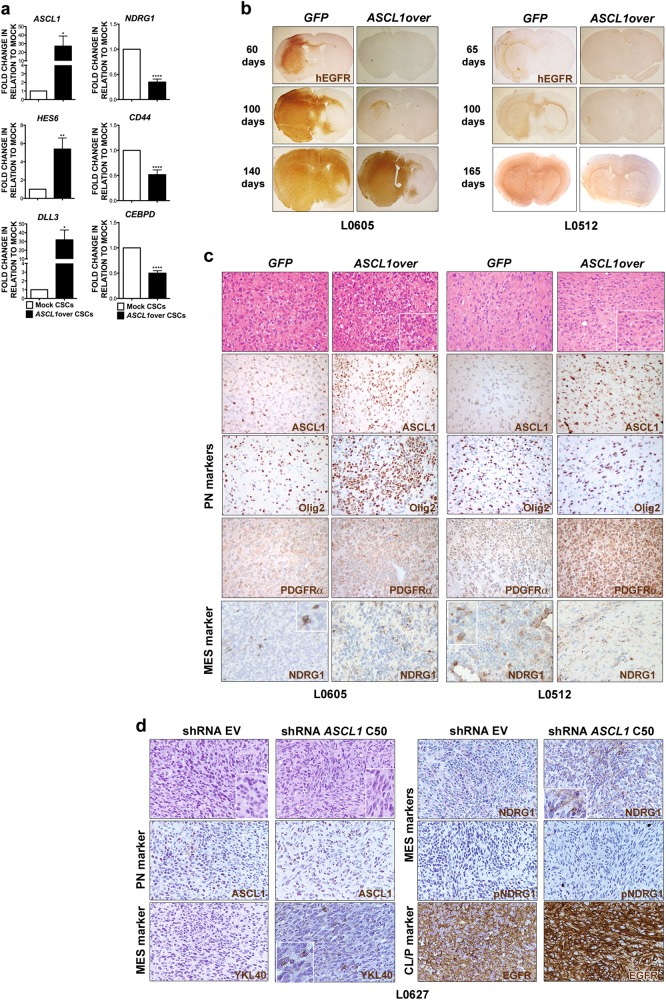
Modulation of the expression of *ASCL1* in GBM CSCs affects their subgroup affiliation and tumorigenic behavior. **a** qPCR indicates that, concomitant with PN marker upregulation, *ASCL1* overexpression in PN CSCs induces downregulation of MES markers. **b** *ASCL1*-overexpressing CSC-derived orthotopic xenografts develop more slowly than controls over a 5 month-period (Representative CSC lines: L0605 and L0512; human-specific EGFR staining: brown, ×20). **c** Intracranial tumors generated by *ASCL1*-overexpressing CSCs, analyzed at days 140 (L0605) and 165 (L0512), show a differentiated morphology, comprise small neuronal-like cells organized as rosettes (H&E, ×400; insets, ×800) and display enhanced expression of the PN markers Olig2 (L0605) and PDGFRα (L0512). Accordingly, the expression of the MES marker NDRG1, which was retrieved only in a minority of tumor cells in control GFP tumors (inset, ×800), was completely turned down after *ASCL1* overexpression (white matter fibers: internal staining control). All markers stained in brown, ×400. **d** *ASCL1*-silenced CSC-derived xenografts analyzed 2 months after transplantation display signs of PN-to-MES transition, as proven by (i) the development of focal areas containing large, spindle-shaped cells with elongated nuclei (H&E, ×400; inset, ×800) and (ii) the immunoreactivity for MES markers as YKL40 and NDRG1. Upregulation of the malignant CL/P marker EGFR was also detected. All markers stained in brown, ×400

To understand whether the enforced expression of *ASCL1* in CSCs was activating known downstream targets of the gene, which are also PN markers, we tested the expression of *HES5*, *HES6,* and *DLL3* by qPCR analysis. In agreement with previous observations [20], their expression strongly increased in *ASCL1*-overexpressing GBM CSCs (Fig. 4a). Most notably, *ASCL1* overexpression not only resulted in the upregulation of PN gene classifiers, but also in the concurrent downregulation of the expression of MES-specific genes (Fig. 4a).

Then, to assess the role of *ASCL1* in modulating subgroup acquisition in vivo, we transplanted into the brain of *nu/nu* mice different PN CSC lines, either over- or under-expressing *ASCL1* (*n* = 4 mice for each condition and each CSC line). *ASCL1*-overexpressing CSC-derived tumors were characterized by a significant reduction in their growth capacity over time with respect to mock controls (Fig. 4b), as shown by the extension of the human-specific EGFR staining, with some *ASCL1*-transduced CSC lines giving rise to slowly growing tumors (i.e., L0605 and L0512) (Fig. 4b), and others even failing in promoting xenografts development at the latest time point assessed for controls (i.e., L0306) (Supplementary Fig. 5a). Interestingly, the majority of cells within the tumor xenografts derived by *ASCL1*-transduced CSCs were small in size and focally organized as circular rosette structures around an eosinophilic neuropil core, thus strongly resembling typical neuronal differentiation (Fig. 4c). Of note, these cells expressed very high level of canonical PN markers such as Olig2 and PDGFRα [31] (Fig. 4c), suggesting that ASCL1 was eliciting a proneural function also in vivo. Notably, the overall immunoreactivity for NDRG1 was very low to negligible in mock tumors, and completely disappeared upon *ASCL1* overexpression (Fig. 4c).

Very interestingly, *ASCL1*-silenced CSCs gave rise to tumors that, while not showing differences in tumor growth, were morphologically pleomorphic and displayed MES features (Fig. 4d). Indeed, whereas tumors induced by control CSCs were characterized by the presence of small and actively proliferating cells, with signs of necrosis and apoptosis, *ASCL1*-silenced CSC-derived tumors did contain several focal areas composed by larger, spindle-shaped pleomorphic cells, with elongated nuclei and organized in bundles (Fig. 4d). Accordingly, *ASCL1*-silenced tumors showed a significant increase in the expression of the mesenchymal markers YKL40, and most remarkably, NDRG1, as expected by the relief of ASCL1-mediated transcriptional repression (Fig. 4d). Likewise, the expression of the EGFR, the other ASCL1 transcriptionally repressed target identified in our study, was upregulated in *ASCL1*-silenced tumors (Fig. 4d).

As such, *ASCL1* expression may modulate GBM malignancy in vivo by regulating the PN-to-MES subgroup switch.

### NDRG1 downregulation in CSCs mirrors the *ASCL1*-induced phenotype, whereas its overexpression promotes PN-to-MES transition (PMT)

**Fig. 5:**
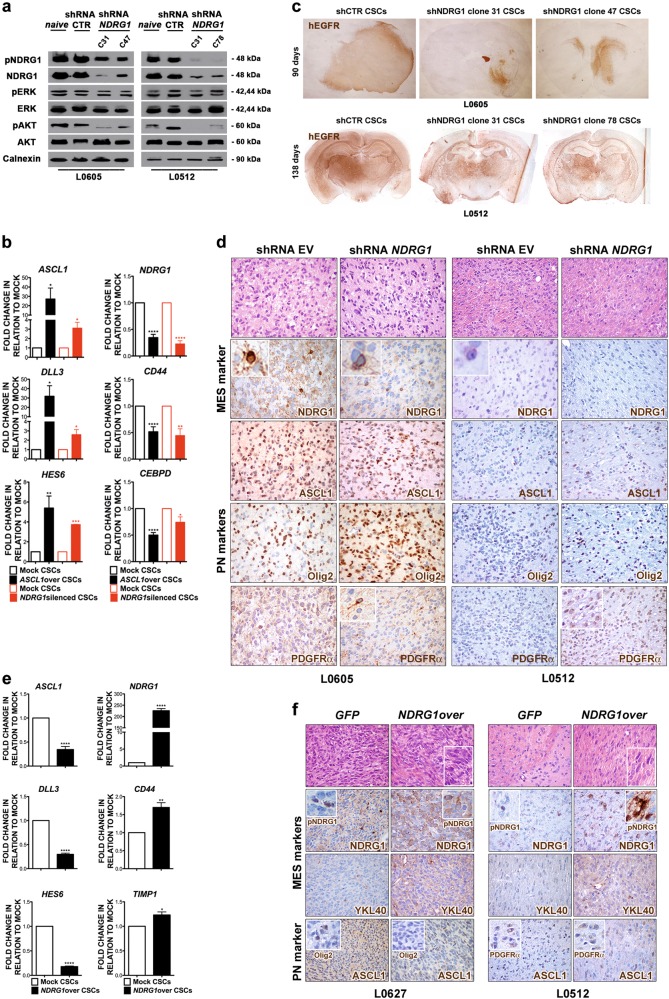
NDRG1 downregulation in CSCs mirrors the *ASCL1*-induced phenotype, whereas its overexpression promotes PN-to-MES transition (PMT). **a** *NDRG1* silencing in PN CSCs results in significant AKT hypoactivation (WB). **b** qPCR analysis of gene classifiers in *NDRG1*-silenced CSCs (red-lined and red-filled bars in the histograms) shows the promotion of the PN and the further reduction of the MES phenotypes, as observed upon *ASCL1* overexpression (black-lined and black-filled bars in the histograms). **c** Intracranial xenografts, induced by *NDRG1*-silenced CSCs and analyzed 3–4 months after transplantation, are significantly smaller than controls (L0605 and L0512; human-specific EGFR staining: brown, ×20). **d** *NDRG1*-silenced CSC-derived xenografts are characterized by the enhancement of PN features, such as the presence of focal areas containing neuronal-like cells organized as rosettes (H&E, ×400), and the increased immunoreactivity for the PN markers Olig2 and PDGFRα (all markers stained in brown, ×400; inset, ×800). **e** Upregulation of MES markers and reduction in PN ones are observed upon NDRG1 transduction of GBM CSCs (qPCR). **f** *NDRG1*-overexpressing tumors display increased MES morphological features, such as the development of areas made up by spindle-shaped cells with large and elongated nuclei (H&E, ×400; inset, ×800), as well as increased MES marker immunoreactivity (all markers stained in brown, ×400)

All together, these results strongly suggest a potential role for reduced NDRG1 expression in mediating the *ASCL1*-induced PN phenotype in CSCs. To test this hypothesis, we silenced *NDRG1* in the PN CSCs that showed the highest endogenous expression (i.e., L0605 and L0512). Decreased *NDRG1* expression by RNAi in two distinct clones of CSCs (clone 31 and 47 for L0605 and clone 31 and 78 for L0512) resulted in significant AKT hypoactivation (Fig. 5a), similar to what observed after *ASCL1* overexpression (Fig. 2b). Interestingly, *NDRG1* silencing in CSCs promoted the transcriptional upregulation of several PN gene classifiers, with the concurrent downregulation of MES-restricted genes (Fig. 5b), as also detected after *ASCL1* overexpression. Most importantly, *NDRG1* silencing severely impaired the in vivo tumorigenic potential of CSCs (Fig. 5c), again recapitulating the effect of *ASCL1* overexpression. Remarkably, *NDRG1* silencing resulted in the upregulation in the expression of ASCL1, Olig2, and PDGFRα (Fig. 5d).

Notably, overexpression of *NDRG1* in PN CSC lines, which were (i) showing endogenous expression of the gene together with ASCL1, i.e., L0512 and L0605, (ii) not showing NDRG1 expression, i.e., L0627, and (iii) showing NDRG1 expression with very low ASCL1 expression (L0125), promoted a significant increase in the expression of MES genes, while decreasing that of PN ones (Fig. 5e). However, *NDRG1* overexpression in vitro did not affect self-renewal, clonogenic ability, multipotency and invasiveness of CSCs (data not shown). Most remarkably, IHC-based analysis on mock- and NDRG1-transduced CSC-derived xenografts indicated the acquisition of MES traits and the concurrent reduction in the PN ones upon NDRG1 overexpression (Fig. 5f and Supplementary Fig. 5b, 5c). Indeed, similar to the phenotype observed upon *ASCL1* silencing (Fig. 4d), *NDRG1*-overexpressing CSC-derived tumors comprised several bundles of large and spindle-shaped cells, with elongated nuclei, typical of mesenchymal differentiation (Fig. 5f and Supplementary Fig. 5b, 5c). Accordingly, the expression of MES markers such as YKL40 (Fig. 5f) as well as CD44, vimentin and MET (Supplementary Fig. 5c) was increased upon *NDRG1* overexpression, whereas the immunoreactivity for PN markers as ASCL1, Olig2 and PDGFRα was less intense than in controls (Fig. 5f and Supplementary Fig. 5b, 5c).

### Enforcing either *NDRG1* or *ASCL1* expression in mesenchymal GBM CSCs induces the acquisition of highly malignant phenotypes in vivo

Thus far, we demonstrated that modulating the expression of either ASCL1 or NDRG1 in PN CSCs promotes the transition from one GBM subgroup to another, thus significantly contributing to the shaping of the transcriptional subgrouping of GBM. Next, we set out to test the same experimental paradigm in MES CSCs.

**Fig. 6:**
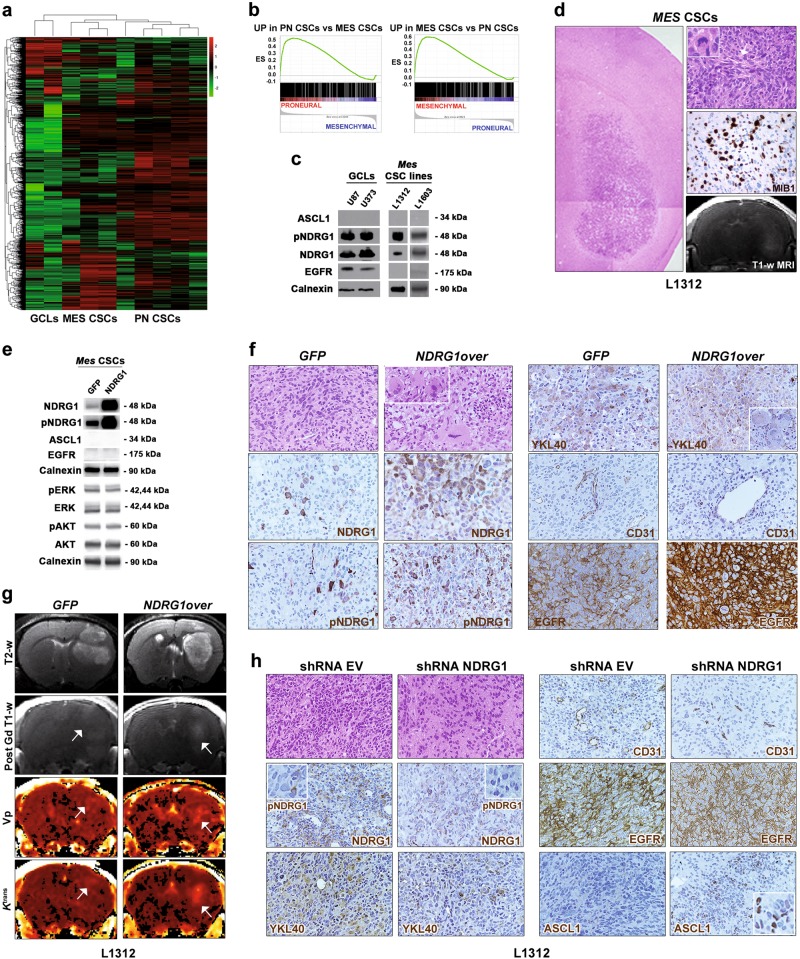
Increasing or decreasing *NDRG1* expression in mesenchymal GBM CSCs induces and impairs the acquisition of malignant phenotypes in vivo. **a** Unsupervised hierarchical whole-transcript expression analysis of GCLs, L1605/L1312 and PN CSCs indicates that L1605/L1312 CSCs are transcriptionally different from PN CSCs. **b** GSEA indicates that genes upregulated in PN CSCs vs. L1605/L1312 CSCs are enriched in the PN subgroup of human GBM, whereas those upregulated in L1605/L1312 CSCs vs. PN CSCs are enriched in the MES subgroup, thus qualifying L1605/L1312 CSCs as MES CSCs. **c** Similar to GCLs, MES CSCs express high level of NDRG1, while not expressing ASCL1 (WB). **d** Ninety days after transplantation, MES CSCs give rise to bulky tumors with well-defined boundaries (left panel, H&E, ×40), which comprise several cells with pleomorphic, spindle-shaped morphology (upper right panel, H&E, ×400, white arrow pointing to spindle-shaped cells) and undergoing mitosis (inset, H&E, ×800). They also display a very high mitotic index (MIB1, middle right panel) and contrast enhancement on T1-weighted MR images (lower right panel). **e** Overexpression of *NDRG1* does not affect the activation of ERK and AKT (WB). **f** Intracranial tumors generated by *NDRG1*-overexpressing CSCs show a highly malignant morphology, with the presence of pleomorphic and atypical cells showing enlarged size (‘bizarre’ cells, H&E, inset, ×800), which are not retrieved in controls. They also show diffused expression of the MES marker YKL40, also in giant cells (inset, ×800), increased angiogenesis and, notably, enhanced EGFR expression (all markers stained in brown, ×400). **g** Anatomic T2 (first row), post-contrast T1 images (second row), *Vp* (third row), and *K*^trans^ maps (fourth row) from DCE MRI indicate that *NDRG1*-overexpressing tumors are endowed with increased contrast enhancement (Post-Gd T1w images, white spot, arrow), enhanced tumor vascularity through quantification of plasma volume (*V*p; orange spots, arrow) and marked vessel permeability (*K*^trans^; orange spots, arrow) as compared to mock tumors. **h** *NDRG1*-silenced CSCs gave rise to tumors that displayed a decrease in MES features and, surprisingly, an increase in the expression of PN markers, such as ASCL1, which is never expressed in MES CSC lines

To this end, we took advantage of two CSC lines, i.e., L1312 and L1603, isolated from a gliosarcoma and a giant cell MES GBM, respectively. The gene expression profile of L1312 and L1603 CSC lines was very different from that of previously microarray-classified PN CSCs and, in fact, the two CSC groups clustered separately (Fig. 6a). To assess whether the gene signatures qualifying L1312 and L1603 CSC lines correlated with a specific human GBM subgroup, we performed GSEA on the same GEO dataset GSE4271 as in Fig. 1. Remarkably, whereas the majority of genes upregulated in PN CSCs were again enriched for the same genes retrieved in PN GBMs (Fig. 6b and Supplementary List 5), most genes overexpressed in L1312 and L1603 CSC lines positively correlated with those upregulated in MES GBMs (Fig. 6b and Supplementary List 6), thus confirming their mesenchymal nature. Both MES CSC lines expressed very high levels of NDRG1 protein, while being negative for ASCL1, i.e., the same pattern observed in MES GCLs (Fig. 6c). Similar to GCLs and different from highly invasive PN CSC lines (Figs. 4b and 5c) [21], MES CSCs gave rise to tumors displaying a poorly invasive growth pattern, high cellular pleomorphism, the presence of bundles of spindle-shaped cells, elevated mitotic index, enhanced angiogenesis and increased contrast uptake on T1-weighted MR images (Fig. 6d). However, as opposed to GCL-derived tumors, MES CSC-induced xenografts developed in vivo with growth kinetics similar to those of tumors derived from PN CSCs (i.e., 80–140 days after transplantation).

*NDRG1* overexpression in MES CSC lines did not affect ERK and AKT activation (Fig. 6e) but resulted in the further promotion of the MES phenotype (Fig. 6f). *NDRG1*-overexpressing CSC-derived xenografts contained highly pleomorphic cells, some of which showed a bizarre and giant cell morphology. These tumors also displayed increased expression of the mesenchymal marker YKL40, enhanced microvascular proliferation and, of note, significant EGFR upregulation (Fig. 6f). Anatomic T1-weighted/T2-weighted and dynamic-contrast enhancement (DCE) MRI analysis indicated a significant increase in post-gadolinium enhancement, tumor vascularity through quantification of plasma volume (*V*p) as well as vessel permeability through calculation of the contrast transfer coefficient (*K*^trans^) in *NDRG1*-overexpressing tumors as compared to mock tumors (Fig. 6g and Supplementary Table 4).

Similar to what detected in PN CSCs (Fig. 5a–d), NDRG1 silencing in MES CSC lines (Supplementary Fig. 6) resulted in the development of tumors that displayed a substantial decrease in MES features, such as reduced cellularity, downregulation of MES markers as YKL40, reduced angiogenesis, and downregulation of EGFR expression (Fig. 6h). Remarkably, increased expression of the PN marker ASCL1, which was never retrieved in MES CSC lines, was detected upon NDRG1 silencing (Fig. 6h), as previously reported in vivo after NDRG1 silencing in PN CSCs (Fig. 5d).

**Fig. 7:**
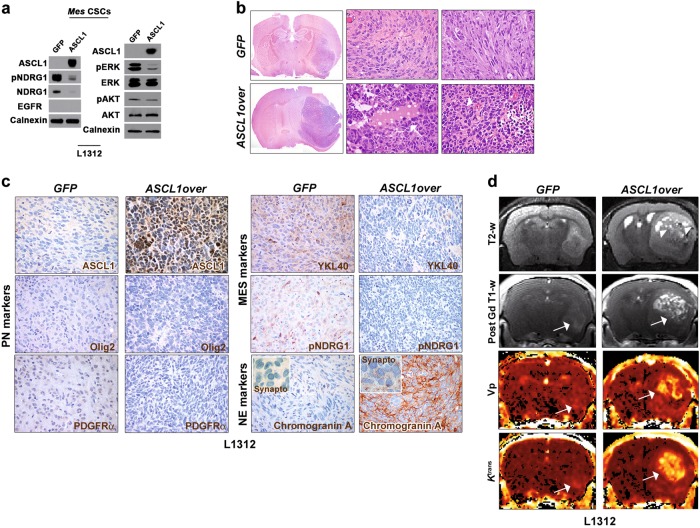
ASCL1 overexpression in mesenchymal GBM CSCs promotes the acquisition of highly malignant neuroendocrine-like features. **a** *ASCL1*-transduced MES CSCs show reduced NDRG1 expression and ERK/AKT activation (WB). **b** *ASCL1*-overexpressing MES CSC-derived tumors acquire highly malignant neuroendocrine-like features such as the presence of follicular structures, lined by cuboidal cells and filled with homogeneous eosinophilic material (colloid) (white arrow; middle lower panel), as well as congested vessels with hemorrhagic infarcts (white arrowheads; left lower panel). **c** Tumors generated by ASCL1-overexpressing MES CSCs do not show induction of PN markers but only reduction in MES markers expression; rather, they display immunoreactivity for neuroendocrine markers as chromogranin A and synaptophysin (inset, ×800). All markers stained in brown, ×400. **d** Anatomic T2 (first row), post-contrast T1 images (second row), *V*p (third row), and *K*^trans^ maps (lower row) from DCE MRI confirms the presence of several secretory areas (arrowheads in T2-w images) as well as increased vessel leakiness, as determined by *K*^trans^ (yellow spots, white arrows), in *ASCL1*-transduced MES CSC-derived tumors

Finally, we assessed whether *ASCL1* expression in MES CSCs might induce the acquisition of a PN phenotype. As observed in PN CSCs, *ASCL1*-transduced MES CSCs showed a decrease in NDRG1 expression and ERK/AKT activation (Fig. 7a). However, tumors induced by *ASCL1*-transduced MES CSCs acquired unexpected malignant features, including the presence of diffuse hemorrhagic necrosis, enhanced angiogenesis and, strikingly, a secretory-like phenotype (Fig. 7b). Specifically, *ASCL1*-overexpressing MES CSC-derived tumors did comprise follicular-like structures, lined by cuboidal cells and filled with homogeneous, eosinophilic, and amorphous material, as well as congested vessels characterized by hemorrhagic infarcts. Accordingly, these same tumors did not show increased expression of PN markers but only reduction in the MES ones (Fig. 7c). Most remarkably, they did display focal expression of the neuroendocrine (NE) markers chromogranin A and synaptophysin (Fig. 7c). These features were reminiscent of poorly differentiated NE-like tumors, closely resembling *ASCL1*-expressing malignant NE small cell lung cancer [28]. Of note, MRI indicated that *ASCL1*-transduced MES CSC-derived tumors showed malignant features such as the presence of several secretory areas, as assessed by high-resolution T2-weighted imaging, as well as increased vessel leakiness, as determined by *K*^trans^ (Fig. 7d and Supplementary Table 5).

## Discussion

To date, the use of transcriptional information to stratify GBM patients remains controversial and no functionally-validated  markers are available for gene expression-based subgroup affiliation [1]. As such, we asked whether subgroup-specific gene classifiers could be pinpointed and endorsed  as subsidiary diagnostic/prognostic markers and potentially actionable vulnerabilities for GBM, as documented for subgroup-restricted molecular mediators, such as *STAT3*, *CEBPB/D*, *TAZ*, *Olig2*, *MLK4*, and *DGKα* [32] [33, 15, 14, 34].

To this end, we focused our interest on the transcription factor *ASCL1*. *Ascl1* is known to enhance the proliferation and expansion of mouse progenitor cells in the ganglionic eminences of the embryonic telencephalon and in the neurogenic regions of the adult mouse brain [35, 36]. Notably, stabilization of Ascl1 by Huwe deletion prevents the return to quiescence of stem cells, thus promoting the contraction of the proliferating stem cell pool [37]. In addition, ASCL1 has a pivotal role as proneural ‘on-target pioneer factor’ in the context of the direct lineage reprogramming of non-neural cells into induced neurons [38].

In agreement with previous findings showing that ASCL1 upregulation through Notch inhibition promotes neuronal differentiation and loss of self-renewal in a subset of GBM CSCs [20], here we present evidence that genetic overexpression of *ASCL1* in CSC lines expressing variable levels of the gene results in efficient neuronal differentiation and almost total glial fate repression.

Most notably, we report that *ASCL1* acts as a switch among different molecular subgroups through the negative regulation of the MES marker *NDRG1*. Indeed, we retrieved a reciprocal trend in the expression of the two genes in our CSC lines, with all PN CSCs expressing ASCL1 but only some expressing also NDRG1. Accordingly, MES CSCs did not express ASCL1, while expressing very high levels of NDRG1, suggesting that a fine-tuned balance in the expression of the two genes may potentially be involved in subgroup specification. *NDRG1* elicits tumor-suppressive and oncogenic functions depending on the tissue/cell type in which it is expressed. In gliomas, data are conflicting. Whereas some reports claim that NDRG1 expression is inversely related with glioma progression from low-grade oligodendrogliomas [39], other studies indicate that the overall survival of GBM patients with upregulation of NDRG1 is reduced as compared to patients with intermediate or low expression of the gene, thus proposing NDRG1 acting mainly as an oncogene in GBM [40, 41]. Again, studies that exploited traditional GCLs as U87 as model system reported that enforced NDRG1 expression promotes a decrease in tumor vascularization and resistance to anti-angiogenic treatment [42]. Here, we provide first evidence that, in GBM CSCs, *NDRG1* is directly and negatively regulated by ASCL1, and, most importantly, that *NDRG1* overexpression and silencing in CSCs enhances and decreases the MES phenotype, respectively.

Another interesting aspect related to *ASCL1* function in CSCs is its repressive activity exerted not only on *NDRG1* but also on *EGFR* expression and ERK activation. In fact, this molecular pattern is reminiscent of the so-called PN-specific G-CIMP + phenotype, which, when induced by exogenous expression of the mutated isocitrate dehydrogenase 1 *IDH1-R132H* gene, results in reduced EGFR protein expression as well as impaired pERK accumulation [43].

As opposed to Dirks’ lab findings [20] and ours, ASCL1 has also been reported to promote the tumorigenicity of GBM CSCs through the activation of Wnt signaling [29]. One possible explanation for this discrepancy might rely on the known biphasic physiological role of ASCL1, which enhances proliferation and induces differentiation depending on its oscillatory temporal expression [44]. Indeed, transient upregulation of Ascl1 before cell division not only results in neuronal differentiation, but also promotes cell proliferation in progenitors. As such, the different GBM CSC culture conditions in use in distinct labs, by influencing the frequency of highly proliferative vs. more committed progenitors and, as such, the analysis of ASCL1 function (in this case by RNAi), might yield opposite results depending on the nature of the cells under scrutiny.

Most significantly, our findings indicate that, similar to the physiological role played by ASCL1 as a repressor of mesendoderm induction [45] and of stem/progenitor fates [46, 47], ASCL1 simultaneously activates and represses alternative GBM gene expression programs, as those leading to the PN and MES phenotypes, respectively.

Overall, we report that the fine balance between the expression of ASCL1 and NDRG1 in GBM CSCs is required to regulate the switch between distinct molecular phenotypes and to modulate PMT. Notably, the pivotal role of ASCL1 in determining GBM subgroups strongly support the view of the PN phenotype as the ground state of GBM from which further malignant evolution takes place [11]. Given that deregulation of the interplay between ASCL1 and NDRG1 might be considered as an initiating molecular event involved in the progression from PN to MES subtypes, a better understanding of the mechanisms underlying gene expression changes (e.g., microenvironment-induced gene repression and derepression, etc.) may lay the foundation for the identification of therapeutic strategies specifically targeted to restrain PMT and GBM evolution.

In addition, we provide evidence that ASCL1-mediated pro-differentiation therapeutic strategies need to be carefully tuned and should take into consideration the nature of the tumor cell to differentiate. In fact, alternative epigenetic landscapes might be driven by ASCL1 expression in PN and MES CSCs and may be likely responsible for the dramatically divergent outcomes, as shown by the induction of highly malignant NE phenotypes by ASCL1 overexpression in MES CSCs.

## Supplementary information

Supplementary Information
