# Supplementary material for: The proneural gene ASCL1 governs the transcriptional subgroup affiliation in glioblastoma stem cells by directly repressing the mesenchymal gene NDRG1
Source: Cell Death Differ. 2018 Dec 11;26(9):1813–31. doi: 10.1038/s41418-018-0248-7 (PMC6748080; doi:10.1038/s41418-018-0248-7)
Supplement: Supplementary file 1 — Supplementary Information [file 41418_2018_248_MOESM1_ESM.pdf]

Supplementary Information

The proneural gene ASCL1 governs the transcriptional subgroup affiliation in glioblastoma stem cells by directly repressing the mesenchymal gene NDRG1

Narayanan et al., submitted to *Cell Death & Differentiation*, 2018

Supplementary Figures

Supplementary Fig. 1 - *ASCL1* overexpression and silencing by lentiviral transduction in GBM CSCs promotes neuronal differentiation and lineage switch.

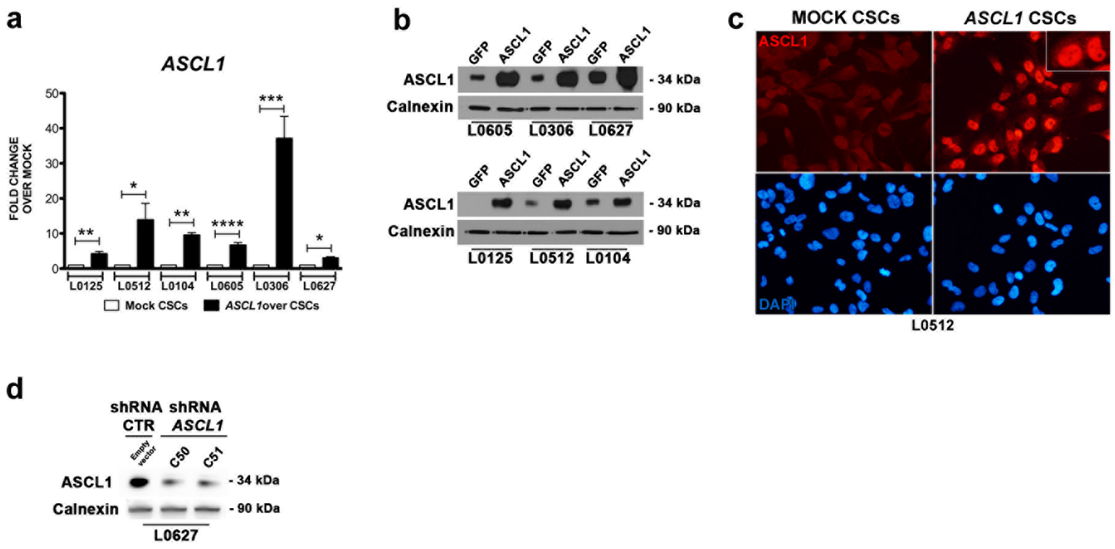

(a) Enhanced *ASCL1* mRNA expression in *ASCL1*-transduced CSCs and GCLs as compared to mock-transduced controls. Fold increase of *ASCL1* mRNA was calculated with respect to matched mock samples (qPCR).
(b) Increased ASCL1 protein expression in *ASCL1*-transduced CSCs and GCLs as compared to mock-transduced controls (WB).
(c) ICC highlights the proper nuclear localization of the exogenous ASCL1 protein in *ASCL1*-transduced CSCs (ASCL1, red; DAPI, light blue; magnification 400x, inset 1000x).
(d) Reduced ASCL1 expression upon *ASCL1* lentiviral-mediated RNAi (Mission shRNAs clone 50 and 51). CTR: empty vector. Representative cell line: L0627.

Supplementary Fig. 2 - ASCL1 overexpression promotes neuronal differentiation of GBM CSCs.

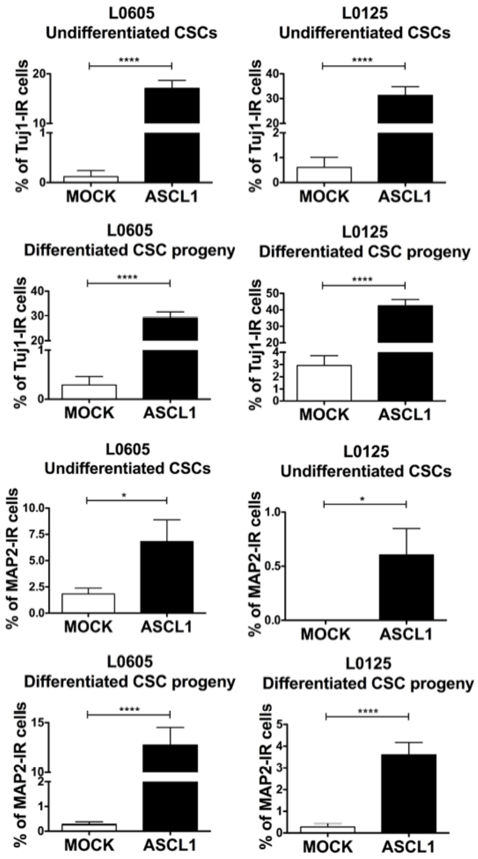

Quantification of the frequency of Tuj1- and GFAP-IR cells after *ASCL1* overexpression (normalized over DAPI-stained nuclei).

Supplementary Fig. 3 - NDRG1 expression associates with high grade staging and IDH1 wildtype mutational status, is not regulated by mTORC2 but is regulated directly by ASCL1 as other known transcriptional targets.

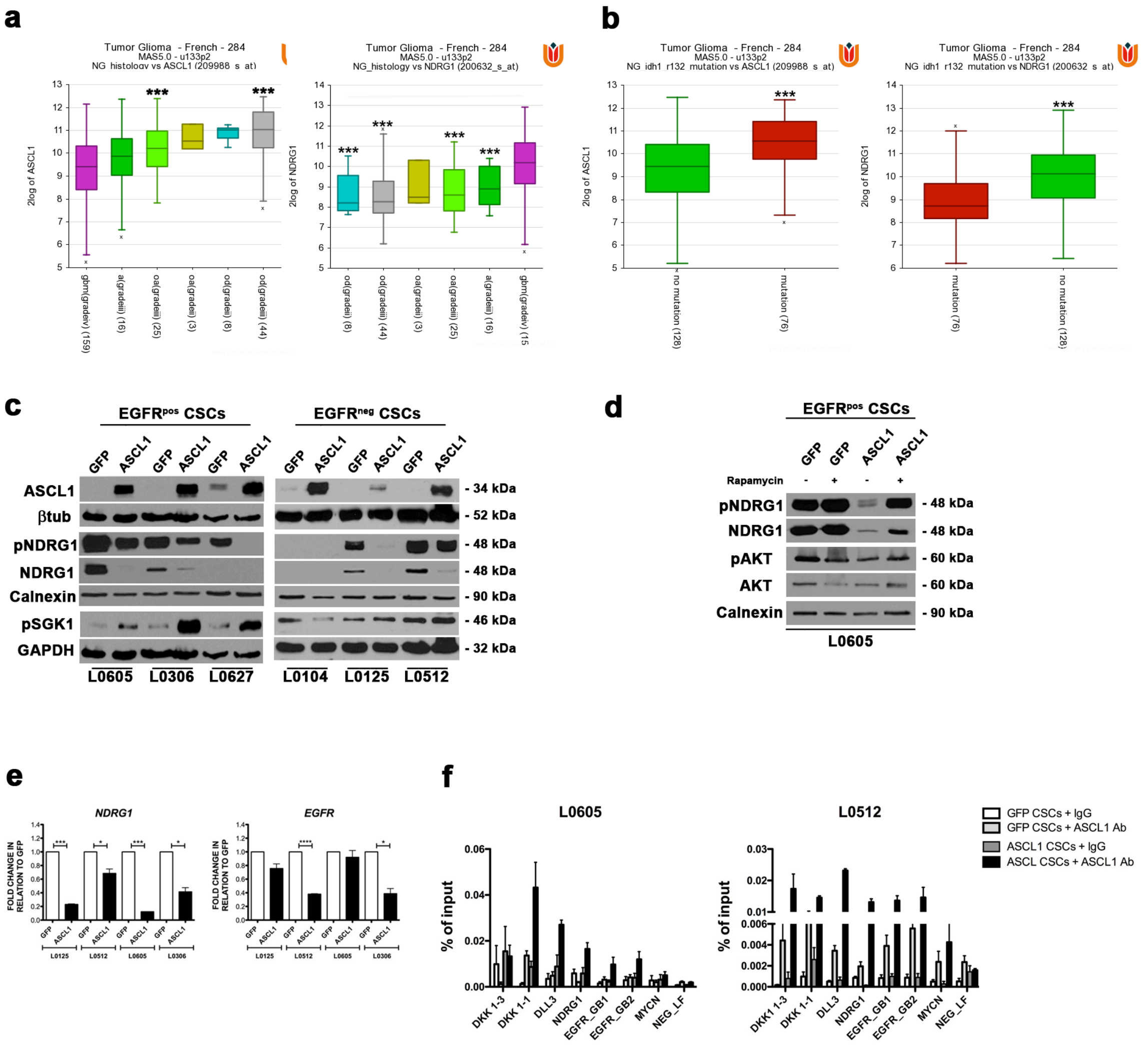

(a-b) *NDRG1* expression is significantly increased in high grade gliomas when compared to low grade (astrocytomas, a; oligoastrocytomas, oa; oligodendroglioma, od; glioblastoma, gbm) as well as in IDH1 wild type vs IDH1 mutant gliomas. *ASCL1* expression shows the opposite trend. R2 analysis on Tumor Glioma French dataset comprising 284 samples.

(c) The pattern of activation of the NDRG1 upstream activator pSGK1 does not correlate with that of pNDRG1.

(d) mTORC2 inhibition by long-term treatment with rapamycin in *ASCL1*-overexpressing GBM CSCs only partially rescues NDRG1 expression.

(e) The expression pattern of *NDRG1* and *EGFR* primary transcripts by qPCR mirrors that of the corresponding proteins.

(f) Binding of ASCL1 is detected within the promoter and enhancer of *DLL3* and *DKK1* genes, respectively.

Supplementary Fig. 4 – NDRG1 expression is inversely related to ASCL1 expression in GBM specimens from the TCGA

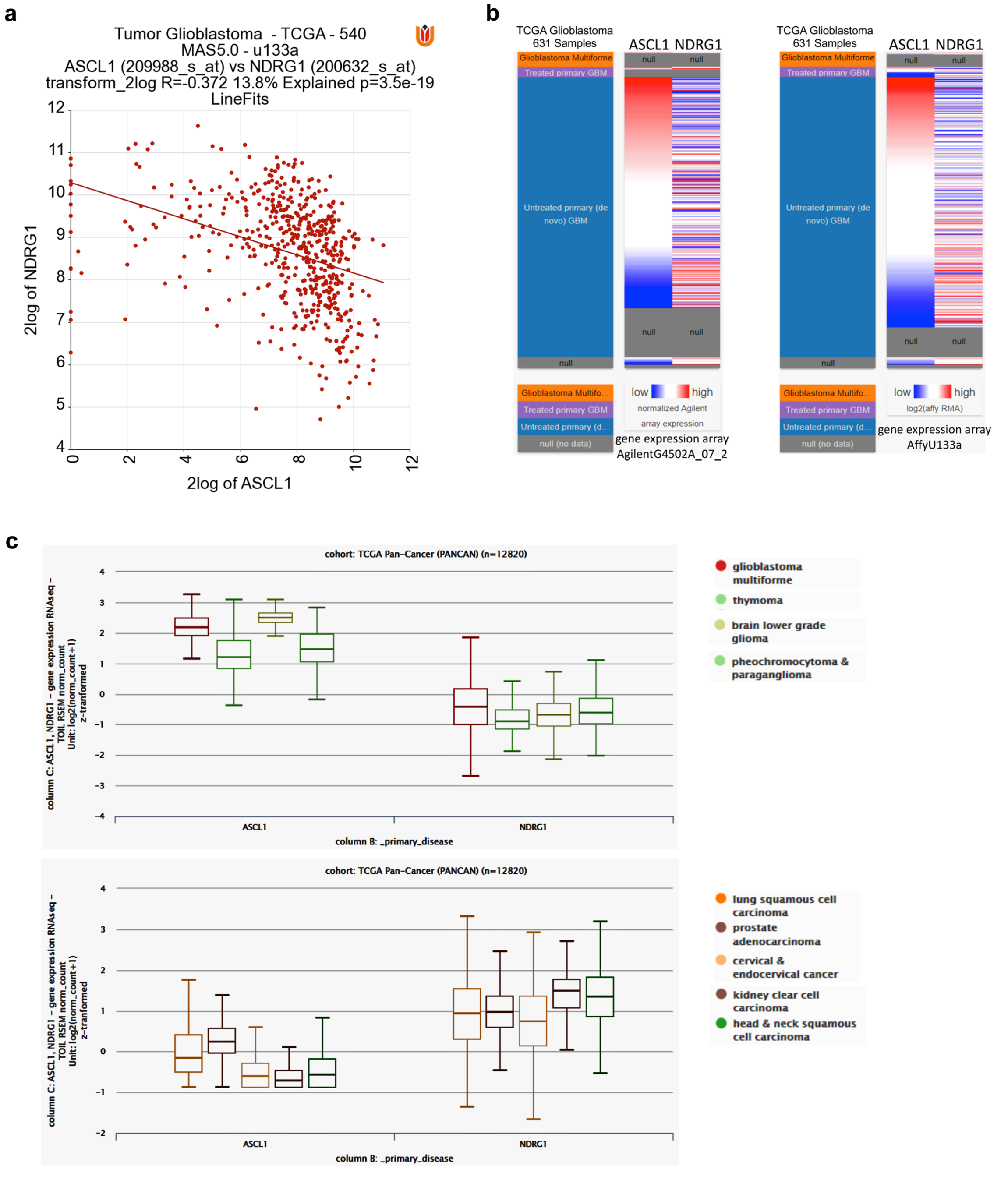

(a) Pearson correlation analysis on human GBM samples from the TCGA data set highlights the significant anticorrelation of ASCL1 and NDRG1 in terms of gene expression.
(b) Heatmaps of microarray data indicate that high expression of ASCL1 correlates with low expression of NDRG1, thus supporting its role of repressor.
(c) In tumors with high expression of ASCL1, NDRG1 is on average lowly expressed (upper panel). The opposite is also true, as high expression of NDRG1 correlates with lower expression of ASCL1 (lower panel).

Supplementary Fig. 5 - Modulation of the expression of *ASCL1* and *NDRG1* in GBM CSCs affects their tumorigenic behavior and subgroup affiliation.

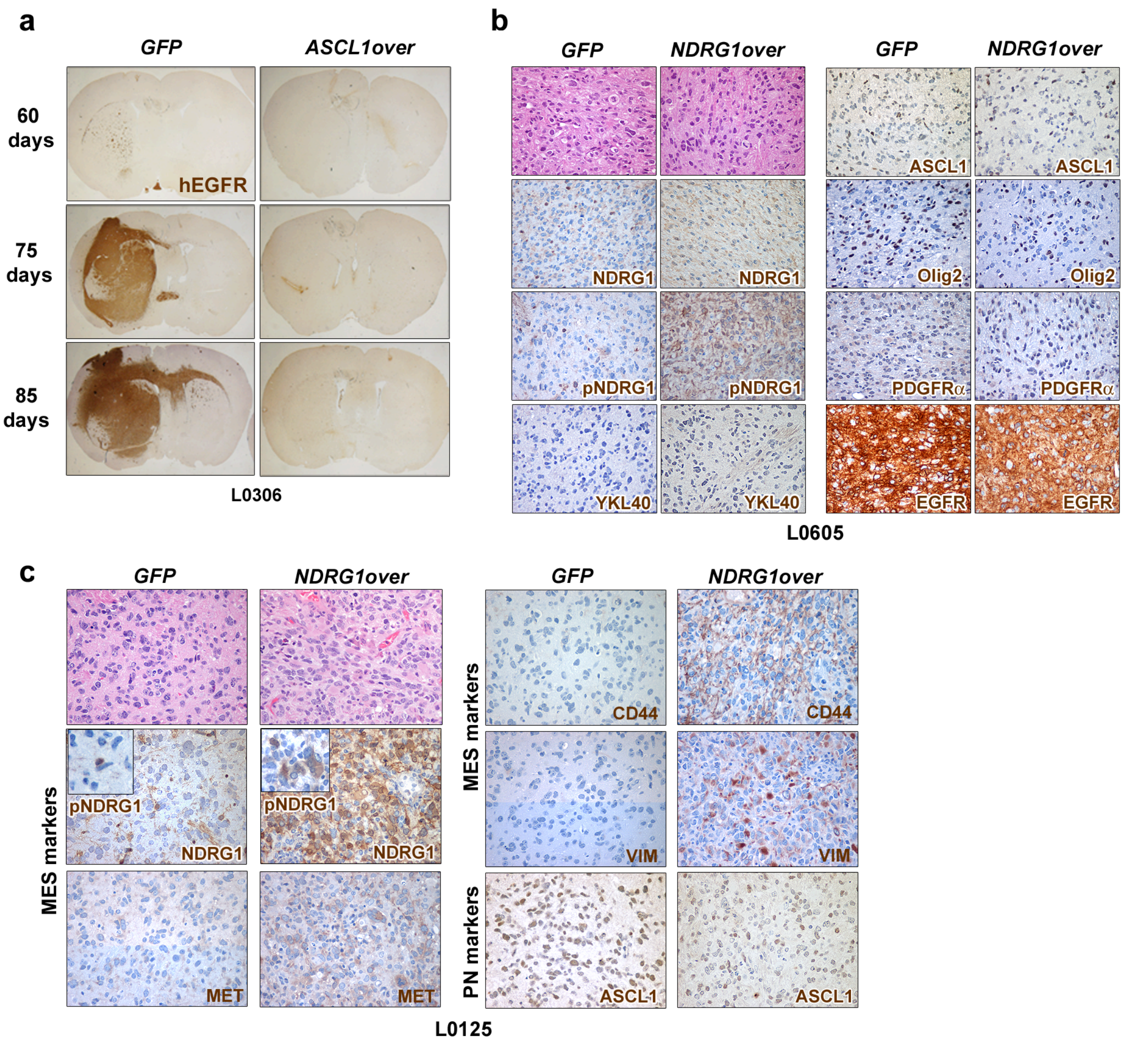

(a) *ASCL1*-overexpressing L0306 CSCs failed to form tumors even at the latest time point assessed for controls. Human-specific EGFR staining: brown, 20x.

(b-c) *NDRG1*-overexpressing L0605 and L0125 CSC-derived tumors displayed increased MES morphological features, such as the development of areas made up by spindle-shaped cells with large and elongated nuclei (H&E, 400x), as well as increased MES marker immunoreactivity at the expense of the expression of PN markers (all markers stained in brown, 400x).

Supplementary Fig. 6 - Modulation of the expression of *ASCL1* and *NDRG1* in GBM CSCs affects their tumorigenic behavior and subgroup affiliation.

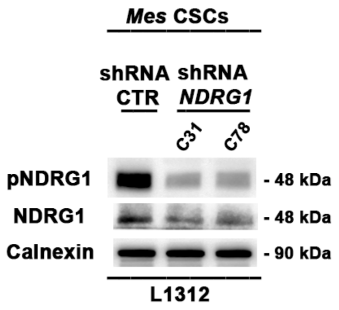

Efficient *NDRG1* silencing in MES CSC lines was obtained by different shRNA clones (Mission, Sigma-Aldrich)

Supplementary Tables

Supplementary Table 1 – Gene set enrichment analysis (GSEA) report for the two distinct gene sets generated through the comparison between CSCs vs GCLs, when tested on ranked lists of proneural and mesenchymal genes.

	Gene set	Size	Compa-rison	Enrich-ment in pheno-type	ES	NES	Nom p-val	FDR q-val	FWER p-val	Rank At Max	Leading edge
1	Genes UP in CSCs vs GCLs	557	PN *vs.* MES	PN	0.50	1.27	0.165	***0.165***	0.086	2588	tags=35%, list=12%, signal=39%
2	Genes UP in GCLs vs CSCs	424	MES *vs.* PN	MES	0.71	1.60	0.002	***0.002***	0.001	1867	tags=43%, list=8%, signal=46%

A significant FDR q-value (> 0.25) has been retrieved for both comparisons (highlighted in bold italic).

Supplementary Table 2 – Gene set enrichment analysis (GSEA) report for the distinct gene sets generated through the comparison between CSCs *vs* GCLs, when tested on ranked lists of proneural, proliferative and mesenchymal genes.

	**Gene set**	**Size**	**Compa-rison**	**Enrich-ment in pheno-type**	**ES**	**NES**	**Nom p-val**	**FDR q-val**	**FWER p-val**	**Rank At Max**	**Leading edge**
1	Genes UP in CSCs vs GCLs	557	PN *vs.* PROL	PN	0.50	1.29	0.149	***0.149***	0.074	3045	tags=38%, list=14%, signal=43%
2	Genes UP in CSCs vs GCLs	557	MES *vs.* PROL	MES	0.28	0.88	0.901	0.901	0.437	2972	tags=24%, list=13%, signal=26%
3	Genes UP in GCLs vs CSCs	424	PN *vs.* PROL	PROL	-0.48	-1.23	0.181	***0.181***	0.090	3390	tags=32%, list=15%, signal=37%
4	Genes UP in GCLs vs CSCs	424	MES *vs.* PROL	MES	0.62	1.46	0.041	***0.041***	0.021	2971	tags=45%, list=13%, signal=50%

A significant FDR q-value (> 0.25) has been retrieved for comparison 1, 3 and 4 (highlighted in bold italic).

Supplementary Table 3 – Gene set enrichment analysis (GSEA) report for the two distinct gene sets generated through the comparison between ASCL1^{high}/NDRG1^{low} or ASCL1^{high}/NDRG1^{high} CSCs vs GCLs, when tested on ranked lists of proneural and mesenchymal genes.

	**Gene set**	**Size**	**Compa-rison**	**Enrich-ment in pheno-type**	**ES**	**NES**	**Nom p-val**	**FDR q-val**	**FWER p-val**	**Rank at max**	**Leading edge**
1	Genes UP in ASCL1^{high}/NDRG1^{low} CSCs vs GCLs	205	PN *vs.* MES	PN	0.61	**1.40**	0.067	***0.067***	0.033	2475	tags=42%, list=11%, signal=47%
2	Genes UP in ASCL1^{high}/NDRG1^{high} CSCs vs GCLs	470	PN *vs.* MES	PN	0.46	**1.19**	0.236	***0.236***	0.117	2517	tags=30%, list=11%, signal=34%

A significant FDR q-value (> 0.25) has been retrieved for both comparisons (highlighted in bold italic). A lower NES in the proneural subgroup was observed for genes upregulated in comparison 2 as compared to those in comparison 1, in line with a positive trend in the level of enrichment detected in the mesenchymal subgroup.

Supplementary Table 4 – Analysis of perfusion MRI parameters in mock- and *NDRG1*-transduced MES L1312 CSC-derived tumors.

Samples	Volume in post-Gadolinium T1	rVp_{median}	$rVp_{90\%tile}$	rK^{trans}_{median}	$rK^{trans}_{90\%tile}$
**Mock**	21.87 (15.90-27.83)	**1.10 (0.82-1.39)**	**2.34 (1.68-3.00)**	**1.49 (1.28-1.69)**	**4.77 (4.00-5.53)**
**NDRG1**	65.11 (20.46-109.76)	**3.82 (2.29-5.34)**	**10.81 (5.46-16.15)**	**38.74 (11.13-66.35)**	**97.32 (45.79-148.85)**

All the values of the plasma volume (Vp) and contrast transfer coefficient (K^{trans}) parameters were normalized over the values measured on the contralateral hemisphere.

Supplementary Table 5 – Analysis of perfusion MRI parameters in mock- and *ASCL1*-transduced MES L1312 CSC-derived tumors

Samples	Volume in post-Gadolinium T1	rVp_{median}	$rVp_{90\%tile}$	rK^{trans}_{median}	$rK^{trans}_{90\%tile}$
**Mock**	19.04 (13.59-24.49)	1.11 (0.77-1.46)	2.30 (1.95-2.65)	**2.06 (1.99-2.12)**	**7.30 (5.66-8.93)**
**ASCL1**	49.67 (13.93-85.40)	1.63 (1.26-1.99)	4.07 (2.24-5.90)	**12.68 (6.25-19.11)**	**43.19 (12.10-74.28)**

All the values of the plasma volume (Vp) and contrast transfer coefficient (K^{trans}) parameters were normalized over the values measured on the contralateral hemisphere.

Supplementary Lists

Supplementary List 1 - List of the 557 top ranking genes upregulated in CSCs *vs*. GCLs and selected based on an Adjusted P value < 0.01 e log2 Fold Change > 1 for Gene Set Enrichment Analysis (GSEA) in the comparison PN *vs*. MES.

PROBE	GENE SYMBOL	GENE TITLE	RANK IN GENE LIST	RANK METRIC SCORE	RUNNING ES	CORE ENRICHMENT
**219537_x_at**	DLL3	delta-like 3 (Drosophila)	13	0.6549095511436462	0.008160949	Yes
**209839_at**	DNM3	dynamin 3	15	0.6499274969100952	0.016807597	Yes
**209987_s_at**	ASCL1	achaete-scute complex-like 1 (Drosophila)	18	0.6169173717498779	0.024966713	Yes
**207103_at**	KCND2	potassium voltage-gated channel, Shal-related subfamily, member 2	19	0.6155011653900146	0.03319894	Yes
**221623_at**	BCAN	brevican	21	0.6073176860809326	0.04127569	Yes
**209988_s_at**	ASCL1	achaete-scute complex-like 1 (Drosophila)	23	0.6053429841995239	0.04932603	Yes
**213824_at**	OLIG2	oligodendrocyte lineage transcription factor 2	29	0.5745991468429565	0.05678106	Yes
**219196_at**	SCG3	secretogranin III	31	0.5723041892051697	0.06438951	Yes
**209815_at**	PTCH1	patched homolog 1 (Drosophila)	38	0.547568678855896	0.07143699	Yes
**213825_at**	OLIG2	oligodendrocyte lineage transcription factor 2	39	0.5462126731872559	0.0787425	Yes
**205638_at**	BAI3	brain-specific angiogenesis inhibitor 3	53	0.5201330780982971	0.08510083	Yes
**203485_at**	RTN1	reticulon 1	56	0.5195413827896118	0.091957554	Yes
**218829_s_at**	CHD7	chromodomain helicase DNA binding protein 7	66	0.5038008689880371	0.09828156	Yes
**210222_s_at**	RTN1	reticulon 1	70	0.4944189488887787	0.10475625	Yes
**219107_at**	BCAN	brevican	74	0.48157739639282227	0.11105919	Yes
**219643_at**	LRP1B	low density lipoprotein-related protein 1B (deleted in tumors)	79	0.47301268577575684	0.117201544	Yes
**205316_at**	SLC15A2	Solute carrier family 15 (H+/peptide transporter), member 2	148	0.39233365654945374	0.11931906	Yes
**210015_s_at**	MAP2	microtubule-associated protein 2	150	0.389330118894577	0.124480255	Yes
**213609_s_at**	SEZ6L	seizure related 6 homolog (mouse)-like	157	0.3846593499183655	0.12934884	Yes
**91920_at**	BCAN	brevican	166	0.3796325623989105	0.13405815	Yes
**203849_s_at**	KIF1A	kinesin family member 1A	190	0.3620590567588806	0.13784198	Yes
**206408_at**	LRRTM2	leucine rich repeat transmembrane neuronal 2	208	0.3538261651992798	0.14179188	Yes
**204913_s_at**	SOX11	SRY (sex determining region Y)-box 11	214	0.3492409586906433	0.14623278	Yes
**204273_at**	EDNRB	endothelin receptor type B	220	0.3460276126861572	0.15063071	Yes
**204915_s_at**	SOX11	SRY (sex determining region Y)-box 11	227	0.34134334325790405	0.15491997	Yes
**205152_at**	SLC6A1	solute carrier family 6 (neurotransmitter transporter, GABA), member 1	233	0.3395020663738251	0.1592306	Yes
**222301_at**	C1ORF61	Chromosome 1 open reading frame 61	240	0.33588707447052	0.16344687	Yes
**219415_at**	TTYH1	tweety homolog 1 (Drosophila)	242	0.33456602692604065	0.16787562	Yes

**221272_s_at**	C1ORF21	chromosome 1 open reading frame 21 /// chromosome 1 open reading frame 21	263	0.32542166113853455	0.17130752	Yes
**202454_s_at**	ERBB3	v-erb-b2 erythroblastic leukemia viral oncogene homolog 3 (avian)	275	0.3210487365722656	0.17509519	Yes
**209867_s_at**	LPHN3	latrophilin 3	280	0.31896060705184937	0.17917714	Yes
**210198_s_at**	PLP1	proteolipid protein 1 (	285	0.31781232357025146	0.1832437	Yes
**206701_x_at**	EDNRB	endothelin receptor type B	287	0.3174482583999634	0.1874435	Yes
**214607_at**	PAK3	p21 (CDKN1A)-activated kinase 3	290	0.31662920117378235	0.1915863	Yes
**203264_s_at**	ARHGEF 9	Cdc42 guanine nucleotide exchange factor (GEF) 9	302	0.31254884600639343	0.19526029	Yes
**209618_at**	CTNND2	catenin (cadherin-associated protein), delta 2 (neural plakophilin-related arm-repeat protein)	304	0.31179970502853394	0.19938454	Yes
**209243_s_at**	PEG3	paternally expressed 3	311	0.3093435764312744	0.20324579	Yes
**206915_at**	NKX2-2	NK2 transcription factor related, locus 2 (Drosophila)	313	0.30857038497924805	0.20732684	Yes
**213768_s_at**	ASCL1	achaete-scute complex-like 1 (Drosophila)	318	0.3064843416213989	0.21124192	Yes
**221088_s_at**	PPP1R9A	protein phosphatase 1, regulatory (inhibitor) subunit 9A	328	0.30357518792152405	0.21488793	Yes
**219743_at**	HEY2	hairy/enhancer-of-split related with YRPW motif 2	333	0.3015672266483307	0.21873723	Yes
**203129_s_at**	KIF5C	kinesin family member 5C	340	0.2999977469444275	0.22247349	Yes
**213032_at**	NFIB	nuclear factor I/B	343	0.29910898208618164	0.22638196	Yes
**209460_at**	ABAT	4-aminobutyrate aminotransferase	365	0.29391294717788696	0.22934642	Yes
**209234_at**	KIF1B	kinesin family member 1B	372	0.29099565744400024	0.23296227	Yes
**214043_at**	PTPRD	Protein tyrosine phosphatase, receptor type, D	385	0.2865099310874939	0.23624197	Yes
**218902_at**	NOTCH1	Notch homolog 1, translocation-associated (Drosophila)	387	0.28616032004356384	0.24002329	Yes
**215807_s_at**	PLXNB1	plexin B1	390	0.2852330803871155	0.24374618	Yes
**204743_at**	TAGLN3	transgelin 3	394	0.2834824323654175	0.24739963	Yes
**213362_at**	PTPRD	Protein tyrosine phosphatase, receptor type, D	398	0.28284069895744324	0.25104448	Yes
**205103_at**	C1ORF61	chromosome 1 open reading frame 61	403	0.28192245960235596	0.25463104	Yes
**206692_at**	KCNJ10	potassium inwardly-rectifying channel, subfamily J, member 10	409	0.2801637351512909	0.25814804	Yes
**204914_s_at**	SOX11	SRY (sex determining region Y)-box 11	431	0.27427634596824646	0.26084986	Yes
**219564_at**	KCNJ16	potassium inwardly-rectifying channel, subfamily J, member 16	439	0.27215638756752014	0.26416773	Yes
**207781_s_at**	ZNF711	zinc finger protein 711	454	0.26772165298461914	0.26710406	Yes
**214680_at**	NTRK2	neurotrophic tyrosine kinase, receptor, type 2	460	0.26689600944519043	0.27044362	Yes
**215164_at**	TCF4	Transcription factor 4	467	0.26542702317237854	0.2737175	Yes
**204271_s_at**	EDNRB	endothelin receptor type B	477	0.26374703645706177	0.27683082	Yes
**205184_at**	GNG4	guanine nucleotide binding protein (G protein), gamma 4	478	0.2635939121246338	0.28035635	Yes
**209459_s_at**	ABAT	4-aminobutyrate aminotransferase	486	0.2625848352909088	0.28354618	Yes
**212816_s_at**	CBS	cystathionine-beta-synthase	488	0.2622484862804413	0.2870077	Yes
**213033_s_at**	NFIB	nuclear factor I/B	516	0.2557018995285034	0.2891849	Yes

**203130_s_at**	KIF5C	kinesin family member 5C	520	0.2547111511230469	0.29245356	Yes
**213996_at**	YPEL1	yippee-like 1 (Drosophila)	534	0.2510828375816345	0.29521337	Yes
**209866_s_at**	LPHN3	latrophilin 3	537	0.2507722079753876	0.29847535	Yes
**205433_at**	BCHE	butyrylcholinesterase	558	0.246237114071846	0.3008482	Yes
**213228_at**	PDE8B	phosphodiesterase 8B	577	0.24149632453918457	0.30324966	Yes
**209985_s_at**	ASCL1	achaete-scute complex-like 1 (Drosophila)	622	0.23341403901576996	0.30434632	Yes
**209293_x_at**	ID4	inhibitor of DNA binding 4, dominant negative helix-loop-helix protein	630	0.23224972188472748	0.30713043	Yes
**206144_at**	MAGI1	membrane associated guanylate kinase, WW and PDZ domain containing 1	636	0.23187875747680664	0.3100016	Yes
**203037_s_at**	MTSS1	metastasis suppressor 1	644	0.23062153160572052	0.31276396	Yes
**205938_at**	PPM1E	protein phosphatase 1E (PP2C domain containing)	649	0.23015613853931427	0.31565815	Yes
**209504_s_at**	PLEKHB1	pleckstrin homology domain containing, family B (evectins) member 1	666	0.22786666452884674	0.31796938	Yes
**213029_at**	NFIB	nuclear factor I/B	676	0.2266375869512558	0.32058638	Yes
**211484_s_at**	DSCAM	Down syndrome cell adhesion molecule	681	0.22623206675052643	0.32342806	Yes
**219701_at**	TMOD2	tropomodulin 2 (neuronal)	683	0.22592082619667053	0.3264037	Yes
**205794_s_at**	NOVA1	neuro-oncological ventral antigen 1	684	0.22585149109363556	0.32942444	Yes
**209617_s_at**	CTNND2	catenin (cadherin-associated protein), delta 2 (neural plakophilin-related arm-repeat protein)	690	0.22536858916282654	0.33220857	Yes
**209292_at**	ID4	Inhibitor of DNA binding 4, dominant negative helix-loop-helix protein	721	0.22115598618984222	0.33378565	Yes
**205822_s_at**	HMGCS1	3-hydroxy-3-methylglutaryl-Coenzyme A synthase 1 (soluble)	756	0.21532808244228363	0.33510068	Yes
**221796_at**	NTRK2	neurotrophic tyrosine kinase, receptor, type 2	764	0.21460260450839996	0.33764878	Yes
**211467_s_at**	NFIB	nuclear factor I/B	765	0.21442680060863495	0.3405167	Yes
**212482_at**	RMND5A	required for meiotic nuclear division 5 homolog A (S. cerevisiae)	771	0.21377712488174438	0.3431458	Yes
**219521_at**	B3GAT1	beta-1,3-glucuronyltransferase 1 (glucuronosyltransferase P)	788	0.21088744699954987	0.34522992	Yes
**218857_s_at**	ASRGL1	asparaginase like 1	798	0.21004018187522888	0.34762493	Yes
**216850_at**	SNRPN	small nuclear ribonucleoprotein polypeptide N	813	0.20797179639339447	0.34976214	Yes
**213197_at**	ASTN1	astrotactin 1	819	0.20723164081573486	0.35230368	Yes
**209242_at**	PEG3	paternally expressed 3	824	0.20672908425331116	0.35488454	Yes
**203080_s_at**	BAZ2B	bromodomain adjacent to zinc finger domain, 2B	825	0.2063278704881668	0.35764414	Yes
**215028_at**	SEMA6A	sema domain, transmembrane domain (TM), and cytoplasmic domain, (semaphorin) 6A	829	0.20583657920360565	0.3602591	Yes
**202517_at**	CRMP1	collapsin response mediator protein 1	872	0.20148856937885284	0.3610208	Yes
**211494_s_at**	SLC4A4	solute carrier family 4, sodium bicarbonate cotransporter, member 4	875	0.2013636827468872	0.36362195	Yes

**211685_s_at**	NCALD	neurocalcin delta /// neurocalcin delta	881	0.2008490264415741	0.36607814	Yes
**213108_at**	CAMK2A	calcium/calmodulin-dependent protein kinase (CaM kinase) II alpha	899	0.19920958578586578	0.36796007	Yes
**221750_at**	HMGCS1	3-hydroxy-3-methylglutaryl-Coenzyme A synthase 1 (soluble)	912	0.19821500778198242	0.37005883	Yes
**203853_s_at**	GAB2	GRB2-associated binding protein 2	915	0.19805459678173065	0.37261572	Yes
**203908_at**	SLC4A4	solute carrier family 4, sodium bicarbonate cotransporter, member 4	931	0.19577796757221222	0.3745438	Yes
**213283_s_at**	SALL2	sal-like 2 (Drosophila)	947	0.19364716112613678	0.3764434	Yes
**205317_s_at**	SLC15A2	solute carrier family 15 (H+/peptide transporter), member 2	952	0.19335025548934937	0.3788453	Yes
**206731_at**	CNKSR2	connector enhancer of kinase suppressor of Ras 2	954	0.19326157867908478	0.3813841	Yes
**203631_s_at**	GPRC5B	G protein-coupled receptor, family C, group 5, member B	978	0.19085897505283356	0.38287818	Yes
**213236_at**	SASH1	SAM and SH3 domain containing 1	979	0.19082601368427277	0.38543046	Yes
**206527_at**	ABAT	4-aminobutyrate aminotransferase	1008	0.18798017501831055	0.38665587	Yes
**206462_s_at**	NTRK3	neurotrophic tyrosine kinase, receptor, type 3	1015	0.1877857893705368	0.3888913	Yes
**202548_s_at**	ARHGEF 7	Rho guanine nucleotide exchange factor (GEF) 7	1022	0.18737341463565826	0.39112124	Yes
**207620_s_at**	CASK	calcium/calmodulin-dependent serine protein kinase (MAGUK family)	1034	0.18620085716247559	0.39310533	Yes
**207437_at**	NOVA1	neuro-oncological ventral antigen 1	1058	0.18462538719177246	0.39451602	Yes
**202967_at**	GSTA4	glutathione S-transferase A4	1062	0.18428051471710205	0.39684266	Yes
**216047_x_at**	SEZ6L	seizure related 6 homolog (mouse)-like	1073	0.18303874135017395	0.3988305	Yes
**216933_x_at**	APC	adenomatosis polyposis coli	1193	0.1723545491695404	0.3956584	Yes
**203526_s_at**	APC	adenomatosis polyposis coli	1201	0.17193534970283508	0.39763582	Yes
**208986_at**	TCF12	transcription factor 12 (HTF4, helix-loop-helix transcription factors 4)	1202	0.17190849781036377	0.39993507	Yes
**207151_at**	ADCYAP 1R1	adenylate cyclase activating polypeptide 1 (pituitary) receptor type I	1204	0.1718164086341858	0.40218705	Yes
**207873_x_at**	SEZ6L	seizure related 6 homolog (mouse)-like	1208	0.17126499116420746	0.4043396	Yes
**206083_at**	BAI1	brain-specific angiogenesis inhibitor 1	1211	0.17112517356872559	0.40653634	Yes
**208552_at**	GRIK4	glutamate receptor, ionotropic, kainate 4	1240	0.16913606226444244	0.4075097	Yes
**202547_s_at**	ARHGEF 7	Rho guanine nucleotide exchange factor (GEF) 7	1246	0.1686599850654602	0.40953538	Yes
**210383_at**	SCN1A	sodium channel, voltage-gated, type I, alpha	1248	0.1684713065624237	0.41174263	Yes
**212486_s_at**	FYN	FYN oncogene related to SRC, FGR, YES	1261	0.16758713126182556	0.41343176	Yes
**211899_s_at**	TRAF4	TNF receptor-associated factor 4	1262	0.1675788015127182	0.4156731	Yes
**202986_at**	ARNT2	aryl-hydrocarbon receptor nuclear translocator 2	1288	0.16556619107723236	0.4167368	Yes

**206330_s_at**	SHC3	SHC (Src homology 2 domain containing) transforming protein 3	1293	0.1653205156326294	0.41876385	Yes
**209583_s_at**	CD200	CD200 molecule	1295	0.16524042189121246	0.42092788	Yes
**205613_at**	SYT17	synaptotagmin XVII	1338	0.16293801367282867	0.421174	Yes
**205593_s_at**	PDE9A	phosphodiesterase 9A	1348	0.1617581844329834	0.42292324	Yes
**216456_at**	PCDH9	Protocadherin 9	1349	0.1616557538509369	0.42508534	Yes
**200884_at**	CKB	creatine kinase, brain	1366	0.16090738773345947	0.426501	Yes
**214393_at**	RND2	Rho family GTPase 2	1386	0.15989132225513458	0.427765	Yes
**204343_at**	ABCA3	ATP-binding cassette, sub-family A (ABC1), member 3	1388	0.15983474254608154	0.42985675	Yes
**221795_at**	NTRK2	neurotrophic tyrosine kinase, receptor, type 2	1390	0.1595579981803894	0.43194476	Yes
**201535_at**	UBL3	ubiquitin-like 3	1417	0.15807877480983734	0.43286234	Yes
**209582_s_at**	CD200	CD200 molecule	1419	0.15787556767463684	0.43492785	Yes
**205062_x_at**	ARID4A	AT rich interactive domain 4A (RBP1-like)	1458	0.15532812476158142	0.4352563	Yes
**209290_s_at**	NFIB	nuclear factor I/B	1469	0.15476998686790466	0.43686604	Yes
**209763_at**	CHRDL1	chordin-like 1	1473	0.15464192628860474	0.43879625	Yes
**212935_at**	MCF2L	MCF.2 cell line derived transforming sequence-like	1488	0.1538587510585785	0.44020972	Yes
**211466_at**	NFIB	nuclear factor I/B	1503	0.1529616266489029	0.44161117	Yes
**210823_s_at**	PTPRS	protein tyrosine phosphatase, receptor type, S	1504	0.15294408798217773	0.44365677	Yes
**202871_at**	TRAF4	TNF receptor-associated factor 4	1517	0.15239082276821136	0.44514263	Yes
**207693_at**	CACNB4	calcium channel, voltage-dependent, beta 4 subunit	1525	0.15203790366649628	0.44685394	Yes
**209469_at**	GPM6A	glycoprotein M6A	1566	0.1499587893486023	0.44701847	Yes
**212624_s_at**	CHN1	chimerin (chimaerin) 1	1571	0.14974671602249146	0.44883722	Yes
**211894_x_at**	SEZ6L	seizure related 6 homolog (mouse)-like /// seizure related 6 homolog (mouse)-like	1580	0.14908277988433838	0.45046294	Yes
**202946_s_at**	BTBD3	BTB (POZ) domain containing 3	1609	0.14737869799137115	0.45114532	Yes
**209816_at**	PTCH1	patched homolog 1 (Drosophila)	1612	0.14708881080150604	0.45302057	Yes
**214070_s_at**	ATP10B	ATPase, Class V, type 10B	1618	0.1464974582195282	0.45474982	Yes
**215115_x_at**	NTRK3	neurotrophic tyrosine kinase, receptor, type 3	1630	0.14575041830539703	0.45619288	Yes
**220977_x_at**	EPB41L5	erythrocyte membrane protein band 4.1 like 5 /// erythrocyte membrane protein band 4.1 like 5	1638	0.14538714289665222	0.45781523	Yes
**219250_s_at**	FLRT3	fibronectin leucine rich transmembrane protein 3	1641	0.14526422321796417	0.45966604	Yes
**210739_x_at**	SLC4A4	solute carrier family 4, sodium bicarbonate cotransporter, member 4	1652	0.14460158348083496	0.4611398	Yes
**210360_s_at**	MTSS1	metastasis suppressor 1	1671	0.14392578601837158	0.4622363	Yes
**207152_at**	NTRK2	neurotrophic tyrosine kinase, receptor, type 2	1673	0.14391720294952393	0.46411514	Yes
**220405_at**	SNTG1	syntrophin, gamma 1	1678	0.1437046080827713	0.46585304	Yes
**209289_at**	NFIB	nuclear factor I/B	1690	0.14325569570064545	0.46726274	Yes
**203527_s_at**	APC	adenomatosis polyposis coli	1707	0.142525777220726	0.46843258	Yes
**204029_at**	CELSR2	cadherin, EGF LAG seven-pass G-type receptor 2 (flamingo homolog, Drosophila)	1709	0.14238378405570984	0.4702909	Yes
**35147_at**	MCF2L	MCF.2 cell line derived transforming sequence-like	1712	0.1423191875219345	0.47210234	Yes

**217377_x_at**	NTRK3	neurotrophic tyrosine kinase, receptor, type 3	1723	0.14192919433116913	0.47354034	Yes
**210073_at**	ST8SIA1	ST8 alpha-N-acetyl-neuraminide alpha-2,8-sialyltransferase 1	1726	0.1418042927980423	0.4753449	Yes
**203525_s_at**	APC	adenomatosis polyposis coli	1731	0.1413954496383667	0.47705194	Yes
**209094_at**	DDAH1	dimethylarginine dimethylaminohydrolase 1	1745	0.14031238853931427	0.47833022	Yes
**201668_x_at**	MARCKS	myristoylated alanine-rich protein kinase C substrate	1750	0.14002220332622528	0.48001888	Yes
**203632_s_at**	GPRC5B	G protein-coupled receptor, family C, group 5, member B	1771	0.1392555832862854	0.48096085	Yes
**1438_at**	EPHB3	EPH receptor B3	1818	0.13688263297080994	0.48067436	Yes
**219726_at**	NLGN3	neuroligin 3	1867	0.13409411907196045	0.48025852	Yes
**209291_at**	ID4	inhibitor of DNA binding 4, dominant negative helix-loop-helix protein	1890	0.13313785195350647	0.4810266	Yes
**205893_at**	NLGN1	neuroligin 1	1914	0.1319299191236496	0.4817325	Yes
**222146_s_at**	TCF4	transcription factor 4	1963	0.12932275235652924	0.48125285	Yes
**216033_s_at**	FYN	FYN oncogene related to SRC, FGR, YES	2015	0.12774091958999634	0.48061395	Yes
**41644_at**	SASH1	SAM and SH3 domain containing 1	2049	0.12633919715881348	0.48078477	Yes
**205712_at**	PTPRD	protein tyrosine phosphatase, receptor type, D	2091	0.12416946142911911	0.4805584	Yes
**217033_x_at**	NTRK3	neurotrophic tyrosine kinase, receptor, type 3	2100	0.12378866970539093	0.48184583	Yes
**214168_s_at**	TJP1	tight junction protein 1 (zona occludens 1)	2158	0.1214049831032753	0.48084602	Yes
**211208_s_at**	CASK	calcium/calmodulin-dependent serine protein kinase (MAGUK family)	2186	0.12030167877674103	0.4812123	Yes
**221003_s_at**	CAB39L	calcium binding protein 39-like /// calcium binding protein 39-like	2220	0.11907308548688889	0.48128593	Yes
**203036_s_at**	MTSS1	metastasis suppressor 1	2228	0.1187651976943016	0.4825522	Yes
**204519_s_at**	PLLP	plasma membrane proteolipid (plasmolipin)	2235	0.11842784285545349	0.48386	Yes
**208070_s_at**	REV3L	REV3-like, catalytic subunit of DNA polymerase zeta (yeast)	2239	0.11828062683343887	0.4853039	Yes
**204966_at**	BAI2	brain-specific angiogenesis inhibitor 2	2243	0.11813624948263168	0.48674586	Yes
**203864_s_at**	ACTN2	actinin, alpha 2	2249	0.11790721118450165	0.48809272	Yes
**213002_at**	MARCKS	Myristoylated alanine-rich protein kinase C substrate	2337	0.11482402682304382	0.48562405	Yes
**214078_at**	PAK3	P21 (CDKN1A)-activated kinase 3	2347	0.11459324508905411	0.48674247	Yes
**202743_at**	PIK3R3	phosphoinositide-3-kinase, regulatory subunit 3 (p55, gamma)	2357	0.11445114016532898	0.48785898	Yes
**207112_s_at**	GAB1	GRB2-associated binding protein 1	2364	0.11403854191303253	0.48910806	Yes
**209470_s_at**	GPM6A	glycoprotein M6A	2398	0.11271186172962189	0.48909664	Yes
**208365_s_at**	GRK4	G protein-coupled receptor kinase 4	2415	0.11219466477632523	0.4898608	Yes
**215668_s_at**	PLXNB1	plexin B1	2419	0.11199069768190384	0.49122056	Yes
**220576_at**	PGAP1	GPI deacylase	2443	0.11089474707841873	0.49164513	Yes
**213467_at**	RND2	Rho family GTPase 2	2475	0.11010928452014923	0.49169096	Yes
**220316_at**	NPAS3	neuronal PAS domain protein 3	2477	0.11008314788341522	0.49311727	Yes

**213721_at**	SOX2	SRY (sex determining region Y)-box 2	2480	0.10999014228582382	0.49449632	Yes
**218528_s_at**	RNF38	ring finger protein 38	2517	0.10887925326824188	0.49429557	Yes
**209737_at**	MAGI2	membrane associated guanylate kinase, WW and PDZ domain containing 2	2563	0.10724000632762909	0.49365863	Yes
**214971_s_at**	ST6GAL1	ST6 beta-galactosamide alpha-2,6-sialyltranferase 1	2572	0.1069362610578537	0.49472067	Yes
**212479_s_at**	RMND5A	required for meiotic nuclear division 5 homolog A (S. cerevisiae)	2575	0.10685732960700989	0.4960578	Yes
**201670_s_at**	MARCKS	myristoylated alanine-rich protein kinase C substrate	2588	0.10615751892328262	0.49692532	Yes
**215611_at**	TCF12	transcription factor 12 (HTF4, helix-loop-helix transcription factors 4)	2722	0.1022481918334961	0.49217117	No
**210359_at**	MTSS1	metastasis suppressor 1	2728	0.10212309658527374	0.4933069	No
**202594_at**	LEPROTL 1	leptin receptor overlapping transcript-like 1	2735	0.1020055040717125	0.49439505	No
**202011_at**	TJP1	tight junction protein 1 (zona occludens 1)	2740	0.10196149349212646	0.49557465	No
**205230_at**	RPH3A	rabphilin 3A homolog (mouse)	2806	0.10024488717317581	0.4939236	No
**207055_at**	GPR37L1	G protein-coupled receptor 37 like 1	2850	0.09912077337503433	0.49327013	No
**206492_at**	FHIT	fragile histidine triad gene	2884	0.09840122610330582	0.49306732	No
**220454_s_at**	SEMA6A	sema domain, transmembrane domain (TM), and cytoplasmic domain, (semaphorin) 6A	2890	0.09823986887931824	0.49415112	No
**214543_x_at**	QKI	quaking homolog, KH domain RNA binding (mouse)	2904	0.09793934971094131	0.49486268	No
**214541_s_at**	QKI	quaking homolog, KH domain RNA binding (mouse)	2963	0.09676247835159302	0.49348727	No
**201235_s_at**	BTG2	BTG family, member 2	3071	0.09396553039550781	0.48981905	No
**213793_s_at**	HOMER1	homer homolog 1 (Drosophila)	3076	0.09388771653175354	0.49089068	No
**200795_at**	SPARCL1	SPARC-like 1 (mast9, hevin)	3125	0.09290486574172974	0.48992392	No
**203753_at**	TCF4	transcription factor 4	3217	0.09091014415025711	0.48695132	No
**215025_at**	NTRK3	neurotrophic tyrosine kinase, receptor, type 3	3237	0.09044092893600464	0.48728642	No
**209197_at**	SYT11	synaptotagmin XI	3257	0.0900738313794136	0.4876166	No
**210105_s_at**	FYN	FYN oncogene related to SRC, FGR, YES	3305	0.08891378343105316	0.4866425	No
**220529_at**	FLJ11710	hypothetical protein FLJ11710	3316	0.08878565579652786	0.48736972	No
**206053_at**	ZNF510	zinc finger protein 510	3318	0.08872948586940765	0.48851043	No
**205426_s_at**	HIP1	huntingtin interacting protein 1	3326	0.08857114613056183	0.48937288	No
**204484_at**	PIK3C2B	phosphoinositide-3-kinase, class 2, beta polypeptide	3330	0.08847223967313766	0.49041808	No
**209608_s_at**	ACAT2	acetyl-Coenzyme A acetyltransferase 2 (acetoacetyl Coenzyme A thiolase)	3384	0.08761182427406311	0.4891504	No
**220920_at**	ATP10B	ATPase, Class V, type 10B	3428	0.08686060458421707	0.48833296	No
**209590_at**	BMP7	Bone morphogenetic protein 7 (osteogenic protein 1)	3522	0.08508636802434921	0.4851904	No
**206140_at**	LHX2	LIM homeobox 2	3555	0.08438161015510559	0.4848461	No

**213722_at**	SOX2	SRY (sex determining region Y)-box 2	3557	0.08430681377649307	0.48592764	No
**210600_s_at**	GRK4	G protein-coupled receptor kinase 4	3703	0.08201771974563599	0.48035058	No
**209597_s_at**	PNMA2	paraneoplastic antigen MA2	3704	0.08201591670513153	0.48144755	No
**210100_s_at**	ABCA2	ATP-binding cassette, sub-family A (ABC1), member 2	3734	0.08145365118980408	0.4812022	No
**211219_s_at**	LHX2	LIM homeobox 2	3772	0.08065430074930191	0.4805779	No
**34697_at**	LRP6	low density lipoprotein receptor-related protein 6	3791	0.08029322326183319	0.48082328	No
**205606_at**	LRP6	low density lipoprotein receptor-related protein 6	3800	0.08022619783878326	0.48152807	No
**219255_x_at**	IL17RB	interleukin 17 receptor B	3845	0.07943178713321686	0.48056525	No
**219738_s_at**	PCDH9	protocadherin 9	3877	0.0788387805223465	0.48019284	No
**209598_at**	PNMA2	paraneoplastic antigen MA2	3899	0.07830220460891724	0.48027354	No
**211913_s_at**	MERTK	c-mer proto-oncogene tyrosine kinase /// c-mer proto-oncogene tyrosine kinase	3925	0.07773786038160324	0.48016256	No
**219737_s_at**	PCDH9	protocadherin 9	3930	0.07771836221218109	0.48101792	No
**203217_s_at**	ST3GAL5	ST3 beta-galactoside alpha-2,3-sialyltransferase 5	3969	0.07711406797170639	0.48030025	No
**216350_s_at**	ZNF10	zinc finger protein 10	4064	0.07551589608192444	0.47698367	No
**215310_at**	APC	Adenomatosis polyposis coli	4101	0.07501500099897385	0.47632998	No
**215962_at**	SNTG1	Syntrophin, gamma 1	4121	0.07471383363008499	0.47645473	No
**207613_s_at**	CAMK2A	calcium/calmodulin-dependent protein kinase (CaM kinase) II alpha	4149	0.07438287138938904	0.47620684	No
**214817_at**	UNC13A	unc-13 homolog A (C. elegans)	4151	0.07433709502220154	0.47715506	No
**208522_s_at**	PTCH1	patched homolog 1 (Drosophila)	4169	0.07409704476594925	0.47736362	No
**220619_at**	CHD7	chromodomain helicase DNA binding protein 7	4226	0.07292832434177399	0.47576147	No
**216113_at**	ABI2	Abl interactor 2	4228	0.07291719317436218	0.4766907	No
**205643_s_at**	PPP2R2B	protein phosphatase 2 (formerly 2A), regulatory subunit B (PR 52), beta isoform	4283	0.07224571704864502	0.47517148	No
**215396_at**	GPR98	G protein-coupled receptor 98	4287	0.0721842348575592	0.47599885	No
**204600_at**	EPHB3	EPH receptor B3	4325	0.07174788415431976	0.47525543	No
**218899_s_at**	BAALC	brain and acute leukemia, cytoplasmic	4444	0.07016818225383759	0.47076264	No
**208920_at**	SRI	sorcin	4469	0.06982914358377457	0.47059193	No
**208564_at**	KCNA2	potassium voltage-gated channel, shaker-related subfamily, member 2	4526	0.06893665343523026	0.46893638	No
**214829_at**	AASS	aminoadipate-semialdehyde synthase	4557	0.06831316649913788	0.46846923	No
**201998_at**	ST6GAL1	ST6 beta-galactosamide alpha-2,6-sialyltranferase 1	4585	0.06792532652616501	0.46813497	No
**212772_s_at**	ABCA2	ATP-binding cassette, sub-family A (ABC1), member 2	4617	0.0674629732966423	0.46761042	No
**211432_s_at**	TYRO3	TYRO3 protein tyrosine kinase	4645	0.06714442372322083	0.4672657	No
**212565_at**	STK38L	serine/threonine kinase 38 like	4671	0.06692934036254883	0.46701017	No
**216487_at**	ITPR2	Inositol 1,4,5-triphosphate receptor, type 2	4807	0.06510455161333084	0.46166718	No
**205475_at**	SCRG1	scrapie responsive protein 1	4833	0.0648324117064476	0.4613836	No
**202661_at**	ITPR2	inositol 1,4,5-triphosphate receptor, type 2	4861	0.06432726234197617	0.46100125	No

**206826_at**	PMP2	peripheral myelin protein 2	4913	0.06371486186981201	0.459506	No
**222153_at**	MYEF2	myelin expression factor 2	4922	0.0636066123843193	0.4599885	No
**202898_at**	SDC3	syndecan 3 (N-syndecan)	4933	0.06340663135051727	0.46037626	No
**209856_x_at**	ABI2	abl interactor 2	5019	0.06252259761095047	0.45730013	No
**214255_at**	ATP10A	ATPase, Class V, type 10A	5087	0.06160857155919075	0.45504028	No
**221615_at**	BMP8B	bone morphogenetic protein 8b (osteogenic protein 2)	5163	0.060924120247364044	0.45240304	No
**203562_at**	FEZ1	fasciculation and elongation protein zeta 1 (zygin I)	5201	0.06051987409591675	0.45150948	No
**207696_at**	FUT9	fucosyltransferase 9 (alpha (1,3) fucosyltransferase)	5217	0.0603003166615963	0.45162556	No
**214786_at**	MAP3K1	mitogen-activated protein kinase kinase kinase 1	5247	0.05998532846570015	0.45109305	No
**203220_s_at**	TLE1	transducin-like enhancer of split 1 (E(sp1) homolog, Drosophila)	5258	0.059860825538635254	0.4514334	No
**212636_at**	QKI	quaking homolog, KH domain RNA binding (mouse)	5281	0.05966051667928696	0.45121872	No
**212315_s_at**	NUP210	nucleoporin 210kDa	5292	0.059553176164627075	0.45155495	No
**213849_s_at**	PPP2R2B	protein phosphatase 2 (formerly 2A), regulatory subunit B (PR 52), beta isoform	5295	0.0595204159617424	0.45225897	No
**219950_s_at**	TIAM2	T-cell lymphoma invasion and metastasis 2	5344	0.05896853283047676	0.45083836	No
**215959_at**	PPFIBP2	PTPRF interacting protein, binding protein 2 (liprin beta 2)	5393	0.05839727073907852	0.44941005	No
**200953_s_at**	CCND2	cyclin D2	5437	0.05805324763059616	0.44820732	No
**212316_at**	NUP210	nucleoporin 210kDa	5488	0.057489022612571716	0.44667482	No
**203940_s_at**	VASH1	vasohibin 1	5525	0.05703815072774887	0.44578072	No
**206582_s_at**	GPR56	G protein-coupled receptor 56	5526	0.05702674388885498	0.44654343	No
**213469_at**	PGAP1	GPI deacylase	5546	0.05684385448694229	0.4464292	No
**212070_at**	GPR56	G protein-coupled receptor 56	5593	0.056430503726005554	0.44506666	No
**203861_s_at**	ACTN2	actinin, alpha 2	5642	0.055955223739147186	0.44360572	No
**222372_at**	MAGI1	Membrane associated guanylate kinase, WW and PDZ domain containing 1	5643	0.05593537539243698	0.44435385	No
**213945_s_at**	NUP210	Nucleoporin 210kDa	5688	0.05545813590288162	0.44307035	No
**220464_at**	MCF2L	MCF.2 cell line derived transforming sequence-like	5697	0.05535415932536125	0.4434425	No
**202524_s_at**	SPOCK2	sparc/osteonectin, cwcv and kazal-like domains proteoglycan (testican) 2	5699	0.0553179569542408	0.44413632	No
**36499_at**	CELSR2	cadherin, EGF LAG seven-pass G-type receptor 2 (flamingo homolog, Drosophila)	5722	0.05507583171129227	0.44386035	No
**201669_s_at**	MARCKS	myristoylated alanine-rich protein kinase C substrate	5851	0.05386100709438324	0.43868917	No
**206751_s_at**	PCYT1B	phosphate cytidylyltransferase 1, choline, beta	5908	0.05336172506213188	0.43682534	No
**217671_at**	RFX3	Regulatory factor X, 3 (influences HLA class II expression)	6006	0.05244576185941696	0.43306208	No
**203263_s_at**	ARHGEF 9	Cdc42 guanine nucleotide exchange factor (GEF) 9	6026	0.05228346586227417	0.43288684	No
**217569_x_at**	CAMK2D	Calcium/calmodulin-dependent protein kinase (CaM kinase) II delta	6106	0.05146346241235733	0.42993894	No

**216081_at**	LAMA4	laminin, alpha 4	6115	0.051393382251262665	0.4302581	No
**206028_s_at**	MERTK	c-mer proto-oncogene tyrosine kinase	6128	0.05129873380064964	0.43039188	No
**221586_s_at**	E2F5	E2F transcription factor 5, p130-binding	6135	0.05123533308506012	0.43080097	No
**216185_at**	FUT9	Fucosyltransferase 9 (alpha (1,3) fucosyltransferase)	6202	0.050705332309007645	0.42844132	No
**203549_s_at**	LPL	lipoprotein lipase	6204	0.05065936967730522	0.42907286	No
**206900_x_at**	ZNF253	zinc finger protein 253	6267	0.05003442242741585	0.42688835	No
**215479_at**	SEMA6A	Sema domain, transmembrane domain (TM), and cytoplasmic domain, (semaphorin) 6A	6302	0.04974331334233284	0.4259887	No
**216799_at**	CORO2B	Coronin, actin binding protein, 2B	6362	0.04919685795903206	0.42393106	No
**212406_s_at**	PCMTD2	protein-L-isoaspartate (D-aspartate) O-methyltransferase domain containing 2	6382	0.04903286695480347	0.42371234	No
**212263_at**	QKI	quaking homolog, KH domain RNA binding (mouse)	6393	0.04886188730597496	0.42390558	No
**201236_s_at**	BTG2	BTG family, member 2	6405	0.04873793199658394	0.42405114	No
**211912_at**	MERTK	c-mer proto-oncogene tyrosine kinase /// c-mer proto-oncogene tyrosine kinase	6424	0.04854901507496834	0.42387196	No
**215802_at**	TLE1	Transducin-like enhancer of split 1 (E(sp1) homolog, Drosophila)	6469	0.048217013478279114	0.42249164	No
**211431_s_at**	TYRO3	TYRO3 protein tyrosine kinase	6481	0.048102740198373795	0.4226287	No
**217502_at**	IFIT2	interferon-induced protein with tetratricopeptide repeats 2	6548	0.0474657341837883	0.4202257	No
**214256_at**	ATP10A	ATPase, Class V, type 10A	6652	0.0465775765478611	0.41610783	No
**222101_s_at**	DCHS1	dachsous 1 (Drosophila)	6876	0.0447600893676281	0.40644228	No
**220685_at**	FAM120C	family with sequence similarity 120C	6908	0.04450911656022072	0.4056107	No
**203850_s_at**	KIF1A	kinesin family member 1A	6967	0.043913304805755615	0.40352845	No
**212478_at**	RMND5A	required for meiotic nuclear division 5 homolog A (S. cerevisiae)	7132	0.042547859251499176	0.39654896	No
**221413_at**	KCNAB3	potassium voltage-gated channel, shaker-related subfamily, beta member 3	7181	0.042040467262268066	0.3949019	No
**211793_s_at**	ABI2	abl interactor 2	7272	0.04138776659965515	0.39131296	No
**211568_at**	BAI3	brain-specific angiogenesis inhibitor 3	7381	0.040551621466875076	0.38688433	No
**215964_at**	SNTG1	Syntrophin, gamma 1	7382	0.04055127874016762	0.3874267	No
**217265_at**	PLLP	plasma membrane proteolipid (plasmolipin)	7399	0.040441934019327164	0.38723114	No
**209591_s_at**	BMP7	bone morphogenetic protein 7 (osteogenic protein 1)	7436	0.04018009081482887	0.38611156	No
**211580_s_at**	PIK3R3	phosphoinositide-3-kinase, regulatory subunit 3 (p55, gamma)	7505	0.03974337875843048	0.3835132	No
**204835_at**	POLA1	polymerase (DNA directed), alpha 1	7560	0.03934360295534134	0.38155395	No
**219779_at**	ZFHX4	zinc finger homeodomain 4	7655	0.038621097803115845	0.37774387	No
**220847_x_at**	ZNF221	zinc finger protein 221	7867	0.03697466850280762	0.36852655	No
**210989_at**	LAMA4	Laminin, alpha 4	8158	0.03470055013895035	0.3556426	No

**202834_at**	AGT	angiotensinogen (serpin peptidase inhibitor, clade A, member 8)	8246	0.03411288186907768	0.3520944	No
**214178_s_at**	SOX2	SRY (sex determining region Y)-box 2	8295	0.033807940781116486	0.35033727	No
**211164_at**	EPHA3	EPH receptor A3	8436	0.03282728046178818	0.34433243	No
**206202_at**	MEOX2	mesenchyme homeobox 2	8595	0.03177214786410332	0.337485	No
**204906_at**	RPS6KA2	ribosomal protein S6 kinase, 90kDa, polypeptide 2	8629	0.031546883285045624	0.336388	No
**216802_at**	CORO2B	Coronin, actin binding protein, 2B	8646	0.03145043924450874	0.3360722	No
**207401_at**	PROX1	prospero-related homeobox 1	8930	0.029513319954276085	0.32344106	No
**220035_at**	NUP210	nucleoporin 210kDa	8971	0.029230043292045593	0.3219909	No
**210099_at**	ABCA2	ATP-binding cassette, sub-family A (ABC1), member 2	9037	0.0288253091275692	0.31938463	No
**202523_s_at**	SPOCK2	sparc/osteonectin, cwcv and kazal-like domains proteoglycan (testican) 2	9043	0.028789151459932327	0.31953955	No
**217697_at**	FYN	FYN oncogene related to SRC, FGR, YES	9105	0.028290431946516037	0.31711024	No
**40837_at**	TLE2	transducin-like enhancer of split 2 (E(sp1) homolog, Drosophila)	9180	0.027798522263765335	0.31407598	No
**203862_s_at**	ACTN2	actinin, alpha 2	9324	0.026713835075497627	0.30785128	No
**212912_at**	RPS6KA2	ribosomal protein S6 kinase, 90kDa, polypeptide 2	9329	0.026698358356952667	0.30802426	No
**215372_x_at**	MAGI1	Membrane associated guanylate kinase, WW and PDZ domain containing 1	9352	0.02655867673456669	0.30736688	No
**207702_s_at**	MAGI2	membrane associated guanylate kinase, WW and PDZ domain containing 2	9550	0.025252047926187515	0.29863712	No
**220829_s_at**	B3GALT1	UDP-Gal:betaGlcNAc beta 1,3-galactosyltransferase, polypeptide 1	9691	0.02442343533039093	0.2925199	No
**201790_s_at**	DHCR7	7-dehydrocholesterol reductase	9723	0.024176480248570442	0.2914164	No
**214227_at**	GNG7	Guanine nucleotide binding protein (G protein), gamma 7	9736	0.024105172604322433	0.29118648	No
**216488_s_at**	ATP11A	ATPase, Class VI, type 11A	9742	0.02408890798687935	0.2912785	No
**202191_s_at**	GAS7	growth arrest-specific 7	10067	0.021688371896743774	0.27665558	No
**211260_at**	BMP7	bone morphogenetic protein 7 (osteogenic protein 1)	10120	0.0213078074157238	0.27454713	No
**209389_x_at**	DBI	diazepam binding inhibitor (GABA receptor modulator, acyl-Coenzyme A binding protein)	10159	0.021035052835941315	0.27307943	No
**208491_s_at**	PGM5	phosphoglucomutase 5	10191	0.02082817070186138	0.2719311	No
**202192_s_at**	GAS7	growth arrest-specific 7	10217	0.020719347521662712	0.27105755	No
**216134_at**	FRMD4B	FERM domain containing 4B	10254	0.020489871501922607	0.2696746	No
**207268_x_at**	ABI2	abl interactor 2	10286	0.020247986540198326	0.26851854	No
**215671_at**	PDE4B	phosphodiesterase 4B, cAMP-specific (phosphodiesterase E4 dunce homolog, Drosophila)	10527	0.018698088824748993	0.25772196	No
**211070_x_at**	DBI	enzyme A binding protein)	10550	0.018555626273155212	0.25695753	No
**210738_s_at**	SLC4A4	solute carrier family 4, sodium bicarbonate cotransporter, member 4	10560	0.018470721319317818	0.2567903	No
**211077_s_at**	TLK1	tousled-like kinase 1 /// tousled-like kinase 1	10599	0.018198544159531593	0.25528467	No

**221047_s_at**	MARK1	MAP/microtubule affinity-regulating kinase 1	10658	0.017806151881814003	0.2528532	No
**204761_at**	USP6NL	USP6 N-terminal like	10796	0.01692674309015274	0.2467738	No
**215428_at**	TJP1	Tight junction protein 1 (zona occludens 1)	10892	0.016282256692647934	0.24261892	No
**216614_at**	ITPR2	Inositol 1,4,5-triphosphate receptor, type 2	10927	0.01603442057967186	0.24126843	No
**208408_at**	PTN	pleiotrophin (heparin binding growth factor 8, neurite growth-promoting factor 1)	11393	0.013187281787395477	0.22004189	No
**205973_at**	FEZ1	fasciculation and elongation protein zeta 1 (zygin I)	11616	0.011515152640640736	0.20997772	No
**212841_s_at**	PPFIBP2	PTPRF interacting protein, binding protein 2 (liprin beta 2)	11619	0.011484823189675808	0.21003927	No
**216707_at**	PCDH9	Protocadherin 9	11656	0.011224310845136642	0.2085324	No
**207865_s_at**	BMP8B	bone morphogenetic protein 8b (osteogenic protein 2)	11767	0.010413113981485367	0.20360862	No
**212265_at**	QKI	quaking homolog, KH domain RNA binding (mouse)	11880	0.009785131551325321	0.19858438	No
**204311_at**	ATP1B2	ATPase, Na+/K+ transporting, beta 2 polypeptide	11994	0.009023511782288551	0.19350392	No
**205738_s_at**	FABP3	fatty acid binding protein 3, muscle and heart (mammary-derived growth inhibitor)	12009	0.008916682563722134	0.19297878	No
**212572_at**	STK38L	serine/threonine kinase 38 like	12037	0.00871847104281187	0.19185264	No
**211827_s_at**	KCND3	potassium voltage-gated channel, Shal-related subfamily, member 3	12045	0.008682196959853172	0.19164658	No
**204453_at**	ZNF84	zinc finger protein 84	12121	0.008187493309378624	0.18830399	No
**207454_at**	GRIK3	glutamate receptor, ionotropic, kainate 3	12185	0.007747315336018801	0.18550786	No
**209789_at**	CORO2B	coronin, actin binding protein, 2B	12295	0.006998586002737284	0.18058443	No
**205112_at**	PLCE1	phospholipase C, epsilon 1	12386	0.006300784181803465	0.1765262	No
**215842_s_at**	ATP11A	ATPase, Class VI, type 11A	12390	0.0062783220782876015	0.1764721	No
**204141_at**	TUBB2A	tubulin, beta 2A	12391	0.006275716703385115	0.17655604	No
**203315_at**	NCK2	NCK adaptor protein 2	12423	0.006101028528064489	0.17521077	No
**204469_at**	PTPRZ1	protein tyrosine phosphatase, receptor-type, Z polypeptide 1	12424	0.006091001443564892	0.17529224	No
**217202_s_at**	GLUL	glutamate-ammonia ligase (glutamine synthetase)	12677	0.004219221416860819	0.16374967	No
**211301_at**	KCND3	potassium voltage-gated channel, Shal-related subfamily, member 3	12700	0.004086614120751619	0.16279171	No
**203185_at**	RASSF2	Ras association (RalGDS/AF-6) domain family 2	12721	0.003979467321187258	0.16192438	No
**214159_at**	PLCE1	Phospholipase C, epsilon 1	12899	0.0026613855734467506	0.15381305	No
**217516_x_at**	ARVCF	armadillo repeat gene deletes in velocardiofacial syndrome	12988	0.002025523455813527	0.14978969	No
**204431_at**	TLE2	transducin-like enhancer of split 2 (E(sp1) homolog, Drosophila)	13040	0.0016287903999909759	0.14746407	No
**202428_x_at**	DBI	diazepam binding inhibitor (GABA receptor modulator, acyl-Coenzyme A binding protein)	13073	0.0013585996348410845	0.14600934	No
**211360_s_at**	ITPR2	inositol 1,4,5-triphosphate receptor, type 2	13130	950886926148,1	0.14344451	No

**215322_at**	LONRF1	LON peptidase N-terminal domain and ring finger 1	13166	737854395993,1	0.1418434	No
**201534_s_at**	UBL3	ubiquitin-like 3	13168	729879073333,0	0.14180714	No
**213050_at**	COBL	cordon-bleu homolog (mouse)	13272	-1153113771579,2	0.13706781	No
**219686_at**	STK32B	serine/threonine kinase 32B	13308	-364479084964,8	0.13546172	No
**210990_s_at**	LAMA4	laminin, alpha 4	13402	-0.0010860445909202099	0.13119565	No
**205111_s_at**	PLCE1	phospholipase C, epsilon 1	13409	-0.00119075062684 7148	0.13093542	No
**219693_at**	AGPAT4	1-acylglycerol-3-phosphate O-acyltransferase 4 (lysophosphatidic acid acyltransferase, delta)	13431	-0.0013542647939175367	0.12998694	No
**218892_at**	DCHS1	dachsous 1 (Drosophila)	13535	-0.0022830679081380367	0.12527661	No
**210830_s_at**	PON2	paraoxonase 2	13694	-0.0034849662333726883	0.118050836	No
**216131_at**	FRMD4B	FERM domain containing 4B	13708	-0.0035891933366656303	0.11750048	No
**207234_at**	RFX3	regulatory factor X, 3 (influences HLA class II expression)	13756	-0.004068463575094938	0.11539158	No
**201194_at**	SEPW1	selenoprotein W, 1	13852	-0.004904320929199457	0.111084536	No
**200952_s_at**	CCND2	cyclin D2	13893	-0.005172996316105127	0.10931262	No
**210872_x_at**	GAS7	growth arrest-specific 7	13916	-0.005442379973828793	0.10837279	No
**203111_s_at**	PTK2B	PTK2B protein tyrosine kinase 2 beta	13949	-0.005807454232126474	0.10697758	No
**200951_s_at**	CCND2	cyclin D2	13974	-0.00595773896202445	0.1059526	No
**220354_at**	MCF2L	MCF.2 cell line derived transforming sequence-like	14144	-0.007303472142666578	0.09827158	No
**204836_at**	GLDC	glycine dehydrogenase (decarboxylating)	14223	-0.008015912026166916	0.09478862	No
**205784_x_at**	ARVCF	armadillo repeat gene deletes in velocardiofacial syndrome	14255	-0.008247883059084415	0.09347207	No
**220287_at**	ADAMTS 9	ADAM metallopeptidase with thrombospondin type 1 motif, 9	14256	-0.008248437196016312	0.0935824	No
**213582_at**	ATP11A	ATPase, Class VI, type 11A	14387	-0.00941505841910839	0.08772471	No
**202254_at**	SIPA1L1	Signal-induced proliferation-associated 1 like 1	14388	-0.00946372002363205	0.08785128	No
**201876_at**	PON2	paraoxonase 2	14489	-0.010424576699733734	0.083387926	No
**211259_s_at**	BMP7	bone morphogenetic protein 7 (osteogenic protein 1)	14536	-0.010865644551813602	0.08141597	No
**211302_s_at**	PDE4B	phosphodiesterase 4B, cAMP-specific (phosphodiesterase E4 dunce homolog, Drosophila)	14577	-0.011201372370123863	0.079724684	No
**215638_at**	ERBB3	v-erb-b2 erythroblastic leukemia viral oncogene homolog 3 (avian)	14695	-0.012305566109716892	0.07450401	No
**219535_at**	HUNK	hormonally upregulated Neu-associated kinase	14729	-0.012644654139876366	0.07315422	No
**219699_at**	LGI2	leucine-rich repeat LGI family, member 2	14768	-0.01299999374896288	0.07157903	No
**213947_s_at**	NUP210	nucleoporin 210kDa	14817	-0.013429706916213036	0.069549315	No
**210456_at**	PCYT1B	phosphate cytidylyltransferase 1, choline, beta	14827	-0.013552075251936913	0.06931633	No
**222172_at**	NPAS3	neuronal PAS domain protein 3	14828	-0.013556758873164654	0.069497645	No
**220226_at**	TRPM8	transient receptor potential cation channel, subfamily M, member 8	14992	-0.01499187108129263	0.062195625	No
**209841_s_at**	LRRN3	leucine rich repeat neuronal 3	15054	-0.015623630955815315	0.059596892	No
**205425_at**	HIP1	huntingtin interacting protein 1	15142	-0.016579143702983856	0.055814218	No
**209840_s_at**	LRRN3	leucine rich repeat neuronal 3	15389	-0.01941380277276039	0.044751037	No

**203222_s_at**	TLE1	transducin-like enhancer of split 1 (E(sp1) homolog, Drosophila)	15486	-0.020446887239813805	0.04060584	No
**211222_s_at**	HAP1	huntingtin-associated protein 1 (neuroan 1)	15494	-0.020559383556246758	0.040558625	No
**214023_x_at**	TUBB2B	tubulin, beta 2B	15636	-0.0223710797727108	0.03436791	No
**203221_at**	TLE1	transducin-like enhancer of split 1 (E(sp1) homolog, Drosophila)	15664	-0.02269693650305271	0.033428732	No
**209686_at**	S100B	S100 calcium binding protein, beta (neural)	15711	-0.02331898733973503	0.031623337	No
**205363_at**	BBOX1	butyrobetaine (gamma), 2-oxoglutarate dioxygenase (gamma-butyrobetaine hydroxylase) 1	15718	-0.023401379585266113	0.031660162	No
**202662_s_at**	ITPR2	inositol 1,4,5-triphosphate receptor, type 2	15825	-0.02474510669708252	0.027112177	No
**205590_at**	RASGRP1	RAS guanyl releasing protein 1 (calcium and DAG-regulated)	15846	-0.02496710605919361	0.026525551	No
**203548_s_at**	LPL	lipoprotein lipase	15963	-0.026519445702433586	0.02154102	No
**208921_s_at**	SRI	sorcin	16017	-0.02723103202879429	0.019465758	No
**214538_x_at**	RGS6	regulator of G-protein signalling 6	16054	-0.02772645093500614	0.018179594	No
**205928_at**	ZNF443	zinc finger protein 443	16067	-0.02788396365940571	0.018000204	No
**219247_s_at**	ZDHHC14	zinc finger, DHHC-type containing 14	16075	-0.02795548550784588	0.01805191	No
**204639_at**	ADA	adenosine deaminase	16096	-0.028294453397393227	0.017509786	No
**212262_at**	QKI	quaking homolog, KH domain RNA binding (mouse)	16123	-0.028757309541106224	0.016697688	No
**221933_at**	NLGN4X	neuroligin 4, X-linked	16149	-0.029051685705780983	0.015935555	No
**222326_at**	PDE4B	Phosphodiesterase 4B, cAMP-specific (phosphodiesterase E4 dunce homolog, Drosophila)	16216	-0.0298608969897031 8	0.013297104	No
**214970_s_at**	ST6GAL1	ST6 beta-galactosamide alpha-2,6-sialyltranferase 1	16258	-0.030453819781541824	0.011817279	No
**219634_at**	CHST11	carbohydrate (chondroitin 4) sulfotransferase 11	16325	-0.03125624731183052	0.009197492	No
**207583_at**	ABCD2	ATP-binding cassette, sub-family D (ALD), member 2	16340	-0.03142266720533371	0.008973376	No
**201847_at**	LIPA	lipase A, lysosomal acid, cholesterol esterase (Wolman disease)	16500	-0.033457837998867035	0.0021024484	No
**220299_at**	SPATA6	spermatogenesis associated 6	16528	-0.033907677978277206	0.0013132073	No
**209695_at**	PTP4A3	protein tyrosine phosphatase type IVA, member 3	16539	-0.03401905298233032	0.0013079287	No
**201791_s_at**	DHCR7	7-dehydrocholesterol reductase	16709	-0.03640759736299515	-0.005983824	No
**203708_at**	PDE4B	phosphodiesterase 4B, cAMP-specific (phosphodiesterase E4 dunce homolog, Drosophila)	16812	-0.03784959018230438	-0.010172428	No
**210379_s_at**	TLK1	tousled-like kinase 1	16817	-0.03788388520479202	-0.009849848	No
**220983_s_at**	SPRY4	sprouty homolog 4 (Drosophila) /// sprouty homolog 4 (Drosophila)	16975	-0.04025355726480484	-0.016537828	No
**213435_at**	SATB2	SATB family member 2	16994	-0.04047943279147148	-0.016824923	No
**202595_s_at**	LEPROTL1	leptin receptor overlapping transcript-like 1	17013	-0.04084492102265358	-0.01710713	No

**204069_at**	MEIS1	Meis1, myeloid ecotropic viral integration site 1 homolog (mouse)	17089	-0.042249780148267746	-0.01999413	No
**215591_at**	SATB2	SATB family member 2	17101	-0.042416565120220184	-0.019933121	No
**209202_s_at**	EXTL3	exostoses (multiple)-like 3	17131	-0.042898453772068024	-0.020694168	No
**207704_s_at**	GAS7	growth arrest-specific 7	17150	-0.04325054958462715	-0.020944199	No
**215001_s_at**	GLUL	glutamate-ammonia ligase (glutamine synthetase)	17251	-0.04479121044278145	-0.024947904	No
**211603_s_at**	ETV4	ets variant gene 4 (E1A enhancer binding protein, E1AF) /// ets variant gene 4 (E1A enhancer binding protein, E1AF)	17302	-0.045655425637960434	-0.02663866	No
**213395_at**	MLC1	megalencephalic leukoencephalopathy with subcortical cysts 1	17427	-0.0479620099067688	-0.031704623	No
**204741_at**	BICD1	bicaudal D homolog 1 (Drosophila)	17475	-0.0488295778632164	-0.03321484	No
**211604_x_at**	HAP1	huntingtin-associated protein 1 (neuroan 1) /// huntingtin-associated protein 1 (neuroan 1)	17493	-0.0492224358022213	-0.03333897	No
**203110_at**	PTK2B	PTK2B protein tyrosine kinase 2 beta	17554	-0.05052013322710991	-0.03542494	No
**204391_x_at**	TRIM24	tripartite motif-containing 24	17614	-0.05157945677638054	-0.037450716	No
**211051_s_at**	EXTL3	exostoses (multiple)-like 3 /// exostoses (multiple)-like 3	17692	-0.05311805382370949	-0.04028441	No
**216705_s_at**	ADA	adenosine deaminase	17701	-0.053226109594106674	-0.03994074	No
**202479_s_at**	TRIB2	tribbles homolog 2 (Drosophila)	17760	-0.054241959005594254	-0.041884877	No
**210852_s_at**	AASS	aminoadipate-semialdehyde synthase	17870	-0.056566569954156876	-0.04614534	No
**218929_at**	CARF	collaborates/cooperates with ARF (alternate reading frame) protein	17893	-0.05712258815765381	-0.046393946	No
**218806_s_at**	VAV3	vav 3 oncogene	18022	-0.05956149846315384	-0.05148888	No
**200648_s_at**	GLUL	glutamate-ammonia ligase (glutamine synthetase)	18064	-0.060373105108737946	-0.052568536	No
**214285_at**	FABP3	fatty acid binding protein 3, muscle and heart (mammary-derived growth inhibitor)	18080	-0.06072692945599556	-0.05244674	No
**220298_s_at**	SPATA6	spermatogenesis associated 6	18129	-0.06182490661740303	-0.05382918	No
**206893_at**	SALL1	sal-like 1 (Drosophila)	18306	-0.06607918441295624	-0.061046273	No
**210170_at**	PDLIM3	PDZ and LIM domain 3	18376	-0.06779727339744568	-0.063315414	No
**219213_at**	JAM2	junctional adhesion molecule 2	18401	-0.06840967386960983	-0.06350511	No
**211067_s_at**	GAS7	growth arrest-specific 7 /// growth arrest-specific 7	18429	-0.06924574822187424	-0.06382171	No
**206333_at**	MSI1	musashi homolog 1 (Drosophila)	18461	-0.07035689055919647	-0.06430756	No
**210270_at**	RGS6	regulator of G-protein signalling 6	18498	-0.07109648734331131	-0.06501366	No
**216375_s_at**	ETV5	ets variant gene 5 (ets-related molecule)	18519	-0.0716598704457283	-0.064975776	No
**201726_at**	ELAVL1	ELAV (embryonic lethal, abnormal vision, Drosophila)-like 1 (Hu antigen R)	18597	-0.07356361299753189	-0.06753601	No
**202478_at**	TRIB2	tribbles homolog 2 (Drosophila)	18655	-0.07531750202178955	-0.06915224	No
**211448_s_at**	RGS6	regulator of G-protein signalling 6	18766	-0.0785321444272995	-0.07316494	No
**204776_at**	THBS4	thrombospondin 4	18775	-0.07885324954986572	-0.07247852	No

**212797_at**	SORT1	sortilin 1	18806	-0.07979696244001389	-0.072792076	No
**216733_s_at**	GATM	glycine amidinotransferase (L-arginine:glycine amidinotransferase)	18909	-0.08268314599990845	-0.07638104	No
**209205_s_at**	LMO4	LIM domain only 4	18916	-0.0828782245516777	-0.07554872	No
**202421_at**	IGSF3	immunoglobulin superfamily, member 3	18964	-0.08396394550800323	-0.076589026	No
**209204_at**	LMO4	LIM domain only 4	18973	-0.08432415127754211	-0.075829424	No
**209198_s_at**	SYT11	synaptotagmin XI	19019	-0.08577919751405716	-0.07675339	No
**206896_s_at**	GNG7	guanine nucleotide binding protein (G protein), gamma 7	19041	-0.08636459708213806	-0.07656486	No
**204724_s_at**	COL9A3	collagen, type IX, alpha 3	19054	-0.08670848608016968	-0.075957485	No
**206136_at**	FZD5	frizzled homolog 5 (Drosophila)	19085	-0.08758776634931564	-0.07616685	No
**209466_x_at**	PTN	pleiotrophin (heparin binding growth factor 8, neurite growth-promoting factor 1)	19117	-0.08817065507173538	-0.07641444	No
**218678_at**	NES	nestin	19121	-0.08823966234922409	-0.07537234	No
**219797_at**	MGAT4A	mannosyl (alpha-1,3-)-glycoprotein beta-1,4-N-acetylglucosaminyltransferase , isozyme A	19134	-0.08862249553203583	-0.07473936	No
**213301_x_at**	TRIM24	tripartite motif-containing 24	19187	-0.09014267474412918	-0.07592716	No
**210089_s_at**	LAMA4	laminin, alpha 4	19258	-0.09220071136951447	-0.07791594	No
**210106_at**	RDH5	retinol dehydrogenase 5 (11-cis/9-cis)	19348	-0.09516537934541702	-0.08073959	No
**201727_s_at**	ELAVL1	ELAV (embryonic lethal, abnormal vision, Drosophila)-like 1 (Hu antigen R)	19369	-0.09619534015655518	-0.08037355	No
**211737_x_at**	PTN	omoting factor 1)	19374	-0.09634647518396378	-0.07926904	No
**205249_at**	EGR2	early growth response 2 (Krox-20 homolog, Drosophila)	19391	-0.09694518148899078	-0.07870886	No
**202606_s_at**	TLK1	tousled-like kinase 1	19487	-0.10138200968503952	-0.08172553	No
**206574_s_at**	PTP4A3	protein tyrosine phosphatase type IVA, member 3	19656	-0.10809318721294403	-0.08801247	No
**220029_at**	ELOVL2	elongation of very long chain fatty acids (FEN1/Elo2, SUR4/Elo3, yeast)-like 2	19749	-0.11160242557525635	-0.09075437	No
**201193_at**	IDH1	isocitrate dehydrogenase 1 (NADP+), soluble	19798	-0.11337675899267197	-0.0914473	No
**204201_s_at**	PTPN13	protein tyrosine phosphatase, non-receptor type 13 (APO-1/CD95 (Fas)-associated phosphatase)	19905	-0.11784041672945023	-0.09475016	No
**202660_at**	ITPR2	Inositol 1,4,5-triphosphate receptor, type 2	20086	-0.12605266273021698	-0.10134923	No
**209474_s_at**	ENTPD1	ectonucleoside triphosphate diphosphohydrolase 1	20102	-0.1267687976360321	-0.10034413	No
**206071_s_at**	EPHA3	EPH receptor A3	20108	-0.12693871557712555	-0.098876484	No
**207691_x_at**	ENTPD1	ectonucleoside triphosphate diphosphohydrolase 1	20115	-0.12747140228748322	-0.09744774	No
**201889_at**	FAM3C	family with sequence similarity 3, member C	20175	-0.1300961673259735	-0.09842337	No
**203349_s_at**	ETV5	ets variant gene 5 (ets-related molecule)	20178	-0.1304338276386261	-0.09677089	No
**219683_at**	FZD3	frizzled homolog 3 (Drosophila)	20237	-0.13350097835063934	-0.097654946	No
**53991_at**	DENND2A	DENN/MADD domain containing 2A	20254	-0.13418114185333252	-0.09659674	No
**221886_at**	DENND2A	DENN/MADD domain containing 2A	20337	-0.13823507726192474	-0.09852215	No

**212807_s_at**	SORT1	sortilin 1	20348	-0.13893693685531616	-0.09712417	No
**220291_at**	GDPD2	glycerophosphodiester phosphodiesterase domain containing 2	20381	-0.14072678983211517	-0.09671486	No
**210233_at**	IL1RAP	interleukin 1 receptor accessory protein	20406	-0.14178511500358582	-0.09592317	No
**203178_at**	GATM	glycine amidinotransferase (L-arginine:glycine amidinotransferase)	20435	-0.14344793558120728	-0.09529336	No
**217757_at**	A2M	alpha-2-macroglobulin	20469	-0.1459314376115799	-0.094860464	No
**218807_at**	VAV3	vav 3 oncogene	20502	-0.14750094711780548	-0.09436055	No
**221885_at**	DENND2 A	DENN/MADD domain containing 2A	20554	-0.150554358959198	-0.09469433	No
**202255_s_at**	SIPA1L1	signal-induced proliferation-associated 1 like 1	20606	-0.15369148552417755	-0.09498615	No
**214390_s_at**	BCAT1	branched chain aminotransferase 1, cytosolic	20750	-0.16539236903190613	-0.09935603	No
**209465_x_at**	PTN	pleiotrophin (heparin binding growth factor 8, neurite growth-promoting factor 1)	20791	-0.16926904022693634	-0.0989332	No
**205186_at**	DNALI1	dynein, axonemal, light intermediate polypeptide 1	20797	-0.16966691613197327	-0.09689407	No
**214844_s_at**	DOK5	docking protein 5	20855	-0.17401714622974396	-0.0971902	No
**216967_at**	GAP43	growth associated protein 43	20954	-0.18246300518512726	-0.09926051	No
**203348_s_at**	ETV5	ets variant gene 5 (ets-related molecule)	21019	-0.18912748992443085	-0.09967674	No
**209674_at**	CRY1	cryptochrome 1 (photolyase-like)	21022	-0.18943123519420624	-0.09723519	No
**203917_at**	CXADR	coxsackie virus and adenovirus receptor	21025	-0.1894795447587967	-0.094792984	No
**215248_at**	GRB10	growth factor receptor-bound protein 10	21031	-0.19003556668758392	-0.09248143	No
**213056_at**	FRMD4B	FERM domain containing 4B	21035	-0.1908007115125656	-0.09006758	No
**207144_s_at**	CITED1	Cbp/p300-interacting transactivator, with Glu/Asp-rich carboxy-terminal domain, 1	21276	-0.22063599526882172	-0.098163284	No
**216331_at**	ITGA7	integrin, alpha 7	21318	-0.22741307318210602	-0.09700881	No
**206306_at**	RYR3	ryanodine receptor 3	21335	-0.2305852770805359	-0.09466121	No
**209410_s_at**	GRB10	growth factor receptor-bound protein 10	21351	-0.23378048837184906	-0.09222485	No
**218966_at**	MYO5C	myosin VC	21446	-0.24898718297481537	-0.0932213	No
**212558_at**	SPRY1	sprouty homolog 1, antagonist of FGF signaling (Drosophila)	21448	-0.2492828518152237	-0.08993321	No
**214180_at**	MAN1C1	mannosidase, alpha, class 1C, member 1	21500	-0.25773727893829346	-0.08883344	No
**219331_s_at**	KLHDC8 A	kelch domain containing 8A	21554	-0.26660335063934326	-0.08770713	No
**205227_at**	IL1RAP	interleukin 1 receptor accessory protein	21572	-0.27019360661506653	-0.08487581	No
**202202_s_at**	LAMA4	laminin, alpha 4	21612	-0.2810133993625641	-0.082912385	No
**219011_at**	PLEKHA 4	pleckstrin homology domain containing, family A (phosphoinositide binding specific) member 4	21614	-0.2813994288444519	-0.07919474	No
**209663_s_at**	ITGA7	integrin, alpha 7	21692	-0.29841142892837524	-0.078747675	No
**216963_s_at**	GAP43	growth associated protein 43	21699	-0.29965510964393616	-0.075016	No
**204471_at**	GAP43	growth associated protein 43	21728	-0.3077133595943451	-0.07218916	No
**206070_s_at**	EPHA3	EPH receptor A3	21749	-0.3153459131717682	-0.06889202	No

**213712_at**	ELOVL2	elongation of very long chain fatty acids (FEN1/Elo2, SUR4/Elo3, yeast)-like 2	21760	-0.31955718994140625	-0.06507827	No
**219099_at**	C12ORF5	chromosome 12 open reading frame 5	21770	-0.32155483961105347	-0.061191775	No
**209409_at**	GRB10	growth factor receptor-bound protein 10	21783	-0.3254410922527313	-0.057391386	No
**204041_at**	MAOB	monoamine oxidase B	21833	-0.342296838760376	-0.055068586	No
**222217_s_at**	SLC27A3	solute carrier family 27 (fatty acid transporter), member 3	21850	-0.3473711907863617	-0.051158994	No
**209621_s_at**	PDLIM3	PDZ and LIM domain 3	21879	-0.355916291475296	-0.04768745	No
**214913_at**	ADAMTS 3	ADAM metallopeptidase with thrombospondin type 1 motif, 3	21914	-0.36738651990890503	-0.04433866	No
**201896_s_at**	PSRC1	proline/serine-rich coiled-coil 1	21934	-0.3756332993507385	-0.040189154	No
**210999_s_at**	GRB10	growth factor receptor-bound protein 10	21958	-0.387459933757782	-0.03606558	No
**220543_at**	C21ORF6 2	chromosome 21 open reading frame 62	21991	-0.4067578911781311	-0.032098144	No
**203561_at**	FCGR2A	Fc fragment of IgG, low affinity IIa, receptor (CD32)	22022	-0.4204965829849243	-0.027854905	No
**214452_at**	BCAT1	branched chain aminotransferase 1, cytosolic	22035	-0.4276231527328491	-0.022687847	No
**201792_at**	AEBP1	AE binding protein 1	22044	-0.4351990222930908	-0.017235354	No
**218918_at**	MAN1C1	mannosidase, alpha, class 1C, member 1	22051	-0.4419667720794678	-0.011600285	No
**204591_at**	CHL1	cell adhesion molecule with homology to L1CAM (close homolog of L1)	22133	-0.4938931167125702	-0.008722795	No
**206201_s_at**	MEOX2	mesenchyme homeobox 2	22134	-0.4949165880680084	-0.002103365	No
**202718_at**	IGFBP2	insulin-like growth factor binding protein 2, 36kDa	22230	-0.6631748676300049	0.0023938513	No

Supplementary List 2 - List of the 424 top ranking genes upregulated in GCLs *vs*. CSCs and selected based on an Adjusted P value < 0.01 e log2 Fold Change > 1 for Gene Set Enrichment Analysis (GSEA) in the comparison PN *vs*. MES.

PROBE	GENE SYMBOL	GENE_TITLE	RANK IN GENE LIST	RANK METRIC SCORE	RUNNING ES	CORE ENRICHMENT
**215446_s_at**	LOX	lysyl oxidase	13	0.8573960065841675	0.011178886	Yes
**201012_at**	ANXA1	annexin A1	14	0.8363754153251648	0.022663843	Yes
**212097_at**	CAV1	caveolin 1, caveolae protein, 22kDa	26	0.7442374229431152	0.03238035	Yes
**202270_at**	GBP1	guanylate binding protein 1, interferon-inducible, 67kDa /// guanylate binding protein 1, interferon-inducible, 67kDa	34	0.7032497525215149	0.041717015	Yes
**201110_s_at**	THBS1	thrombospondin 1	35	0.7013509273529053	0.05134784	Yes
**202269_x_at**	GBP1	guanylate binding protein 1, interferon-inducible, 67kDa /// guanylate binding protein 1, interferon-inducible, 67kDa	36	0.7007303833961487	0.060970142	Yes
**204298_s_at**	LOX	lysyl oxidase	46	0.6733514666557312	0.06980476	Yes
**201289_at**	CYR61	cysteine-rich, angiogenic inducer, 61	69	0.6132373213768005	0.077219166	Yes
**204517_at**	PPIC	peptidylprolyl isomerase C (cyclophilin C)	85	0.5832906365394592	0.084542595	Yes
**204490_s_at**	CD44	CD44 molecule (Indian blood group)	100	0.562261700630188	0.091623	Yes
**202856_s_at**	SLC16A3	solute carrier family 16 (monocarboxylic acid transporters), member 3	104	0.5567436218261719	0.09913086	Yes
**203065_s_at**	CAV1	caveolin 1, caveolae protein, 22kDa	108	0.5499752163887024	0.106545776	Yes
**202620_s_at**	PLOD2	procollagen-lysine, 2-oxoglutarate 5-dioxygenase 2	109	0.548766553401947	0.11408134	Yes
**201506_at**	TGFBI	transforming growth factor, beta-induced, 68kDa	115	0.5383884906768799	0.12124565	Yes
**205479_s_at**	PLAU	plasminogen activator, urokinase	127	0.5219894647598267	0.1279103	Yes
**202458_at**	PRSS23	protease, serine, 23	130	0.5206725597381592	0.13496858	Yes
**210978_s_at**	TAGLN2	transgelin 2	139	0.5071057677268982	0.14156608	Yes
**200660_at**	S100A11	S100 calcium binding protein A11 (calgizzarin)	146	0.5002387166023254	0.14816079	Yes
**209835_x_at**	CD44	CD44 molecule (Indian blood group)	154	0.4927939474582672	0.1546075	Yes
**202748_at**	GBP2	guanylate binding protein 2, interferon-inducible /// guanylate binding protein 2, interferon-inducible	157	0.49117493629455566	0.16126074	Yes
**201136_at**	PLP2	proteolipid protein 2 (colonic epithelium-enriched)	162	0.48545920848846436	0.16774398	Yes
**211668_s_at**	PLAU	plasminogen activator, urokinase /// plasminogen activator, urokinase	170	0.4788953363895416	0.17399986	Yes
**212014_x_at**	CD44	CD44 molecule (Indian blood group)	171	0.4788469970226288	0.1805753	Yes
**201438_at**	COL6A3	collagen, type VI, alpha 3	172	0.47728532552719116	0.18712929	Yes
**218880_at**	FOSL2	FOS-like antigen 2	174	0.4763893187046051	0.19362524	Yes
**213503_x_at**	ANXA2	annexin A2	176	0.4740018844604492	0.2000884	Yes
**202619_s_at**	PLOD2	procollagen-lysine, 2-oxoglutarate 5-dioxygenase 2	181	0.4703013002872467	0.2063635	Yes
**210764_s_at**	CYR61	cysteine-rich, angiogenic inducer, 61	183	0.4690304100513458	0.21275839	Yes

**210427_x_at**	ANXA2	annexin A2	186	0.4669896066188812	0.21907951	Yes
**217523_at**	CD44	CD44 molecule (Indian blood group)	187	0.46663156151771545	0.22548722	Yes
**202733_at**	P4HA2	procollagen-proline, 2-oxoglutarate 4-dioxygenase (proline 4-hydroxylase), alpha polypeptide II	188	0.4664992094039917	0.23189309	Yes
**201590_x_at**	ANXA2	annexin A2	193	0.46406906843185425	0.23808262	Yes
**201888_s_at**	IL13RA1	interleukin 13 receptor, alpha 1	224	0.44502687454223633	0.24282122	Yes
**202998_s_at**	LOXL2	lysyl oxidase-like 2	227	0.4441116750240326	0.24882817	Yes
**203827_at**	WIPI1	WD repeat domain, phosphoinositide interacting 1	232	0.4408568739891052	0.25469896	Yes
**206359_at**	SOCS3	suppressor of cytokine signaling 3	234	0.4392493665218353	0.2606849	Yes
**210916_s_at**	CD44	CD44 molecule (Indian blood group)	240	0.4329775273799896	0.26640174	Yes
**200916_at**	TAGLN2	transgelin 2	243	0.4296192228794098	0.2722097	Yes
**205266_at**	LIF	leukemia inhibitory factor (cholinergic differentiation factor)	254	0.4250584542751312	0.27758902	Yes
**213139_at**	SNAI2	snail homolog 2 (Drosophila)	255	0.42383459210395813	0.28340906	Yes
**212464_s_at**	FN1	fibronectin 1	257	0.42142003774642944	0.28915018	Yes
**204489_s_at**	CD44	CD44 molecule (Indian blood group)	270	0.4166492819786072	0.29432255	Yes
**208816_x_at**	ANXA2P2	annexin A2 pseudogene 2	271	0.4163329601287842	0.30003956	Yes
**213836_s_at**	WIPI1	WD repeat domain, phosphoinositide interacting 1	284	0.41079267859458923	0.30513152	Yes
**204362_at**	SKAP2	src kinase associated phosphoprotein 2	287	0.40917593240737915	0.31065875	Yes
**212063_at**	CD44	CD44 molecule (Indian blood group)	289	0.4086081385612488	0.31622395	Yes
**202948_at**	IL1R1	interleukin 1 receptor, type I	304	0.39582425355911255	0.32101884	Yes
**211612_s_at**	IL13RA1	interleukin 13 receptor, alpha 1 /// interleukin 13 receptor, alpha 1	306	0.3953624665737152	0.32640216	Yes
**203665_at**	HMOX1	heme oxygenase (decycling) 1	313	0.3906041383743286	0.33149138	Yes
**211719_x_at**	FN1	fibronectin 1 /// fibronectin 1	314	0.3903660178184509	0.3368518	Yes
**211368_s_at**	CASP1	caspase 1, apoptosis-related cysteine peptidase (interleukin 1, beta, convertase)	319	0.38932448625564575	0.34201497	Yes
**202375_at**	SEC24D	SEC24 related gene family, member D (S. cerevisiae)	320	0.3886062502861023	0.34735122	Yes
**209515_s_at**	RAB27A	RAB27A, member RAS oncogene family	325	0.3867569863796234	0.3524791	Yes
**201105_at**	LGALS1	lectin, galactoside-binding, soluble, 1 (galectin 1)	339	0.37973153591156006	0.3570988	Yes
**202071_at**	SDC4	syndecan 4 (amphiglycan, ryudocan)	356	0.3723054826259613	0.36147925	Yes
**217867_x_at**	BACE2	beta-site APP-cleaving enzyme 2	358	0.37180477380752563	0.36653906	Yes
**216442_x_at**	FN1	fibronectin 1	360	0.3714999258518219	0.3715947	Yes
**202393_s_at**	KLF10	Kruppel-like factor 10	364	0.3679405748844147	0.37650993	Yes
**201109_s_at**	THBS1	thrombospondin 1	371	0.36620810627937317	0.38126415	Yes
**204518_s_at**	PPIC	peptidylprolyl isomerase C (cyclophilin C)	380	0.36392879486083984	0.38589558	Yes
**209276_s_at**	GLRX	glutaredoxin (thioltransferase)	391	0.3603009283542633	0.3903857	Yes
**216899_s_at**	SKAP2	src kinase associated phosphoprotein 2	394	0.35950884222984314	0.39523092	Yes
**210495_x_at**	FN1	fibronectin 1	424	0.350460946559906	0.3987167	Yes
**213943_at**	TWIST1	twist homolog 1 (acrocephalosyndactyly 3; Saethre-Chotzen syndrome) (Drosophila)	430	0.34818288683891296	0.40326914	Yes
**217901_at**	DSG2	Desmoglein 2	435	0.3469637930393219	0.4078506	Yes
**209357_at**	CITED2	Cbp/p300-interacting transactivator, with Glu/Asp-rich carboxy-terminal domain, 2	442	0.34406736493110657	0.41230077	Yes

**202855_s_at**	SLC16A3	solute carrier family 16 (monocarboxylic acid transporters), member 3	462	0.3353567123413086	0.41603664	Yes
**221766_s_at**	FAM46A	family with sequence similarity 46, member A	466	0.334749698638916	0.4204961	Yes
**211367_s_at**	CASP1	caspase 1, apoptosis-related cysteine peptidase (interleukin 1, beta, convertase)	472	0.3322663903236389	0.42483	Yes
**202555_s_at**	MYLK	myosin, light polypeptide kinase /// myosin, light polypeptide kinase	474	0.33212363719940186	0.4293449	Yes
**206011_at**	CASP1	caspase 1, apoptosis-related cysteine peptidase (interleukin 1, beta, convertase)	475	0.33189988136291504	0.4339025	Yes
**201302_at**	ANXA4	annexin A4	478	0.33125653862953186	0.43835974	Yes
**201887_at**	IL13RA1	interleukin 13 receptor, alpha 1	507	0.32253432273864746	0.4415078	Yes
**221773_at**	ELK3	ELK3, ETS-domain protein (SRF accessory protein 2)	519	0.32019734382629395	0.44540146	Yes
**201301_s_at**	ANXA4	annexin A4	524	0.3192821145057678	0.44960278	Yes
**202087_s_at**	CTSL	cathepsin L	537	0.31377771496772766	0.45336255	Yes
**210845_s_at**	PLAUR	plasminogen activator, urokinase receptor	540	0.31307563185691833	0.45757017	Yes
**208540_x_at**	S100A11	S100 calcium binding protein A11 (calgizzarin)	564	0.3052438497543335	0.4607095	Yes
**202180_s_at**	MVP	major vault protein	570	0.3041495680809021	0.4646573	Yes
**202766_s_at**	FBN1	fibrillin 1	572	0.3039049506187439	0.46878472	Yes
**218341_at**	PPCS	phosphopantothenoylcysteine synthetase	574	0.3035537600517273	0.47290733	Yes
**201474_s_at**	ITGA3	integrin, alpha 3 (antigen CD49C, alpha 3 subunit of VLA-3 receptor)	586	0.2990971803665161	0.47651124	Yes
**201389_at**	ITGA5	integrin, alpha 5 (fibronectin receptor, alpha polypeptide)	596	0.2974662482738495	0.48018426	Yes
**204361_s_at**	SKAP2	src kinase associated phosphoprotein 2	615	0.2939990162849426	0.48339796	Yes
**218194_at**	REXO2	REX2, RNA exonuclease 2 homolog (S. cerevisiae)	619	0.2932628393173218	0.48728773	Yes
**31845_at**	ELF4	E74-like factor 4 (ets domain transcription factor)	650	0.2853066027164459	0.4898331	Yes
**203282_at**	GBE1	glucan (1,4-alpha-), branching enzyme 1 (glycogen branching enzyme, Andersen disease, glycogen storage disease type IV)	657	0.2835060656070709	0.49345165	Yes
**203430_at**	HEBP2	heme binding protein 2	662	0.2830619215965271	0.4971556	Yes
**217744_s_at**	PERP	PERP, TP53 apoptosis effector	673	0.28056660294532776	0.5005508	Yes
**209970_x_at**	CASP1	caspase 1, apoptosis-related cysteine peptidase (interleukin 1, beta, convertase)	686	0.27736103534698486	0.5038105	Yes
**210904_s_at**	IL13RA1	interleukin 13 receptor, alpha 1	723	0.26761722564697266	0.50583845	Yes
**203510_at**	MET	met proto-oncogene (hepatocyte growth factor receptor)	731	0.266333669424057	0.5091755	Yes
**204158_s_at**	TCIRG1	T-cell, immune regulator 1, ATPase, H+ transporting, lysosomal V0 subunit A3	751	0.2622676491737366	0.5119077	Yes
**200838_at**	CTSB	cathepsin B	752	0.26218244433403015	0.51550794	Yes
**207980_s_at**	CITED2	Cbp/p300-interacting transactivator, with Glu/Asp-rich carboxy-terminal domain, 2	759	0.2614690661430359	0.5188239	Yes
**202862_at**	FAH	fumarylacetoacetate hydrolase (fumarylacetoacetase)	812	0.25204724073410034	0.5199061	Yes
**203650_at**	PROCR	protein C receptor, endothelial (EPCR)	846	0.24640558660030365	0.52178	Yes
**202014_at**	PPP1R15A	protein phosphatase 1, regulatory (inhibitor) subunit 15A	848	0.24615949392318726	0.5251145	Yes

**201041_s_at**	DUSP1	dual specificity phosphatase 1	853	0.24571162462234497	0.52830553	Yes
**204463_s_at**	EDNRA	endothelin receptor type A	863	0.24430687725543976	0.5312486	Yes
**209154_at**	TAX1BP3	Tax1 (human T-cell leukemia virus type I) binding protein 3	864	0.2442103624343872	0.53460205	Yes
**201809_s_at**	ENG	endoglin (Osler-Rendu-Weber syndrome 1)	868	0.243181511759758	0.5378041	Yes
**206662_at**	GLRX	glutaredoxin (thioltransferase)	872	0.24281901121139526	0.54100126	Yes
**215464_s_at**	TAX1BP3	Tax1 (human T-cell leukemia virus type I) binding protein 3	873	0.24280719459056854	0.5443354	Yes
**203593_at**	CD2AP	CD2-associated protein	878	0.24078933894634247	0.5474589	Yes
**209310_s_at**	CASP4	caspase 4, apoptosis-related cysteine peptidase	882	0.23990480601787567	0.55061597	Yes
**202949_s_at**	FHL2	four and a half LIM domains 2	889	0.23861421644687653	0.55361813	Yes
**201531_at**	ZFP36	zinc finger protein 36, C3H type, homolog (mouse)	890	0.2385438233613968	0.55689377	Yes
**209417_s_at**	IFI35	interferon-induced protein 35	899	0.2376018464565277	0.5597905	Yes
**218109_s_at**	MFSD1	major facilitator superfamily domain containing 1	906	0.23637214303016663	0.5627618	Yes
**208944_at**	TGFBR2	transforming growth factor, beta receptor II (70/80kDa)	908	0.23630952835083008	0.565961	Yes
**209264_s_at**	TSPAN4	tetraspanin 4	912	0.2361619770526886	0.5690667	Yes
**201925_s_at**	CD55	CD55 molecule, decay accelerating factor for complement (Cromer blood group)	926	0.23453949391841888	0.57169265	Yes
**208864_s_at**	TXN	thioredoxin	932	0.23376061022281647	0.5746739	Yes
**204005_s_at**	PAWR	PRKC, apoptosis, WT1, regulator	933	0.2336987406015396	0.577883	Yes
**210951_x_at**	RAB27A	RAB27A, member RAS oncogene family	940	0.23244047164916992	0.5808003	Yes
**205542_at**	STEAP1	six transmembrane epithelial antigen of the prostate 1	941	0.23221173882484436	0.583989	Yes
**213640_s_at**	LOX	lysyl oxidase	942	0.23160390555858612	0.58716935	Yes
**202531_at**	IRF1	interferon regulatory factor 1	975	0.22559523582458496	0.58880323	Yes
**211366_x_at**	CASP1	caspase 1, apoptosis-related cysteine peptidase (interleukin 1, beta, convertase)	979	0.22538000345230103	0.5917609	Yes
**200701_at**	NPC2	Niemann-Pick disease, type C2	993	0.22219493985176086	0.5942173	Yes
**207992_s_at**	AMPD3	adenosine monophosphate deaminase (isoform E)	994	0.22194162011146545	0.59726495	Yes
**212472_at**	MICAL2	microtubule associated monoxygenase, calponin and LIM domain containing 2	996	0.2217075377702713	0.60026366	Yes
**204464_s_at**	EDNRA	endothelin receptor type A	1021	0.21836185455322266	0.6021642	Yes
**211926_s_at**	MYH9	myosin, heavy polypeptide 9, non-muscle	1029	0.2174818366765976	0.60483044	Yes
**218164_at**	SPATA20	spermatogenesis associated 20	1056	0.21344247460365295	0.6065719	Yes
**203851_at**	IGFBP6	insulin-like growth factor binding protein 6	1057	0.21328292787075043	0.6095007	Yes
**204004_at**	PAWR	PRKC, apoptosis, WT1, regulator	1061	0.2129514068365097	0.61228764	Yes
**200839_s_at**	CTSB	cathepsin B	1091	0.20926234126091003	0.61383456	Yes
**200766_at**	CTSD	cathepsin D (lysosomal aspartyl peptidase)	1097	0.20890501141548157	0.61647445	Yes
**211730_s_at**	POLR2L	polymerase (RNA) II (DNA directed) polypeptide L, 7.6kDa /// polymerase (RNA) II (DNA directed) polypeptide L, 7.6kDa	1100	0.20860786736011505	0.6192475	Yes
**201288_at**	ARHGDIB	Rho GDP dissociation inhibitor (GDI) beta	1101	0.20849205553531647	0.6221105	Yes
**201108_s_at**	THBS1	thrombospondin 1	1104	0.20820096135139465	0.624878	Yes
**217478_s_at**	HLA-DMA	major histocompatibility complex, class II, DM alpha	1123	0.20616960525512695	0.6268856	Yes
**203912_s_at**	DNASE1L1	deoxyribonuclease I-like 1	1125	0.2061261683702469	0.6296703	Yes

**217691_x_at**	SLC16A3	solute carrier family 16 (monocarboxylic acid transporters), member 3	1144	0.20370571315288544	0.6316441	Yes
**203233_at**	IL4R	interleukin 4 receptor	1149	0.2031058669090271	0.63425016	Yes
**213416_at**	ITGA4	integrin, alpha 4 (antigen CD49D, alpha 4 subunit of VLA-4 receptor)	1167	0.20107731223106384	0.63623357	Yes
**212245_at**	MCFD2	multiple coagulation factor deficiency 2	1170	0.2008926421403885	0.6389007	Yes
**219229_at**	SLCO3A1	solute carrier organic anion transporter family, member 3A1	1185	0.1990458071231842	0.64099354	Yes
**213425_at**	WNT5A	wingless-type MMTV integration site family, member 5A /// wingless-type MMTV integration site family, member 5A	1230	0.19238199293613434	0.64162236	Yes
**200931_s_at**	VCL	vinculin	1249	0.19057346880435944	0.6434158	Yes
**205990_s_at**	WNT5A	wingless-type MMTV integration site family, member 5A	1264	0.18889759480953217	0.64536923	Yes
**209263_x_at**	TSPAN4	tetraspanin 4	1298	0.18532811105251312	0.6464045	Yes
**218217_at**	SCPEP1	serine carboxypeptidase 1	1334	0.18199490010738373	0.64730245	Yes
**212647_at**	RRAS	related RAS viral (r-ras) oncogene homolog	1342	0.18132968246936798	0.6494722	Yes
**213274_s_at**	CTSB	cathepsin B	1351	0.18011315166950226	0.6515795	Yes
**202201_at**	BLVRB	biliverdin reductase B (flavin reductase (NADPH))	1373	0.17808659374713898	0.65306425	Yes
**209238_at**	STX3	syntaxin 3	1384	0.17728596925735474	0.6550412	Yes
**201412_at**	LRP10	low density lipoprotein receptor-related protein 10	1394	0.17642341554164886	0.6570521	Yes
**220272_at**	BNC2	basonuclin 2	1417	0.1749013215303421	0.6584474	Yes
**202052_s_at**	RAI14	retinoic acid induced 14	1428	0.17400670051574707	0.6603793	Yes
**213275_x_at**	CTSB	cathepsin B	1433	0.17361535131931305	0.6625804	Yes
**212246_at**	MCFD2	multiple coagulation factor deficiency 2	1457	0.17218783497810364	0.6638926	Yes
**204475_at**	MMP1	matrix metallopeptidase 1 (interstitial collagenase)	1465	0.17168064415454865	0.66592985	Yes
**205812_s_at**	TMED9	transmembrane emp24 protein transport domain containing 9	1490	0.16931109130382538	0.6671569	Yes
**201926_s_at**	CD55	CD55 molecule, decay accelerating factor for complement (Cromer blood group)	1501	0.16855520009994507	0.669014	Yes
**209584_x_at**	APOBEC3C	apolipoprotein B mRNA editing enzyme, catalytic polypeptide-like 3C	1513	0.1670963317155838	0.6708053	Yes
**201883_s_at**	B4GALT1	UDP-Gal:betaGlcNAc beta 1,4-galactosyltransferase, polypeptide 1	1529	0.16573166847229004	0.6723949	Yes
**37028_at**	PPP1R15A	protein phosphatase 1, regulatory (inhibitor) subunit 15A	1531	0.16561362147331238	0.6746233	Yes
**201200_at**	CREG1	cellular repressor of E1A-stimulated genes 1	1535	0.1652938276529312	0.67675585	Yes
**203085_s_at**	TGFB1	transforming growth factor, beta 1 (Camurati-Engelmann disease)	1571	0.16259360313415527	0.67738736	Yes
**214701_s_at**	FN1	fibronectin 1	1573	0.16239358484745026	0.67957157	Yes
**213603_s_at**	RAC2	ras-related C3 botulinum toxin substrate 2 (rho family, small GTP binding protein Rac2)	1577	0.16217166185379028	0.68166125	Yes
**205422_s_at**	ITGBL1	integrin, beta-like 1 (with EGF-like repeat domains)	1599	0.16050466895103455	0.68290454	Yes
**209365_s_at**	ECM1	extracellular matrix protein 1	1600	0.16050222516059875	0.68510854	Yes
**211924_s_at**	PLAUR	plasminogen activator, urokinase receptor /// plasminogen activator, urokinase receptor	1608	0.15966731309890747	0.68698084	Yes

**201189_s_at**	ITPR3	inositol 1,4,5-triphosphate receptor, type 3	1617	0.15890559554100037	0.68879694	Yes
**219561_at**	COPZ2	coatomer protein complex, subunit zeta 2	1620	0.1586531549692154	0.69088405	Yes
**203108_at**	GPRC5A	G protein-coupled receptor, family C, group 5, member A	1631	0.1575879007577896	0.69259053	Yes
**205452_at**	PIGB	phosphatidylinositol glycan, class B	1645	0.1567411869764328	0.6941481	Yes
**202804_at**	ABCC1	ATP-binding cassette, sub-family C (CFTR/MRP), member 1	1669	0.1539832353591919	0.6952104	Yes
**205409_at**	FOSL2	FOS-like antigen 2	1694	0.1528746634721756	0.6962117	Yes
**214321_at**	NOV	nephroblastoma overexpressed gene	1699	0.15258371829986572	0.698124	Yes
**206027_at**	S100A3	S100 calcium binding protein A3	1705	0.15203261375427246	0.69998294	Yes
**201044_x_at**	DUSP1	dual specificity phosphatase 1	1715	0.15151840448379517	0.7016518	Yes
**201188_s_at**	ITPR3	inositol 1,4,5-triphosphate receptor, type 3	1729	0.1504964977502823	0.7031237	Yes
**201939_at**	PLK2	polo-like kinase 2 (Drosophila)	1746	0.14940042793750763	0.7044433	Yes
**205798_at**	IL7R	interleukin 7 receptor /// interleukin 7 receptor	1777	0.14765189588069916	0.70509833	Yes
**209514_s_at**	RAB27A	RAB27A, member RAS oncogene family	1802	0.1464088410139084	0.7060109	Yes
**205945_at**	IL6R	interleukin 6 receptor /// interleukin 6 receptor	1821	0.14499707520008087	0.7071785	Yes
**213506_at**	F2RL1	coagulation factor II (thrombin) receptor-like 1	1846	0.14350001513957977	0.708051	Yes
**208757_at**	TMED9	transmembrane emp24 protein transport domain containing 9	1855	0.14292097091674805	0.70964766	Yes
**212509_s_at**	MXRA7	matrix-remodelling associated 7	1867	0.14219358563423157	0.711097	Yes
**216609_at**	TXN	Thioredoxin	2073	0.13200226426124573	0.7035313	No
**209193_at**	PIM1	pim-1 oncogene /// pim-1 oncogene	2116	0.12991943955421448	0.70339394	No
**200904_at**	HLA-E	major histocompatibility complex, class I, E	2136	0.12896126508712769	0.70429564	No
**205032_at**	ITGA2	integrin, alpha 2 (CD49B, alpha 2 subunit of VLA-2 receptor)	2151	0.12818500399589539	0.70541537	No
**35820_at**	GM2A	GM2 ganglioside activator	2154	0.12809701263904572	0.70708287	No
**214866_at**	PLAUR	plasminogen activator, urokinase receptor	2200	0.12594032287597656	0.7067536	No
**209707_at**	PIGK	phosphatidylinositol glycan, class K	2251	0.12367995083332062	0.7061646	No
**209318_x_at**	PLAGL1	pleiomorphic adenoma gene-like 1	2292	0.12187694758176804	0.70600826	No
**201278_at**	DAB2	Disabled homolog 2, mitogen-responsive phosphoprotein (Drosophila)	2307	0.12099678814411163	0.7070293	No
**218530_at**	FHOD1	formin homology 2 domain containing 1	2367	0.11830638349056244	0.70595473	No
**214446_at**	ELL2	elongation factor, RNA polymerase II, 2	2373	0.11808978766202927	0.7073476	No
**203438_at**	STC2	stanniocalcin 2	2439	0.11507274955511093	0.70595413	No
**210876_at**	ANXA2P1	annexin A2 pseudogene 1	2469	0.1139754056930542	0.70619255	No
**200905_x_at**	HLA-E	major histocompatibility complex, class I, E	2485	0.1133526936173439	0.7070629	No
**212737_at**	GM2A	GM2 ganglioside activator	2546	0.11105906218290329	0.70584303	No
**216235_s_at**	EDNRA	endothelin receptor type A	2619	0.10840295255184174	0.7040378	No
**217456_x_at**	HLA-E	major histocompatibility complex, class I, E	2621	0.10821345448493958	0.705478	No
**208837_at**	TMED3	transmembrane emp24 protein transport domain containing 3	2695	0.10496263206005096	0.7035797	No
**206360_s_at**	SOCS3	suppressor of cytokine signaling 3	2721	0.1040676012635231	0.7038651	No
**213187_x_at**	FTL	ferritin, light polypeptide	2791	0.10171438753604889	0.7021052	No
**202765_s_at**	FBN1	fibrillin 1	2814	0.10058450698852539	0.70247996	No
**205891_at**	ADORA2B	adenosine A2b receptor	2853	0.09897909313440323	0.70210075	No

**218826_at**	SLC35F2	solute carrier family 35, member F2	2871	0.09786799550056458	0.70266694	No
**208683_at**	CAPN2	calpain 2, (m/II) large subunit	2880	0.09746172279119492	0.70363927	No
**218881_s_at**	FOSL2	FOS-like antigen 2	2886	0.09719928354024887	0.70474523	No
**212185_x_at**	MT2A	metallothionein 2A	2927	0.09557738900184631	0.7042278	No
**202026_at**	SDHD	succinate dehydrogenase complex, subunit D, integral membrane protein	2982	0.09355971217155457	0.70304215	No
**202805_s_at**	ABCC1	ATP-binding cassette, sub-family C (CFTR/MRP), member 1	3068	0.09100973606109619	0.70040333	No
**206693_at**	IL7	interleukin 7	3098	0.09010353684425354	0.7003139	No
**201953_at**	CIB1	calcium and integrin binding 1 (calmyrin)	3101	0.0899982899427414	0.7014583	No
**202686_s_at**	AXL	AXL receptor tyrosine kinase	3113	0.08957750350236893	0.7021851	No
**211742_s_at**	EVI2B	ecotropic viral integration site 2B /// ecotropic viral integration site 2B	3189	0.08771281689405441	0.6999585	No
**203490_at**	ELF4	E74-like factor 4 (ets domain transcription factor)	3352	0.0831630527973175	0.69368935	No
**205884_at**	ITGA4	integrin, alpha 4 (antigen CD49D, alpha 4 subunit of VLA-4 receptor)	3412	0.08188691735267639	0.69211465	No
**212788_x_at**	FTL	ferritin, light polypeptide	3434	0.08132059872150421	0.69227064	No
**201280_s_at**	DAB2	disabled homolog 2, mitogen-responsive phosphoprotein (Drosophila)	3451	0.08059244602918625	0.6926454	No
**218498_s_at**	ERO1L	ERO1-like (S. cerevisiae)	3466	0.07997353374958038	0.6931031	No
**207438_s_at**	SNUPN	snurportin 1	3505	0.07897774875164032	0.69244915	No
**210757_x_at**	DAB2	disabled homolog 2, mitogen-responsive phosphoprotein (Drosophila)	3523	0.07833681255578995	0.6927472	No
**213865_at**	DCBLD2	discoidin, CUB and LCCL domain containing 2	3551	0.07742627710103989	0.69257516	No
**217168_s_at**	HERPUD1	homocysteine-inducible, endoplasmic reticulum stress-inducible, ubiquitin-like domain member 1	3607	0.07589632272720337	0.69110125	No
**202974_at**	MPP1	membrane protein, palmitoylated 1, 55kDa	3653	0.074372798204422	0.6900639	No
**217678_at**	SLC7A11	solute carrier family 7, (cationic amino acid transporter, y+ system) member 11	3698	0.0731629952788353	0.6890556	No
**202768_at**	FOSB	FBJ murine osteosarcoma viral oncogene homolog B	3786	0.07106613367795944	0.6860514	No
**201605_x_at**	CNN2	calponin 2	3810	0.0705334022641182	0.6859678	No
**205885_s_at**	ITGA4	integrin, alpha 4 (antigen CD49D, alpha 4 subunit of VLA-4 receptor)	3924	0.06727144867181778	0.68172204	No
**203439_s_at**	STC2	stanniocalcin 2	3947	0.06677090376615524	0.6816325	No
**202997_s_at**	LOXL2	lysyl oxidase-like 2	3962	0.06646708399057388	0.68190473	No
**207396_s_at**	ALG3	asparagine-linked glycosylation 3 homolog (S. cerevisiae, alpha-1,3-mannosyltransferase)	4006	0.06546047329902649	0.6808365	No
**207002_s_at**	PLAGL1	pleiomorphic adenoma gene-like 1	4037	0.06496132165193558	0.6803561	No
**205743_at**	STAC	SH3 and cysteine rich domain	4049	0.06460411846637726	0.68074	No
**214927_at**	ITGBL1	Integrin, beta-like 1 (with EGF-like repeat domains)	4062	0.06416531652212143	0.6810721	No
**205503_at**	PTPN14	protein tyrosine phosphatase, non-receptor type 14	4099	0.06314267218112946	0.68029225	No
**201279_s_at**	DAB2	disabled homolog 2, mitogen-responsive phosphoprotein (Drosophila)	4117	0.06266304850578308	0.68037504	No
**201107_s_at**	THBS1	thrombospondin 1	4175	0.061346568167209625	0.67860985	No

**202275_at**	G6PD	glucose-6-phosphate dehydrogenase	4263	0.05952858924865723	0.6754472	No
**209921_at**	SLC7A11	solute carrier family 7, (cationic amino acid transporter, y+ system) member 11	4311	0.05858634039759636	0.67410153	No
**213596_at**	CASP4	caspase 4, apoptosis-related cysteine peptidase	4314	0.05852356180548668	0.6748137	No
**200924_s_at**	SLC3A2	solute carrier family 3 (activators of dibasic and neutral amino acid transport), member 2	4475	0.05526905879378319	0.668253	No
**204501_at**	NOV	nephroblastoma overexpressed gene	4760	0.04981270805001259	0.65594465	No
**201502_s_at**	NFKBIA	nuclear factor of kappa light polypeptide gene enhancer in B-cells inhibitor, alpha	4772	0.04952409118413925	0.6561215	No
**212614_at**	ARID5B	AT rich interactive domain 5B (MRF1-like)	4898	0.047140344977378845	0.6510503	No
**215561_s_at**	IL1R1	interleukin 1 receptor, type I	5155	0.042823318392038345	0.63992697	No
**207334_s_at**	TGFBR2	transforming growth factor, beta receptor II (70/80kDa)	5176	0.042487259954214096	0.63959545	No
**214656_x_at**	MYO1C	myosin IC	5242	0.04141290858387947	0.6371905	No
**217685_at**	SLC16A3	Solute carrier family 16 (monocarboxylic acid transporters), member 3	5290	0.04045959189534187	0.6355959	No
**207419_s_at**	RAC2	ras-related C3 botulinum toxin substrate 2 (rho family, small GTP binding protein Rac2)	5418	0.03848147764801979	0.6303144	No
**215209_at**	SEC24D	SEC24 related gene family, member D (S. cerevisiae)	5420	0.038450032472610474	0.6307967	No
**202586_at**	POLR2L	polymerase (RNA) II (DNA directed) polypeptide L, 7.6kDa	5445	0.03810495138168335	0.63022196	No
**211631_x_at**	B4GALT1	UDP-Gal:betaGlcNAc beta 1,4-galactosyltransferase, polypeptide 1 /// UDP-Gal:betaGlcNAc beta 1,4- galactosyltransferase, polypeptide 1	5602	0.03600631654262543	0.62357974	No
**201717_at**	MRPL49	mitochondrial ribosomal protein L49	5644	0.03554988652467728	0.62219226	No
**203789_s_at**	SEMA3C	sema domain, immunoglobulin domain (Ig), short basic domain, secreted, (semaphorin) 3C	5660	0.035209253430366516	0.6219895	No
**212473_s_at**	MICAL2	microtubule associated monoxygenase, calponin and LIM domain containing 2	5713	0.03430015593767166	0.6200816	No
**210664_s_at**	TFPI	tissue factor pathway inhibitor (lipoprotein-associated coagulation inhibitor)	5716	0.0342230349779129	0.6204601	No
**208232_x_at**	NRG1	neuregulin 1	5733	0.03408794105052948	0.6201962	No
**201945_at**	FURIN	furin (paired basic amino acid cleaving enzyme)	5966	0.031133875250816345	0.61001027	No
**213813_x_at**	FTL	Ferritin, light polypeptide	6029	0.030399037525057793	0.60759133	No
**206200_s_at**	ANXA11	annexin A11	6197	0.028204642236232758	0.60033876	No
**220016_at**	AHNAK	AHNAK nucleoprotein (desmoyokin)	6199	0.028164714574813843	0.60067976	No
**219431_at**	ARHGAP10	Rho GTPase activating protein 10	6202	0.028043173253536224	0.60097337	No
**205808_at**	ASPH	aspartate beta-hydroxylase	6360	0.026052841916680336	0.5941487	No
**209491_s_at**	AMPD3	adenosine monophosphate deaminase (isoform E)	6370	0.025883030146360397	0.59409237	No
**205828_at**	MMP3	matrix metallopeptidase 3 (stromelysin 1, progelatinase)	6373	0.025840461254119873	0.59435576	No
**216985_s_at**	STX3	syntaxin 3	6440	0.02493244968354702	0.59167874	No

**203258_at**	DRAP1	DR1-associated protein 1 (negative cofactor 2 alpha)	6674	0.02206147089600563	0.5813225	No
**204049_s_at**	PHACTR2	phosphatase and actin regulator 2	6789	0.020543495193123817	0.5763893	No
**201870_at**	TOMM34	translocase of outer mitochondrial membrane 34	7050	0.017489025369286537	0.56473505	No
**213522_s_at**	SLC16A3	Solute carrier family 16 (monocarboxylic acid transporters), member 3	7078	0.01720244251191616	0.5637361	No
**203586_s_at**	ARL4D	ADP-ribosylation factor-like 4D	7241	0.015457389876246452	0.5565372	No
**211320_s_at**	PTPRU	protein tyrosine phosphatase, receptor type, U	7295	0.014945099130272865	0.55431783	No
**206429_at**	F2RL1	coagulation factor II (thrombin) receptor-like 1	7313	0.014809953048825264	0.5537435	No
**211671_s_at**	NR3C1	)	7329	0.014647078700363636	0.55325836	No
**213862_at**	TSPAN4	Tetraspanin 4	7501	0.01312720961868763	0.5456158	No
**206382_s_at**	BDNF	brain-derived neurotrophic factor	7509	0.013045595958828926	0.5454747	No
**201187_s_at**	ITPR3	inositol 1,4,5-triphosphate receptor, type 3	7518	0.012951699085533619	0.54528654	No
**214237_x_at**	PAWR	PRKC, apoptosis, WT1, regulator	7607	0.0121694877743721	0.54142785	No
**215818_at**	NUDT7	nudix (nucleoside diphosphate linked moiety X)-type motif 7	7769	0.010669912211596966	0.534209	No
**205066_s_at**	ENPP1	ectonucleotide pyrophosphatase/phosphodiesterase 1	7777	0.010568496771156788	0.5340339	No
**208600_s_at**	GPR39	G protein-coupled receptor 39	7779	0.010554437525570393	0.5341331	No
**215775_at**	THBS1	Thrombospondin 1	7843	0.009911179542541504	0.53138703	No
**200752_s_at**	CAPN1	calpain 1, (mu/I) large subunit	8007	0.008342987857758999	0.52404475	No
**33646_g_at**	GM2A	GM2 ganglioside activator	8009	0.008323464542627335	0.5241133	No
**203788_s_at**	SEMA3C	sema domain, immunoglobulin domain (Ig), short basic domain, secreted, (semaphorin) 3C	8183	0.006880403961986303	0.5162934	No
**32811_at**	MYO1C	myosin IC	8296	0.0060845124535262585	0.51125324	No
**201865_x_at**	NR3C1	nuclear receptor subfamily 3, group C, member 1 (glucocorticoid receptor)	8373	0.005325019359588623	0.5078495	No
**215891_s_at**	GM2A	GM2 ganglioside activator	8454	0.004670027177780867	0.5042538	No
**208230_s_at**	NRG1	neuregulin 1	8750	0.0022610221058130264	0.49078926	No
**209727_at**	GM2A	GM2 ganglioside activator	8843	0.0014507316518574953	0.4866004	No
**203084_at**	TGFB1	transforming growth factor, beta 1 (Camurati-Engelmann disease)	8946	5,84E+11	0.48194215	No
**211124_s_at**	KITLG	KIT ligand	9153	-9,79E+11	0.47253156	No
**203680_at**	PRKAR2B	protein kinase, cAMP-dependent, regulatory, type II, beta	9231	-0.001533363712951541	0.46903005	No
**214105_at**	SOCS3	suppressor of cytokine signaling 3	9254	-0.001723128603771329	0.46804726	No
**215727_x_at**	CHD3	Chromodomain helicase DNA binding protein 3	9285	- 0.0019444242352619767	0.46670154	No
**207133_x_at**	ALPK1	alpha-kinase 1	9297	- 0.0020472484175115824	0.4662264	No
**201808_s_at**	ENG	endoglin (Osler-Rendu-Weber syndrome 1)	9439	- 0.0030464204028248787	0.45981783	No
**216466_at**	NAV3	neuron navigator 3	9520	- 0.0036111201625317335	0.45620757	No
**207528_s_at**	SLC7A11	solute carrier family 7, (cationic amino acid transporter, y+ system) member 11	9538	-0.003764189314097166	0.45548156	No
**202999_s_at**	LOXL2	lysyl oxidase-like 2	9655	-0.004631041083484888	0.4502384	No
**207943_x_at**	PLAGL1	pleiomorphic adenoma gene-like 1	9742	-0.005382364150136709	0.44637802	No
**207029_at**	KITLG	KIT ligand	9762	-0.005489609204232693	0.44558418	No
**206237_s_at**	NRG1	neuregulin 1	9777	-0.005576720926910639	0.4450203	No
**214702_at**	FN1	fibronectin 1	9804	- 0.0057636587880551815	0.44391	No

**214445_at**	ELL2	elongation factor, RNA polymerase II, 2	9822	-0.005860977806150913	0.44321278	No
**216405_at**	LGALS1	lectin, galactoside-binding, soluble, 1 (galectin 1)	10010	-0.007182544097304344	0.43475658	No
**207017_at**	RAB27B	RAB27B, member RAS oncogene family	10072	-0.007539245299949455	0.43206948	No
**200930_s_at**	VCL	vinculin	10115	-0.007873965427279472	0.43025622	No
**206750_at**	MAFK	v-maf musculoaponeurotic fibrosarcoma oncogene homolog K (avian)	10303	-0.009177165105938911	0.4218274	No
**214090_at**	PAWR	PRKC, apoptosis, WT1, regulator	10522	-0.010483972728252411	0.41199836	No
**213816_s_at**	MET	met proto-oncogene (hepatocyte growth factor receptor)	10655	-0.011435887776315212	0.4061167	No
**208340_at**	CASP4	Caspase 4, apoptosis-related cysteine peptidase	10744	-0.012238416820764542	0.40225893	No
**207301_at**	EFNA5	ephrin-A5	10867	-0.01303959172219038	0.39685678	No
**216627_s_at**	B4GALT1	UDP-Gal:betaGlcNAc beta 1,4-galactosyltransferase, polypeptide 1	10875	-0.013069703243672848	0.396716	No
**218651_s_at**	LARP6	La ribonucleoprotein domain family, member 6	10925	-0.01344485767185688	0.39465898	No
**207284_s_at**	ASPH	aspartate beta-hydroxylase	11018	-0.014021249487996101	0.39064273	No
**212642_s_at**	HIVEP2	human immunodeficiency virus type I enhancer binding protein 2	11112	-0.014589029364287853	0.38658854	No
**216062_at**	CD44	CD44 molecule (Indian blood group)	11117	-0.014604855328798294	0.3866061	No
**209946_at**	VEGFC	vascular endothelial growth factor C	11145	-0.014780386351048946	0.38557386	No
**203587_at**	ARL4D	ADP-ribosylation factor-like 4D	11246	-0.015346121042966843	0.38120982	No
**201995_at**	EXT1	exostoses (multiple) 1	11363	-0.016085870563983917	0.37612396	No
**209406_at**	BAG2	BCL2-associated athanogene 2	11393	-0.01629002019762993	0.37502098	No
**215641_at**	SEC24D	SEC24 related gene family, member D (S. cerevisiae)	11461	-0.01677914336323738	0.37218627	No
**216974_at**	KITLG	KIT ligand	11634	-0.017827462404966354	0.36456248	No
**201866_s_at**	NR3C1	nuclear receptor subfamily 3, group C, member 1 (glucocorticoid receptor)	11696	-0.018290000036358833	0.36202303	No
**203741_s_at**	ADCY7	adenylate cyclase 7	11756	-0.018699292093515396	0.35958067	No
**203435_s_at**	MME	membrane metallo-endopeptidase (neutral endopeptidase, enkephalinase, CALLA, CD10)	11846	-0.01928236335515976	0.3557739	No
**213258_at**	TFPI	tissue factor pathway inhibitor (lipoprotein-associated coagulation inhibitor)	12019	-0.02044552005827427	0.34818605	No
**210542_s_at**	SLCO3A1	solute carrier organic anion transporter family, member 3A1	12041	-0.02058481238782406	0.347508	No
**206127_at**	ELK3	ELK3, ETS-domain protein (SRF accessory protein 2)	12050	-0.020647713914513588	0.34742555	No
**209529_at**	PPAP2C	phosphatidic acid phosphatase type 2C	12126	-0.021047472953796387	0.3442835	No
**204048_s_at**	PHACTR2	phosphatase and actin regulator 2	12127	-0.021068155765533447	0.3445728	No
**202685_s_at**	AXL	AXL receptor tyrosine kinase	12209	-0.021637901663780212	0.34116438	No
**206079_at**	CHML	choroideremia-like (Rab escort protein 2)	12274	-0.022142870351672173	0.33854058	No
**208378_x_at**	FGF5	fibroblast growth factor 5	12293	-0.022293366491794586	0.33802325	No
**211986_at**	AHNAK	AHNAK nucleoprotein (desmoyokin)	12368	-0.02284310571849346	0.33495158	No
**205935_at**	FOXF1	forkhead box F1	12539	-0.02408873848617077	0.32750526	No
**206757_at**	PDE5A	phosphodiesterase 5A, cGMP-specific	12626	-0.024625500664114952	0.3239091	No
**205017_s_at**	MBNL2	muscleblind-like 2 (Drosophila)	12767	-0.0255371555685997	0.3178551	No

**215182_x_at**	MYH9	Myosin, heavy polypeptide 9, non-muscle	12793	-0.02568201720714569	0.31706405	No
**203434_s_at**	MME	membrane metallo-endopeptidase (neutral endopeptidase, enkephalinase, CALLA, CD10)	12905	-0.026403814554214478	0.31234863	No
**216321_s_at**	NR3C1	nuclear receptor subfamily 3, group C, member 1 (glucocorticoid receptor)	12906	-0.026409391313791275	0.31271127	No
**210310_s_at**	FGF5	fibroblast growth factor 5	12933	-0.02657688595354557	0.3118868	No
**221292_at**	PTCH2	patched homolog 2 (Drosophila)	13481	-0.03037938103079796	0.28727993	No
**207446_at**	TLR6	toll-like receptor 6	13575	-0.031021419912576675	0.28345138	No
**216056_at**	CD44	CD44 molecule (Indian blood group)	13599	-0.031193288043141365	0.2828275	No
**221283_at**	RUNX2	runt-related transcription factor 2	13654	-0.031551145017147064	0.2807904	No
**215890_at**	GM2A	GM2 ganglioside activator	13724	-0.032052990049123764	0.27807394	No
**204047_s_at**	PHACTR2	phosphatase and actin regulator 2	13742	-0.03212316334247589	0.27773732	No
**212960_at**	TBC1D9	TBC1 domain family, member 9	13803	-0.03253284841775894	0.2754392	No
**216994_s_at**	RUNX2	runt-related transcription factor 2	13867	-0.03297000005841255	0.27300984	No
**220169_at**	TMEM156	transmembrane protein 156	13881	-0.0330638587474823	0.27286914	No
**210349_at**	CAMK4	calcium/calmodulin-dependent protein kinase IV	14123	-0.03470020741224289	0.26232043	No
**215913_s_at**	GULP1	GULP, engulfment adaptor PTB domain containing 1	14464	-0.03726976737380028	0.24727798	No
**214378_at**	TFPI	tissue factor pathway inhibitor (lipoprotein-associated coagulation inhibitor)	14615	-0.0385291688144207	0.24094489	No
**217670_at**	TSPAN4	Tetraspanin 4	14888	-0.04046539217233658	0.22905716	No
**204237_at**	GULP1	GULP, engulfment adaptor PTB domain containing 1	15070	-0.04178429767489433	0.2213506	No
**201882_x_at**	B4GALT1	UDP-Gal:betaGlcNAc beta 1,4-galactosyltransferase, polypeptide 1	15435	-0.04502340778708458	0.20531668	No
**217489_s_at**	IL6R	interleukin 6 receptor	15595	-0.046334486454725266	0.19867904	No
**207018_s_at**	RAB27B	RAB27B, member RAS oncogene family	15634	-0.046617500483989716	0.19758077	No
**208231_at**	NRG1	neuregulin 1	15721	-0.04740168899297714	0.19429737	No
**206343_s_at**	NRG1	neuregulin 1	15761	-0.0477057509124279	0.1931683	No
**204235_s_at**	GULP1	GULP, engulfment adaptor PTB domain containing 1	15834	-0.04839256778359413	0.19053897	No
**203640_at**	MBNL2	muscleblind-like 2 (Drosophila)	15870	-0.048664484173059464	0.18960606	No
**221282_x_at**	RUNX2	runt-related transcription factor 2	15884	-0.0488256961107254	0.1896818	No
**215331_at**	MYH15	myosin, heavy polypeptide 15	15964	-0.04957849159836769	0.18674853	No
**216632_at**	NAV3	Neuron navigator 3	16172	-0.05142241716384888	0.17798488	No
**205846_at**	PTPRB	protein tyrosine phosphatase, receptor type, B	16258	-0.05229029804468155	0.17481436	No
**218901_at**	PLSCR4	phospholipid scramblase 4	16589	-0.05541754141449928	0.16047858	No
**215459_at**	TAX1BP3	Tax1 (human T-cell leukemia virus type I) binding protein 3	16660	-0.056131668388843536	0.15804704	No
**215824_at**	NUDT7	nudix (nucleoside diphosphate linked moiety X)-type motif 7	16676	-0.05630377680063248	0.15813397	No
**208807_s_at**	CHD3	chromodomain helicase DNA binding protein 3	16976	-0.059405773878097534	0.14527114	No
**213807_x_at**	MET	met proto-oncogene (hepatocyte growth factor receptor)	17075	-0.060402762144804	0.1416173	No
**209615_s_at**	PAK1	p21/Cdc42/Rac1-activated kinase 1 (STE20 homolog, yeast)	17157	-0.06127350777387619	0.13875313	No
**205214_at**	STK17B	serine/threonine kinase 17b (apoptosis-inducing)	17354	-0.06349744647741318	0.1306585	No
**209135_at**	ASPH	aspartate beta-hydroxylase	17500	-0.06533317267894745	0.12492223	No
**212552_at**	HPCAL1	hippocalcin-like 1	17631	-0.0671054869890213	0.1198965	No

**215584_at**	HECW1	HECT, C2 and WW domain containing E3 ubiquitin protein ligase 1	17731	-0.0684022381901741	0.11630676	No
**207379_at**	EDIL3	EGF-like repeats and discoidin I-like domains 3	17840	-0.07020572572946548	0.11233006	No
**215915_at**	GULP1	GULP, engulfment adaptor PTB domain containing 1	17875	-0.07056725770235062	0.11174365	No
**218631_at**	AVPI1	arginine vasopressin-induced 1	17920	-0.07132795453071594	0.11071021	No
**210665_at**	TFPI	tissue factor pathway inhibitor (lipoprotein-associated coagulation inhibitor)	17968	-0.07191572338342667	0.1095476	No
**215221_at**	FOXP1	Forkhead box P1	18035	-0.07270743697881699	0.10752665	No
**214783_s_at**	ANXA11	annexin A11	18050	-0.0728620290756256	0.10788672	No
**211599_x_at**	MET	met proto-oncogene (hepatocyte growth factor receptor) /// met proto-oncogene (hepatocyte growth factor receptor)	18217	-0.07550634443759918	0.10132943	No
**201329_s_at**	ETS2	v-ets erythroblastosis virus E26 oncogene homolog 2 (avian)	18840	-0.08652237057685852	0.07406244	No
**219921_s_at**	DOCK5	dedicator of cytokinesis 5	19002	-0.08949630707502365	0.067926	No
**215206_at**	EXT1	Exostoses (multiple) 1	19148	-0.09272073209285736	0.062565796	No
**204686_at**	IRS1	insulin receptor substrate 1	19175	-0.09318052977323532	0.062655896	No
**208241_at**	NRG1	neuregulin 1	19259	-0.09519052505493164	0.06016597	No
**214888_at**	CAPN2	calpain 2, (m/II) large subunit	19273	-0.09567064046859741	0.060884982	No
**210311_at**	FGF5	fibroblast growth factor 5	19410	-0.09869006276130676	0.056018483	No
**209676_at**	TFPI	tissue factor pathway inhibitor (lipoprotein-associated coagulation inhibitor)	19428	-0.09902141988277435	0.05660052	No
**201117_s_at**	CPE	carboxypeptidase E	19570	-0.10240734368562698	0.051556323	No
**219682_s_at**	TBX3	T-box 3 (ulnar mammary syndrome)	19679	-0.10563391447067261	0.048066113	No
**205018_s_at**	MBNL2	muscleblind-like 2 (Drosophila)	19706	-0.106841541826725	0.0483438	No
**206767_at**	RBMS3	RNA binding motif, single stranded interacting protein	19910	-0.11370080709457397	0.040618327	No
**201328_at**	ETS2	v-ets erythroblastosis virus E26 oncogene homolog 2 (avian)	20041	-0.11817789822816849	0.036293916	No
**210896_s_at**	ASPH	aspartate beta-hydroxylase	20071	-0.11945558339357376	0.036607575	No
**205462_s_at**	HPCAL1	hippocalcin-like 1	20330	-0.1301066130399704	0.026591256	No
**212641_at**	HIVEP2	human immunodeficiency virus type I enhancer binding protein 2	20418	-0.13437095284461975	0.02445636	No
**205372_at**	PLAG1	pleiomorphic adenoma gene 1	20531	-0.14001375436782837	0.021255257	No
**204790_at**	SMAD7	SMAD, mothers against DPP homolog 7 (Drosophila)	20631	-0.14461910724639893	0.018712113	No
**217853_at**	TNS3	tensin 3	20665	-0.14655247330665588	0.019214869	No
**203837_at**	MAP3K5	mitogen-activated protein kinase kinase kinase 5	20805	-0.15429745614528656	0.014974716	No
**210331_at**	HECW1	HECT, C2 and WW domain containing E3 ubiquitin protein ligase 1	20834	-0.156039297580719	0.015836483	No
**218292_s_at**	PRKAG2	protein kinase, AMP-activated, gamma 2 non-catalytic subunit	20899	-0.16014905273914337	0.015107765	No
**212956_at**	TBC1D9	TBC1 domain family, member 9	21156	-0.1779506802558899	0.005839928	No
**208937_s_at**	ID1	inhibitor of DNA binding 1, dominant negative helix-loop-helix protein	21164	-0.178724005818367	0.007973899	No
**206275_s_at**	MICAL2	microtubule associated monoxygenase, calponin and LIM domain containing 2	21181	-0.1806897521018982	0.009723134	No
**203836_s_at**	MAP3K5	mitogen-activated protein kinase kinase kinase 5	21202	-0.18258097767829895	0.011315349	No
**208806_at**	CHD3	chromodomain helicase DNA binding protein 3	21256	-0.18705326318740845	0.011459299	No

**209684_at**	RIN2	Ras and Rab interactor 2	21261	-0.18751776218414307	0.013851269	No
**201116_s_at**	CPE	carboxypeptidase E	21353	-0.19601711630821228	0.012379896	No
**205856_at**	SLC14A1	solute carrier family 14 (urea transporter), member 1 (Kidd blood group)	21377	-0.19867843389511108	0.014055913	No
**209576_at**	GNAI1	guanine nucleotide binding protein (G protein), alpha inhibiting activity polypeptide 1	21535	-0.2170681655406952	0.009854258	No
**204454_at**	LDOC1	leucine zipper, down-regulated in cancer 1	21603	-0.22635743021965027	0.009897458	No
**204823_at**	NAV3	neuron navigator 3	21620	-0.22803175449371338	0.012296786	No
**216594_x_at**	AKR1C1	aldo-keto reductase family 1, member C1 (dihydrodiol dehydrogenase 1; 20-alpha (3-alpha)-hydroxysteroid dehydrogenase)	21882	-0.28260666131973267	0.004237327	No
**204151_x_at**	AKR1C1	aldo-keto reductase family 1, member C1 (dihydrodiol dehydrogenase 1; 20-alpha (3-alpha)-hydroxysteroid dehydrogenase)	21993	-0.31690549850463867	0.0035567638	No
**214098_at**	KIAA1107	KIAA1107	21998	-0.3180968761444092	0.0077418215	No

Supplementary List 3 - List of the 205 top ranking genes upregulated in ASCL1^{high}/NDRG1^{low} CSCs *vs*. GCL and selected based on an
Adjusted P value < 0.01 e log2 Fold Change > 1 for Gene Set Enrichment Analysis (GSEA) in the comparison PN *vs*. MES.

PROBE	GENE SYMBOL	GENE_TITLE	RANK IN GENE LIST	RANK METRIC SCORE	RUNNING ES	CORE ENRICHMENT
**219537_x_at**	DLL3	delta-like 3 (Drosophila)	13	0.6549095511436462	0.020421617	Yes
**209839_at**	DNM3	dynamin 3	15	0.6499274969100952	0.041226927	Yes
**209987_s_at**	ASCL1	achaete-scute complex-like 1 (Drosophila)	18	0.6169173717498779	0.060927935	Yes
**209988_s_at**	ASCL1	achaete-scute complex-like 1 (Drosophila)	23	0.6053429841995239	0.080167025	Yes
**213824_at**	OLIG2	oligodendrocyte lineage transcription factor 2	29	0.5745991468429565	0.09837452	Yes
**219196_at**	SCG3	secretogranin III	31	0.5723041892051697	0.11668957	Yes
**209815_at**	PTCH1	patched homolog 1 (Drosophila)	38	0.547568678855896	0.1339846	Yes
**213825_at**	OLIG2	oligodendrocyte lineage transcription factor 2	39	0.5462126731872559	0.15150788	Yes
**204851_s_at**	DCX	doublecortex; lissencephaly, X-linked (doublecortin)	43	0.5291727185249329	0.16834861	Yes
**205638_at**	BAI3	brain-specific angiogenesis inhibitor 3	53	0.5201330780982971	0.18462758	Yes
**204850_s_at**	DCX	doublecortex; lissencephaly, X-linked (doublecortin)	60	0.5122037529945374	0.20078805	Yes
**218829_s_at**	CHD7	chromodomain helicase DNA binding protein 7	66	0.5038008689880371	0.21672423	Yes
**205316_at**	SLC15A2	Solute carrier family 15 (H+/peptide transporter), member 2	148	0.39233365654945374	0.22564206	Yes
**213609_s_at**	SEZ6L	seizure related 6 homolog (mouse)-like	157	0.3846593499183655	0.23762013	Yes
**203849_s_at**	KIF1A	kinesin family member 1A	190	0.3620590567588806	0.24778609	Yes
**222301_at**	C1ORF61	Chromosome 1 open reading frame 61	240	0.33588707447052	0.2563424	Yes
**219415_at**	TTYH1	tweety homolog 1 (Drosophila)	242	0.33456602692604065	0.26703048	Yes
**210198_s_at**	PLP1	proteolipid protein 1 (Pelizaeus-Merzbacher disease, spastic paraplegia 2, uncomplicated)	285	0.31781232357025146	0.27532402	Yes
**220277_at**	CXXC4	CXXC finger 4	297	0.31427985429763794	0.28490832	Yes
**203264_s_at**	ARHGEF9	Cdc42 guanine nucleotide exchange factor (GEF) 9	302	0.31254884600639343	0.29475418	Yes
**209243_s_at**	PEG3	paternally expressed 3	311	0.3093435764312744	0.304316	Yes
**213768_s_at**	ASCL1	achaete-scute complex-like 1 (Drosophila)	318	0.3064843416213989	0.3138767	Yes
**221088_s_at**	PPP1R9A	protein phosphatase 1, regulatory (inhibitor) subunit 9A	328	0.30357518792152405	0.32320818	Yes

**213032_at**	NFIB	nuclear factor I/B	343	0.29910898208618164	0.33216992	Yes
**209460_at**	ABAT	4-aminobutyrate aminotransferase	365	0.29391294717788696	0.34064788	Yes
**209234_at**	KIF1B	kinesin family member 1B	372	0.29099565744400024	0.3497117	Yes
**214043_at**	PTPRD	Protein tyrosine phosphatase, receptor type, D	385	0.2865099310874939	0.3583598	Yes
**213362_at**	PTPRD	Protein tyrosine phosphatase, receptor type, D	398	0.28284069895744324	0.36689022	Yes
**205103_at**	C1ORF61	chromosome 1 open reading frame 61	403	0.28192245960235596	0.37575352	Yes
**206692_at**	KCNJ10	potassium inwardly-rectifying channel, subfamily J, member 10	409	0.2801637351512909	0.3845151	Yes
**205184_at**	GNG4	guanine nucleotide binding protein (G protein), gamma 4	478	0.2635939121246338	0.38989156	Yes
**209459_s_at**	ABAT	4-aminobutyrate aminotransferase	486	0.2625848352909088	0.3979986	Yes
**213033_s_at**	NFIB	nuclear factor I/B	516	0.2557018995285034	0.40488836	Yes
**205433_at**	BCHE	butyrylcholinesterase	558	0.246237114071846	0.41093096	Yes
**209985_s_at**	ASCL1	achaete-scute complex-like 1 (Drosophila)	622	0.23341403901576996	0.4155657	Yes
**205938_at**	PPM1E	protein phosphatase 1E (PP2C domain containing)	649	0.23015613853931427	0.4217718	Yes
**209504_s_at**	PLEKHB1	pleckstrin homology domain containing, family B (evectins) member 1	666	0.22786666452884674	0.4283574	Yes
**213029_at**	NFIB	nuclear factor I/B	676	0.2266375869512558	0.4352206	Yes
**211484_s_at**	DSCAM	Down syndrome cell adhesion molecule	681	0.22623206675052643	0.44229728	Yes
**205794_s_at**	NOVA1	neuro-oncological ventral antigen 1	684	0.22585149109363556	0.4494523	Yes
**211467_s_at**	NFIB	nuclear factor I/B	765	0.21442680060863495	0.45270792	Yes
**212482_at**	RMND5A	required for meiotic nuclear division 5 homolog A (S. cerevisiae)	771	0.21377712488174438	0.45933974	Yes
**204364_s_at**	REEP1	receptor accessory protein 1	787	0.21145495772361755	0.4654441	Yes
**213092_x_at**	DNAJC9	DnaJ (Hsp40) homolog, subfamily C, member 9	808	0.20860856771469116	0.4712307	Yes
**209242_at**	PEG3	paternally expressed 3	824	0.20672908425331116	0.47718343	Yes
**211685_s_at**	NCALD	neurocalcin delta /// neurocalcin delta	881	0.2008490264415741	0.4810905	Yes
**203853_s_at**	GAB2	GRB2-associated binding protein 2	915	0.19805459678173065	0.48594967	Yes
**213283_s_at**	SALL2	sal-like 2 (Drosophila)	947	0.19364716112613678	0.49075803	Yes
**205317_s_at**	SLC15A2	solute carrier family 15 (H+/peptide transporter), member 2	952	0.19335025548934937	0.49677983	Yes

**206731_at**	CNKSR2	connector enhancer of kinase suppressor of Ras 2	954	0.1932615786790847 8	0.50293463	Yes
**206527_at**	ABAT	4-aminobutyrate aminotransferase	1008	0.1879801750183105 5	0.50656474	Yes
**202548_s_at**	ARHGEF7	Rho guanine nucleotide exchange factor (GEF) 7	1022	0.1873734146356582 6	0.5119871	Yes
**207620_s_at**	CASK	calcium/calmodulin-dependent serine protein kinase (MAGUK family)	1034	0.1862008571624755 9	0.5174625	Yes
**207437_at**	NOVA1	neuro-oncological ventral antigen 1	1058	0.1846253871917724 6	0.52234375	Yes
**216047_x_at**	SEZ6L	seizure related 6 homolog (mouse)-like	1073	0.1830387413501739 5	0.5275818	Yes
**220892_s_at**	PSAT1	phosphoserine aminotransferase 1	1076	0.1827980577945709 2	0.5333556	Yes
**207873_x_at**	SEZ6L	seizure related 6 homolog (mouse)-like	1208	0.1712649911642074 6	0.53291655	Yes
**206083_at**	BAI1	brain-specific angiogenesis inhibitor 1	1211	0.1711251735687255 9	0.5383159	Yes
**202547_s_at**	ARHGEF7	Rho guanine nucleotide exchange factor (GEF) 7	1246	0.1686599850654602	0.54218674	Yes
**216456_at**	PCDH9	Protocadherin 9	1349	0.1616557538509369	0.5427529	Yes
**206465_at**	ACSBG1	acyl-CoA synthetase bubblegum family member 1	1361	0.1610462814569473 3	0.5474213	Yes
**214393_at**	RND2	Rho family GTPase 2	1386	0.1598913222551345 8	0.5514638	Yes
**204343_at**	ABCA3	ATP-binding cassette, sub-family A (ABC1), member 3	1388	0.1598347425460815 4	0.5565462	Yes
**204365_s_at**	REEP1	receptor accessory protein 1	1413	0.1582867056131363	0.5605372	Yes
**209290_s_at**	NFIB	nuclear factor I/B	1469	0.1547699868679046 6	0.5630113	Yes
**212935_at**	MCF2L	MCF.2 cell line derived transforming sequence-like	1488	0.1538587510585785	0.567132	Yes
**211466_at**	NFIB	nuclear factor I/B	1503	0.1529616266489029	0.5714051	Yes
**207693_at**	CACNB4	calcium channel, voltage-dependent, beta 4 subunit	1525	0.1520379036664962 8	0.5753315	Yes
**209469_at**	GPM6A	glycoprotein M6A	1566	0.1499587893486023	0.5783307	Yes
**211894_x_at**	SEZ6L	seizure related 6 homolog (mouse)-like /// seizure related 6 homolog (mouse)-like	1580	0.1490827798843383 8	0.58252466	Yes
**209816_at**	PTCH1	patched homolog 1 (Drosophila)	1612	0.1470888108015060 4	0.58583933	Yes
**220405_at**	SNTG1	syntrophin, gamma 1	1678	0.1437046080827713	0.58750546	Yes
**209289_at**	NFIB	nuclear factor I/B	1690	0.1432556957006454 5	0.5916031	Yes
**204029_at**	CELSR2	cadherin, EGF LAG seven-pass G-type receptor 2 (flamingo homolog, Drosophila)	1709	0.1423837840557098 4	0.5953557	Yes
**35147_at**	MCF2L	MCF.2 cell line derived transforming sequence-like	1712	0.1423191875219345	0.5998309	Yes
**215010_s_at**	BRSK2	BR serine/threonine kinase 2	1769	0.1393263936042785 6	0.6017642	Yes
**1438_at**	EPHB3	EPH receptor B3	1818	0.1368826329708099 4	0.6039815	Yes

**219726_at**	NLGN3	neuroligin 3	1867	0.13409411907196045	0.6061093	Yes
**205712_at**	PTPRD	protein tyrosine phosphatase, receptor type, D	2091	0.12416946142911911	0.59999233	Yes
**211208_s_at**	CASK	calcium/calmodulin-dependent serine protein kinase (MAGUK family)	2186	0.12030167877674103	0.5995941	Yes
**208070_s_at**	REV3L	REV3-like, catalytic subunit of DNA polymerase zeta (yeast)	2239	0.11828062683343887	0.60103345	Yes
**204966_at**	BAI2	brain-specific angiogenesis inhibitor 2	2243	0.11813624948263168	0.6046876	Yes
**203864_s_at**	ACTN2	actinin, alpha 2	2249	0.11790721118450165	0.6082437	Yes
**207112_s_at**	GAB1	GRB2-associated binding protein 1	2364	0.11403854191303253	0.60673875	Yes
**209470_s_at**	GPM6A	glycoprotein M6A	2398	0.11271186172962189	0.60886	Yes
**220576_at**	PGAP1	GPI deacylase	2443	0.11089474707841873	0.6104247	Yes
**213467_at**	RND2	Rho family GTPase 2	2475	0.11010928452014923	0.61255306	Yes
**212479_s_at**	RMND5A	required for meiotic nuclear division 5 homolog A (S. cerevisiae)	2575	0.10685732960700989	0.6114971	No
**207055_at**	GPR37L1	G protein-coupled receptor 37 like 1	2850	0.09912077337503433	0.6022665	No
**214543_x_at**	QKI	quaking homolog, KH domain RNA binding (mouse)	2904	0.09793934971094131	0.603008	No
**214541_s_at**	QKI	quaking homolog, KH domain RNA binding (mouse)	2963	0.09676247835159302	0.60348517	No
**201235_s_at**	BTG2	BTG family, member 2	3071	0.09396553039550781	0.6016533	No
**202778_s_at**	ZMYM2	zinc finger, MYM-type 2	3220	0.090818390250206	0.5978634	No
**219829_at**	ITGB1BP2	integrin beta 1 binding protein (melusin) 2	3252	0.09017063677310944	0.59935206	No
**210281_s_at**	ZMYM2	zinc finger, MYM-type 2	3492	0.08575667440891266	0.59127796	No
**34697_at**	LRP6	low density lipoprotein receptor-related protein 6	3791	0.08029322326183319	0.5803563	No
**205606_at**	LRP6	low density lipoprotein receptor-related protein 6	3800	0.08022619783878326	0.58256775	No
**214220_s_at**	ALMS1	Alstrom syndrome 1	3844	0.07944794744253159	0.58316886	No
**219255_x_at**	IL17RB	interleukin 17 receptor B	3845	0.07943178713321686	0.5857172	No
**219738_s_at**	PCDH9	protocadherin 9	3877	0.0788387805223465	0.5868423	No
**219737_s_at**	PCDH9	protocadherin 9	3930	0.07771836221218109	0.58698034	No
**215962_at**	SNTG1	Syntrophin, gamma 1	4121	0.07471383363008499	0.58077145	No
**214817_at**	UNC13A	unc-13 homolog A (C. elegans)	4151	0.07433709502220154	0.5818427	No

**208522_s_at**	PTCH1	patched homolog 1 (Drosophila)	4169	0.07409704476594925	0.5834499	No
**220619_at**	CHD7	chromodomain helicase DNA binding protein 7	4226	0.07292832434177399	0.5832531	No
**216113_at**	ABI2	Abl interactor 2	4228	0.07291719317436218	0.5855471	No
**204600_at**	EPHB3	EPH receptor B3	4325	0.07174788415431976	0.5835006	No
**208920_at**	SRI	sorcin	4469	0.06982914358377457	0.5792638	No
**208564_at**	KCNA2	potassium voltage-gated channel, shaker-related subfamily, member 2	4526	0.06893665343523026	0.5789389	No
**206466_at**	ACSBG1	acyl-CoA synthetase bubblegum family member 1	4571	0.06807364523410797	0.5791299	No
**210282_at**	ZMYM2	zinc finger, MYM-type 2	4800	0.06516125053167343	0.57089335	No
**216487_at**	ITPR2	Inositol 1,4,5-triphosphate receptor, type 2	4807	0.06510455161333084	0.5727102	No
**202661_at**	ITPR2	inositol 1,4,5-triphosphate receptor, type 2	4861	0.06432726234197617	0.57237333	No
**206826_at**	PMP2	peripheral myelin protein 2	4913	0.06371486186981201	0.57210743	No
**209856_x_at**	ABI2	abl interactor 2	5019	0.06252259761095047	0.5693574	No
**221615_at**	BMP8B	bone morphogenetic protein 8b (osteogenic protein 2)	5163	0.060924120247364044	0.5648349	No
**207696_at**	FUT9	fucosyltransferase 9 (alpha (1,3) fucosyltransferase)	5217	0.0603003166615963	0.5643688	No
**203220_s_at**	TLE1	transducin-like enhancer of split 1 (E(sp1) homolog, Drosophila)	5258	0.059860825538635254	0.5644775	No
**212636_at**	QKI	quaking homolog, KH domain RNA binding (mouse)	5281	0.05966051667928696	0.565395	No
**203940_s_at**	VASH1	vasohibin 1	5525	0.05703815072774887	0.55621845	No
**213469_at**	PGAP1	GPI deacylase	5546	0.05684385448694229	0.5571362	No
**203861_s_at**	ACTN2	actinin, alpha 2	5642	0.055955223739147186	0.5546284	No
**220464_at**	MCF2L	MCF.2 cell line derived transforming sequence-like	5697	0.05535415932536125	0.55395836	No
**36499_at**	CELSR2	cadherin, EGF LAG seven-pass G-type receptor 2 (flamingo homolog, Drosophila)	5722	0.05507583171129227	0.5546382	No
**213088_s_at**	DNAJC9	DnaJ (Hsp40) homolog, subfamily C, member 9	5836	0.05405291169881821	0.5512541	No
**203263_s_at**	ARHGEF9	Cdc42 guanine nucleotide exchange factor (GEF) 9	6026	0.05228346586227417	0.5443709	No
**216185_at**	FUT9	Fucosyltransferase 9 (alpha (1,3) fucosyltransferase)	6202	0.050705332309007645	0.5380711	No
**212263_at**	QKI	quaking homolog, KH domain RNA binding (mouse)	6393	0.04886188730597496	0.5310328	No

**201236_s_at**	BTG2	BTG family, member 2	6405	0.04873793199658394	0.5320982	No
**215802_at**	TLE1	Transducin-like enhancer of split 1 (E(sp1) homolog, Drosophila)	6469	0.048217013478279114	0.5307915	No
**215006_at**	EZH2	Enhancer of zeste homolog 2 (Drosophila)	6710	0.04613575339317322	0.5214011	No
**203850_s_at**	KIF1A	kinesin family member 1A	6967	0.043913304805755615	0.5112146	No
**212478_at**	RMND5A	required for meiotic nuclear division 5 homolog A (S. cerevisiae)	7132	0.04254785925149917 6	0.5051514	No
**211793_s_at**	ABI2	abl interactor 2	7272	0.04138776659965515	0.50018334	No
**203702_s_at**	TTLL4	tubulin tyrosine ligase-like family, member 4	7349	0.04078348353505134 6	0.49804938	No
**216104_at**	PHF14	PHD finger protein 14	7370	0.04060140252113342	0.49844605	No
**211568_at**	BAI3	brain-specific angiogenesis inhibitor 3	7381	0.04055162146687507 6	0.49929407	No
**215964_at**	SNTG1	Syntrophin, gamma 1	7382	0.04055127874016762	0.50059503	No
**204835_at**	POLA1	polymerase (DNA directed), alpha 1	7560	0.03934360295534134	0.4938402	No
**206202_at**	MEOX2	mesenchyme homeobox 2	8595	0.03177214786410332	0.44802552	No
**204906_at**	RPS6KA2	ribosomal protein S6 kinase, 90kDa, polypeptide 2	8629	0.031546883285045624	0.4475429	No
**216097_at**	PHF14	PHD finger protein 14	9253	0.027227625250816345	0.42019826	No
**203862_s_at**	ACTN2	actinin, alpha 2	9324	0.026713835075497627	0.4178847	No
**212912_at**	RPS6KA2	ribosomal protein S6 kinase, 90kDa, polypeptide 2	9329	0.026698358356952667	0.41856006	No
**214227_at**	GNG7	Guanine nucleotide binding protein (G protein), gamma 7	9736	0.024105172604322433	0.40094402	No
**202191_s_at**	GAS7	growth arrest-specific 7	10067	0.021688371896743774	0.38669282	No
**202192_s_at**	GAS7	growth arrest-specific 7	10217	0.020719347521662712	0.38060874	No
**207268_x_at**	ABI2	abl interactor 2	10286	0.020247986540198326	0.37817833	No
**214707_x_at**	ALMS1	Alstrom syndrome 1	10421	0.019371218979358673	0.3727304	No
**216614_at**	ITPR2	Inositol 1,4,5-triphosphate receptor, type 2	10927	0.01603442057967186	0.35037136	No
**216707_at**	PCDH9	Protocadherin 9	11656	0.011224310845136642	0.31775743	No
**207865_s_at**	BMP8B	bone morphogenetic protein 8b (osteogenic protein 2)	11767	0.010413113981485367	0.31310916	No
**212265_at**	QKI	quaking homolog, KH domain RNA binding (mouse)	11880	0.009785131551325321	0.30835018	No

**204311_at**	ATP1B2	ATPase, Na+/K+ transporting, beta 2 polypeptide	11994	0.009023511782288551	0.30352145	No
**205738_s_at**	FABP3	fatty acid binding protein 3, muscle and heart (mammary-derived growth inhibitor)	12009	0.008916682563722134	0.3031734	No
**214221_at**	ALMS1	Alstrom syndrome 1	12039	0.0087151918560266 5	0.30213946	No
**207454_at**	GRIK3	glutamate receptor, ionotropic, kainate 3	12185	0.007747315336018801	0.2958204	No
**204469_at**	PTPRZ1	protein tyrosine phosphatase, receptor-type, Z polypeptide 1	12424	0.006091001443564892	0.28523582	No
**203185_at**	RASSF2	Ras association (RalGDS/AF-6) domain family 2	12721	0.003979467321187258	0.27195647	No
**207792_at**	OPRD1	opioid receptor, delta 1	12991	0.002000864129513502	0.25983658	No
**203358_s_at**	EZH2	enhancer of zeste homolog 2 (Drosophila)	13026	0.0017549480544403195	0.2583529	No
**211360_s_at**	ITPR2	inositol 1,4,5-triphosphate receptor, type 2	13130	9,51E+11	0.25371814	No
**213050_at**	COBL	cordon-bleu homolog (mouse)	13272	-1,15E+12	0.24733539	No
**210872_x_at**	GAS7	growth arrest-specific 7	13916	-0.005442379973828793	0.21838596	No
**220354_at**	MCF2L	MCF.2 cell line derived transforming sequence-like	14144	-0.007303472142666578	0.20833854	No
**204836_at**	GLDC	glycine dehydrogenase (decarboxylating)	14223	-0.008015912026166916	0.20506278	No
**219699_at**	LGI2	leucine-rich repeat LGI family, member 2	14768	-0.0129999937489628 8	0.18083993	No
**203222_s_at**	TLE1	transducin-like enhancer of split 1 (E(sp1) homolog, Drosophila)	15486	-0.020446887239813805	0.14902012	No
**203221_at**	TLE1	transducin-like enhancer of split 1 (E(sp1) homolog, Drosophila)	15664	-0.0226969365030527 1	0.14173125	No
**205363_at**	BBOX1	butyrobetaine (gamma), 2-oxoglutarate dioxygenase (gamma-butyrobetaine hydroxylase) 1	15718	-0.023401379585266113	0.14008142	No
**202662_s_at**	ITPR2	inositol 1,4,5-triphosphate receptor, type 2	15825	-0.0247451066970825 2	0.13607411	No
**205590_at**	RASGRP1	RAS guanyl releasing protein 1 (calcium and DAG-regulated)	15846	-0.0249671060591936 1	0.13596922	No
**208921_s_at**	SRI	sorcin	16017	-0.0272310320287942 9	0.12914285	No

**214538_x_at**	RGS6	regulator of G-protein signalling 6	16054	-0.02772645093500614	0.12840177	No
**204525_at**	PHF14	PHD finger protein 14	16115	-0.028639474883675575	0.12660293	No
**212262_at**	QKI	quaking homolog, KH domain RNA binding (mouse)	16123	-0.028757309541106224	0.12720846	No
**207704_s_at**	GAS7	growth arrest-specific 7	17150	-0.04325054958462715	0.08212439	No
**213395_at**	MLC1	megalencephalic leukoencephalopathy with subcortical cysts 1	17427	-0.0479620099067688	0.07116195	No
**204391_x_at**	TRIM24	tripartite motif-containing 24	17614	-0.05157945677638054	0.064392015	No
**214285_at**	FABP3	fatty acid binding protein 3, muscle and heart (mammary-derived growth inhibitor)	18080	-0.06072692945599556	0.045278534	No
**211067_s_at**	GAS7	growth arrest-specific 7 /// growth arrest-specific 7	18429	-0.06924574822187424	0.031737737	No
**210270_at**	RGS6	regulator of G-protein signalling 6	18498	-0.07109648734331131	0.030938627	No
**201726_at**	ELAVL1	ELAV (embryonic lethal, abnormal vision, Drosophila)-like 1 (Hu antigen R)	18597	-0.07356361299753189	0.028859844	No
**211448_s_at**	RGS6	regulator of G-protein signalling 6	18766	-0.0785321444272995	0.023769883	No
**216733_s_at**	GATM	glycine amidinotransferase (L-arginine:glycine amidinotransferase)	18909	-0.08268314599990845	0.019990737	No
**206896_s_at**	GNG7	guanine nucleotide binding protein (G protein), gamma 7	19041	-0.08636459708213806	0.016827928	No
**206136_at**	FZD5	frizzled homolog 5 (Drosophila)	19085	-0.08758776634931564	0.017690228	No
**219797_at**	MGAT4A	mannosyl (alpha-1,3-)-glycoprotein beta-1,4-N-acetylglucosaminyltransferase, isozyme A	19134	-0.08862249553203583	0.018359255	No
**213301_x_at**	TRIM24	tripartite motif-containing 24	19187	-0.09014267474412918	0.018895876	No
**201727_s_at**	ELAVL1	ELAV (embryonic lethal, abnormal vision, Drosophila)-like 1 (Hu antigen R)	19369	-0.09619534015655518	0.013783755	No

**220029_at**	ELOVL2	elongation of very long chain fatty acids (FEN1/Elo2, SUR4/Elo3, yeast)-like 2	19749	-0.11160242557525635	1,98E+02	No
**202660_at**	ITPR2	Inositol 1,4,5-triphosphate receptor, type 2	20086	-0.12605266273021698	-0.010977108	No
**209474_s_at**	ENTPD1	ectonucleoside triphosphate diphosphohydrolase 1	20102	-0.1267687976360321	-0.007589593	No
**207691_x_at**	ENTPD1	ectonucleoside triphosphate diphosphohydrolase 1	20115	-0.12747140228748322	-0.0040436555	No
**219683_at**	FZD3	frizzled homolog 3 (Drosophila)	20237	-0.13350097835063934	-0.005241321	No
**203178_at**	GATM	glycine amidinotransferase (L-arginine:glycine amidinotransferase)	20435	-0.14344793558120728	-0.009562215	No
**216331_at**	ITGA7	integrin, alpha 7	21318	-0.22741307318210602	-0.042215746	No
**206306_at**	RYR3	ryanodine receptor 3	21335	-0.2305852770805359	-0.035542943	No
**219331_s_at**	KLHDC8A	kelch domain containing 8A	21554	-0.26660335063934326	-0.03686401	No
**219011_at**	PLEKHA4	pleckstrin homology domain containing, family A (phosphoinositide binding specific) member 4	21614	-0.2813994288444519	-0.030508656	No
**209663_s_at**	ITGA7	integrin, alpha 7	21692	-0.29841142892837524	-0.024422824	No
**213712_at**	ELOVL2	elongation of very long chain fatty acids (FEN1/Elo2, SUR4/Elo3, yeast)-like 2	21760	-0.31955718994140625	-0.017205667	No
**214913_at**	ADAMTS3	ADAM metallopeptidase with thrombospondin type 1 motif, 3	21914	-0.36738651990890503	-0.012349356	No
**220543_at**	C21ORF62	chromosome 21 open reading frame 62	21991	-0.4067578911781311	-0.0027423203	No
**206201_s_at**	MEOX2	mesenchyme homeobox 2	22134	-0.4949165880680084	0.0067035723	No

Supplementary List 4 - List of the 470 top ranking genes upregulated in ASCL1^{high}/NDRG1^{high} CSCs *vs.* GCL and selected based on an Adjusted P value < 0.001 e log2 Fold Change > 1 for Gene Set Enrichment Analysis (GSEA) in the comparison PN *vs.* MES.

Genes upregulated in ASCL1^{high}/NDRG1^{high} CSCs but positively enriched in the MES subgroup (*i.e.* those with a negative running ES) are highlighted in red.

PROBE	GENE SYMBOL	GENE TITLE	RANK IN GENE LIST	RANK METRIC SCORE	RUNNING ES	CORE ENRICHMENT
**209839_at**	DNM3	dynamin 3	15	0.6499274969100952	0.010114332	Yes
**209987_s_at**	ASCL1	achaete-scute complex-like 1 (Drosophila)	18	0.6169173717498779	0.020276	Yes
**221623_at**	BCAN	brevican	21	0.6073176860809326	0.030278118	Yes
**209988_s_at**	ASCL1	achaete-scute complex-like 1 (Drosophila)	23	0.6053429841995239	0.04029326	Yes
**213824_at**	OLIG2	oligodendrocyte lineage transcription factor 2	29	0.5745991468429565	0.049614057	Yes
**219196_at**	SCG3	secretogranin III	31	0.5723041892051697	0.059080083	Yes
**209815_at**	PTCH1	patched homolog 1 (Drosophila)	38	0.547568678855896	0.067905776	Yes
**213825_at**	OLIG2	oligodendrocyte lineage transcription factor 2	39	0.5462126731872559	0.076984	Yes
**205638_at**	BAI3	brain-specific angiogenesis inhibitor 3	53	0.5201330780982971	0.0850328	Yes
**203485_at**	RTN1	reticulon 1	56	0.5195413827896118	0.09357605	Yes
**218829_s_at**	CHD7	chromodomain helicase DNA binding protein 7	66	0.5038008689880371	0.10153678	Yes
**209757_s_at**	MYCN	v-myc myelocytomatosis viral related oncogene, neuroblastoma derived (avian)	68	0.5012710094451904	0.10982221	Yes
**210222_s_at**	RTN1	reticulon 1	70	0.4944189488887787	0.117993765	Yes
**219107_at**	BCAN	brevican	74	0.48157739639282227	0.1258602	Yes
**203608_at**	ALDH5A 1	aldehyde dehydrogenase 5 family, member A1 (succinate-semialdehyde dehydrogenase)	112	0.4274754822254181	0.13126874	Yes
**205316_at**	SLC15A2	Solute carrier family 15 (H+/peptide transporter), member 2	148	0.39233365654945374	0.13618489	Yes
**210015_s_at**	MAP2	microtubule-associated protein 2	150	0.389330118894577	0.14260983	Yes
**213609_s_at**	SEZ6L	seizure related 6 homolog (mouse)-like	157	0.3846593499183655	0.14872792	Yes
**91920_at**	BCAN	brevican	166	0.3796325623989105	0.15467079	Yes
**203849_s_at**	KIF1A	kinesin family member 1A	190	0.3620590567588806	0.1596339	Yes
**206408_at**	LRRTM2	leucine rich repeat transmembrane neuronal 2	208	0.3538261651992798	0.16473524	Yes
**205152_at**	SLC6A1	solute carrier family 6 (neurotransmitter transporter, GABA), member 1	233	0.3395020663738251	0.16927761	Yes
**222301_at**	C1ORF61	Chromosome 1 open reading frame 61	240	0.33588707447052	0.1745851	Yes
**219415_at**	TTYH1	tweety homolog 1 (Drosophila)	242	0.33456602692604065	0.18009984	Yes

**202454_s_at**	ERBB3	v-erb-b2 erythroblastic leukemia viral oncogene homolog 3 (avian)	275	0.3210487365722656	0.18396875	Yes
**209867_s_at**	LPHN3	latrophilin 3	280	0.31896060705184937	0.1890866	Yes
**220116_at**	KCNN2	potassium intermediate/small conductance calcium-activated channel, subfamily N, member 2	282	0.31813845038414	0.19432832	Yes
**210198_s_at**	PLP1	proteolipid protein 1 (Pelizaeus-Merzbacher disease, spastic paraplegia 2, uncomplicated)	285	0.31781232357025146	0.19951877	Yes
**203264_s_at**	ARHGEF 9	Cdc42 guanine nucleotide exchange factor (GEF) 9	302	0.31254884600639343	0.20397992	Yes
**209618_at**	CTNND2	catenin (cadherin-associated protein), delta 2 (neural plakophilin-related arm-repeat protein)	304	0.31179970502853394	0.20911628	Yes
**209243_s_at**	PEG3	paternally expressed 3	311	0.3093435764312744	0.2139826	Yes
**206915_at**	NKX2-2	NK2 transcription factor related, locus 2 (Drosophila)	313	0.30857038497924805	0.2190653	Yes
**213768_s_at**	ASCL1	achaete-scute complex-like 1 (Drosophila)	318	0.3064843416213989	0.22397578	Yes
**221088_s_at**	PPP1R9A	protein phosphatase 1, regulatory (inhibitor) subunit 9A	328	0.30357518792152405	0.2286087	Yes
**219743_at**	HEY2	hairy/enhancer-of-split related with YRPW motif 2	333	0.3015672266483307	0.23343746	Yes
**213032_at**	NFIB	nuclear factor I/B	343	0.29910898208618164	0.23799615	Yes
**209460_at**	ABAT	4-aminobutyrate aminotransferase	365	0.29391294717788696	0.24191834	Yes
**209234_at**	KIF1B	kinesin family member 1B	372	0.29099565744400024	0.24647972	Yes
**214043_at**	PTPRD	Protein tyrosine phosphatase, receptor type, D	385	0.2865099310874939	0.25069147	Yes
**215807_s_at**	PLXNB1	plexin B1	390	0.2852330803871155	0.25524876	Yes
**213362_at**	PTPRD	Protein tyrosine phosphatase, receptor type, D	398	0.28284069895744324	0.25962874	Yes
**205103_at**	C1ORF61	chromosome 1 open reading frame 61	403	0.28192245960235596	0.264131	Yes
**206692_at**	KCNJ10	potassium inwardly-rectifying channel, subfamily J, member 10	409	0.2801637351512909	0.26855817	Yes
**219564_at**	KCNJ16	potassium inwardly-rectifying channel, subfamily J, member 16	439	0.27215638756752014	0.27175203	Yes
**201525_at**	APOD	apolipoprotein D	450	0.26947832107543945	0.2757724	Yes
**205413_at**	MPPED2	metallophosphoesterase domain containing 2	456	0.2674659192562103	0.27998856	Yes
**214680_at**	NTRK2	neurotrophic tyrosine kinase, receptor, type 2	460	0.26689600944519043	0.28428692	Yes
**209459_s_at**	ABAT	4-aminobutyrate aminotransferase	486	0.2625848352909088	0.28750503	Yes
**213033_s_at**	NFIB	nuclear factor I/B	516	0.2557018995285034	0.2904254	Yes
**209558_s_at**	HIP1R	huntingtin interacting protein 1 related	526	0.2531532645225525	0.2942203	Yes
**213996_at**	YPEL1	yippee-like 1 (Drosophila)	534	0.2510828375816345	0.29807246	Yes
**209866_s_at**	LPHN3	latrophilin 3	537	0.2507722079753876	0.30214867	Yes

**213228_at**	PDE8B	phosphodiesterase 8B	577	0.24149632453918457	0.3043745	Yes
**209985_s_at**	ASCL1	achaete-scute complex-like 1 (Drosophila)	622	0.23341403901576996	0.30623677	Yes
**205938_at**	PPM1E	protein phosphatase 1E (PP2C domain containing)	649	0.23015613853931427	0.30887008	Yes
**209504_s_at**	PLEKHB1	pleckstrin homology domain containing, family B (evectins) member 1	666	0.22786666452884674	0.3119238	Yes
**38340_at**	HIP1R	huntingtin interacting protein 1 related	671	0.22760829329490662	0.31552333	Yes
**213029_at**	NFIB	nuclear factor I/B	676	0.2266375869512558	0.31910673	Yes
**211484_s_at**	DSCAM	Down syndrome cell adhesion molecule	681	0.22623206675052643	0.32268342	Yes
**205794_s_at**	NOVA1	neuro-oncological ventral antigen 1	684	0.22585149109363556	0.32634544	Yes
**209617_s_at**	CTNND2	catenin (cadherin-associated protein), delta 2 (neural plakophilin-related arm-repeat protein)	690	0.22536858916282654	0.3298619	Yes
**221796_at**	NTRK2	neurotrophic tyrosine kinase, receptor, type 2	764	0.21460260450839996	0.33008206	Yes
**211467_s_at**	NFIB	nuclear factor I/B	765	0.21442680060863495	0.33364588	Yes
**219521_at**	B3GAT1	beta-1,3-glucuronyltransferase 1 (glucuronosyltransferase P)	788	0.21088744699954987	0.33614233	Yes
**213578_at**	BMPR1A	bone morphogenetic protein receptor, type IA	790	0.21050889790058136	0.3395952	Yes
**213197_at**	ASTN1	astrotactin 1	819	0.20723164081573486	0.34175584	Yes
**209242_at**	PEG3	paternally expressed 3	824	0.20672908425331116	0.34500834	Yes
**215028_at**	SEMA6A	sema domain, transmembrane domain (TM), and cytoplasmic domain, (semaphorin) 6A	829	0.20583657920360565	0.34824604	Yes
**211494_s_at**	SLC4A4	solute carrier family 4, sodium bicarbonate cotransporter, member 4	875	0.2013636827468872	0.34952977	Yes
**211685_s_at**	NCALD	neurocalcin delta /// neurocalcin delta	881	0.2008490264415741	0.35263872	Yes
**203609_s_at**	ALDH5A1	aldehyde dehydrogenase 5 family, member A1 (succinate-semialdehyde dehydrogenase)	909	0.1983320266008377	0.3546973	Yes
**203853_s_at**	GAB2	GRB2-associated binding protein 2	915	0.19805459678173065	0.35775977	Yes
**203908_at**	SLC4A4	solute carrier family 4, sodium bicarbonate cotransporter, member 4	931	0.19577796757221222	0.36032602	Yes
**213283_s_at**	SALL2	sal-like 2 (Drosophila)	947	0.19364716112613678	0.36285684	Yes
**205317_s_at**	SLC15A2	solute carrier family 15 (H+/peptide transporter), member 2	952	0.19335025548934937	0.365887	Yes
**206731_at**	CNKSR2	connector enhancer of kinase suppressor of Ras 2	954	0.19326157867908478	0.3690532	Yes
**203029_s_at**	PTPRN2	protein tyrosine phosphatase, receptor type, N polypeptide 2	960	0.19263990223407745	0.37202573	Yes
**206527_at**	ABAT	4-aminobutyrate aminotransferase	1008	0.18798017501831055	0.37299535	Yes

**206462_s_at**	NTRK3	neurotrophic tyrosine kinase, receptor, type 3	1015	0.1877857893705368	0.37584132	Yes
**207437_at**	NOVA1	neuro-oncological ventral antigen 1	1058	0.18462538719177246	0.3769844	Yes
**216047_x_at**	SEZ6L	seizure related 6 homolog (mouse)-like	1073	0.18303874135017395	0.37938473	Yes
**202561_at**	TNKS	tankyrase, TRF1-interacting ankyrin-related ADP-ribose polymerase	1161	0.1751076728105545	0.37830663	Yes
**221045_s_at**	PER3	period homolog 3 (Drosophila)	1189	0.17253828048706055	0.3799365	Yes
**216933_x_at**	APC	adenomatosis polyposis coli	1193	0.1723545491695404	0.38266355	Yes
**203526_s_at**	APC	adenomatosis polyposis coli	1201	0.17193534970283508	0.38520023	Yes
**207151_at**	ADCYAP 1R1	adenylate cyclase activating polypeptide 1 (pituitary) receptor type I	1204	0.1718164086341858	0.3879642	Yes
**207873_x_at**	SEZ6L	seizure related 6 homolog (mouse)-like	1208	0.17126499116420746	0.39067313	Yes
**206083_at**	BAI1	brain-specific angiogenesis inhibitor 1	1211	0.17112517356872559	0.3934256	Yes
**208552_at**	GRIK4	glutamate receptor, ionotropic, kainate 4	1240	0.16913606226444244	0.39495307	Yes
**210383_at**	SCN1A	sodium channel, voltage-gated, type I, alpha	1248	0.1684713065624237	0.3974322	Yes
**211899_s_at**	TRAF4	TNF receptor-associated factor 4	1262	0.1675788015127182	0.39962143	Yes
**202986_at**	ARNT2	aryl-hydrocarbon receptor nuclear translocator 2	1288	0.16556619107723236	0.4012271	Yes
**209583_s_at**	CD200	CD200 molecule	1295	0.16524042189121246	0.40369835	Yes
**205613_at**	SYT17	synaptotagmin XVII	1338	0.16293801367282867	0.404481	Yes
**205593_s_at**	PDE9A	phosphodiesterase 9A	1348	0.1617581844329834	0.40675688	Yes
**216456_at**	PCDH9	Protocadherin 9	1349	0.1616557538509369	0.40944365	Yes
**214393_at**	RND2	Rho family GTPase 2	1386	0.15989132225513458	0.4104507	Yes
**204343_at**	ABCA3	ATP-binding cassette, sub-family A (ABC1), member 3	1388	0.15983474254608154	0.41306135	Yes
**221795_at**	NTRK2	neurotrophic tyrosine kinase, receptor, type 2	1390	0.1595579981803894	0.4156674	Yes
**209582_s_at**	CD200	CD200 molecule	1419	0.15787556767463684	0.4170077	Yes
**205062_x_at**	ARID4A	AT rich interactive domain 4A (RBP1-like)	1458	0.15532812476158142	0.41784722	Yes
**209290_s_at**	NFIB	nuclear factor I/B	1469	0.15476998686790466	0.4199611	Yes
**209763_at**	CHRDL1	chordin-like 1	1473	0.15464192628860474	0.42239377	Yes
**212935_at**	MCF2L	MCF.2 cell line derived transforming sequence-like	1488	0.1538587510585785	0.42430913	Yes
**211466_at**	NFIB	nuclear factor I/B	1503	0.1529616266489029	0.42620957	Yes
**202871_at**	TRAF4	TNF receptor-associated factor 4	1517	0.15239082276821136	0.4281464	Yes
**207693_at**	CACNB4	calcium channel, voltage-dependent, beta 4 subunit	1525	0.15203790366649628	0.4303524	Yes
**209469_at**	GPM6A	glycoprotein M6A	1566	0.1499587893486023	0.431011	Yes
**211894_x_at**	SEZ6L	seizure related 6 homolog (mouse)-like /// seizure related 6 homolog (mouse)-like	1580	0.14908277988433838	0.43289283	Yes
**202946_s_at**	BTBD3	BTB (POZ) domain containing 3	1609	0.14737869799137115	0.43405867	Yes

**209816_at**	PTCH1	patched homolog 1 (Drosophila)	1612	0.14708881080150604	0.43641162	Yes
**214070_s_at**	ATP10B	ATPase, Class V, type 10B	1618	0.1464974582195282	0.43861723	Yes
**215115_x_at**	NTRK3	neurotrophic tyrosine kinase, receptor, type 3	1630	0.14575041830539703	0.44053537	Yes
**219250_s_at**	FLRT3	fibronectin leucine rich transmembrane protein 3	1641	0.14526422321796417	0.44249126	Yes
**210739_x_at**	SLC4A4	solute carrier family 4, sodium bicarbonate cotransporter, member 4	1652	0.14460158348083496	0.44443616	Yes
**207152_at**	NTRK2	neurotrophic tyrosine kinase, receptor, type 2	1673	0.14391720294952393	0.4459112	Yes
**220405_at**	SNTG1	syntrophin, gamma 1	1678	0.1437046080827713	0.44811624	Yes
**209289_at**	NFIB	nuclear factor I/B	1690	0.14325569570064545	0.44999292	Yes
**203527_s_at**	APC	adenomatosis polyposis coli	1707	0.142525777220726	0.45162824	Yes
**204029_at**	CELSR2	cadherin, EGF LAG seven-pass G-type receptor 2 (flamingo homolog, Drosophila)	1709	0.14238378405570984	0.45394886	Yes
**35147_at**	MCF2L	MCF.2 cell line derived transforming sequence-like	1712	0.1423191875219345	0.45622256	Yes
**217377_x_at**	NTRK3	neurotrophic tyrosine kinase, receptor, type 3	1723	0.14192919433116913	0.45812303	Yes
**203525_s_at**	APC	adenomatosis polyposis coli	1731	0.1413954496383667	0.46015215	Yes
**1438_at**	EPHB3	EPH receptor B3	1818	0.13688263297080994	0.45848456	Yes
**219726_at**	NLGN3	neuroligin 3	1867	0.13409411907196045	0.45851272	Yes
**200979_at**	MAP3K15	Mitogen-activated protein kinase kinase kinase 15	2083	0.12464014440774918	0.4507278	Yes
**205712_at**	PTPRD	protein tyrosine phosphatase, receptor type, D	2091	0.12416946142911911	0.4524706	Yes
**217033_x_at**	NTRK3	neurotrophic tyrosine kinase, receptor, type 3	2100	0.12378866970539093	0.45416126	Yes
**215692_s_at**	MPPED2	metallophosphoesterase domain containing 2	2145	0.12197543680667877	0.4541714	Yes
**214168_s_at**	TJP1	tight junction protein 1 (zona occludens 1)	2158	0.1214049831032753	0.45563903	Yes
**204519_s_at**	PLLP	plasma membrane proteolipid (plasmolipin)	2235	0.11842784285545349	0.4541232	Yes
**208070_s_at**	REV3L	REV3-like, catalytic subunit of DNA polymerase zeta (yeast)	2239	0.11828062683343887	0.4559515	Yes
**204966_at**	BAI2	brain-specific angiogenesis inhibitor 2	2243	0.11813624948263168	0.45777744	Yes
**203864_s_at**	ACTN2	actinin, alpha 2	2249	0.11790721118450165	0.45950788	Yes
**209559_at**	HIP1R	huntingtin interacting protein 1 related	2345	0.11467336118221283	0.45705858	Yes
**207112_s_at**	GAB1	GRB2-associated binding protein 1	2364	0.11403854191303253	0.45812875	Yes
**209470_s_at**	GPM6A	glycoprotein M6A	2398	0.11271186172962189	0.45848918	Yes
**208365_s_at**	GRK4	G protein-coupled receptor kinase 4	2415	0.11219466477632523	0.4596204	Yes
**215668_s_at**	PLXNB1	plexin B1	2419	0.11199069768190384	0.46134418	Yes
**220576_at**	PGAP1	GPI deacylase	2443	0.11089474707841873	0.46213287	Yes
**213467_at**	RND2	Rho family GTPase 2	2475	0.11010928452014923	0.46254176	Yes
**213721_at**	SOX2	SRY (sex determining region Y)-box 2	2480	0.10999014228582382	0.46418643	Yes

**218528_s_at**	RNF38	ring finger protein 38	2517	0.10887925326824188	0.46434566	Yes
**214971_s_at**	ST6GAL1	ST6 beta-galactosamide alpha-2,6-sialyltranferase 1	2572	0.1069362610578537	0.46364737	No
**202594_at**	LEPROTL 1	leptin receptor overlapping transcript-like 1	2735	0.1020055040717125	0.457916	No
**202011_at**	TJP1	tight junction protein 1 (zona occludens 1)	2740	0.10196149349212646	0.45942724	No
**204832_s_at**	BMPR1A	bone morphogenetic protein receptor, type IA	2743	0.10192868113517761	0.46102962	No
**205230_at**	RPH3A	rabphilin 3A homolog (mouse)	2806	0.10024488717317581	0.45985338	No
**207055_at**	GPR37L1	G protein-coupled receptor 37 like 1	2850	0.09912077337503433	0.45952952	No
**206492_at**	FHIT	fragile histidine triad gene	2884	0.09840122610330582	0.4596521	No
**220454_s_at**	SEMA6A	sema domain, transmembrane domain (TM), and cytoplasmic domain, (semaphorin) 6A	2890	0.09823986887931824	0.46105567	No
**214543_x_at**	QKI	quaking homolog, KH domain RNA binding (mouse)	2904	0.09793934971094131	0.46208748	No
**214541_s_at**	QKI	quaking homolog, KH domain RNA binding (mouse)	2963	0.09676247835159302	0.4610367	No
**213793_s_at**	HOMER1	homer homolog 1 (Drosophila)	3076	0.09388771653175354	0.4574626	No
**214966_at**	GRIK5	glutamate receptor, ionotropic, kainate 5	3114	0.09304976463317871	0.45731288	No
**200795_at**	SPARCL1	SPARC-like 1 (mast9, hevin)	3125	0.09290486574172974	0.45839855	No
**212831_at**	MEGF9	multiple EGF-like-domains 9	3226	0.09057017415761948	0.45531943	No
**215025_at**	NTRK3	neurotrophic tyrosine kinase, receptor, type 3	3237	0.09044092893600464	0.45636415	No
**220529_at**	FLJ11710	hypothetical protein FLJ11710	3316	0.08878565579652786	0.45426396	No
**205426_s_at**	HIP1	huntingtin interacting protein 1	3326	0.08857114613056183	0.45532343	No
**204484_at**	PIK3C2B	phosphoinositide-3-kinase, class 2, beta polypeptide	3330	0.08847223967313766	0.45665634	No
**220920_at**	ATP10B	ATPase, Class V, type 10B	3428	0.08686060458421707	0.4536531	No
**209590_at**	BMP7	Bone morphogenetic protein 7 (osteogenic protein 1)	3522	0.08508636802434921	0.45080376	No
**206140_at**	LHX2	LIM homeobox 2	3555	0.08438161015510559	0.45073918	No
**213722_at**	SOX2	SRY (sex determining region Y)-box 2	3557	0.08430681377649307	0.45209455	No
**210600_s_at**	GRK4	G protein-coupled receptor kinase 4	3703	0.08201771974563599	0.44681028	No
**209597_s_at**	PNMA2	paraneoplastic antigen MA2	3704	0.08201591670513153	0.44817343	No
**210100_s_at**	ABCA2	ATP-binding cassette, sub-family A (ABC1), member 2	3734	0.08145365118980408	0.44819772	No
**211219_s_at**	LHX2	LIM homeobox 2	3772	0.08065430074930191	0.44784197	No
**34697_at**	LRP6	low density lipoprotein receptor-related protein 6	3791	0.08029322326183319	0.4483513	No
**205606_at**	LRP6	low density lipoprotein receptor-related protein 6	3800	0.08022619783878326	0.4493179	No
**219255_x_at**	IL17RB	interleukin 17 receptor B	3845	0.07943178713321686	0.44862095	No
**219738_s_at**	PCDH9	protocadherin 9	3877	0.0788387805223465	0.4485101	No
**209598_at**	PNMA2	paraneoplastic antigen MA2	3899	0.07830220460891724	0.44884878	No

**211913_s_at**	MERTK	c-mer proto-oncogene tyrosine kinase /// c-mer proto-oncogene tyrosine kinase	3925	0.07773786038160324	0.4489947	No
**219737_s_at**	PCDH9	protocadherin 9	3930	0.07771836221218109	0.45010304	No
**212830_at**	MEGF9	multiple EGF-like-domains 9	4008	0.07639111578464508	0.44784266	No
**203069_at**	SV2A	synaptic vesicle glycoprotein 2A	4069	0.07546748965978622	0.4463463	No
**215310_at**	APC	Adenomatosis polyposis coli	4101	0.07501500099897385	0.4461719	No
**215153_at**	NOS1AP	nitric oxide synthase 1 (neuronal) adaptor protein	4110	0.07491857558488846	0.44705033	No
**215962_at**	SNTG1	Syntrophin, gamma 1	4121	0.07471383363008499	0.44783366	No
**208522_s_at**	PTCH1	patched homolog 1 (Drosophila)	4169	0.07409704476594925	0.4469105	No
**220619_at**	CHD7	chromodomain helicase DNA binding protein 7	4226	0.07292832434177399	0.4455553	No
**216113_at**	ABI2	Abl interactor 2	4228	0.07291719317436218	0.44672137	No
**205523_at**	HAPLN1	hyaluronan and proteoglycan link protein 1	4255	0.07254550606012344	0.44673514	No
**205643_s_at**	PPP2R2B	protein phosphatase 2 (formerly 2A), regulatory subunit B (PR 52), beta isoform	4283	0.07224571704864502	0.4466981	No
**204600_at**	EPHB3	EPH receptor B3	4325	0.07174788415431976	0.44601095	No
**218899_s_at**	BAALC	brain and acute leukemia, cytoplasmic	4444	0.07016818225383759	0.44176754	No
**208920_at**	SRI	sorcin	4469	0.06982914358377457	0.44182786	No
**208564_at**	KCNA2	potassium voltage-gated channel, shaker-related subfamily, member 2	4526	0.06893665343523026	0.44040635	No
**205723_at**	CNTFR	ciliary neurotrophic factor receptor	4574	0.06805847585201263	0.43938282	No
**201998_at**	ST6GAL1	ST6 beta-galactosamide alpha-2,6-sialyltranferase 1	4585	0.06792532652616501	0.4400533	No
**212772_s_at**	ABCA2	ATP-binding cassette, sub-family A (ABC1), member 2	4617	0.0674629732966423	0.4397534	No
**211534_x_at**	PTPRN2	protein tyrosine phosphatase, receptor type, N polypeptide 2	4637	0.0672188475728035	0.43999955	No
**216487_at**	ITPR2	Inositol 1,4,5-triphosphate receptor, type 2	4807	0.06510455161333084	0.43333393	No
**214833_at**	TMEM63 A	transmembrane protein 63A	4822	0.0649619996547699	0.43377182	No
**205475_at**	SCRG1	scrapie responsive protein 1	4833	0.0648324117064476	0.4343909	No
**202661_at**	ITPR2	inositol 1,4,5-triphosphate receptor, type 2	4861	0.06432726234197617	0.43422225	No
**203030_s_at**	PTPRN2	protein tyrosine phosphatase, receptor type, N polypeptide 2	4862	0.06432104110717773	0.4352913	No
**206826_at**	PMP2	peripheral myelin protein 2	4913	0.06371486186981201	0.43405804	No
**205957_at**	PLXNB3	plexin B3	4945	0.06329122930765152	0.4336888	No
**209856_x_at**	ABI2	abl interactor 2	5019	0.06252259761095047	0.43138131	No
**214255_at**	ATP10A	ATPase, Class V, type 10A	5087	0.06160857155919075	0.4293337	No
**221002_s_at**	TSPAN14	tetraspanin 14 /// tetraspanin 14	5119	0.06133117899298668	0.42893186	No
**221615_at**	BMP8B	bone morphogenetic protein 8b (osteogenic protein 2)	5163	0.060924120247364044	0.42797315	No

**214786_at**	MAP3K1	mitogen-activated protein kinase kinase kinase 1	5247	0.05998532846570015	0.42516506	No
**212636_at**	QKI	quaking homolog, KH domain RNA binding (mouse)	5281	0.05966051667928696	0.42464375	No
**213849_s_at**	PPP2R2B	protein phosphatase 2 (formerly 2A), regulatory subunit B (PR 52), beta isoform	5295	0.0595204159617424	0.42503703	No
**215959_at**	PPFIBP2	PTPRF interacting protein, binding protein 2 (liprin beta 2)	5393	0.05839727073907852	0.42156073	No
**205524_s_at**	HAPLN1	hyaluronan and proteoglycan link protein 1	5430	0.05813540145754814	0.42087656	No
**215740_at**	PTPRN2	Protein tyrosine phosphatase, receptor type, N polypeptide 2	5434	0.05808907002210617	0.4217045	No
**200953_s_at**	CCND2	cyclin D2	5437	0.05805324763059616	0.42257765	No
**203940_s_at**	VASH1	vasohibin 1	5525	0.05703815072774887	0.41953722	No
**213469_at**	PGAP1	GPI deacylase	5546	0.05684385448694229	0.41956508	No
**219877_at**	ZMAT4	zinc finger, matrin type 4	5549	0.05681689456105232	0.42041773	No
**203861_s_at**	ACTN2	actinin, alpha 2	5642	0.055955223739147186	0.41713002	No
**210412_at**	GRIN2B	glutamate receptor, ionotropic, N-methyl D-aspartate 2B	5694	0.05539704114198685	0.41571268	No
**220464_at**	MCF2L	MCF.2 cell line derived transforming sequence-like	5697	0.05535415932536125	0.416541	No
**202524_s_at**	SPOCK2	sparc/osteonectin, cwcv and kazal-like domains proteoglycan (testican) 2	5699	0.0553179569542408	0.41741458	No
**36499_at**	CELSR2	cadherin, EGF LAG seven-pass G-type receptor 2 (flamingo homolog, Drosophila)	5722	0.05507583171129227	0.41732138	No
**214118_x_at**	PCM1	pericentriolar material 1	5903	0.05343657359480858	0.40995756	No
**206751_s_at**	PCYT1B	phosphate cytidylyltransferase 1, choline, beta	5908	0.05336172506213188	0.41066107	No
**217671_at**	RFX3	Regulatory factor X, 3 (influences HLA class II expression)	6006	0.05244576185941696	0.40708584	No
**203263_s_at**	ARHGEF 9	Cdc42 guanine nucleotide exchange factor (GEF) 9	6026	0.05228346586227417	0.40708375	No
**216081_at**	LAMA4	laminin, alpha 4	6115	0.051393382251262665	0.40390363	No
**206028_s_at**	MERTK	c-mer proto-oncogene tyrosine kinase	6128	0.05129873380064964	0.4042061	No
**203549_s_at**	LPL	lipoprotein lipase	6204	0.05065936967730522	0.40160978	No
**215479_at**	SEMA6A	Sema domain, transmembrane domain (TM), and cytoplasmic domain, (semaphorin) 6A	6302	0.04974331334233284	0.39798963	No
**205073_at**	CYP2J2	cytochrome P450, family 2, subfamily J, polypeptide 2	6325	0.049514807760715485	0.39780402	No
**214796_at**	KIAA145 6	KIAA1456 protein	6329	0.04949033260345459	0.39848903	No
**212406_s_at**	PCMTD2	protein-L-isoaspartate (D-aspartate) O-	6382	0.04903286695480347	0.39692006	No

		methyltransferase domain containing 2				
**212263_at**	QKI	quaking homolog, KH domain RNA binding (mouse)	6393	0.04886188730597496	0.39727372	No
**211912_at**	MERTK	c-mer proto-oncogene tyrosine kinase /// c-mer proto-oncogene tyrosine kinase	6424	0.04854901507496834	0.3967053	No
**217502_at**	IFIT2	interferon-induced protein with tetratricopeptide repeats 2	6548	0.0474657341837883	0.39185536	No
**210411_s_at**	GRIN2B	glutamate receptor, ionotropic, N-methyl D-aspartate 2B	6617	0.04687647521495819	0.38951704	No
**214256_at**	ATP10A	ATPase, Class V, type 10A	6652	0.0465775765478611	0.38873246	No
**211538_s_at**	HSPA2	heat shock 70kDa protein 2	6742	0.045908696949481964	0.38541535	No
**222101_s_at**	DCHS1	dachsous 1 (Drosophila)	6876	0.0447600893676281	0.38006198	No
**203850_s_at**	KIF1A	kinesin family member 1A	6967	0.043913304805755615	0.37666586	No
**221413_at**	KCNAB3	potassium voltage-gated channel, shaker-related subfamily, beta member 3	7181	0.042040467262268066	0.3675998	No
**211793_s_at**	ABI2	abl interactor 2	7272	0.04138776659965515	0.36416167	No
**211568_at**	BAI3	brain-specific angiogenesis inhibitor 3	7381	0.040551621466875076	0.35988447	No
**215964_at**	SNTG1	Syntrophin, gamma 1	7382	0.04055127874016762	0.36055845	No
**217265_at**	PLLP	plasma membrane proteolipid (plasmolipin)	7399	0.040441934019327164	0.36049712	No
**209591_s_at**	BMP7	bone morphogenetic protein 7 (osteogenic protein 1)	7436	0.04018009081482887	0.3595145	No
**210480_s_at**	MYO6	myosin VI	7512	0.03968271240592003	0.35673574	No
**203215_s_at**	MYO6	myosin VI	7625	0.0388752743601799	0.3522473	No
**205331_s_at**	REEP2	receptor accessory protein 2	7714	0.038158565759658813	0.3488472	No
**209756_s_at**	MYCN	v-myc myelocytomatosis viral related oncogene, neuroblastoma derived (avian)	7793	0.0374346598982811	0.34589353	No
**216695_s_at**	TNKS	tankyrase, TRF1-interacting ankyrin-related ADP-ribose polymerase	8019	0.03586189076304436	0.33617464	No
**210989_at**	LAMA4	Laminin, alpha 4	8158	0.03470055013895035	0.33042488	No
**202834_at**	AGT	angiotensinogen (serpin peptidase inhibitor, clade A, member 8)	8246	0.03411288186907768	0.3270034	No
**214178_s_at**	SOX2	SRY (sex determining region Y)-box 2	8295	0.033807940781116486	0.32536477	No
**207636_at**	SERPINI2	serpin peptidase inhibitor, clade I (pancpin), member 2	8315	0.033653635531663895	0.32505307	No
**211164_at**	EPHA3	EPH receptor A3	8436	0.03282728046178818	0.32009736	No
**214937_x_at**	PCM1	pericentriolar material 1	8592	0.03181585669517517	0.31352028	No
**204906_at**	RPS6KA2	ribosomal protein S6 kinase, 90kDa, polypeptide 2	8629	0.031546883285045624	0.3123942	No
**202699_s_at**	TMEM63 A	transmembrane protein 63A	8695	0.031106088310480118	0.30993134	No
**209997_x_at**	PCM1	pericentriolar material 1	8886	0.029816320165991783	0.30171648	No
**207401_at**	PROX1	prospero-related homeobox 1	8930	0.029513319954276085	0.3002357	No

**219450_at**	C4ORF19	chromosome 4 open reading frame 19	8988	0.029128124937415123	0.2981067	No
**210099_at**	ABCA2	ATP-binding cassette, sub-family A (ABC1), member 2	9037	0.0288253091275692	0.29638526	No
**202523_s_at**	SPOCK2	sparc/osteonectin, cwcv and kazal-like domains proteoglycan (testican) 2	9043	0.028789151459932327	0.29663453	No
**217121_at**	TNKS	tankyrase, TRF1-interacting ankyrin-related ADP-ribose polymerase	9046	0.028761504217982292	0.29702085	No
**206154_at**	RLBP1	retinaldehyde binding protein 1	9166	0.027887379750609398	0.2920289	No
**40837_at**	TLE2	transducin-like enhancer of split 2 (E(sp1) homolog, Drosophila)	9180	0.027798522263765335	0.29189494	No
**203862_s_at**	ACTN2	actinin, alpha 2	9324	0.026713835075497627	0.2857832	No
**212912_at**	RPS6KA2	ribosomal protein S6 kinase, 90kDa, polypeptide 2	9329	0.026698358356952667	0.28604355	No
**220829_s_at**	B3GALT1	UDP-Gal:betaGlcNAc beta 1,3-galactosyltransferase, polypeptide 1	9691	0.02442343533039093	0.26989973	No
**201790_s_at**	DHCR7	7-dehydrocholesterol reductase	9723	0.024176480248570442	0.26888037	No
**214227_at**	GNG7	Guanine nucleotide binding protein (G protein), gamma 7	9736	0.024105172604322433	0.26873088	No
**219999_at**	MAN2A2	mannosidase, alpha, class 2A, member 2	9963	0.022493580356240273	0.25874394	No
**202191_s_at**	GAS7	growth arrest-specific 7	10067	0.021688371896743774	0.25438246	No
**202800_at**	SLC1A3	solute carrier family 1 (glial high affinity glutamate transporter), member 3	10107	0.02136935666203499	0.25294968	No
**211260_at**	BMP7	bone morphogenetic protein 7 (osteogenic protein 1)	10120	0.0213078074157238	0.2527537	No
**209389_x_at**	DBI	diazepam binding inhibitor (GABA receptor modulator, acyl-Coenzyme A binding protein)	10159	0.021035052835941315	0.25136122	No
**208491_s_at**	PGM5	phosphoglucomutase 5	10191	0.02082817070186138	0.25028622	No
**202192_s_at**	GAS7	growth arrest-specific 7	10217	0.020719347521662712	0.24948448	No
**207268_x_at**	ABI2	abl interactor 2	10286	0.020247986540198326	0.24670361	No
**204456_s_at**	GAS1	growth arrest-specific 1	10323	0.019932933151721954	0.2453845	No
**211070_x_at**	DBI	enzyme A binding protein)	10550	0.018555626273155212	0.23533212	No
**210738_s_at**	SLC4A4	solute carrier family 4, sodium bicarbonate cotransporter, member 4	10560	0.018470721319317818	0.2352265	No
**211077_s_at**	TLK1	tousled-like kinase 1 /// tousled-like kinase 1	10599	0.018198544159531593	0.23378688	No
**209996_x_at**	PCM1	pericentriolar material 1	10643	0.01787281408905983	0.23211263	No
**221815_at**	ABHD2	abhydrolase domain containing 2	10802	0.016891129314899445	0.22514999	No
**215428_at**	TJP1	Tight junction protein 1 (zona occludens 1)	10892	0.016282256692647934	0.22134046	No
**216614_at**	ITPR2	Inositol 1,4,5-triphosphate receptor, type 2	10927	0.01603442057967186	0.22004826	No
**202174_s_at**	PCM1	pericentriolar material 1	10941	0.01597871072590351	0.21971786	No
**202700_s_at**	TMEM63 A	transmembrane protein 63A	11028	0.015409993007779121	0.21603137	No

**220860_at**	PURG	purine-rich element binding protein G	11248	0.014127103611826897	0.20622629	No
**87100_at**	ABHD2	abhydrolase domain containing 2	11276	0.013967218808829784	0.20522062	No
**216551_x_at**	PLCG1	phospholipase C, gamma 1	11325	0.013658006675541401	0.2032471	No
**208408_at**	PTN	pleiotrophin (heparin binding growth factor 8, neurite growth-promoting factor 1)	11393	0.013187281787395477	0.20039472	No
**212841_s_at**	PPFIBP2	PTPRF interacting protein, binding protein 2 (liprin beta 2)	11619	0.011484823189675808	0.19027065	No
**216707_at**	PCDH9	Protocadherin 9	11656	0.011224310845136642	0.18880682	No
**207865_s_at**	BMP8B	bone morphogenetic protein 8b (osteogenic protein 2)	11767	0.010413113981485367	0.18393701	No
**204457_s_at**	GAS1	growth arrest-specific 1	11797	0.01021136250346899	0.18277724	No
**212265_at**	QKI	quaking homolog, KH domain RNA binding (mouse)	11880	0.009785131551325321	0.17918065	No
**204311_at**	ATP1B2	ATPase, Na+/K+ transporting, beta 2 polypeptide	11994	0.009023511782288551	0.17415023	No
**211827_s_at**	KCND3	potassium voltage-gated channel, Shal-related subfamily, member 3	12045	0.008682196959853172	0.17200232	No
**204453_at**	ZNF84	zinc finger protein 84	12121	0.008187493309378624	0.16870008	No
**207454_at**	GRIK3	glutamate receptor, ionotropic, kainate 3	12185	0.007747315336018801	0.16594066	No
**206529_x_at**	SLC26A4	solute carrier family 26, member 4	12216	0.0074711330235004425	0.16468951	No
**63825_at**	ABHD2	abhydrolase domain containing 2	12413	0.006138916127383709	0.15580606	No
**203315_at**	NCK2	NCK adaptor protein 2	12423	0.006101028528064489	0.15549487	No
**204469_at**	PTPRZ1	protein tyrosine phosphatase, receptor-type, Z polypeptide 1	12424	0.006091001443564892	0.1555961	No
**211011_at**	COL19A1	collagen, type XIX, alpha 1	12558	0.005234541371464729	0.14958583	No
**211301_at**	KCND3	potassium voltage-gated channel, Shal-related subfamily, member 3	12700	0.004086614120751619	0.14318971	No
**203185_at**	RASSF2	Ras association (RalGDS/AF-6) domain family 2	12721	0.003979467321187258	0.14233896	No
**203216_s_at**	MYO6	myosin VI	12835	0.003089692210778594	0.13720992	No
**202032_s_at**	MAN2A2	mannosidase, alpha, class 2A, member 2	12902	0.0026080645620822906	0.13422754	No
**205566_at**	ABHD2	abhydrolase domain containing 2	12924	0.002463381038978696	0.13330576	No
**215583_at**	TMEM63 A	Transmembrane protein 63A	12935	0.0023709721863269806	0.13288672	No
**217516_x_at**	ARVCF	armadillo repeat gene deletes in velocardiofacial syndrome	12988	0.002025523455813527	0.13053648	No
**204431_at**	TLE2	transducin-like enhancer of split 2 (E(sp1) homolog, Drosophila)	13040	0.0016287903999909759	0.1282255	No
**202428_x_at**	DBI	diazepam binding inhibitor (GABA receptor modulator, acyl-Coenzyme A binding protein)	13073	0.0013585996348410845	0.12678108	No
**211360_s_at**	ITPR2	inositol 1,4,5-triphosphate receptor, type 2	13130	9,51E+11	0.124229595	No

**208657_s_at**	SEPT9	septin 9	13177	6,48E+11	0.122131534	No
**213050_at**	COBL	cordon-bleu homolog (mouse)	13272	-1,15E+12	0.11782409	No
**219686_at**	STK32B	serine/threonine kinase 32B	13308	-3,64E+11	0.1162256	No
**212667_at**	SPARC	secreted protein, acidic, cysteine-rich (osteonectin)	13392	-0.0010299704736098647	0.11243765	No
**210990_s_at**	LAMA4	laminin, alpha 4	13402	-0.0010860445909202099	0.112043105	No
**219693_at**	AGPAT4	1-acylglycerol-3-phosphate O-acyltransferase 4 (lysophosphatidic acid acyltransferase, delta)	13431	-0.0013542647939175367	0.110781975	No
**218892_at**	DCHS1	dachsous 1 (Drosophila)	13535	-0.0022830679081380367	0.10609797	No
**211377_x_at**	MYCN	v-myc myelocytomatosis viral related oncogene, neuroblastoma derived (avian)	13593	-0.002733703004196286	0.10353028	No
**207234_at**	RFX3	regulatory factor X, 3 (influences HLA class II expression)	13756	-0.004068463575094938	0.09617113	No
**200952_s_at**	CCND2	cyclin D2	13893	-0.005172996316105127	0.090022296	No
**210872_x_at**	GAS7	growth arrest-specific 7	13916	-0.005442379973828793	0.089104176	No
**217509_x_at**	GRIK5	glutamate receptor, ionotropic, kainate 5	13926	-0.005540823098272085	0.08878367	No
**203111_s_at**	PTK2B	PTK2B protein tyrosine kinase 2 beta	13949	-0.005807454232126474	0.08787162	No
**200951_s_at**	CCND2	cyclin D2	13974	-0.00595773896202445	0.08687038	No
**220354_at**	MCF2L	MCF.2 cell line derived transforming sequence-like	14144	-0.007303472142666578	0.07924409	No
**204836_at**	GLDC	glycine dehydrogenase (decarboxylating)	14223	-0.008015912026166916	0.07580147	No
**205784_x_at**	ARVCF	armadillo repeat gene deletes in velocardiofacial syndrome	14255	-0.008247883059084415	0.07451738	No
**220287_at**	ADAMTS 9	ADAM metallopeptidase with thrombospondin type 1 motif, 9	14256	-0.008248437196016312	0.07465447	No
**211312_s_at**	WISP1	WNT1 inducible signaling pathway protein 1	14312	-0.008661068975925446	0.07227699	No
**202254_at**	SIPA1L1	Signal-induced proliferation-associated 1 like 1	14388	-0.00946372002363205	0.06899596	No
**211259_s_at**	BMP7	bone morphogenetic protein 7 (osteogenic protein 1)	14536	-0.010865644551813602	0.06243745	No
**202936_s_at**	SOX9	SRY (sex determining region Y)-box 9 (campomelic dysplasia, autosomal sex-reversal)	14605	-0.011483950540423393	0.05951091	No
**212447_at**	KBTBD2	kelch repeat and BTB (POZ) domain containing 2	14648	-0.011944456957280636	0.057783972	No
**215638_at**	ERBB3	v-erb-b2 erythroblastic leukemia viral oncogene homolog 3 (avian)	14695	-0.012305566109716892	0.05587966	No
**219699_at**	LGI2	leucine-rich repeat LGI family, member 2	14768	-0.01299999374896288	0.05279494	No
**216125_s_at**	RANBP9	RAN binding protein 9	14806	-0.013322141021490097	0.05132012	No
**210456_at**	PCYT1B	phosphate cytidylyltransferase 1, choline, beta	14827	-0.013552075251936913	0.050628476	No
**215871_at**	PLA2G5	phospholipase A2, group V	14914	-0.014357399195432663	0.046924498	No

**220226_at**	TRPM8	transient receptor potential cation channel, subfamily M, member 8	14992	-0.01499187108129263	0.043643665	No
**209841_s_at**	LRRN3	leucine rich repeat neuronal 3	15054	-0.015623630955815315	0.041106835	No
**205425_at**	HIP1	huntingtin interacting protein 1	15142	-0.016579143702983856	0.03739394	No
**41220_at**	SEPT9	septin 9	15283	-0.018195172771811485	0.031278156	No
**209840_s_at**	LRRN3	leucine rich repeat neuronal 3	15389	-0.01941380277276039	0.026787177	No
**219736_at**	TRIM36	tripartite motif-containing 36	15491	-0.02053162455558777	0.022498151	No
**211222_s_at**	HAP1	huntingtin-associated protein 1 (neuroan 1)	15494	-0.020559383556246758	0.022748167	No
**214023_x_at**	TUBB2B	tubulin, beta 2B	15636	-0.0223710797727108	0.016655946	No
**209686_at**	S100B	S100 calcium binding protein, beta (neural)	15711	-0.02331898733973503	0.013651042	No
**205363_at**	BBOX1	butyrobetaine (gamma), 2-oxoglutarate dioxygenase (gamma-butyrobetaine hydroxylase) 1	15718	-0.023401379585266113	0.013764915	No
**202789_at**	PLCG1	phospholipase C, gamma 1	15799	-0.024369673803448677	0.010502408	No
**202662_s_at**	ITPR2	inositol 1,4,5-triphosphate receptor, type 2	15825	-0.02474510669708252	0.009767574	No
**205590_at**	RASGRP1	RAS guanyl releasing protein 1 (calcium and DAG-regulated)	15846	-0.02496710605919361	0.009265651	No
**210263_at**	KCNF1	potassium voltage-gated channel, subfamily F, member 1	15925	-0.026103463023900986	0.006123649	No
**203548_s_at**	LPL	lipoprotein lipase	15963	-0.026519445702433586	0.004868174	No
**208921_s_at**	SRI	sorcin	16017	-0.02723103202879429	0.0028910185	No
**214538_x_at**	RGS6	regulator of G-protein signalling 6	16054	-0.02772645093500614	0.0017014487	No
**219247_s_at**	ZDHHC14	zinc finger, DHHC-type containing 14	16075	-0.02795548550784588	0.001249193	No
**204639_at**	ADA	adenosine deaminase	16096	-0.028294453397393227	8,03E+03	No
**212262_at**	QKI	quaking homolog, KH domain RNA binding (mouse)	16123	-0.028757309541106224	8,86E+01	No
**214970_s_at**	ST6GAL1	ST6 beta-galactosamide alpha-2,6-sialyltranferase 1	16258	-0.030453819781541824	-0.0055483975	No
**219634_at**	CHST11	carbohydrate (chondroitin 4) sulfotransferase 11	16325	-0.03125624731183052	-0.008054627	No
**201847_at**	LIPA	lipase A, lysosomal acid, cholesterol esterase (Wolman disease)	16500	-0.033457837998867035	-0.015475443	No
**220299_at**	SPATA6	spermatogenesis associated 6	16528	-0.033907677978277206	-0.016149681	No
**209695_at**	PTP4A3	protein tyrosine phosphatase type IVA, member 3	16539	-0.03401905298233032	-0.016042715	No
**201791_s_at**	DHCR7	7-dehydrocholesterol reductase	16709	-0.03640759736299515	-0.023185283	No
**210379_s_at**	TLK1	tousled-like kinase 1	16817	-0.03788388520479202	-0.027460974	No
**220983_s_at**	SPRY4	sprouty homolog 4 (Drosophila) /// sprouty homolog 4 (Drosophila)	16975	-0.04025355726480484	-0.03398949	No
**213435_at**	SATB2	SATB family member 2	16994	-0.04047943279147148	-0.034141906	No
**202595_s_at**	LEPROTL1	leptin receptor overlapping transcript-like 1	17013	-0.04084492102265358	-0.034288246	No

**215591_at**	SATB2	SATB family member 2	17101	-0.042416565120220184	-0.037571717	No
**209202_s_at**	EXTL3	exostoses (multiple)-like 3	17131	-0.042898453772068024	-0.038188215	No
**207704_s_at**	GAS7	growth arrest-specific 7	17150	-0.04325054958462715	-0.038294572	No
**211603_s_at**	ETV4	ets variant gene 4 (E1A enhancer binding protein, E1AF) /// ets variant gene 4 (E1A enhancer binding protein, E1AF)	17302	-0.045655425637960434	-0.044458244	No
**202935_s_at**	SOX9	SRY (sex determining region Y)-box 9 (campomelic dysplasia, autosomal sex-reversal)	17311	-0.045873865485191345	-0.04406256	No
**213395_at**	MLC1	megalencephalic leukoencephalopathy with subcortical cysts 1	17427	-0.0479620099067688	-0.0485375	No
**204741_at**	BICD1	bicaudal D homolog 1 (Drosophila)	17475	-0.0488295778632164	-0.049880616	No
**211604_x_at**	HAP1	huntingtin-associated protein 1 (neuroan 1) /// huntingtin-associated protein 1 (neuroan 1)	17493	-0.0492224358022213	-0.049841877	No
**203110_at**	PTK2B	PTK2B protein tyrosine kinase 2 beta	17554	-0.05052013322710991	-0.05175287	No
**204391_x_at**	TRIM24	tripartite motif-containing 24	17614	-0.05157945677638054	-0.05360041	No
**202583_s_at**	RANBP9	RAN binding protein 9	17624	-0.051855381578207016	-0.053151157	No
**211051_s_at**	EXTL3	exostoses (multiple)-like 3 /// exostoses (multiple)-like 3	17692	-0.05311805382370949	-0.055339884	No
**216705_s_at**	ADA	adenosine deaminase	17701	-0.053226109594106674	-0.054822	No
**202479_s_at**	TRIB2	tribbles homolog 2 (Drosophila)	17760	-0.054241959005594254	-0.05657945	No
**207425_s_at**	SEPT9	septin 9	17900	-0.05720384418964386	-0.06200105	No
**218806_s_at**	VAV3	vav 3 oncogene	18022	-0.05956149846315384	-0.06655827	No
**219372_at**	IFT81	intraflagellar transport 81 homolog (Chlamydomonas)	18096	-0.061014484614133835	-0.06889082	No
**220298_s_at**	SPATA6	spermatogenesis associated 6	18129	-0.06182490661740303	-0.06933028	No
**206765_at**	KCNJ2	potassium inwardly-rectifying channel, subfamily J, member 2	18178	-0.062881238758564	-0.0704857	No
**206893_at**	SALL1	sal-like 1 (Drosophila)	18306	-0.06607918441295624	-0.07520966	No
**211067_s_at**	GAS7	growth arrest-specific 7 /// growth arrest-specific 7	18429	-0.06924574822187424	-0.07965177	No
**210270_at**	RGS6	regulator of G-protein signalling 6	18498	-0.07109648734331131	-0.08158753	No
**202478_at**	TRIB2	tribbles homolog 2 (Drosophila)	18655	-0.07531750202178955	-0.08748743	No
**218309_at**	CAMK2N 1	calcium/calmodulin-dependent protein kinase II inhibitor 1	18697	-0.07670991122722626	-0.0880921	No
**211448_s_at**	RGS6	regulator of G-protein signalling 6	18766	-0.0785321444272995	-0.08990428	No
**204776_at**	THBS4	thrombospondin 4	18775	-0.07885324954986572	-0.08896047	No
**212797_at**	SORT1	sortilin 1	18806	-0.07979696244001389	-0.089009546	No
**216733_s_at**	GATM	glycine amidinotransferase (L-arginine:glycine amidinotransferase)	18909	-0.08268314599990845	-0.092311434	No
**209205_s_at**	LMO4	LIM domain only 4	18916	-0.0828782245516777	-0.09120904	No

**209204_at**	LMO4	LIM domain only 4	18973	-0.08432415127754211	-0.092374824	No
**206896_s_at**	GNG7	guanine nucleotide binding protein (G protein), gamma 7	19041	-0.08636459708213806	-0.09401098	No
**204724_s_at**	COL9A3	collagen, type IX, alpha 3	19054	-0.08670848608016968	-0.093119994	No
**206136_at**	FZD5	frizzled homolog 5 (Drosophila)	19085	-0.08758776634931564	-0.09303958	No
**209466_x_at**	PTN	pleiotrophin (heparin binding growth factor 8, neurite growth-promoting factor 1)	19117	-0.08817065507173538	-0.09299533	No
**219797_at**	MGAT4A	mannosyl (alpha-1,3-)-glycoprotein beta-1,4-N-acetylglucosaminyltransferase , isozyme A	19134	-0.08862249553203583	-0.092255905	No
**213301_x_at**	TRIM24	tripartite motif-containing 24	19187	-0.09014267474412918	-0.0931416	No
**210089_s_at**	LAMA4	laminin, alpha 4	19258	-0.09220071136951447	-0.094818294	No
**210106_at**	RDH5	retinol dehydrogenase 5 (11-cis/9-cis)	19348	-0.09516537934541702	-0.09731675	No
**216867_s_at**	PDGFA	platelet-derived growth factor alpha polypeptide	19364	-0.0960480198264122	-0.09640807	No
**211737_x_at**	PTN	omoting factor 1)	19374	-0.09634647518396378	-0.09521936	No
**205249_at**	EGR2	early growth response 2 (Krox-20 homolog, Drosophila)	19391	-0.09694518148899078	-0.094341606	No
**202606_s_at**	TLK1	tousled-like kinase 1	19487	-0.10138200968503952	-0.097011805	No
**221485_at**	B4GALT5	UDP-Gal:betaGlcNAc beta 1,4- galactosyltransferase, polypeptide 5	19626	-0.10662457346916199	-0.10156618	No
**202582_s_at**	RANBP9	RAN binding protein 9	19631	-0.10697333514690399	-0.09997162	No
**206574_s_at**	PTP4A3	protein tyrosine phosphatase type IVA, member 3	19656	-0.10809318721294403	-0.09927534	No
**200665_s_at**	SPARC	secreted protein, acidic, cysteine-rich (osteonectin) /// secreted protein, acidic, cysteine-rich (osteonectin)	19788	-0.11302465945482254	-0.103402436	No
**204201_s_at**	PTPN13	protein tyrosine phosphatase, non-receptor type 13 (APO-1/CD95 (Fas)-associated phosphatase)	19905	-0.11784041672945023	-0.10676182	No
**221484_at**	B4GALT5	UDP-Gal:betaGlcNAc beta 1,4- galactosyltransferase, polypeptide 5	19933	-0.11899551749229431	-0.106021866	No
**201133_s_at**	PJA2	praja 2, RING-H2 motif containing	20035	-0.12383551895618439	-0.10859395	No
**202660_at**	ITPR2	Inositol 1,4,5-triphosphate receptor, type 2	20086	-0.12605266273021698	-0.10879113	No
**206071_s_at**	EPHA3	EPH receptor A3	20108	-0.12693871557712555	-0.107644096	No
**219683_at**	FZD3	frizzled homolog 3 (Drosophila)	20237	-0.13350097835063934	-0.11129333	No
**53991_at**	DENND2 A	DENN/MADD domain containing 2A	20254	-0.13418114185333252	-0.1097967	No
**221886_at**	DENND2 A	DENN/MADD domain containing 2A	20337	-0.13823507726192474	-0.111258425	No
**212807_s_at**	SORT1	sortilin 1	20348	-0.13893693685531616	-0.10940769	No
**220291_at**	GDPD2	glycerophosphodiester phosphodiesterase domain containing 2	20381	-0.14072678983211517	-0.10853578	No
**210233_at**	IL1RAP	interleukin 1 receptor accessory protein	20406	-0.14178511500358582	-0.10727953	No

**203178_at**	GATM	glycine amidinotransferase (L-arginine:glycine amidinotransferase)	20435	-0.14344793558120728	-0.10617902	No
**206796_at**	WISP1	WNT1 inducible signaling pathway protein 1	20445	-0.1442936658859253	-0.10419341	No
**217757_at**	A2M	alpha-2-macroglobulin	20469	-0.1459314376115799	-0.10282241	No
**218807_at**	VAV3	vav 3 oncogene	20502	-0.14750094711780548	-0.10183791	No
**221885_at**	DENND2A	DENN/MADD domain containing 2A	20554	-0.150554358959198	-0.10167371	No
**202255_s_at**	SIPA1L1	signal-induced proliferation-associated 1 like 1	20606	-0.15369148552417755	-0.10145736	No
**209465_x_at**	PTN	pleiotrophin (heparin binding growth factor 8, neurite growth-promoting factor 1)	20791	-0.16926904022693634	-0.107079394	No
**216967_at**	GAP43	growth associated protein 43	20954	-0.18246300518512726	-0.11147357	No
**209674_at**	CRY1	cryptochrome 1 (photolyase-like)	21022	-0.18943123519420624	-0.11139672	No
**215248_at**	GRB10	growth factor receptor-bound protein 10	21031	-0.19003556668758392	-0.10860503	No
**207107_at**	RPE65	retinal pigment epithelium-specific protein 65kDa	21103	-0.20029199123382568	-0.10853105	No
**207144_s_at**	CITED1	Cbp/p300-interacting transactivator, with Glu/Asp-rich carboxy-terminal domain, 1	21276	-0.22063599526882172	-0.11274922	No
**216331_at**	ITGA7	integrin, alpha 7	21318	-0.22741307318210602	-0.11084916	No
**209410_s_at**	GRB10	growth factor receptor-bound protein 10	21351	-0.23378048837184906	-0.10843067	No
**218966_at**	MYO5C	myosin VC	21446	-0.24898718297481537	-0.10860178	No
**212558_at**	SPRY1	sprouty homolog 1, antagonist of FGF signaling (Drosophila)	21448	-0.2492828518152237	-0.104504466	No
**214180_at**	MAN1C1	mannosidase, alpha, class 1C, member 1	21500	-0.25773727893829346	-0.10255885	No
**219331_s_at**	KLHDC8A	kelch domain containing 8A	21554	-0.26660335063934326	-0.100557566	No
**205227_at**	IL1RAP	interleukin 1 receptor accessory protein	21572	-0.27019360661506653	-0.096846215	No
**202202_s_at**	LAMA4	laminin, alpha 4	21612	-0.2810133993625641	-0.09396361	No
**209663_s_at**	ITGA7	integrin, alpha 7	21692	-0.29841142892837524	-0.09262561	No
**216963_s_at**	GAP43	growth associated protein 43	21699	-0.29965510964393616	-0.087920316	No
**204471_at**	GAP43	growth associated protein 43	21728	-0.3077133595943451	-0.08408967	No
**206070_s_at**	EPHA3	EPH receptor A3	21749	-0.3153459131717682	-0.0797654	No
**219099_at**	C12ORF5	chromosome 12 open reading frame 5	21770	-0.32155483961105347	-0.07533795	No
**209409_at**	GRB10	growth factor receptor-bound protein 10	21783	-0.3254410922527313	-0.07047915	No
**206178_at**	PLA2G5	phospholipase A2, group V	21806	-0.3317764699459076	-0.06597349	No
**205463_s_at**	PDGFA	platelet-derived growth factor alpha polypeptide	21866	-0.3517414927482605	-0.06283225	No
**214913_at**	ADAMTS3	ADAM metallopeptidase with thrombospondin type 1 motif, 3	21914	-0.36738651990890503	-0.05888085	No
**201896_s_at**	PSRC1	proline/serine-rich coiled-coil 1	21934	-0.3756332993507385	-0.05350875	No
**210999_s_at**	GRB10	growth factor receptor-bound protein 10	21958	-0.387459933757782	-0.048123464	No

**220543_at**	C21ORF62	chromosome 21 open reading frame 62	21991	-0.4067578911781311	-0.04283004	No
**201792_at**	AEBP1	AE binding protein 1	22044	-0.4351990222930908	-0.037980795	No
**218918_at**	MAN1C1	mannosidase, alpha, class 1C, member 1	22051	-0.4419667720794678	-0.030910237	No
**204591_at**	CHL1	cell adhesion molecule with homology to L1CAM (close homolog of L1)	22133	-0.4938931167125702	-0.026414962	No
**215870_s_at**	PLA2G5	phospholipase A2, group V	22198	-0.5835093259811401	-0.019650888	No
**206026_s_at**	TNFAIP6	tumor necrosis factor, alpha-induced protein 6	22226	-0.639116644859314	-0.0102663655	No
**206025_s_at**	TNFAIP6	tumor necrosis factor, alpha-induced protein 6	22259	-0.7694337964057922	0.0010548462	No

Supplementary List 5 - List of the 1206 top ranking genes upregulated in PN CSCs *vs*. MES CSCs and selected based on an Adjusted P value < 0.001 e log2 Fold Change > 1 for Gene Set Enrichment Analysis (GSEA) in the comparison PN *vs*. MES.

PROBE	GENE SYMBOL	GENE TITLE	RANK IN GENE LIST	RANK METRIC SCORE	RUNNING ES	CORE ENRICHMENT
**219537_x_at**	DLL3	delta-like 3 (Drosophila)	13	0.6549095511436462	0.004172992	Yes
**204995_at**	CDK5R1	cyclin-dependent kinase 5, regulatory subunit 1 (p35)	17	0.626320481300354	0.008611345	Yes
**209987_s_at**	ASCL1	achaete-scute complex-like 1 (Drosophila)	18	0.6169173717498779	0.013123261	Yes
**205278_at**	GAD1	glutamate decarboxylase 1 (brain, 67kDa)	20	0.6125731468200684	0.01755596	Yes
**221623_at**	BCAN	brevican	21	0.6073176860809326	0.021997668	Yes
**209988_s_at**	ASCL1	achaete-scute complex-like 1 (Drosophila)	23	0.6053429841995239	0.02637749	Yes
**206190_at**	GPR17	G protein-coupled receptor 17	24	0.6010344624519348	0.030773243	Yes
**205737_at**	KCNQ2	potassium voltage-gated channel, KQT-like subfamily, member 2	27	0.5821036696434021	0.034935653	Yes
**213824_at**	OLIG2	oligodendrocyte lineage transcription factor 2	29	0.5745991468429565	0.039090626	Yes
**219196_at**	SCG3	secretogranin III	31	0.5723041892051697	0.043228813	Yes
**209914_s_at**	NRXN1	neurexin 1	35	0.5565249919891357	0.047156706	Yes
**209815_at**	PTCH1	patched homolog 1 (Drosophila)	38	0.547568678855896	0.05106654	Yes
**213825_at**	OLIG2	oligodendrocyte lineage transcription factor 2	39	0.5462126731872559	0.055061348	Yes
**214952_at**	NCAM1	neural cell adhesion molecule 1	40	0.5398505926132202	0.059009623	Yes
**204851_s_at**	DCX	doublecortex; lissencephaly, X-linked (doublecortin)	43	0.5291727185249329	0.06278492	Yes
**204762_s_at**	GNAO1	guanine nucleotide binding protein (G protein), alpha activating activity polypeptide O	45	0.526117742061615	0.06658531	Yes
**207012_at**	MMP16	matrix metallopeptidase 16 (membrane-inserted)	52	0.5204265713691711	0.07010686	Yes
**217359_s_at**	NCAM1	neural cell adhesion molecule 1	54	0.5201124548912048	0.073863335	Yes
**204850_s_at**	DCX	doublecortex; lissencephaly, X-linked (doublecortin)	60	0.5122037529945374	0.077372186	Yes
**215225_s_at**	GPR17	G protein-coupled receptor 17	62	0.511105477809906	0.08106279	Yes
**218829_s_at**	CHD7	chromodomain helicase DNA binding protein 7	66	0.5038008689880371	0.084605075	Yes
**209757_s_at**	MYCN	v-myc myelocytomatosis viral related oncogene, neuroblastoma derived (avian)	68	0.5012710094451904	0.08822375	Yes
**206051_at**	ELAVL4	ELAV (embryonic lethal, abnormal vision, Drosophila)-like 4 (Hu antigen D)	71	0.4877665042877197	0.09169621	Yes
**210016_at**	MYT1L	myelin transcription factor 1-like	72	0.4861578941345215	0.0952518	Yes
**219107_at**	BCAN	brevican	74	0.48157739639282227	0.098726444	Yes

**205651_x_at**	RAPGEF4	Rap guanine nucleotide exchange factor (GEF) 4	87	0.4601998031139374	0.10152284	Yes
**203146_s_at**	GABBR1	gamma-aminobutyric acid (GABA) B receptor, 1	91	0.45689764618873596	0.10472209	Yes
**210432_s_at**	SCN3A	sodium channel, voltage-gated, type III, alpha	101	0.44231125712394714	0.10753	Yes
**221236_s_at**	STMN4	stathmin-like 4 /// stathmin-like 4	108	0.43377649784088135	0.11041781	Yes
**203295_s_at**	ATP1A2	ATPase, Na+/K+ transporting, alpha 2 (+) polypeptide	135	0.41126593947410583	0.112192094	Yes
**215323_at**	LUZP2	leucine zipper protein 2	136	0.40814173221588135	0.1151771	Yes
**203929_s_at**	MAPT	microtubule-associated protein tau	139	0.4035382866859436	0.11803355	Yes
**209915_s_at**	NRXN1	neurexin 1	140	0.40220311284065247	0.12097512	Yes
**210015_s_at**	MAP2	microtubule-associated protein 2	150	0.389330118894577	0.12339554	Yes
**211898_s_at**	EPHB1	EPH receptor B1	156	0.38499826192855835	0.12597406	Yes
**213609_s_at**	SEZ6L	seizure related 6 homolog (mouse)-like	157	0.3846593499183655	0.12878732	Yes
**207659_s_at**	MOBP	myelin-associated oligodendrocyte basic protein	160	0.3842295706272125	0.13150255	Yes
**207447_s_at**	MGAT4C	mannosyl (alpha-1,3-)-glycoprotein beta-1,4-N-acetylglucosaminyltransferase, isozyme C (putative)	161	0.3831326961517334	0.13430464	Yes
**204870_s_at**	PCSK2	proprotein convertase subtilisin/kexin type 2	162	0.38270479440689087	0.13710362	Yes
**220115_s_at**	CDH10	cadherin 10, type 2 (T2-cadherin)	163	0.38215842843055725	0.13989858	Yes
**206401_s_at**	MAPT	microtubule-associated protein tau	165	0.3811182975769043	0.1426385	Yes
**91920_at**	BCAN	brevican	166	0.3796325623989105	0.14541501	Yes
**205344_at**	CSPG5	chondroitin sulfate proteoglycan 5 (neuroglycan C)	171	0.3777303099632263	0.14798781	Yes
**203724_s_at**	RUFY3	RUN and FYVE domain containing 3	178	0.3728996515274048	0.1504304	Yes
**210414_at**	FLRT1	fibronectin leucine rich transmembrane protein 1	185	0.3667686879634857	0.15282814	Yes
**204411_at**	KIF21B	kinesin family member 21B	187	0.3661482334136963	0.15545858	Yes
**203849_s_at**	KIF1A	kinesin family member 1A	190	0.3620590567588806	0.15801166	Yes
**217897_at**	FXYD6	FXYD domain containing ion transport regulator 6	191	0.36127081513404846	0.16065387	Yes
**213601_at**	SLIT1	slit homolog 1 (Drosophila)	192	0.36123645305633545	0.16329582	Yes
**210753_s_at**	EPHB1	EPH receptor B1	200	0.3575272858142853	0.16557854	Yes
**203930_s_at**	MAPT	microtubule-associated protein tau	202	0.35648253560066223	0.16813828	Yes
**206408_at**	LRRTM2	leucine rich repeat transmembrane neuronal 2	208	0.3538261651992798	0.17048882	Yes
**204913_s_at**	SOX11	SRY (sex determining region Y)-box 11	214	0.3492409586906433	0.17280582	Yes
**203296_s_at**	ATP1A2	ATPase, Na+/K+ transporting, alpha 2 (+) polypeptide	217	0.34670180082321167	0.17524658	Yes
**203796_s_at**	BCL7A	B-cell CLL/lymphoma 7A	221	0.3457597494125366	0.177633	Yes

**210882_s_at**	TRO	trophinin	222	0.3455111086368561	0.18015996	Yes
**204915_s_at**	SOX11	SRY (sex determining region Y)-box 11	227	0.34134334325790405	0.18246664	Yes
**213880_at**	LGR5	leucine-rich repeat-containing G protein-coupled receptor 5	232	0.34000903367996216	0.18476357	Yes
**205152_at**	SLC6A1	solute carrier family 6 (neurotransmitter transporter, GABA), member 1	233	0.3395020663738251	0.18724656	Yes
**222301_at**	C1ORF61	Chromosome 1 open reading frame 61	240	0.33588707447052	0.18941845	Yes
**219415_at**	TTYH1	tweety homolog 1 (Drosophila)	242	0.33456602692604065	0.19181791	Yes
**206039_at**	RAB33A	RAB33A, member RAS oncogene family	243	0.3343508243560791	0.19426323	Yes
**213217_at**	ADCY2	adenylate cyclase 2 (brain)	262	0.32726725935935974	0.19580273	Yes
**220889_s_at**	CA10	carbonic anhydrase X	264	0.32525932788848877	0.19813412	Yes
**209982_s_at**	NRXN2	neurexin 2	266	0.3249979317188263	0.20046361	Yes
**204586_at**	BSN	bassoon (presynaptic cytomatrix protein)	267	0.32472172379493713	0.2028385	Yes
**203928_x_at**	MAPT	microtubule-associated protein tau	268	0.32458820939064026	0.20521243	Yes
**202454_s_at**	ERBB3	v-erb-b2 erythroblastic leukemia viral oncogene homolog 3 (avian)	275	0.3210487365722656	0.2072758	Yes
**203795_s_at**	BCL7A	B-cell CLL/lymphoma 7A	279	0.31992411613464355	0.20947327	Yes
**209871_s_at**	APBA2	amyloid beta (A4) precursor protein-binding, family A, member 2 (X11-like)	288	0.31733861565589905	0.2114146	Yes
**219668_at**	GDAP1L1	ganglioside-induced differentiation-associated protein 1-like 1	291	0.3164435625076294	0.21363407	Yes
**219144_at**	DUSP26	dual specificity phosphatase 26 (putative)	294	0.3160901367664337	0.21585096	Yes
**206385_s_at**	ANK3	ankyrin 3, node of Ranvier (ankyrin G)	301	0.31259462237358093	0.2178525	Yes
**203264_s_at**	ARHGEF 9	Cdc42 guanine nucleotide exchange factor (GEF) 9	302	0.31254884600639343	0.22013837	Yes
**206678_at**	GABRA1	gamma-aminobutyric acid (GABA) A receptor, alpha 1	305	0.3114071190357208	0.222321	Yes
**209983_s_at**	NRXN2	neurexin 2	309	0.3097401261329651	0.224444	Yes
**206915_at**	NKX2-2	NK2 transcription factor related, locus 2 (Drosophila)	313	0.30857038497924805	0.22655843	Yes
**213768_s_at**	ASCL1	achaete-scute complex-like 1 (Drosophila)	318	0.3064843416213989	0.22861017	Yes
**208320_at**	CABP1	calcium binding protein 1 (calbrain)	323	0.30574509501457214	0.2306565	Yes
**39966_at**	CSPG5	chondroitin sulfate proteoglycan 5 (neuroglycan C)	325	0.3049124479293823	0.23283908	Yes
**212611_at**	DTX4	deltex 4 homolog (Drosophila)	331	0.3026011288166046	0.23481497	Yes
**219743_at**	HEY2	hairy/enhancer-of-split related with YRPW motif 2	333	0.3015672266483307	0.23697309	Yes
**205155_s_at**	SPTBN2	spectrin, beta, non-erythrocytic 2	334	0.30142924189567566	0.23917763	Yes
**209735_at**	ABCG2	ATP-binding cassette, sub-family G (WHITE), member 2	337	0.300706148147583	0.241282	Yes

**206806_at**	DGKI	diacylglycerol kinase, iota	339	0.3002871870994568	0.24343075	Yes
**203129_s_at**	KIF5C	kinesin family member 5C	340	0.2999977469444275	0.24562483	Yes
**205923_at**	RELN	reelin	341	0.29995784163475037	0.24781862	Yes
**220028_at**	ACVR2B	activin A receptor, type IIB	346	0.29736003279685974	0.24980362	Yes
**218332_at**	BEX1	brain expressed, X-linked 1	354	0.29547441005706787	0.2516325	Yes
**210341_at**	MYT1	myelin transcription factor 1	361	0.2943975627422333	0.25350097	Yes
**209460_at**	ABAT	4-aminobutyrate aminotransferase	365	0.29391294717788696	0.25550818	Yes
**206888_s_at**	ARHGDIG	Rho GDP dissociation inhibitor (GDI) gamma	367	0.29335302114486694	0.25760624	Yes
**216307_at**	DGKB	diacylglycerol kinase, beta 90kDa	378	0.2891417145729065	0.25924647	Yes
**212677_s_at**	CEP68	centrosomal protein 68kDa	382	0.2882428467273712	0.26121223	Yes
**217053_x_at**	ETV1	ets variant gene 1	383	0.2874913513660431	0.26331484	Yes
**214043_at**	PTPRD	Protein tyrosine phosphatase, receptor type, D	385	0.2865099310874939	0.26536283	Yes
**218902_at**	NOTCH1	Notch homolog 1, translocation-associated (Drosophila)	387	0.28616032004356384	0.26740825	Yes
**215807_s_at**	PLXNB1	plexin B1	390	0.2852330803871155	0.26939946	Yes
**204743_at**	TAGLN3	transgelin 3	394	0.2834824323654175	0.27133042	Yes
**215785_s_at**	CYFIP2	cytoplasmic FMR1 interacting protein 2	397	0.28300976753234863	0.27330536	Yes
**213362_at**	PTPRD	Protein tyrosine phosphatase, receptor type, D	398	0.28284069895744324	0.27537397	Yes
**219945_at**	DDX25	DEAD (Asp-Glu-Ala-Asp) box polypeptide 25	402	0.28193312883377075	0.2772936	Yes
**205103_at**	C1ORF61	chromosome 1 open reading frame 61	403	0.28192245960235596	0.27935547	Yes
**213219_at**	ADCY2	adenylate cyclase 2 (brain)	408	0.28094255924224854	0.2812204	Yes
**206692_at**	KCNJ10	potassium inwardly-rectifying channel, subfamily J, member 10	409	0.2801637351512909	0.28326944	Yes
**204914_s_at**	SOX11	SRY (sex determining region Y)-box 11	431	0.27427634596824646	0.28427905	Yes
**219564_at**	KCNJ16	potassium inwardly-rectifying channel, subfamily J, member 16	439	0.27215638756752014	0.28593737	Yes
**204428_s_at**	LCAT	lecithin-cholesterol acyltransferase	442	0.2712463140487671	0.2878263	Yes
**209431_s_at**	PATZ1	POZ (BTB) and AT hook containing zinc finger 1	452	0.26877695322036743	0.28936502	Yes
**207781_s_at**	ZNF711	zinc finger protein 711	454	0.26772165298461914	0.2912756	Yes
**205413_at**	MPPED2	metallophosphoesterase domain containing 2	456	0.2674659192562103	0.2931843	Yes
**221911_at**	ETV1	ets variant gene 1	461	0.2665580213069916	0.29494405	Yes
**215164_at**	TCF4	Transcription factor 4	467	0.26542702317237854	0.29664806	Yes
**205184_at**	GNG4	guanine nucleotide binding protein (G protein), gamma 4	478	0.2635939121246338	0.29810143	Yes
**216096_s_at**	NRXN1	neurexin 1	483	0.2630660831928253	0.29983562	Yes
**219152_at**	PODXL2	podocalyxin-like 2	484	0.2630467712879181	0.30175945	Yes
**209459_s_at**	ABAT	4-aminobutyrate aminotransferase	486	0.2625848352909088	0.30363247	Yes

**210247_at**	SYN2	synapsin II	492	0.2601883113384247	0.30529818	Yes
**205318_at**	KIF5A	kinesin family member 5A	502	0.25827643275260925	0.3067601	Yes
**202242_at**	TSPAN7	tetraspanin 7	513	0.2560529410839081	0.30815834	Yes
**203130_s_at**	KIF5C	kinesin family member 5C	520	0.2547111511230469	0.30973652	Yes
**210315_at**	SYN2	synapsin II	521	0.2546623647212982	0.31159905	Yes
**205375_at**	MDFI	MyoD family inhibitor	524	0.25345656275749207	0.31335783	Yes
**209558_s_at**	HIP1R	huntingtin interacting protein 1 related	526	0.2531532645225525	0.31516188	Yes
**219170_at**	FSD1	fibronectin type III and SPRY domain containing 1	529	0.2523006200790405	0.31691223	Yes
**206670_s_at**	GAD1	glutamate decarboxylase 1 (brain, 67kDa)	539	0.2506353259086609	0.31831828	Yes
**206501_x_at**	ETV1	ets variant gene 1	548	0.24899880588054657	0.31975982	Yes
**205814_at**	GRM3	glutamate receptor, metabotropic 3	551	0.24750317633152008	0.32147506	Yes
**213939_s_at**	RUFY3	RUN and FYVE domain containing 3	556	0.24635063111782074	0.323087	Yes
**205433_at**	BCHE	butyrylcholinesterase	558	0.246237114071846	0.32484046	Yes
**204301_at**	KBTBD11	kelch repeat and BTB (POZ) domain containing 11	571	0.24379202723503113	0.32605413	Yes
**212843_at**	NCAM1	neural cell adhesion molecule 1	605	0.2355537861585617	0.32621118	Yes
**210942_s_at**	ST3GAL6	ST3 beta-galactoside alpha-2,3-sialyltransferase 6	610	0.23475052416324615	0.32773829	Yes
**206103_at**	RAC3	ras-related C3 botulinum toxin substrate 3 (rho family, small GTP binding protein Rac3)	612	0.23473027348518372	0.32940757	Yes
**204869_at**	PCSK2	proprotein convertase subtilisin/kexin type 2	615	0.2344672530889511	0.3310275	Yes
**209985_s_at**	ASCL1	achaete-scute complex-like 1 (Drosophila)	622	0.23341403901576996	0.33244994	Yes
**217687_at**	ADCY2	adenylate cyclase 2 (brain)	629	0.2326376736164093	0.3338667	Yes
**209293_x_at**	ID4	inhibitor of DNA binding 4, dominant negative helix-loop-helix protein	630	0.23224972188472748	0.3355653	Yes
**203037_s_at**	MTSS1	metastasis suppressor 1	644	0.23062153160572052	0.3366352	Yes
**208321_s_at**	CABP1	calcium binding protein 1 (calbrain)	651	0.22994865477085114	0.33803228	Yes
**210246_s_at**	ABCC8	ATP-binding cassette, sub-family C (CFTR/MRP), member 8	657	0.2286706119775772	0.33946747	Yes
**206780_at**	GAD2	glutamate decarboxylase 2 (pancreatic islets and brain, 65kDa)	664	0.22790458798408508	0.3408496	Yes
**209504_s_at**	PLEKHB1	pleckstrin homology domain containing, family B (evectins) member 1	666	0.22786666452884674	0.3424687	Yes
**203139_at**	DAPK1	death-associated protein kinase 1	667	0.22782756388187408	0.34413496	Yes
**38340_at**	HIP1R	huntingtin interacting protein 1 related	671	0.22760829329490662	0.34565726	Yes
**212850_s_at**	LRP4	low density lipoprotein receptor-related protein 4	673	0.22713911533355713	0.34727105	Yes
**211484_s_at**	DSCAM	Down syndrome cell adhesion molecule	681	0.22623206675052643	0.3485935	Yes

**208427_s_at**	ELAVL2	ELAV (embryonic lethal, abnormal vision, Drosophila)-like 2 (Hu antigen B)	682	0.22598542273044586	0.35024628	Yes
**219701_at**	TMOD2	tropomodulin 2 (neuronal)	683	0.22592082619667053	0.35189858	Yes
**205794_s_at**	NOVA1	neuro-oncological ventral antigen 1	684	0.22585149109363556	0.3535504	Yes
**220522_at**	CRB1	crumbs homolog 1 (Drosophila)	688	0.225514218211174	0.3550574	Yes
**210181_s_at**	CABP1	calcium binding protein 1 (calbrain)	689	0.2254524677991867	0.35670626	Yes
**201951_at**	ALCAM	activated leukocyte cell adhesion molecule	695	0.22470608353614807	0.35811245	Yes
**204763_s_at**	GNAO1	guanine nucleotide binding protein (G protein), alpha activating activity polypeptide O	698	0.2244458794593811	0.3596591	Yes
**205839_s_at**	BZRAP1	benzodiazapine receptor (peripheral) associated protein 1	714	0.2223183810710907	0.36057338	Yes
**217061_s_at**	ETV1	ets variant gene 1	717	0.22188569605350494	0.3621013	Yes
**209292_at**	ID4	Inhibitor of DNA binding 4, dominant negative helix-loop-helix protein	721	0.22115598618984222	0.3635764	Yes
**218005_at**	ZNF22	zinc finger protein 22 (KOX 15)	726	0.2204393446445465	0.36499885	Yes
**207336_at**	SOX5	SRY (sex determining region Y)-box 5	735	0.21875615417957306	0.3662192	Yes
**201952_at**	ALCAM	activated leukocyte cell adhesion molecule	752	0.21636928617954254	0.3670425	Yes
**214811_at**	RIMBP2	RIMS binding protein 2	762	0.21465571224689484	0.36818543	Yes
**218032_at**	SNN	stannin	763	0.21460957825183868	0.369755	Yes
**211700_s_at**	TRO	trophinin /// trophinin	767	0.21429435908794403	0.37117994	Yes
**212710_at**	CAMSAP 1	calmodulin regulated spectrin-associated protein 1	768	0.21424899995326996	0.37274688	Yes
**210508_s_at**	KCNQ2	potassium voltage-gated channel, KQT-like subfamily, member 2	778	0.21248136460781097	0.3738739	Yes
**209468_at**	LRP5	low density lipoprotein receptor-related protein 5	782	0.21192383766174316	0.37528148	Yes
**219521_at**	B3GAT1	beta-1,3-glucuronyltransferase 1 (glucuronosyltransferase P)	788	0.21088744699954987	0.37658662	Yes
**218857_s_at**	ASRGL1	asparaginase like 1	798	0.21004018187522888	0.37769577	Yes
**205747_at**	CBLN1	cerebellin 1 precursor	803	0.20916320383548737	0.37903574	Yes
**213610_s_at**	KLHL23	kelch-like 23 (Drosophila)	810	0.20829902589321136	0.3802745	Yes
**203400_s_at**	TF	transferrin	815	0.20774386823177338	0.38160408	Yes
**205889_s_at**	JAKMIP2	janus kinase and microtubule interacting protein 2	817	0.2076825350522995	0.38307554	Yes
**217231_s_at**	MAST1	microtubule associated serine/threonine kinase 1	823	0.20680105686187744	0.3843508	Yes
**209842_at**	SOX10	SRY (sex determining region Y)-box 10	831	0.20559556782245636	0.38552234	Yes
**203859_s_at**	PALM	paralemmin	839	0.20477136969566345	0.38668784	Yes
**221234_s_at**	BACH2	BTB and CNC homology 1, basic leucine zipper transcription factor 2 /// BTB	848	0.20361819863319397	0.38779747	Yes

		and CNC homology 1, basic leucine zipper transcription factor 2				
**210193_at**	MOBP	myelin-associated oligodendrocyte basic protein	851	0.20319849252700806	0.3891887	Yes
**219511_s_at**	SNCAIP	synuclein, alpha interacting protein (synphilin)	856	0.20285870134830475	0.39048257	Yes
**206090_s_at**	DISC1	disrupted in schizophrenia 1	860	0.2027972787618637	0.3918234	Yes
**207971_s_at**	CEP68	centrosomal protein 68kDa	863	0.20233099162578583	0.3932083	Yes
**205268_s_at**	ADD2	adducin 2 (beta)	864	0.20226283371448517	0.3946876	Yes
**220443_s_at**	VAX2	ventral anterior homeobox 2	865	0.20226049423217773	0.39616683	Yes
**212711_at**	CAMSAP 1	calmodulin regulated spectrin-associated protein 1	867	0.2021510899066925	0.39759785	Yes
**218006_s_at**	ZNF22	zinc finger protein 22 (KOX 15)	868	0.20214210450649261	0.39907625	Yes
**202517_at**	CRMP1	collapsin response mediator protein 1	872	0.20148856937885284	0.40040755	Yes
**211494_s_at**	SLC4A4	solute carrier family 4, sodium bicarbonate cotransporter, member 4	875	0.2013636827468872	0.40178534	Yes
**216093_at**	NCAM1	Neural cell adhesion molecule 1	880	0.201042041182518	0.40306592	Yes
**211685_s_at**	NCALD	neurocalcin delta /// neurocalcin delta	881	0.2008490264415741	0.40453488	Yes
**209343_at**	EFHD1	EF-hand domain family, member D1	882	0.20073413848876953	0.40600297	Yes
**219279_at**	DOCK10	dedicator of cytokinesis 10	885	0.20046555995941162	0.4073742	Yes
**213355_at**	ST3GAL6	ST3 beta-galactoside alpha-2,3-sialyltransferase 6	888	0.20020121335983276	0.40874353	Yes
**40148_at**	APBB2	amyloid beta (A4) precursor protein-binding, family B, member 2 (Fe65-like)	895	0.1993916928768158	0.40991712	Yes
**203097_s_at**	RAPGEF2	Rap guanine nucleotide exchange factor (GEF) 2	906	0.19859859347343445	0.41089517	Yes
**210381_s_at**	CCKBR	cholecystokinin B receptor	910	0.19831369817256927	0.41220322	Yes
**203853_s_at**	GAB2	GRB2-associated binding protein 2	915	0.19805459678173065	0.41346192	Yes
**219365_s_at**	CAMKV	CaM kinase-like vesicle-associated	916	0.1980513483285904	0.4149104	Yes
**203908_at**	SLC4A4	solute carrier family 4, sodium bicarbonate cotransporter, member 4	931	0.19577796757221222	0.41567802	Yes
**213178_s_at**	MAPK8IP 3	mitogen-activated protein kinase 8 interacting protein 3	934	0.19540831446647644	0.4170123	Yes
**209407_s_at**	DEAF1	deformed epidermal autoregulatory factor 1 (Drosophila)	942	0.19433316588401794	0.41810146	Yes
**213283_s_at**	SALL2	sal-like 2 (Drosophila)	947	0.19364716112613678	0.41932794	Yes
**211391_s_at**	PATZ1	POZ (BTB) and AT hook containing zinc finger 1	948	0.19355691969394684	0.42074355	Yes
**213419_at**	APBB2	amyloid beta (A4) precursor protein-binding, family B, member 2 (Fe65-like)	950	0.1934010088443756	0.4221106	Yes
**213683_at**	ACSL6	acyl-CoA synthetase long-chain family member 6	965	0.19216911494731903	0.4228518	Yes
**203631_s_at**	GPRC5B	G protein-coupled receptor, family C, group 5, member B	978	0.19085897505283356	0.42367834	Yes

**210251_s_at**	RUFY3	RUN and FYVE domain containing 3	999	0.18853677809238434	0.42410833	Yes
**221582_at**	HIST3H2A	histone 3, H2a	1001	0.18848684430122375	0.42543942	Yes
**206527_at**	ABAT	4-aminobutyrate aminotransferase	1008	0.18798017501831055	0.42652956	Yes
**218033_s_at**	SNN	stannin	1009	0.18796882033348083	0.4279043	Yes
**203096_s_at**	RAPGEF2	Rap guanine nucleotide exchange factor (GEF) 2	1016	0.18773013353347778	0.42899263	Yes
**216452_at**	TRPM3	transient receptor potential cation channel, subfamily M, member 3	1019	0.18755050003528595	0.43026942	Yes
**202548_s_at**	ARHGEF7	Rho guanine nucleotide exchange factor (GEF) 7	1022	0.18737341463565826	0.4315449	Yes
**210560_at**	GBX2	gastrulation brain homeobox 2	1033	0.18625997006893158	0.43243268	Yes
**211701_s_at**	TRO	trophinin /// trophinin	1042	0.18566827476024628	0.43341103	Yes
**207437_at**	NOVA1	neuro-oncological ventral antigen 1	1058	0.18462538719177246	0.43404967	Yes
**216047_x_at**	SEZ6L	seizure related 6 homolog (mouse)-like	1073	0.18303874135017395	0.4347241	Yes
**206107_at**	RGS11	regulator of G-protein signalling 11	1090	0.18184123933315277	0.4352949	Yes
**219429_at**	FA2H	fatty acid 2-hydroxylase	1112	0.1793103665113449	0.43560997	Yes
**213122_at**	TSPYL5	TSPY-like 5	1133	0.17746655642986298	0.43595898	Yes
**205320_at**	APC2	adenomatosis polyposis coli 2	1147	0.1756337732076645	0.43662673	Yes
**220999_s_at**	CYFIP2	cytoplasmic FMR1 interacting protein 2 /// cytoplasmic FMR1 interacting protein 2	1155	0.17534762620925903	0.43757704	Yes
**208986_at**	TCF12	transcription factor 12 (HTF4, helix-loop-helix transcription factors 4)	1202	0.17190849781036377	0.43665186	Yes
**207151_at**	ADCYAP1R1	adenylate cyclase activating polypeptide 1 (pituitary) receptor type I	1204	0.1718164086341858	0.43786103	Yes
**207873_x_at**	SEZ6L	seizure related 6 homolog (mouse)-like	1208	0.17126499116420746	0.43897125	Yes
**222073_at**	COL4A3	collagen, type IV, alpha 3 (Goodpasture antigen)	1238	0.16919894516468048	0.4388328	Yes
**208552_at**	GRIK4	glutamate receptor, ionotropic, kainate 4	1240	0.16913606226444244	0.44002235	Yes
**202547_s_at**	ARHGEF7	Rho guanine nucleotide exchange factor (GEF) 7	1246	0.1686599850654602	0.44101864	Yes
**210383_at**	SCN1A	sodium channel, voltage-gated, type I, alpha	1248	0.1684713065624237	0.44220334	Yes
**212361_s_at**	ATP2A2	ATPase, Ca++ transporting, cardiac muscle, slow twitch 2	1260	0.16761435568332672	0.44290733	Yes
**211899_s_at**	TRAF4	TNF receptor-associated factor 4	1262	0.1675788015127182	0.44408548	Yes
**219663_s_at**	TMEM121	transmembrane protein 121	1267	0.1672510951757431	0.44511893	Yes
**214063_s_at**	TF	transferrin	1271	0.16677160561084747	0.4461963	Yes
**206030_at**	ASPA	aspartoacylase (Canavan disease)	1276	0.16649048030376434	0.44722417	Yes
**219894_at**	MAGEL2	MAGE-like 2	1279	0.16617967188358307	0.44834468	Yes
**220147_s_at**	FAM60A	family with sequence similarity 60, member A	1280	0.16608281433582306	0.44955933	Yes

**213749_at**	MASP1	mannan-binding lectin serine peptidase 1 (C4/C2 activating component of Ra-reactive factor)	1284	0.16580602526664734	0.45062965	Yes
**202986_at**	ARNT2	aryl-hydrocarbon receptor nuclear translocator 2	1288	0.16556619107723236	0.4516982	Yes
**206330_s_at**	SHC3	SHC (Src homology 2 domain containing) transforming protein 3	1293	0.1653205156326294	0.4527175	Yes
**209583_s_at**	CD200	CD200 molecule	1295	0.16524042189121246	0.45387858	Yes
**220739_s_at**	CNNM3	cyclin M3	1305	0.16455009579658508	0.45465505	Yes
**204718_at**	EPHB6	EPH receptor B6	1318	0.16406011581420898	0.45528558	Yes
**221585_at**	CACNG4	calcium channel, voltage-dependent, gamma subunit 4	1326	0.1635659784078598	0.45614973	Yes
**210581_x_at**	PATZ1	POZ (BTB) and AT hook containing zinc finger 1	1332	0.16330087184906006	0.45710683	Yes
**205593_s_at**	PDE9A	phosphodiesterase 9A	1348	0.1617581844329834	0.45757818	Yes
**200884_at**	CKB	creatine kinase, brain	1366	0.16090738773345947	0.45794845	Yes
**217025_s_at**	DBN1	drebrin 1	1367	0.16087786853313446	0.45912504	Yes
**204343_at**	ABCA3	ATP-binding cassette, sub-family A (ABC1), member 3	1388	0.15983474254608154	0.45934513	Yes
**214821_at**	SLC25A4	Solute carrier family 25 (mitochondrial carrier; adenine nucleotide translocator), member 4	1389	0.15964198112487793	0.4605127	Yes
**209582_s_at**	CD200	CD200 molecule	1419	0.15787556767463684	0.46029142	Yes
**219645_at**	CASQ1	calsequestrin 1 (fast-twitch, skeletal muscle)	1432	0.15680620074272156	0.46086892	Yes
**220679_s_at**	CDH7	cadherin 7, type 2	1468	0.15480691194534302	0.46034053	Yes
**209763_at**	CHRDL1	chordin-like 1	1473	0.15464192628860474	0.46128175	Yes
**212935_at**	MCF2L	MCF.2 cell line derived transforming sequence-like	1488	0.1538587510585785	0.4617428	Yes
**210823_s_at**	PTPRS	protein tyrosine phosphatase, receptor type, S	1504	0.15294408798217773	0.46214968	Yes
**202871_at**	TRAF4	TNF receptor-associated factor 4	1517	0.15239082276821136	0.46269488	Yes
**220727_at**	KCNK10	potassium channel, subfamily K, member 10	1531	0.15176719427108765	0.46318808	Yes
**206243_at**	TIMP4	TIMP metallopeptidase inhibitor 4	1541	0.1510978639125824	0.46386614	Yes
**216290_x_at**	DPP6	Dipeptidyl-peptidase 6	1568	0.1498195230960846	0.4637283	Yes
**211894_x_at**	SEZ6L	seizure related 6 homolog (mouse)-like /// seizure related 6 homolog (mouse)-like	1580	0.14908277988433838	0.46429673	Yes
**203120_at**	TP53BP2	tumor protein p53 binding protein, 2	1584	0.14899973571300507	0.46524414	Yes
**202946_s_at**	BTBD3	BTB (POZ) domain containing 3	1609	0.14737869799137115	0.46518332	Yes
**209816_at**	PTCH1	patched homolog 1 (Drosophila)	1612	0.14708881080150604	0.4661642	Yes
**214070_s_at**	ATP10B	ATPase, Class V, type 10B	1618	0.1464974582195282	0.4669984	Yes
**209870_s_at**	APBA2	amyloid beta (A4) precursor protein-binding, family A, member 2 (X11-like)	1634	0.1456134021282196	0.4673517	Yes

**204777_s_at**	MAL	mal, T-cell differentiation protein	1636	0.1454414427280426	0.46836796	Yes
**219250_s_at**	FLRT3	fibronectin leucine rich transmembrane protein 3	1641	0.14526422321796417	0.46924058	Yes
**210739_x_at**	SLC4A4	solute carrier family 4, sodium bicarbonate cotransporter, member 4	1652	0.14460158348083496	0.4698237	Yes
**206135_at**	ST18	suppression of tumorigenicity 18 (breast carcinoma) (zinc finger protein)	1656	0.1443929225206375	0.4707374	Yes
**216744_at**	LUZP2	Leucine zipper protein 2	1657	0.14438173174858093	0.47179335	Yes
**210360_s_at**	MTSS1	metastasis suppressor 1	1671	0.14392578601837158	0.47222918	Yes
**203911_at**	RAP1GA P	RAP1 GTPase activating protein	1677	0.14372053742408752	0.47304308	Yes
**220405_at**	SNTG1	syntrophin, gamma 1	1678	0.1437046080827713	0.4740941	Yes
**219661_at**	RANBP17	RAN binding protein 17	1680	0.14361968636512756	0.47509703	Yes
**210307_s_at**	KLHL25	kelch-like 25 (Drosophila)	1704	0.142541766166687	0.4750483	Yes
**35147_at**	MCF2L	MCF.2 cell line derived transforming sequence-like	1712	0.1423191875219345	0.47575706	Yes
**205933_at**	SETBP1	SET binding protein 1	1741	0.14064763486385345	0.47545725	Yes
**215010_s_at**	BRSK2	BR serine/threonine kinase 2	1769	0.13932639360427856	0.4751952	Yes
**203632_s_at**	GPRC5B	G protein-coupled receptor, family C, group 5, member B	1771	0.1392555832862854	0.47616622	Yes
**200973_s_at**	TSPAN3	tetraspanin 3	1773	0.1389930695295334	0.47713533	Yes
**204030_s_at**	SCHIP1	schwannomin interacting protein 1	1817	0.13691891729831696	0.47609657	Yes
**205031_at**	EFNB3	ephrin-B3	1850	0.13504521548748016	0.475566	Yes
**219726_at**	NLGN3	neuroligin 3	1867	0.13409411907196045	0.4757876	Yes
**207897_at**	CRHR2	corticotropin releasing hormone receptor 2	1874	0.13395865261554718	0.47648266	Yes
**205821_at**	KLRK1	killer cell lectin-like receptor subfamily K, member 1	1883	0.1334802806377411	0.47707933	Yes
**209291_at**	ID4	inhibitor of DNA binding 4, dominant negative helix-loop-helix protein	1890	0.13313785195350647	0.47776836	Yes
**219194_at**	SEMA4G	sema domain, immunoglobulin domain (Ig), transmembrane domain (TM) and short cytoplasmic domain, (semaphorin) 4G	1920	0.13145846128463745	0.4773539	Yes
**203638_s_at**	FGFR2	ffer syndrome, Jackson-Weiss syndrome)	1929	0.13096927106380463	0.4779322	Yes
**213348_at**	CDKN1C	Cyclin-dependent kinase inhibitor 1C (p57, Kip2)	1939	0.1306934356689453	0.47846106	Yes
**217564_s_at**	CPS1	carbamoyl-phosphate synthetase 1, mitochondrial	1954	0.1299719512462616	0.4787474	Yes
**203026_at**	ZBTB5	zinc finger and BTB domain containing 5	1956	0.12991853058338165	0.4796501	Yes
**212982_at**	ZDHHC1 7	zinc finger, DHHC-type containing 17	1958	0.1297745704650879	0.4805518	Yes
**222146_s_at**	TCF4	transcription factor 4	1963	0.12932275235652924	0.48130783	Yes
**222258_s_at**	SH3BP4	SH3-domain binding protein 4	2013	0.1277947574853897	0.47991768	Yes
**208029_s_at**	LAPTM4 B	lysosomal associated protein transmembrane 4 beta ///	2048	0.12636232376098633	0.4792287	Yes

		lysosomal associated protein transmembrane 4 beta				
**208767_s_at**	LAPTM4 B	lysosomal associated protein transmembrane 4 beta	2060	0.12557706236839294	0.47962525	Yes
**217988_at**	CCNB1IP 1	cyclin B1 interacting protein 1	2071	0.12536166608333588	0.48006764	Yes
**213259_s_at**	SARM1	sterile alpha and TIR motif containing 1	2073	0.12517087161540985	0.48093566	Yes
**205986_at**	AATK	apoptosis-associated tyrosine kinase	2088	0.12443907558917999	0.48118153	Yes
**205712_at**	PTPRD	protein tyrosine phosphatase, receptor type, D	2091	0.12416946142911911	0.48199478	Yes
**221364_at**	GRID2	glutamate receptor, ionotropic, delta 2	2104	0.12363821268081665	0.48232967	Yes
**206939_at**	DCC	deleted in colorectal carcinoma	2114	0.123259998857975	0.48280415	Yes
**206848_at**	HOXA7	homeobox A7	2133	0.12259785830974579	0.48284677	Yes
**208477_at**	KCNC1	potassium voltage-gated channel, Shaw-related subfamily, member 1	2142	0.12199751287698746	0.48335946	Yes
**215692_s_at**	MPPED2	metallophosphoesterase domain containing 2	2145	0.12197543680667877	0.48415667	Yes
**205019_s_at**	VIPR1	vasoactive intestinal peptide receptor 1	2176	0.1205596998333931	0.48361504	Yes
**221032_s_at**	TMPRSS5	transmembrane protease, serine 5 (spinesin) /// transmembrane protease, serine 5 (spinesin)	2200	0.11990063637495041	0.4834007	Yes
**214928_at**	OBSL1	obscurin-like 1	2215	0.11917585879564285	0.4836081	Yes
**208167_s_at**	MMP16	matrix metallopeptidase 16 (membrane-inserted)	2217	0.11916545033454895	0.4844322	Yes
**203036_s_at**	MTSS1	metastasis suppressor 1	2228	0.1187651976943016	0.48482633	Yes
**204519_s_at**	PLLP	plasma membrane proteolipid (plasmolipin)	2235	0.11842784285545349	0.4854078	Yes
**208070_s_at**	REV3L	REV3-like, catalytic subunit of DNA polymerase zeta (yeast)	2239	0.11828062683343887	0.48613054	Yes
**203864_s_at**	ACTN2	actinin, alpha 2	2249	0.11790721118450165	0.48656586	Yes
**208221_s_at**	SLIT1	slit homolog 1 (Drosophila)	2255	0.11759885400533676	0.4871887	Yes
**205700_at**	HSD17B6	hydroxysteroid (17-beta) dehydrogenase 6	2256	0.1175198033452034	0.4880482	Yes
**211392_s_at**	PATZ1	POZ (BTB) and AT hook containing zinc finger 1	2272	0.11706855893135071	0.48819274	Yes
**207789_s_at**	DPP6	dipeptidyl-peptidase 6	2307	0.1157582476735115	0.48742622	Yes
**202652_at**	APBB1	amyloid beta (A4) precursor protein-binding, family B, member 1 (Fe65)	2333	0.11492689698934555	0.48708063	Yes
**209968_s_at**	NCAM1	neural cell adhesion molecule 1	2342	0.11477482318878174	0.48754048	Yes
**209559_at**	HIP1R	huntingtin interacting protein 1 related	2345	0.11467336118221283	0.48828426	Yes
**207112_s_at**	GAB1	GRB2-associated binding protein 1	2364	0.11403854191303253	0.4882643	Yes
**215314_at**	ANK3	Ankyrin 3, node of Ranvier (ankyrin G)	2385	0.11310596019029617	0.4881426	Yes
**205647_at**	RAD52	RAD52 homolog (S. cerevisiae)	2392	0.11295856535434723	0.4886841	Yes

**205262_at**	KCNH2	potassium voltage-gated channel, subfamily H (eag-related), member 2	2402	0.11265470087528229	0.489081	Yes
**213547_at**	CAND2	cullin-associated and neddylation-dissociated 2 (putative)	2412	0.11228868365287781	0.48947522	Yes
**215668_s_at**	PLXNB1	plexin B1	2419	0.11199069768190384	0.4900096	Yes
**222008_at**	COL9A1	collagen, type IX, alpha 1	2420	0.11198754608631134	0.49082866	Yes
**202806_at**	DBN1	drebrin 1	2429	0.11158332228660583	0.49126518	Yes
**220576_at**	PGAP1	GPI deacylase	2443	0.11089474707841873	0.49145943	Yes
**209442_x_at**	ANK3	ankyrin 3, node of Ranvier (ankyrin G)	2451	0.11066010594367981	0.49193665	Yes
**220316_at**	NPAS3	neuronal PAS domain protein 3	2477	0.11008314788341522	0.49155563	Yes
**213721_at**	SOX2	SRY (sex determining region Y)-box 2	2480	0.10999014228582382	0.49226516	Yes
**205405_at**	SEMA5A	sema domain, seven thrombospondin repeats (type 1 and type 1-like), transmembrane domain (TM) and short cytoplasmic domain, (semaphorin) 5A	2493	0.10952439159154892	0.49249685	Yes
**207070_at**	RGR	retinal G protein coupled receptor	2501	0.10921073704957962	0.49296346	Yes
**206732_at**	SLITRK3	SLIT and NTRK-like family, member 3	2502	0.10920432209968567	0.49376214	Yes
**206253_at**	DLG2	discs, large homolog 2, chapsyn-110 (Drosophila)	2510	0.10898575186729431	0.4942271	Yes
**221321_s_at**	KCNIP2	Kv channel interacting protein 2	2514	0.10892613232135773	0.49488142	Yes
**208228_s_at**	FGFR2	ffer syndrome, Jackson-Weiss syndrome)	2515	0.10891741514205933	0.495678	Yes
**219306_at**	KIF15	kinesin family member 15	2529	0.10837526619434357	0.49585384	Yes
**206233_at**	B4GALT6	UDP-Gal:betaGlcNAc beta 1,4- galactosyltransferase, polypeptide 6	2542	0.10790827125310898	0.4960737	Yes
**209494_s_at**	PATZ1	POZ (BTB) and AT hook containing zinc finger 1	2564	0.1071600615978241	0.49586108	Yes
**206708_at**	FOXN2	forkhead box N2	2573	0.10692840814590454	0.49626356	Yes
**206322_at**	SYN3	synapsin III	2580	0.10668238997459412	0.49675915	Yes
**208237_x_at**	ADAM22	ADAM metallopeptidase domain 22	2597	0.10574273020029068	0.49677336	Yes
**204137_at**	GPR137B	G protein-coupled receptor 137B	2601	0.10566823184490204	0.49740386	Yes
**219051_x_at**	METRN	meteorin, glial cell differentiation regulator	2623	0.10518742352724075	0.49717683	Yes
**212362_at**	ATP2A2	ATPase, Ca++ transporting, cardiac muscle, slow twitch 2	2649	0.10449957102537155	0.49675497	Yes
**213257_at**	SARM1	sterile alpha and TIR motif containing 1	2669	0.10381542146205902	0.4966128	Yes
**211214_s_at**	DAPK1	death-associated protein kinase 1	2684	0.10342154651880264	0.49670494	Yes
**210518_at**	CDH8	cadherin 8, type 2	2691	0.10325038433074951	0.4971754	Yes
**214983_at**	TTTY15	testis-specific transcript, Y-linked 15	2695	0.10318353027105331	0.4977877	Yes
**215611_at**	TCF12	transcription factor 12 (HTF4, helix-loop-helix transcription factors 4)	2722	0.1022481918334961	0.49730194	Yes

**210359_at**	MTSS1	metastasis suppressor 1	2728	0.10212309658527374	0.49781162	Yes
**202594_at**	LEPROTL 1	leptin receptor overlapping transcript-like 1	2735	0.1020055040717125	0.498273	Yes
**209521_s_at**	AMOT	angiomotin	2767	0.10133185982704163	0.49754328	Yes
**206585_at**	MKRN3	makorin, ring finger protein, 3	2779	0.10114347189664841	0.49776113	Yes
**212712_at**	CAMSAP 1	calmodulin regulated spectrin-associated protein 1	2783	0.10093642771244049	0.498357	Yes
**209051_s_at**	RALGDS	ral guanine nucleotide dissociation stimulator	2784	0.10089396685361862	0.4990949	Yes
**221296_at**	TECTA	tectorin alpha	2791	0.10068350285291672	0.4995466	Yes
**205230_at**	RPH3A	rabphilin 3A homolog (mouse)	2806	0.10024488717317581	0.49961552	Yes
**210550_s_at**	RASGRF1	Ras protein-specific guanine nucleotide-releasing factor 1	2809	0.10016467422246933	0.5002532	Yes
**207055_at**	GPR37L1	G protein-coupled receptor 37 like 1	2850	0.09912077337503433	0.49908033	Yes
**216309_x_at**	JRK	jerky homolog (mouse)	2857	0.09896295517683029	0.49951944	Yes
**219491_at**	LRFN4	leucine rich repeat and fibronectin type III domain containing 4	2863	0.09881506860256195	0.5000049	Yes
**216265_x_at**	MYH7	myosin, heavy polypeptide 7, cardiac muscle, beta	2866	0.09875856339931488	0.5006323	Yes
**218207_s_at**	STMN3	stathmin-like 3	2867	0.09872634708881378	0.50135434	Yes
**211207_s_at**	ACSL6	acyl-CoA synthetase long-chain family member 6	2873	0.09868761897087097	0.5018389	Yes
**207950_s_at**	ANK3	ankyrin 3, node of Ranvier (ankyrin G)	2878	0.09853394329547882	0.50236976	Yes
**206492_at**	FHIT	fragile histidine triad gene	2884	0.09840122610330582	0.5028522	Yes
**208065_at**	ST8SIA3	ST8 alpha-N-acetyl-neuraminide alpha-2,8-sialyltransferase 3	2892	0.09822498261928558	0.5032385	Yes
**216048_s_at**	RHOBTB 3	Rho-related BTB domain containing 3	2925	0.09745904803276062	0.502433	No
**220510_at**	RHBG	Rh family, B glycoprotein	2936	0.09736185520887375	0.50267065	No
**208227_x_at**	ADAM22	ADAM metallopeptidase domain 22	2983	0.09636653959751129	0.5011929	No
**218869_at**	MLYCD	malonyl-CoA decarboxylase	2995	0.09599577635526657	0.5013731	No
**216662_at**	MYO7B	myosin VIIB	3006	0.09570692479610443	0.50159866	No
**217991_x_at**	SSBP3	single stranded DNA binding protein 3	3044	0.09462647140026093	0.50053525	No
**210630_s_at**	RAD52	RAD52 homolog (S. cerevisiae)	3063	0.09417770057916641	0.50037	No
**206624_at**	USP9Y	ubiquitin specific peptidase 9, Y-linked (fat facets-like, Drosophila)	3083	0.09367545694112778	0.50015366	No
**217187_at**	MUC5AC	mucin 5AC, oligomeric mucus/gel-forming	3096	0.09343143552541733	0.5002676	No
**204233_s_at**	CHKA	choline kinase alpha	3099	0.09340747445821762	0.5008559	No
**207013_s_at**	MMP16	matrix metallopeptidase 16 (membrane-inserted)	3118	0.09300340712070465	0.5006821	No
**204874_x_at**	BAIAP3	BAI1-associated protein 3	3122	0.09298370033502579	0.5012198	No
**215822_x_at**	MYT1	myelin transcription factor 1	3129	0.09283340722322464	0.5016141	No
**203639_s_at**	FGFR2	ffer syndrome, Jackson-Weiss syndrome)	3145	0.09249762445688248	0.5015789	No

**219914_at**	ECEL1	endothelin converting enzyme-like 1	3180	0.09164201468229294	0.50063604	No
**203753_at**	TCF4	transcription factor 4	3217	0.09091014415025711	0.49959287	No
**208064_s_at**	ST8SIA3	ST8 alpha-N-acetyl-neuraminide alpha-2,8-sialyltransferase 3	3234	0.09050977230072021	0.49949571	No
**207527_at**	KCNJ9	potassium inwardly-rectifying channel, subfamily J, member 9	3250	0.09026741236448288	0.49944422	No
**204713_s_at**	F5	coagulation factor V (proaccelerin, labile factor)	3266	0.08982355147600174	0.49938947	No
**209112_at**	CDKN1B	cyclin-dependent kinase inhibitor 1B (p27, Kip1)	3282	0.08943604677915573	0.49933192	No
**210393_at**	LGR5	leucine-rich repeat-containing G protein-coupled receptor 5	3292	0.08923842012882233	0.49955755	No
**204086_at**	PRAME	preferentially expressed antigen in melanoma	3295	0.08920224756002426	0.50011504	No
**206121_at**	AMPD1	adenosine monophosphate deaminase 1 (isoform M)	3308	0.08888734877109528	0.5001958	No
**206669_at**	GAD1	glutamate decarboxylase 1 (brain, 67kDa)	3333	0.08843149989843369	0.49970388	No
**208578_at**	SCN10A	sodium channel, voltage-gated, type X, alpha	3349	0.08820153027772903	0.49963728	No
**220463_at**	TRPM3	transient receptor potential cation channel, subfamily M, member 3	3368	0.08787468820810318	0.49942595	No
**206774_at**	FRMPD1	FERM and PDZ domain containing 1	3392	0.08752831816673279	0.49897486	No
**220920_at**	ATP10B	ATPase, Class V, type 10B	3428	0.08686060458421707	0.49794957	No
**218553_s_at**	KCTD15	potassium channel tetramerisation domain containing 15	3489	0.08580724895000458	0.49573043	No
**220282_at**	RIC3	resistance to inhibitors of cholinesterase 3 homolog (C. elegans)	3498	0.08565028011798859	0.49597728	No
**215989_at**	CBX2	chromobox homolog 2 (Pc class homolog, Drosophila)	3502	0.0855984091758728	0.49646097	No
**209590_at**	BMP7	Bone morphogenetic protein 7 (osteogenic protein 1)	3522	0.08508636802434921	0.49618182	No
**206140_at**	LHX2	LIM homeobox 2	3555	0.08438161015510559	0.4952807	No
**213722_at**	SOX2	SRY (sex determining region Y)-box 2	3557	0.08430681377649307	0.49584985	No
**214602_at**	COL4A4	collagen, type IV, alpha 4	3583	0.08400199562311172	0.4952781	No
**216139_s_at**	MAPK8IP3	mitogen-activated protein kinase 8 interacting protein 3	3586	0.08395005762577057	0.4957972	No
**206232_s_at**	B4GALT6	UDP-Gal:betaGlcNAc beta 1,4- galactosyltransferase, polypeptide 6	3617	0.08343029767274857	0.494984	No
**200972_at**	TSPAN3	tetraspanin 3	3630	0.08322255313396454	0.49502334	No
**200811_at**	CIRBP	cold inducible RNA binding protein	3644	0.08290980756282806	0.4950129	No
**203524_s_at**	MPST	mercaptopyruvate sulfurtransferase	3650	0.08281807601451874	0.49538139	No
**206679_at**	APBA1	amyloid beta (A4) precursor protein-binding, family A, member 1 (X11)	3665	0.08252977579832077	0.49532077	No
**208468_at**	SOX21	SRY (sex determining region Y)-box 21	3686	0.08228618651628494	0.49497366	No
**219173_at**	MYO15B	myosin XVB pseudogene	3687	0.0822678878903389	0.49557534	No

**209597_s_at**	PNMA2	paraneoplastic antigen MA2	3704	0.08201591670513153	0.49541605	No	
**207179_at**	TLX1	T-cell leukemia homeobox 1	3724	0.08161943405866623	0.49511153	No	
**219898_at**	GPR85	G protein-coupled receptor 85	3726	0.08160798996686935	0.49566093	No	
**219534_x_at**	CDKN1C	cyclin-dependent kinase inhibitor 1C (p57, Kip2)	3732	0.08147569745779037	0.4960196	No	
**210100_s_at**	ABCA2	ATP-binding cassette, sub-family A (ABC1), member 2	3734	0.08145365118980408	0.49656788	No	
**221655_x_at**	EPS8L1	EPS8-like 1	3756	0.08094415068626404	0.49616352	No	
**214558_at**	GPR12	G protein-coupled receptor 12	3766	0.08077207952737808	0.49632725	No	
**211219_s_at**	LHX2	LIM homeobox 2	3772	0.08065430074930191	0.4966799	No	
**216894_x_at**	CDKN1C	cyclin-dependent kinase inhibitor 1C (p57, Kip2)	3773	0.08065394312143326	0.49726978	No	
**208226_x_at**	ADAM22	ADAM metallopeptidase domain 22	3775	0.08056870847940445	0.4978116	No	
**216356_x_at**	BAIAP3	BAI1-associated protein 3	3781	0.0804498940706253	0.49816275	No	
**214293_at**	Sep-11	Septin 11	3811	0.07999443262815475	0.49737188	No	
**213090_s_at**	TAF4	TAF4 RNA polymerase II, TATA box binding protein (TBP)-associated factor, 135kDa	3813	0.07996392250061035	0.49790928	No	
**205038_at**	IKZF1	IKAROS family zinc finger 1 (Ikaros)	3837	0.0796123668551445	0.4974003	No	
**220722_s_at**	SLC5A7	solute carrier family 5 (choline transporter), member 7	3885	0.07870329171419144	0.495746	No	
**216155_at**	NAV1	Neuron navigator 1	3896	0.07834555208683014	0.49584454	No	
**209598_at**	PNMA2	paraneoplastic antigen MA2	3899	0.07830220460891724	0.4963223	No	
**59375_at**	MYO15B	myosin XVB pseudogene	3901	0.07828476279973984	0.49684742	No	
**205070_at**	ING3	inhibitor of growth family, member 3	3903	0.07823556661605835	0.49737215	No	
**213177_at**	MAPK8IP3	mitogen-activated protein kinase 8 interacting protein 3	3921	0.0778149664402008	0.49713472	No	
**204099_at**	SMARCD3	SWI/SNF related, matrix associated, actin dependent regulator of chromatin, subfamily d, member 3	3997	0.07655147463083267	0.49413618	No	
**213464_at**	SHC2	SHC (Src homology 2 domain containing) transforming protein 2	3998	0.07654128223657608	0.494696	No	
**205256_at**	ZBTB39	zinc finger and BTB domain containing 39	4023	0.07613123208284378	0.4941141	No	
**208166_at**	MMP16	matrix metallopeptidase 16 (membrane-inserted)	4027	0.07605987787246704	0.49452806	No	
**208399_s_at**	EDN3	endothelin 3	4040	0.07588770240545273	0.49451372	No	
**211401_s_at**	FGFR2	ffer syndrome, Jackson-Weiss syndrome)	4062	0.0755293145775795	0.49406976	No	
**203069_at**	SV2A	synaptic vesicle glycoprotein 2A	4069	0.07546748965978622	0.49433705	No	
**204920_at**	CPS1	carbamoyl-phosphate synthetase 1, mitochondrial	4106	0.0749620869755745	0.49317726	No	
**215962_at**	SNTG1	Syntrophin, gamma 1	4121	0.07471383363008499	0.49305946	No	
**215518_at**	STXBP5L	syntaxin binding protein 5-like	4130	0.07461017370223999	0.49322557	No	
**208526_at**	OR2F1	olfactory receptor, family 2, subfamily F, member 1	4136	0.07452789694070816	0.49353343	No	

**214817_at**	UNC13A	unc-13 homolog A (C. elegans)	4151	0.07433709502220154	0.49341285	No
**204714_s_at**	F5	coagulation factor V (proaccelerin, labile factor)	4164	0.07414034008979797	0.49338576	No
**208522_s_at**	PTCH1	patched homolog 1 (Drosophila)	4169	0.07409704476594925	0.4937379	No
**205424_at**	TBKBP1	TBK1 binding protein 1	4189	0.07366108149290085	0.49337518	No
**208563_x_at**	POU3F3	POU domain, class 3, transcription factor 3	4205	0.07335122674703598	0.49319997	No
**220619_at**	CHD7	chromodomain helicase DNA binding protein 7	4226	0.07292832434177399	0.49278444	No
**216113_at**	ABI2	Abl interactor 2	4228	0.07291719317436218	0.49327028	No
**215221_at**	FOXP1	Forkhead box P1	4247	0.07270743697881699	0.49294803	No
**205523_at**	HAPLN1	hyaluronan and proteoglycan link protein 1	4255	0.07254550606012344	0.49314648	No
**207316_at**	HAS1	hyaluronan synthase 1	4288	0.07217880338430405	0.49215612	No
**203747_at**	AQP3	aquaporin 3 (Gill blood group)	4340	0.07152972370386124	0.49025956	No
**217020_at**	RARB	retinoic acid receptor, beta	4354	0.07138713449239731	0.49016488	No
**208104_s_at**	TSC22D4	TSC22 domain family, member 4 /// TSC22 domain family, member 4	4377	0.07105457782745361	0.48964077	No
**206724_at**	CBX4	chromobox homolog 4 (Pc class homolog, Drosophila)	4381	0.07098549604415894	0.4900176	No
**221393_at**	TAAR3	trace amine associated receptor 3	4411	0.07048962265253067	0.48915723	No
**218899_s_at**	BAALC	brain and acute leukemia, cytoplasmic	4444	0.07016818225383759	0.48815218	No
**216785_at**	FKBP1B	FK506 binding protein 1B, 12.6 kDa	4468	0.06985452026128769	0.4875718	No
**208920_at**	SRI	sorcin	4469	0.06982914358377457	0.48808253	No
**218469_at**	GREM1	gremlin 1, cysteine knot superfamily, homolog (Xenopus laevis)	4504	0.06925275921821594	0.48697588	No
**218468_s_at**	GREM1	gremlin 1, cysteine knot superfamily, homolog (Xenopus laevis)	4540	0.06864780187606812	0.48581737	No
**220114_s_at**	STAB2	stabilin 2	4570	0.06808468699455261	0.4849394	No
**205723_at**	CNTFR	ciliary neurotrophic factor receptor	4574	0.06805847585201263	0.48529482	No
**212772_s_at**	ABCA2	ATP-binding cassette, sub-family A (ABC1), member 2	4617	0.0674629732966423	0.48379552	No
**215987_at**	RAPGEF2	Rap guanine nucleotide exchange factor (GEF) 2	4622	0.0673607736825943	0.4840984	No
**210323_at**	TEKT2	tektin 2 (testicular)	4623	0.06736049056053162	0.48459107	No
**204223_at**	PRELP	proline/arginine-rich end leucine-rich repeat protein	4635	0.06722372770309448	0.48456082	No
**216102_at**	PHLDB1	pleckstrin homology-like domain, family B, member 1	4663	0.06699768453836441	0.4837698	No
**212565_at**	STK38L	serine/threonine kinase 38 like	4671	0.06692934036254883	0.4839272	No
**213182_x_at**	CDKN1C	cyclin-dependent kinase inhibitor 1C (p57, Kip2)	4737	0.06599050015211105	0.48132586	No
**200810_s_at**	CIRBP	cold inducible RNA binding protein	4777	0.06537070870399475	0.47995362	No
**217515_s_at**	CACNA1S	calcium channel, voltage-dependent, L type, alpha 1S subunit	4793	0.06525437533855438	0.4797192	No

**211872_s_at**	RGS11	regulator of G-protein signalling 11	4795	0.06524379551410675	0.4801489	No
**208411_x_at**	PPEF2	protein phosphatase, EF-hand calcium binding domain 2	4797	0.06522386521100998	0.48057848	No
**205475_at**	SCRG1	scrapie responsive protein 1	4833	0.0648324117064476	0.47939208	No
**204996_s_at**	CDK5R1	cyclin-dependent kinase 5, regulatory subunit 1 (p35)	4843	0.06455157697200775	0.47943717	No
**218779_x_at**	EPS8L1	EPS8-like 1	4880	0.06412055343389511	0.4781981	No
**206826_at**	PMP2	peripheral myelin protein 2	4913	0.06371486186981201	0.47714585	No
**207516_at**	CHRNB4	cholinergic receptor, nicotinic, beta 4	4919	0.06363377720117569	0.47737402	No
**202898_at**	SDC3	syndecan 3 (N-syndecan)	4933	0.06340663135051727	0.47722098	No
**217324_at**	EPHB1	EPH receptor B1	4963	0.0630660206079483	0.47630632	No
**220163_s_at**	HR	hairless homolog (mouse)	4969	0.06300701946020126	0.4765299	No
**214064_at**	TF	transferrin	4973	0.06295309960842133	0.47684798	No
**207568_at**	CHRNA6	cholinergic receptor, nicotinic, alpha 6	5002	0.06265553086996078	0.47597775	No
**209856_x_at**	ABI2	abl interactor 2	5019	0.06252259761095047	0.4756759	No
**39249_at**	AQP3	aquaporin 3 (Gill blood group)	5040	0.06216568499803543	0.47518167	No
**218376_s_at**	MICAL1	microtubule associated monoxygenase, calponin and LIM domain containing 1	5042	0.062138598412275314	0.47558868	No
**206615_s_at**	ADAM22	ADAM metallopeptidase domain 22	5166	0.06089816242456436	0.4701983	No
**221289_at**	DLX6	distal-less homeobox 6	5169	0.06086201220750809	0.47054854	No
**204695_at**	CDC25A	cell division cycle 25A	5185	0.060728948563337326	0.47028103	No
**212864_at**	CDS2	CDP-diacylglycerol synthase (phosphatidate cytidylyltransferase) 2	5190	0.06067006289958954	0.47053495	No
**216409_at**	ACSL6	Acyl-CoA synthetase long-chain family member 6	5194	0.06057073920965195	0.47083563	No
**203562_at**	FEZ1	fasciculation and elongation protein zeta 1 (zygin I)	5201	0.06051987409591675	0.47099358	No
**207696_at**	FUT9	fucosyltransferase 9 (alpha (1,3) fucosyltransferase)	5217	0.0603003166615963	0.4707229	No
**205999_x_at**	CYP3A4	cytochrome P450, family 3, subfamily A, polypeptide 4	5228	0.06021495535969734	0.47068885	No
**214786_at**	MAP3K1	mitogen-activated protein kinase kinase kinase 1	5247	0.05998532846570015	0.47027355	No
**201308_s_at**	Sep-11	septin 11	5268	0.05978086590766907	0.46976185	No
**212315_s_at**	NUP210	nucleoporin 210kDa	5292	0.059553176164627075	0.46910617	No
**208242_at**	RAX	retina and anterior neural fold homeobox	5312	0.059313397854566574	0.4686385	No
**217487_x_at**	FOLH1	folate hydrolase (prostate-specific membrane antigen) 1	5318	0.05926007777452469	0.4688347	No
**205028_at**	TRO	trophinin	5358	0.05882865935564041	0.4674146	No
**208530_s_at**	RARB	retinoic acid receptor, beta	5370	0.05871797353029251	0.46732214	No
**216651_s_at**	GAD2	glutamate decarboxylase 2 (pancreatic islets and brain, 65kDa)	5390	0.058411385864019394	0.4668479	No
**205817_at**	SIX1	sine oculis homeobox homolog 1 (Drosophila)	5391	0.05840606242418289	0.46727505	No

**215959_at**	PPFIBP2	PTPRF interacting protein, binding protein 2 (liprin beta 2)	5393	0.05839727073907852	0.4676547	No
**213870_at**	COL11A2	collagen, type XI, alpha 2	5412	0.058271266520023346	0.46722686	No
**205524_s_at**	HAPLN1	hyaluronan and proteoglycan link protein 1	5430	0.05813540145754814	0.46684548	No
**216286_at**	B4GALT6	UDP-Gal:betaGlcNAc beta 1,4- galactosyltransferase, polypeptide 6	5436	0.058071110397577286	0.46703297	No
**200953_s_at**	CCND2	cyclin D2	5437	0.05805324763059616	0.46745753	No
**217486_s_at**	ZDHHC17	zinc finger, DHHC-type containing 17	5487	0.05751461163163185	0.46555337	No
**212316_at**	NUP210	nucleoporin 210kDa	5488	0.057489022612571716	0.46597382	No
**204002_s_at**	ICA1	islet cell autoantigen 1, 69kDa	5511	0.05719894543290138	0.46534836	No
**203940_s_at**	VASH1	vasohibin 1	5525	0.05703815072774887	0.46514875	No
**213469_at**	PGAP1	GPI deacylase	5546	0.05684385448694229	0.46461558	No
**37872_at**	JRK	jerky homolog (mouse)	5562	0.05673378333449364	0.46431884	No
**206572_x_at**	ZNF85	zinc finger protein 85	5580	0.056524857878685	0.46392566	No
**207826_s_at**	ID3	inhibitor of DNA binding 3, dominant negative helix-loop-helix protein	5602	0.05636050924658775	0.46334153	No
**214039_s_at**	LAPTM4B	lysosomal associated protein transmembrane 4 beta	5603	0.056359149515628815	0.4637537	No
**210086_at**	HR	hairless homolog (mouse)	5609	0.0562567375600338	0.46392792	No
**204394_at**	SLC43A1	solute carrier family 43, member 1	5610	0.05624398961663246	0.4643393	No
**205595_at**	DSG3	desmoglein 3 (pemphigus vulgaris antigen)	5613	0.05619383975863457	0.46465537	No
**205056_s_at**	GPR162	G protein-coupled receptor 162	5620	0.05613713711500168	0.46478125	No
**216743_at**	ADCY6	Adenylate cyclase 6	5625	0.05609642341732979	0.46500176	No
**203861_s_at**	ACTN2	actinin, alpha 2	5642	0.055955223739147186	0.46465188	No
**213329_at**	SRGAP2	SLIT-ROBO Rho GTPase activating protein 2	5660	0.05572003871202469	0.46425283	No
**213945_s_at**	NUP210	Nucleoporin 210kDa	5688	0.05545813590288162	0.46337742	No
**210412_at**	GRIN2B	glutamate receptor, ionotropic, N-methyl D-aspartate 2B	5694	0.05539704114198685	0.46354532	No
**220464_at**	MCF2L	MCF.2 cell line derived transforming sequence-like	5697	0.05535415932536125	0.4638553	No
**214692_s_at**	JRK	jerky homolog (mouse)	5719	0.05509088933467865	0.46326184	No
**211099_s_at**	CNGB1	cyclic nucleotide gated channel beta 1	5755	0.05469316244125366	0.4620013	No
**207626_s_at**	SLC7A2	solute carrier family 7 (cationic amino acid transporter, y+ system), member 2	5839	0.05400886386632919	0.45845833	No
**211044_at**	TRIM14	tripartite motif-containing 14 /// tripartite motif-containing 14	5861	0.05383390188217163	0.45785573	No
**210577_at**	CASR	calcium-sensing receptor (hypocalciuric hypercalcemia 1, severe neonatal hyperparathyroidism)	5876	0.0536842979490757	0.4575841	No

**211171_s_at**	PDE10A	phosphodiesterase 10A	5941	0.05311587452888489	0.45493612	No
**220194_at**	NSUN7	NOL1/NOP2/Sun domain family, member 7	5948	0.05304398760199547	0.45503938	No
**208206_s_at**	RASGRP2	RAS guanyl releasing protein 2 (calcium and DAG-regulated)	5965	0.052875958383083344	0.45466697	No
**208083_s_at**	ITGB6	integrin, beta 6	5971	0.05285454913973808	0.4548163	No
**215688_at**	RASGRF1	Ras protein-specific guanine nucleotide-releasing factor 1	5987	0.05263673514127731	0.4544896	No
**217671_at**	RFX3	Regulatory factor X, 3 (influences HLA class II expression)	6006	0.05244576185941696	0.45401916	No
**203263_s_at**	ARHGEF 9	Cdc42 guanine nucleotide exchange factor (GEF) 9	6026	0.05228346586227417	0.4535001	No
**216055_at**	PDGFB	platelet-derived growth factor beta polypeptide (simian sarcoma viral (v-sis) oncogene homolog)	6092	0.05161573365330696	0.45079365	No
**207183_at**	GPR19	G protein-coupled receptor 19	6102	0.051527801901102066	0.4507435	No
**204356_at**	LIMK1	LIM domain kinase 1	6114	0.05139835923910141	0.45059752	No
**207250_at**	SIX6	sine oculis homeobox homolog 6 (Drosophila)	6131	0.05128207430243492	0.45021346	No
**217583_at**	PAH	phenylalanine hydroxylase	6143	0.05115504562854767	0.45006567	No
**207429_at**	SLC22A2	solute carrier family 22 (organic cation transporter), member 2	6178	0.05089115351438522	0.44882476	No
**207202_s_at**	NR1I2	nuclear receptor subfamily 1, group I, member 2	6186	0.05081223323941231	0.44886425	No
**217483_at**	FOLH1	folate hydrolase (prostate-specific membrane antigen) 1	6196	0.05073104798793793	0.44880828	No
**216185_at**	FUT9	Fucosyltransferase 9 (alpha (1,3) fucosyltransferase)	6202	0.050705332309007645	0.4489419	No
**221022_s_at**	PMFBP1	polyamine modulated factor 1 binding protein 1 /// polyamine modulated factor 1 binding protein 1	6232	0.05037388950586319	0.44793442	No
**206900_x_at**	ZNF253	zinc finger protein 253	6267	0.05003442242741585	0.44668722	No
**206476_s_at**	NOVA2	neuro-oncological ventral antigen 2	6271	0.05002732574939728	0.44691077	No
**209843_s_at**	SOX10	SRY (sex determining region Y)-box 10	6279	0.04994599148631096	0.44694394	No
**206807_s_at**	ADD2	adducin 2 (beta)	6294	0.04978920891880989	0.44664383	No
**214796_at**	KIAA145 6	KIAA1456 protein	6329	0.04949033260345459	0.44539267	No
**213492_at**	COL2A1	collagen, type II, alpha 1 (primary osteoarthritis, spondyloepiphyseal dysplasia, congenital)	6335	0.04941311478614807	0.44551682	No
**210853_at**	SCN11A	sodium channel, voltage-gated, type XI, alpha	6338	0.04939498007297516	0.4457832	No
**214142_at**	ZG16	zymogen granule protein 16	6350	0.04929807037115097	0.44562185	No
**206279_at**	PRKY	protein kinase, Y-linked	6353	0.04927925392985344	0.44588736	No
**207147_at**	DLX2	distal-less homeobox 2	6368	0.04914433881640434	0.44558257	No
**205592_at**	SLC4A1	Solute carrier family 4, anion exchanger, member 1 (erythrocyte membrane	6370	0.049135174602270126	0.44589448	No

		protein band 3, Diego blood group)				
**212406_s_at**	PCMTD2	protein-L-isoaspartate (D-aspartate) O-methyltransferase domain containing 2	6382	0.04903286695480347	0.4457312	No
**216443_at**	LUZP2	Leucine zipper protein 2	6395	0.048838067799806595	0.44551903	No
**201020_at**	YWHAH	tyrosine 3-monooxygenase/tryptophan 5-monooxygenase activation protein, eta polypeptide	6403	0.048757925629615784	0.4455435	No
**221241_s_at**	BCL2L14	BCL2-like 14 (apoptosis facilitator) /// BCL2-like 14 (apoptosis facilitator)	6422	0.04859134182333946	0.44504488	No
**209050_s_at**	RALGDS	ral guanine nucleotide dissociation stimulator	6454	0.048356473445892334	0.44392774	No
**219314_s_at**	ZNF219	zinc finger protein 219	6462	0.048269905149936676	0.44394866	No
**217105_at**	MAG	malignancy-associated protein	6480	0.04810299351811409	0.4434939	No
**215768_at**	SOX5	SRY (sex determining region Y)-box 5	6547	0.047469284385442734	0.4407097	No
**221337_s_at**	ADAM29	ADAM metallopeptidase domain 29	6555	0.04744482412934303	0.44072458	No
**216787_at**	FKBP1B	FK506 binding protein 1B, 12.6 kDa	6560	0.04741218686103821	0.44088155	No
**220409_at**	CAMSAP1	calmodulin regulated spectrin-associated protein 1	6600	0.04711681976914406	0.4393758	No
**211789_s_at**	MLXIP	MLX interacting protein	6607	0.04697238281369209	0.43943465	No
**210411_s_at**	GRIN2B	glutamate receptor, ionotropic, N-methyl D-aspartate 2B	6617	0.04687647521495819	0.4393505	No
**216368_s_at**	COL4A3	collagen, type IV, alpha 3 (Goodpasture antigen)	6618	0.04687458276748657	0.4396933	No
**205080_at**	RARB	retinoic acid receptor, beta	6629	0.046758316457271576	0.43956083	No
**213778_x_at**	ZNF276	zinc finger protein 276	6670	0.04643084108829498	0.43800262	No
**207458_at**	C8ORF51	chromosome 8 open reading frame 51	6689	0.046309176832437515	0.4374873	No
**209518_at**	SMARCD1	SWI/SNF related, matrix associated, actin dependent regulator of chromatin, subfamily d, member 1	6705	0.046174515038728714	0.4371133	No
**207311_at**	DOC2B	double C2-like domains, beta	6731	0.04596186429262161	0.43626335	No
**217228_s_at**	ASB4	ankyrin repeat and SOCS box-containing 4	6752	0.04582621529698372	0.4356496	No
**214939_x_at**	MLLT4	myeloid/lymphoid or mixed-lineage leukemia (trithorax homolog, Drosophila); translocated to, 4	6755	0.04580801725387573	0.43588972	No
**220717_at**	ADAMTS20	ADAM metallopeptidase with thrombospondin type 1 motif, 20	6761	0.04574244096875191	0.43598706	No
**204380_s_at**	FGFR3	fibroblast growth factor receptor 3 (achondroplasia, thanatophoric dwarfism)	6822	0.04519418999552727	0.43347088	No
**217330_at**	DISC1	disrupted in schizophrenia 1	6851	0.04498634859919548	0.43247142	No
**205779_at**	RAMP2	receptor (calcitonin) activity modifying protein 2	6853	0.04497183859348297	0.4327529	No

**206449_s_at**	MASP1	mannan-binding lectin serine peptidase 1 (C4/C2 activating component of Ra-reactive factor)	6861	0.0449250265955925	0.43274933	No
**205327_s_at**	ACVR2A	activin A receptor, type IIA	6875	0.04476447403430939	0.43245995	No
**222101_s_at**	DCHS1	dachsous 1 (Drosophila)	6876	0.0447600893676281	0.4327873	No
**221991_at**	NXPH3	neurexophilin 3 /// neurexophilin 3	6939	0.044147513806819916	0.4301686	No
**218960_at**	TMPRSS4	transmembrane protease, serine 4	6951	0.044056374579668045	0.42996892	No
**207203_s_at**	NR1I2	nuclear receptor subfamily 1, group I, member 2	6954	0.044046588242053986	0.43019617	No
**203850_s_at**	KIF1A	kinesin family member 1A	6967	0.043913304805755615	0.42994797	No
**217574_at**	CDH8	Cadherin 8, type 2	6974	0.04387916252017021	0.42998424	No
**207887_s_at**	CALCR	calcitonin receptor	7004	0.04366377741098404	0.42892766	No
**216750_at**	APBB2	amyloid beta (A4) precursor protein-binding, family B, member 2 (Fe65-like)	7026	0.04347606003284454	0.4282493	No
**216049_at**	RHOBTB3	Rho-related BTB domain containing 3	7032	0.043431833386421204	0.4283297	No
**210618_at**	RAP1GAP	RAP1 GTPase activating protein	7070	0.04306463897228241	0.4268892	No
**211486_s_at**	KCNQ2	potassium voltage-gated channel, KQT-like subfamily, member 2	7085	0.042899973690509796	0.42653874	No
**204696_s_at**	CDC25A	cell division cycle 25A	7103	0.04277239367365837	0.42604497	No
**204379_s_at**	FGFR3	fibroblast growth factor receptor 3 (achondroplasia, thanatophoric dwarfism)	7138	0.0424848310649395	0.42474258	No
**206477_s_at**	NOVA2	neuro-oncological ventral antigen 2	7160	0.042227357625961304	0.42405507	No
**208608_s_at**	SNTB1	syntrophin, beta 1 (dystrophin-associated protein A1, 59kDa, basic component 1)	7177	0.042062245309352875	0.42360356	No
**221413_at**	KCNAB3	potassium voltage-gated channel, shaker-related subfamily, beta member 3	7181	0.042040467262268066	0.4237687	No
**210313_at**	LILRA4	leukocyte immunoglobulin-like receptor, subfamily A (with TM domain), member 4	7222	0.0417402982711792	0.42217618	No
**211793_s_at**	ABI2	abl interactor 2	7272	0.04138776659965515	0.42015406	No
**216761_at**	RAB33A	RAB33A, member RAS oncogene family	7287	0.041298240423202515	0.41979188	No
**207161_at**	KIAA0087	KIAA0087 gene product	7288	0.04129013791680336	0.42009383	No
**219039_at**	SEMA4C	sema domain, immunoglobulin domain (Ig), transmembrane domain (TM) and short cytoplasmic domain, (semaphorin) 4C	7348	0.0408315472304821	0.4175932	No
**203702_s_at**	TTLL4	tubulin tyrosine ligase-like family, member 4	7349	0.040783483535051346	0.4178915	No
**211155_s_at**	THPO	thrombopoietin (myeloproliferative leukemia virus oncogene ligand, megakaryocyte growth and development factor)	7364	0.04066287353634834	0.41752467	No

**215964_at**	SNTG1	Syntrophin, gamma 1	7382	0.04055127874016762	0.41701466	No
**217265_at**	PLLP	plasma membrane proteolipid (plasmolipin)	7399	0.040441934019327164	0.41655132	No
**208481_at**	ASB4	ankyrin repeat and SOCS box-containing 4	7418	0.04029150679707527	0.415992	No
**205039_s_at**	IKZF1	IKAROS family zinc finger 1 (Ikaros)	7430	0.04019298776984215	0.41576403	No
**209591_s_at**	BMP7	bone morphogenetic protein 7 (osteogenic protein 1)	7436	0.04018009081482887	0.4158207	No
**213181_s_at**	MOCS1	molybdenum cofactor synthesis 1	7473	0.039912957698106766	0.41440457	No
**39248_at**	AQP3	aquaporin 3 (Gill blood group)	7486	0.0398515984416008	0.4141267	No
**208234_x_at**	FGFR2	ffer syndrome, Jackson-Weiss syndrome)	7517	0.039631932973861694	0.4129932	No
**62987_r_at**	CACNG4	calcium channel, voltage-dependent, gamma subunit 4	7533	0.03948699310421944	0.4125703	No
**209931_s_at**	FKBP1B	FK506 binding protein 1B, 12.6 kDa	7548	0.03939610719680786	0.41219422	No
**214641_at**	COL4A3	collagen, type IV, alpha 3 (Goodpasture antigen)	7567	0.039290651679039	0.41162756	No
**216061_x_at**	PDGFB	platelet-derived growth factor beta polypeptide (simian sarcoma viral (v-sis) oncogene homolog)	7589	0.03912224993109703	0.41091734	No
**208545_x_at**	TAF4	TAF4 RNA polymerase II, TATA box binding protein (TBP)-associated factor, 135kDa	7590	0.039098773151636124	0.4112033	No
**206635_at**	CHRNB2	cholinergic receptor, nicotinic, beta 2 (neuronal)	7632	0.0388038344681263	0.40954185	No
**211398_at**	FGFR2	ffer syndrome, Jackson-Weiss syndrome)	7639	0.03877075016498566	0.4095407	No
**208462_s_at**	ABCC9	ATP-binding cassette, sub-family C (CFTR/MRP), member 9	7729	0.0380464605987072	0.40559638	No
**211802_x_at**	CACNA1 G	calcium channel, voltage-dependent, alpha 1G subunit	7737	0.03799137845635414	0.4055421	No
**220571_at**	PRDM11	PR domain containing 11	7751	0.03783513978123665	0.40520203	No
**209756_s_at**	MYCN	v-myc myelocytomatosis viral related oncogene, neuroblastoma derived (avian)	7793	0.0374346598982811	0.40353057	No
**214369_s_at**	RASGRP2	RAS guanyl releasing protein 2 (calcium and DAG-regulated)	7797	0.03741854056715965	0.4036619	No
**216893_s_at**	COL4A3	collagen, type IV, alpha 3 (Goodpasture antigen)	7825	0.03721407800912857	0.40265307	No
**216390_at**	LCAT	Lecithin-cholesterol acyltransferase	7842	0.03710060566663742	0.40216526	No
**220910_at**	FRAS1	Fraser syndrome 1	7846	0.037074558436870575	0.4022941	No
**207819_s_at**	ABCB4	ATP-binding cassette, sub-family B (MDR/TAP), member 4	7916	0.036612581461668015	0.39928815	No
**216747_at**	APBB2	amyloid beta (A4) precursor protein-binding, family B, member 2 (Fe65-like)	7924	0.036559995263814926	0.39922342	No
**214010_s_at**	ATP9B	ATPase, Class II, type 9B	7941	0.03642239049077034	0.3987307	No
**210547_x_at**	ICA1	islet cell autoantigen 1, 69kDa	7950	0.036377254873514175	0.39861718	No

**215239_x_at**	ZNF273	zinc finger protein 273	7952	0.036350835114717484	0.3988356	No
**203483_at**	SEMA4G	sema domain, immunoglobulin domain (Ig), transmembrane domain (TM) and short cytoplasmic domain, (semaphorin) 4G	7973	0.036229465156793594	0.39815167	No
**215804_at**	EPHA1	EPH receptor A1	7984	0.03618964925408363	0.3979419	No
**219594_at**	NINJ2	ninjurin 2	8082	0.03539733588695526	0.3935986	No
**219281_at**	MSRA	methionine sulfoxide reductase A	8083	0.035390663892030716	0.39385742	No
**208196_x_at**	NFATC1	nuclear factor of activated T-cells, cytoplasmic, calcineurin-dependent 1	8091	0.03534364700317383	0.3937838	No
**211238_at**	ADAM7	ADAM metallopeptidase domain 7	8136	0.034971728920936584	0.39195198	No
**217229_at**	ASB4	ankyrin repeat and SOCS box-containing 4	8142	0.03490102291107178	0.39197	No
**206984_s_at**	RIT2	Ras-like without CAAX 2	8143	0.03489011153578758	0.39222518	No
**221455_s_at**	WNT3	wingless-type MMTV integration site family, member 3 /// wingless-type MMTV integration site family, member 3	8149	0.03481176495552063	0.39224258	No
**219227_at**	CCNJL	cyclin J-like	8156	0.034709494560956955	0.39221174	No
**206181_at**	SLAMF1	signaling lymphocytic activation molecule family member 1	8213	0.03433835878968239	0.38980597	No
**206032_at**	DSC3	desmocollin 3	8255	0.03405901789665222	0.3881098	No
**208562_s_at**	ABCC9	ATP-binding cassette, sub-family C (CFTR/MRP), member 9	8263	0.034026648849248886	0.38802657	No
**220804_s_at**	TP73	tumor protein p73	8268	0.034011393785476685	0.3880855	No
**214178_s_at**	SOX2	SRY (sex determining region Y)-box 2	8295	0.033807940781116486	0.3870992	No
**207811_at**	KRT12	keratin 12 (Meesmann corneal dystrophy)	8338	0.033486902713775635	0.38535142	No
**208533_at**	SOX1	SRY (sex determining region Y)-box 1	8342	0.03344961628317833	0.38545373	No
**218330_s_at**	NAV2	neuron navigator 2	8358	0.033341292291879654	0.3849859	No
**211768_at**	LAT2	linker for activation of T cells family, member 2 /// linker for activation of T cells family, member 2	8368	0.03325332701206207	0.3848021	No
**211831_s_at**	THPO	thrombopoietin (myeloproliferative leukemia virus oncogene ligand, megakaryocyte growth and development factor)	8372	0.03322906419634819	0.38490278	No
**216896_at**	COL4A3	collagen, type IV, alpha 3 (Goodpasture antigen)	8398	0.033087048679590225	0.38395864	No
**215868_x_at**	SOX5	SRY (sex determining region Y)-box 5	8399	0.03308258578181267	0.3842006	No
**212919_at**	DCP2	DCP2 decapping enzyme homolog (S. cerevisiae)	8410	0.0330033153295517	0.38396752	No
**214563_at**	PCDHGC3	protocadherin gamma subfamily C, 3	8418	0.03295605257153511	0.38387644	No
**211248_s_at**	CHRD	chordin	8429	0.032889850437641144	0.38364252	No

**211164_at**	EPHA3	EPH receptor A3	8436	0.03282728046178818	0.38359794	No
**216672_s_at**	MYT1L	myelin transcription factor 1-like	8452	0.032674770802259445	0.38312525	No
**211264_at**	GAD2	glutamate decarboxylase 2 (pancreatic islets and brain, 65kDa)	8475	0.032551079988479614	0.3823195	No
**216675_at**	C9ORF106	Chromosome 9 open reading frame 106	8486	0.032504282891750336	0.3820828	No
**213335_s_at**	ST3GAL6	ST3 beta-galactoside alpha-2,3-sialyltransferase 6	8494	0.03248415142297745	0.38198826	No
**221674_s_at**	CHRD	chordin	8498	0.03245009109377861	0.38208324	No
**220343_at**	PDE7B	phosphodiesterase 7B	8521	0.03228595480322838	0.3812756	No
**220036_s_at**	LMBR1L	limb region 1 homolog (mouse)-like	8541	0.032117828726768494	0.38060904	No
**202976_s_at**	RHOBTB3	Rho-related BTB domain containing 3	8596	0.03175629675388336	0.37827924	No
**207024_at**	CHRND	cholinergic receptor, nicotinic, delta	8612	0.03165870159864426	0.37779912	No
**208412_s_at**	RARB	retinoic acid receptor, beta	8613	0.031654391437768936	0.37803063	No
**222022_at**	DTX3	Deltex 3 homolog (Drosophila)	8616	0.03160746768116951	0.37816688	No
**204906_at**	RPS6KA2	ribosomal protein S6 kinase, 90kDa, polypeptide 2	8629	0.031546883285045624	0.37782827	No
**206616_s_at**	ADAM22	ADAM metallopeptidase domain 22	8656	0.0313715897500515	0.37682414	No
**207421_at**	CA5A	carbonic anhydrase VA, mitochondrial	8675	0.03124677576124668	0.37619868	No
**219740_at**	VASH2	vasohibin 2	8694	0.031107082962989807	0.37557214	No
**214535_s_at**	ADAMTS2	ADAM metallopeptidase with thrombospondin type 1 motif, 2	8712	0.030977824702858925	0.37499216	No
**215151_at**	DOCK10	dedicator of cytokinesis 10	8737	0.030843788757920265	0.37407905	No
**214454_at**	ADAMTS2	ADAM metallopeptidase with thrombospondin type 1 motif, 2	8749	0.030766185373067856	0.37378216	No
**217702_at**	IL27RA	interleukin 27 receptor, alpha	8751	0.03075677901506424	0.37395966	No
**210380_s_at**	CACNA1G	calcium channel, voltage-dependent, alpha 1G subunit	8767	0.030672984197735786	0.37347233	No
**202409_at**	IGF2	insulin-like growth factor 2 (somatomedin A)	8824	0.030276624485850334	0.37103683	No
**216953_s_at**	WT1	Wilms tumor 1	8889	0.029800759628415108	0.3682183	No
**207401_at**	PROX1	prospero-related homeobox 1	8930	0.029513319954276085	0.36653635	No
**220634_at**	TBX4	T-box 4	8936	0.029477624222636223	0.3665147	No
**211105_s_at**	NFATC1	nuclear factor of activated T-cells, cytoplasmic, calcineurin-dependent 1	8946	0.029439030215144157	0.366303	No
**205972_at**	SLC38A3	solute carrier family 38, member 3	8957	0.029343636706471443	0.36604318	No
**220035_at**	NUP210	nucleoporin 210kDa	8971	0.029230043292045593	0.36564016	No
**204973_at**	GJB1	gap junction protein, beta 1, 32kDa (connexin 32, Charcot-Marie-Tooth neuropathy, X-linked)	8995	0.029084719717502594	0.36476165	No
**215960_at**	SLC5A4	solute carrier family 5 (low affinity glucose cotransporter), member 4	9033	0.02886972390115261	0.36321732	No

**37512_at**	HSD17B6	hydroxysteroid (17-beta) dehydrogenase 6	9036	0.02883606217801571	0.3633333	No
**210099_at**	ABCA2	ATP-binding cassette, sub-family A (ABC1), member 2	9037	0.0288253091275692	0.36354414	No
**213183_s_at**	CDKN1C	Cyclin-dependent kinase inhibitor 1C (p57, Kip2)	9058	0.028650185093283653	0.36280477	No
**207452_s_at**	CNTN5	contactin 5	9090	0.0283796563744545	0.36154154	No
**217447_at**	MAG	myelin associated glycoprotein	9098	0.028327811509370804	0.3614166	No
**211400_at**	FGFR2	ffer syndrome, Jackson-Weiss syndrome)	9099	0.028321726247668266	0.36162373	No
**206154_at**	RLBP1	retinaldehyde binding protein 1	9166	0.027887379750609398	0.3586963	No
**220791_x_at**	SCN11A	sodium channel, voltage-gated, type XI, alpha	9289	0.026959456503391266	0.3531052	No
**218457_s_at**	DNMT3A	DNA (cytosine-5-)-methyltransferase 3 alpha	9296	0.02688978798687458	0.35301718	No
**49049_at**	DTX3	deltex 3 homolog (Drosophila)	9317	0.02677629329264164	0.3522641	No
**203862_s_at**	ACTN2	actinin, alpha 2	9324	0.026713835075497627	0.35217482	No
**212912_at**	RPS6KA2	ribosomal protein S6 kinase, 90kDa, polypeptide 2	9329	0.026698358356952667	0.3521803	No
**214137_at**	PTPRJ	Protein tyrosine phosphatase, receptor type, J	9330	0.026687199249863625	0.35237548	No
**211239_s_at**	ADAM7	ADAM metallopeptidase domain 7	9356	0.02652675472199917	0.35138336	No
**216019_x_at**	PHLDB1	pleckstrin homology-like domain, family B, member 1	9467	0.02581789344549179	0.34635323	No
**210680_s_at**	MASP1	mannan-binding lectin serine peptidase 1 (C4/C2 activating component of Ra-reactive factor)	9487	0.025705929845571518	0.34563977	No
**221716_s_at**	ACSBG2	acyl-CoA synthetase bubblegum family member 2	9501	0.025617707520723343	0.34521034	No
**220503_at**	SLC13A1	solute carrier family 13 (sodium/sulfate symporters), member 1	9506	0.02559812366962433	0.34520778	No
**214750_at**	PLAC4	placenta-specific 4	9643	0.024702291935682297	0.3389359	No
**206937_at**	SPTA1	spectrin, alpha, erythrocytic 1 (elliptocytosis 2)	9646	0.024681318551301956	0.33902153	No
**216660_at**	MYO7B	myosin VIIB	9648	0.024680329486727715	0.3391546	No
**204680_s_at**	RAPGEF5	Rap guanine nucleotide exchange factor (GEF) 5	9658	0.02462179958820343	0.33890766	No
**38521_at**	MAG	myelin associated glycoprotein	9661	0.024605076760053635	0.3389927	No
**220829_s_at**	B3GALT1	UDP-Gal:betaGlcNAc beta 1,3-galactosyltransferase, polypeptide 1	9691	0.02442343533039093	0.33779544	No
**202975_s_at**	RHOBTB 3	Rho-related BTB domain containing 3	9695	0.024401050060987473	0.33783156	No
**46665_at**	SEMA4C	sema domain, immunoglobulin domain (Ig), transmembrane domain (TM) and short cytoplasmic domain, (semaphorin) 4C	9700	0.024361824616789818	0.33781996	No
**217102_at**	MAG	malignancy-associated protein	9701	0.02435067668557167	0.33799806	No
**217174_s_at**	APC2	adenomatosis polyposis coli 2	9717	0.024221740663051605	0.33746353	No

**214227_at**	GNG7	Guanine nucleotide binding protein (G protein), gamma 7	9736	0.024105172604322433	0.33678582	No
**216122_at**	NAV1	Neuron navigator 1	9745	0.02407165803015232	0.3365823	No
**203661_s_at**	TMOD1	tropomodulin 1	9772	0.02391033247113228	0.3355236	No
**203183_s_at**	SMARCD 1	SWI/SNF related, matrix associated, actin dependent regulator of chromatin, subfamily d, member 1	9798	0.02368004247546196	0.33451065	No
**205538_at**	CORO2A	coronin, actin binding protein, 2A	9802	0.023654695600271225	0.33454132	No
**211740_at**	ICA1	islet cell autoantigen 1, 69kDa /// islet cell autoantigen 1, 69kDa	9809	0.023576881736516953	0.3344291	No
**216192_at**	FABP7	Fatty acid binding protein 7, brain	9823	0.023462416604161263	0.3339839	No
**221387_at**	NPFFR1	neuropeptide FF receptor 1	9854	0.023220300674438477	0.33273038	No
**206046_at**	ADAM23	ADAM metallopeptidase domain 23	9859	0.02318810671567917	0.33271018	No
**215363_x_at**	FOLH1	folate hydrolase (prostate-specific membrane antigen) 1	9881	0.02309575118124485	0.33188275	No
**207586_at**	SHH	sonic hedgehog homolog (Drosophila)	9911	0.02287386544048786	0.33067414	No
**205719_s_at**	PAH	phenylalanine hydroxylase	9926	0.022772612050175667	0.33017644	No
**220313_at**	GPR88	G protein-coupled receptor 88	9934	0.022692011669278145	0.3300103	No
**211673_s_at**	MOCS1	molybdenum cofactor synthesis 1 /// molybdenum cofactor synthesis 1	9936	0.022682568058371544	0.33012876	No
**203540_at**	GFAP	glial fibrillary acidic protein	9937	0.022681528702378273	0.33029464	No
**207886_s_at**	CALCR	calcitonin receptor	9938	0.022679483518004417	0.3304605	No
**209195_s_at**	ADCY6	adenylate cyclase 6	9939	0.022666465491056442	0.33062628	No
**205998_x_at**	CYP3A4	cytochrome P450, family 3, subfamily A, polypeptide 4	9970	0.02243264578282833	0.32936698	No
**215104_at**	NRIP2	nuclear receptor interacting protein 2	10013	0.022092178463935852	0.32753587	No
**206706_at**	NTF3	neurotrophin 3	10046	0.021833840757608414	0.3261773	No
**211314_at**	CACNA1 G	calcium channel, voltage-dependent, alpha 1G subunit	10058	0.021747378632426262	0.32581446	No
**219707_at**	CPNE7	copine VII	10064	0.021702945232391357	0.32573596	No
**202191_s_at**	GAS7	growth arrest-specific 7	10067	0.021688371896743774	0.3257997	No
**220109_at**	TF	transferrin	10068	0.021679295226931572	0.32595825	No
**211260_at**	BMP7	bone morphogenetic protein 7 (osteogenic protein 1)	10120	0.0213078074157238	0.3236944	No
**215907_at**	BACH2	BTB and CNC homology 1, basic leucine zipper transcription factor 2	10131	0.02123257890343666	0.32337523	No
**218778_x_at**	EPS8L1	EPS8-like 1	10136	0.021192079409956932	0.32334045	No
**205710_at**	LRP2	low density lipoprotein-related protein 2	10138	0.021183691918849945	0.3234479	No
**209389_x_at**	DBI	diazepam binding inhibitor (GABA receptor modulator, acyl-Coenzyme A binding protein)	10159	0.021035052835941315	0.32265288	No
**220234_at**	CA8	carbonic anhydrase VIII	10173	0.020970387384295464	0.32218945	No

**202410_x_at**	IGF2	insulin-like growth factor 2 (somatomedin A)	10178	0.020934714004397392	0.3221528	No
**208491_s_at**	PGM5	phosphoglucomutase 5	10191	0.02082817070186138	0.32173577	No
**202192_s_at**	GAS7	growth arrest-specific 7	10217	0.020719347521662712	0.32070118	No
**206745_at**	HOXC11	homeobox C11	10221	0.020700298249721527	0.32071024	No
**216758_at**	RAB33A	RAB33A, member RAS oncogene family	10244	0.020555544644594193	0.31981677	No
**216134_at**	FRMD4B	FERM domain containing 4B	10254	0.020489871501922607	0.31953964	No
**207106_s_at**	LTK	leukocyte tyrosine kinase	10257	0.020478103309869766	0.3195945	No
**207268_x_at**	ABI2	abl interactor 2	10286	0.020247986540198326	0.31841412	No
**220410_s_at**	CAMSAP 1	calmodulin regulated spectrin-associated protein 1	10312	0.020000627264380455	0.3173743	No
**204456_s_at**	GAS1	growth arrest-specific 1	10323	0.019932933151721954	0.31704563	No
**207342_at**	CNGB1	cyclic nucleotide gated channel beta 1	10343	0.0197895597666502	0.3162889	No
**219812_at**	PVRIG	poliovirus receptor related immunoglobulin domain containing	10372	0.019623562693595886	0.31510395	No
**215795_at**	MYH7B	myosin, heavy polypeptide 7B, cardiac muscle, beta	10405	0.019428357481956482	0.3137278	No
**206270_at**	PRKCG	protein kinase C, gamma	10408	0.01941685378551483	0.3137749	No
**206955_at**	AQP7	aquaporin 7	10451	0.01916755922138691	0.3119224	No
**221114_at**	AMBN	ameloblastin (enamel matrix protein)	10496	0.018846403807401657	0.30997267	No
**216148_at**	Sep-11	Septin 11	10512	0.01876448094844818	0.30939823	No
**210632_s_at**	SGCA	sarcoglycan, alpha (50kDa dystrophin-associated glycoprotein)	10518	0.01874689571559429	0.3092981	No
**207605_x_at**	ZNF117	zinc finger protein 117 (HPF9)	10549	0.018558425828814507	0.3080105	No
**211070_x_at**	DBI	enzyme A binding protein)	10550	0.018555626273155212	0.30814618	No
**210738_s_at**	SLC4A4	solute carrier family 4, sodium bicarbonate cotransporter, member 4	10560	0.018470721319317818	0.30785426	No
**206478_at**	KIAA012 5	KIAA0125	10593	0.01822872832417488	0.30646935	No
**207450_s_at**	POU6F2	POU domain, class 6, transcription factor 2	10620	0.018077000975608826	0.30536798	No
**211424_x_at**	METTL7 A	methyltransferase like 7A	10624	0.018039537593722343	0.30535758	No
**204681_s_at**	RAPGEF5	Rap guanine nucleotide exchange factor (GEF) 5	10647	0.017839636653661728	0.30444428	No
**221047_s_at**	MARK1	MAP/microtubule affinity-regulating kinase 1	10658	0.017806151881814003	0.30410004	No
**221112_at**	IL1RAPL 2	interleukin 1 receptor accessory protein-like 2	10684	0.01767987757921219	0.30304322	No
**201651_s_at**	PACSIN2	protein kinase C and casein kinase substrate in neurons 2	10711	0.01746818795800209	0.3019374	No
**217146_at**	JRK	jerky homolog (mouse)	10725	0.017361845821142197	0.3014476	No
**215271_at**	TNN	tenascin N	10744	0.017260337248444557	0.30071983	No
**215904_at**	MLLT4	myeloid/lymphoid or mixed-lineage leukemia (trithorax homolog, Drosophila); translocated to, 4	10874	0.01637425273656845	0.29471916	No

**216489_at**	TRPM3	transient receptor potential cation channel, subfamily M, member 3	10937	0.01598743349313736	0.2918945	No
**208416_s_at**	SPTB	spectrin, beta, erythrocytic (includes spherocytosis, clinical type I)	10958	0.015798037871718407	0.29106113	No
**220821_at**	GALR1	galanin receptor 1	10988	0.01565413922071457	0.28979972	No
**213633_at**	SH3BP1	SH3-domain binding protein 1	10991	0.01562411431223154	0.2898191	No
**208413_at**	RARB	Retinoic acid receptor, beta	11003	0.015572638250887394	0.2894111	No
**210036_s_at**	KCNH2	potassium voltage-gated channel, subfamily H (eag-related), member 2	11006	0.015562360174953938	0.28943002	No
**216147_at**	Sep-11	Septin 11	11016	0.015474226325750351	0.28911617	No
**208383_s_at**	PCK1	phosphoenolpyruvate carboxykinase 1 (soluble)	11047	0.01526274997740984	0.28780445	No
**208367_x_at**	CYP3A4	cytochrome P450, family 3, subfamily A, polypeptide 4	11076	0.015126502141356468	0.28658664	No
**217892_s_at**	LIMA1	LIM domain and actin binding 1	11087	0.015071691945195198	0.2862224	No
**210726_at**	CYP3A4	cytochrome P450, family 3, subfamily A, polypeptide 4	11105	0.014949347823858261	0.28552517	No
**215912_at**	GNAO1	guanine nucleotide binding protein (G protein), alpha activating activity polypeptide O	11131	0.014813216403126717	0.28444737	No
**211170_s_at**	PDE10A	phosphodiesterase 10A	11161	0.014618772082030773	0.2831784	No
**219804_at**	SYNPO2L	synaptopodin 2-like	11167	0.014593825675547123	0.2830479	No
**220780_at**	PLA2G3	phospholipase A2, group III	11190	0.014476564712822437	0.28210998	No
**212093_s_at**	MTUS1	mitochondrial tumor suppressor 1	11217	0.01429695449769497	0.28098097	No
**221359_at**	GDNF	glial cell derived neurotrophic factor	11224	0.01426398940384388	0.28080064	No
**220860_at**	PURG	purine-rich element binding protein G	11248	0.014127103611826897	0.27981272	No
**221162_at**	HHLA1	HERV-H LTR-associating 1	11286	0.013882198370993137	0.27815878	No
**221004_s_at**	ITM2C	integral membrane protein 2C /// integral membrane protein 2C	11295	0.013809094205498695	0.27788022	No
**220378_at**	TCP11	t-complex 11 (mouse)	11366	0.013360731303691864	0.27465677	No
**216642_at**	SEC14L1	SEC14-like 1 (S. cerevisiae)	11380	0.013289530761539936	0.27413717	No
**208408_at**	PTN	pleiotrophin (heparin binding growth factor 8, neurite growth-promoting factor 1)	11393	0.013187281787395477	0.2736643	No
**210883_x_at**	EFNB3	ephrin-B3	11403	0.013094275258481503	0.27333304	No
**208225_at**	FGFR2	ffer syndrome, Jackson-Weiss syndrome)	11412	0.013051682151854038	0.27304894	No
**211299_s_at**	FLOT2	flotillin 2	11431	0.012890171259641647	0.27228922	No
**205973_at**	FEZ1	fasciculation and elongation protein zeta 1 (zygin I)	11616	0.011515152640640736	0.26364353	No
**212841_s_at**	PPFIBP2	PTPRF interacting protein, binding protein 2 (liprin beta 2)	11619	0.011484823189675808	0.26363263	No
**209664_x_at**	NFATC1	nuclear factor of activated T-cells, cytoplasmic, calcineurin-dependent 1	11660	0.011200416833162308	0.26181674	No

**216367_at**	COL4A3	collagen, type IV, alpha 3 (Goodpasture antigen)	11674	0.011106622405350208	0.2612812	No
**217184_s_at**	LTK	leukocyte tyrosine kinase	11687	0.010996508412063122	0.2607923	No
**211510_s_at**	CRHR2	corticotropin releasing hormone receptor 2	11693	0.010963546112179756	0.26063523	No
**216137_s_at**	MAPK8IP 3	Mitogen-activated protein kinase 8 interacting protein 3	11733	0.01065627858042717	0.2588628	No
**212134_at**	PHLDB1	pleckstrin homology-like domain, family B, member 1	11791	0.010268443264067173	0.25623354	No
**204457_s_at**	GAS1	growth arrest-specific 1	11797	0.01021136250346899	0.256071	No
**215685_s_at**	DLX2	distal-less homeobox 2	11798	0.010209361091256142	0.25614566	No
**221401_at**	CACNG5	calcium channel, voltage-dependent, gamma subunit 5	11818	0.01010869350284338	0.25531814	No
**207676_at**	ONECUT 2	one cut domain, family member 2	11851	0.009903931058943272	0.25387233	No
**220886_at**	GABRQ	gamma-aminobutyric acid (GABA) receptor, theta	11892	0.009700438939034939	0.25204548	No
**219735_s_at**	TFCP2L1	transcription factor CP2-like 1	11906	0.009634390473365784	0.25149915	No
**220315_at**	PARP11	poly (ADP-ribose) polymerase family, member 11	11911	0.009624493308365345	0.25137976	No
**203662_s_at**	TMOD1	tropomodulin 1	11937	0.0094788558781147	0.25026295	No
**214646_at**	HIST1H3J	Histone 1, H3j	11959	0.00934150442481041	0.24933493	No
**219935_at**	ADAMTS 5	ADAM metallopeptidase with thrombospondin type 1 motif, 5 (aggrecanase-2)	11982	0.009160556830465794	0.24835813	No
**208271_at**	PAPOLB	poly(A) polymerase beta (testis specific)	11983	0.009160221554338932	0.24842513	No
**204311_at**	ATP1B2	ATPase, Na+/K+ transporting, beta 2 polypeptide	11994	0.009023511782288551	0.24801667	No
**205888_s_at**	JAKMIP2	janus kinase and microtubule interacting protein 2	12014	0.00890358816832304	0.24718033	No
**208601_s_at**	TUBB1	tubulin, beta 1 /// tubulin, beta 1	12027	0.008775628171861172	0.24667518	No
**212572_at**	STK38L	serine/threonine kinase 38 like	12037	0.00871847104281187	0.24631193	No
**211827_s_at**	KCND3	potassium voltage-gated channel, Shal-related subfamily, member 3	12045	0.008682196959853172	0.24604331	No
**208208_at**	MYH13	myosin, heavy polypeptide 13, skeletal muscle	12142	0.008053421042859554	0.2415475	No
**207454_at**	GRIK3	glutamate receptor, ionotropic, kainate 3	12185	0.007747315336018801	0.23961146	No
**215914_at**	DPP6	Dipeptidyl-peptidase 6	12239	0.00737507501617074	0.2371508	No
**204708_at**	MAPK4	mitogen-activated protein kinase 4	12257	0.007299037184566259	0.23639762	No
**207701_at**	C22ORF2 4	chromosome 22 open reading frame 24	12261	0.007250089664012194	0.2363083	No
**205860_x_at**	FOLH1	folate hydrolase (prostate-specific membrane antigen) 1	12274	0.007172096986323595	0.23579143	No
**206337_at**	CCR7	chemokine (C-C motif) receptor 7 /// chemokine (C-C motif) receptor 7	12294	0.0069995964877307415	0.23494115	No
**202519_at**	MLXIP	MLX interacting protein	12365	0.006422848906368017	0.23166698	No
**219041_s_at**	REPIN1	replication initiator 1	12387	0.0062902760691940784	0.23071663	No

**204141_at**	TUBB2A	tubulin, beta 2A	12391	0.00627571670338 5115	0.2306202	No
**211154_at**	THPO	thrombopoietin (myeloproliferative leukemia virus oncogene ligand, megakaryocyte growth and development factor)	12398	0.0062131392769515514	0.23038097	No
**204469_at**	PTPRZ1	protein tyrosine phosphatase, receptor-type, Z polypeptide 1	12424	0.006091001443564892	0.22923939	No
**207184_at**	SLC6A13	solute carrier family 6 (neurotransmitter transporter, GABA), member 13	12441	0.00599058298394084	0.22852409	No
**201494_at**	PRCP	prolylcarboxypeptidase (angiotensinase C)	12446	0.005960449110716581	0.2283779	No
**221835_at**	DTX3	deltex 3 homolog (Drosophila)	12495	0.0056373500265181065	0.22614177	No
**211315_s_at**	CACNA1 G	calcium channel, voltage-dependent, alpha 1G subunit	12509	0.005554113071411848	0.2255656	No
**219533_at**	CDKN1C	cyclin-dependent kinase inhibitor 1C (p57, Kip2)	12527	0.005440108478069305	0.22479881	No
**216901_s_at**	IKZF1	IKAROS family zinc finger 1 (Ikaros)	12537	0.005390054080635309	0.22441123	No
**220428_at**	CD207	CD207 molecule, langerin	12547	0.005330903455615044	0.22402321	No
**211011_at**	COL19A1	collagen, type XIX, alpha 1	12558	0.005234541371464729	0.22358705	No
**221557_s_at**	LEF1	lymphoid enhancer-binding factor 1	12575	0.005111456383019686	0.22286531	No
**202732_at**	PKIG	protein kinase (cAMP-dependent, catalytic) inhibitor gamma	12584	0.005010879598557949	0.2225224	No
**204486_at**	KCNQ1	potassium voltage-gated channel, KQT-like subfamily, member 1	12598	0.0048546576872467995	0.22194111	No
**207949_s_at**	ICA1	islet cell autoantigen 1, 69kDa	12610	0.004786042496562004	0.22145422	No
**205502_at**	CYP17A1	cytochrome P450, family 17, subfamily A, polypeptide 1	12642	0.004478018265217543	0.22001618	No
**217202_s_at**	GLUL	glutamate-ammonia ligase (glutamine synthetase)	12677	0.004219221416860819	0.2184339	No
**211301_at**	KCND3	potassium voltage-gated channel, Shal-related subfamily, member 3	12700	0.004086614120751619	0.21742	No
**203185_at**	RASSF2	Ras association (RalGDS/AF-6) domain family 2	12721	0.003979467321187258	0.2165002	No
**214297_at**	CSPG4	Chondroitin sulfate proteoglycan 4 (melanoma-associated)	12752	0.0037024738267064095	0.21510392	No
**206067_s_at**	WT1	Wilms tumor 1	12760	0.0036492443177849054	0.2147985	No
**201307_at**	Sep-11	septin 11	12789	0.0033828034065663815	0.21349478	No
**207777_s_at**	SP140	SP140 nuclear body protein	12792	0.003366639604791999	0.2134245	No
**215270_at**	LFNG	lunatic fringe homolog (Drosophila)	12867	0.0028536797035485506	0.20993444	No
**211904_x_at**	RAD52	RAD52 homolog (S. cerevisiae)	12873	0.0028287325985729694	0.20971791	No
**214191_at**	ICA1	islet cell autoantigen 1, 69kDa	12910	0.002571334596723318	0.20802869	No
**202084_s_at**	SEC14L1	SEC14-like 1 (S. cerevisiae)	12953	0.002245529321953654 3	0.20605242	No
**219554_at**	RHCG	Rh family, C glycoprotein	12959	0.0022284097503870726	0.2058315	No
**208256_at**	EFNA2	ephrin-A2	12982	0.002066739834845066	0.20480281	No

**217516_x_at**	ARVCF	armadillo repeat gene deletes in velocardiofacial syndrome	12988	0.002025523455813527	0.20458041	No
**218825_at**	EGFL7	EGF-like-domain, multiple 7	12998	0.0019434434361755848	0.20416762	No
**205993_s_at**	TBX2	T-box 2	13056	0.001488514943048358	0.20147413	No
**202428_x_at**	DBI	diazepam binding inhibitor (GABA receptor modulator, acyl-Coenzyme A binding protein)	13073	0.0013585996348410845	0.20072494	No
**214564_s_at**	PCDHGC 3	protocadherin gamma subfamily C, 3	13144	8,58E+11	0.19741006	No
**215322_at**	LONRF1	LON peptidase N-terminal domain and ring finger 1	13166	7,38E+11	0.19641912	No
**208657_s_at**	Sep-09	septin 9	13177	6,48E+11	0.1959494	No
**213050_at**	COBL	cordon-bleu homolog (mouse)	13272	-1,15E+12	0.19149041	No
**211384_s_at**	CASR	calcium-sensing receptor (hypocalciuric hypercalcemia 1, severe neonatal hyperparathyroidism)	13279	-1,42E+12	0.19120678	No
**210601_at**	CDH6	cadherin 6, type 2, K-cadherin (fetal kidney)	13318	-4,59E+12	0.18940721	No
**220502_s_at**	SLC13A1	solute carrier family 13 (sodium/sulfate symporters), member 1	13388	-9,92E+11	0.18614076	No
**214708_at**	SNTB1	syntrophin, beta 1 (dystrophin-associated protein A1, 59kDa, basic component 1)	13410	-0.0011911025503650308	0.18515313	No
**219693_at**	AGPAT4	1-acylglycerol-3-phosphate O-acyltransferase 4 (lysophosphatidic acid acyltransferase, delta)	13431	-0.0013542647939175367	0.18421413	No
**216993_s_at**	COL11A2	collagen, type XI, alpha 2	13454	-0.0016234813956543803	0.18318221	No
**206563_s_at**	OPRL1	opiate receptor-like 1	13487	-0.0019427123479545116	0.18167818	No
**218892_at**	DCHS1	dachsous 1 (Drosophila)	13535	-0.0022830679081380367	0.17946495	No
**206033_s_at**	DSC3	desmocollin 3	13555	-0.0024244440719485283	0.17858122	No
**214385_s_at**	MUC5AC	mucin 5AC, oligomeric mucus/gel-forming	13578	-0.0026312454137951136	0.17755668	No
**217505_at**	KLHL23	kelch-like 23 (Drosophila)	13582	-0.0026628952473402023	0.17743382	No
**211377_x_at**	MYCN	v-myc myelocytomatosis viral related oncogene, neuroblastoma derived (avian)	13593	-0.002733703004196286	0.17697936	No
**207761_s_at**	METTL7 A	methyltransferase like 7A	13646	-0.003171112621203065	0.17453541	No
**214368_at**	RASGRP2	RAS guanyl releasing protein 2 (calcium and DAG-regulated)	13652	-0.0032042593229562044	0.17432162	No
**205291_at**	IL2RB	interleukin 2 receptor, beta /// interleukin 2 receptor, beta	13658	-0.0032456726767122746	0.17410813	No
**216131_at**	FRMD4B	FERM domain containing 4B	13708	-0.0035891933366656303	0.17180957	No
**207234_at**	RFX3	regulatory factor X, 3 (influences HLA class II expression)	13756	-0.004068463575094938	0.16960941	No
**208406_s_at**	GRAP2	GRB2-related adaptor protein 2	13813	-0.004543493967503309	0.16698572	No
**221665_s_at**	EPS8L1	EPS8-like 1	13833	-0.004764922428876162	0.16611911	No

**219837_s_at**	CYTL1	cytokine-like 1	13857	-0.004981667269021273	0.1650643	No
**217112_at**	PDGFB	platelet-derived growth factor beta polypeptide (simian sarcoma viral (v-sis) oncogene homolog)	13862	-0.005016474984586239	0.16491121	No
**217404_s_at**	COL2A1	collagen, type II, alpha 1 (primary osteoarthritis, spondyloepiphyseal dysplasia, congenital)	13871	-0.005035871174186468	0.16456848	No
**217154_s_at**	EDN3	endothelin 3	13872	-0.005049763713032007	0.16460542	No
**200952_s_at**	CCND2	cyclin D2	13893	-0.005172996316105127	0.16369435	No
**210173_at**	PTPRJ	protein tyrosine phosphatase, receptor type, J	13899	-0.005256743635982275	0.16349557	No
**210872_x_at**	GAS7	growth arrest-specific 7	13916	-0.005442379973828793	0.16277625	No
**202825_at**	SLC25A4	solute carrier family 25 (mitochondrial carrier; adenine nucleotide translocator), member 4	13934	-0.005613507237285376	0.16201074	No
**200951_s_at**	CCND2	cyclin D2	13974	-0.00595773896202445	0.16020395	No
**206012_at**	LEFTY2	left-right determination factor 2	13998	-0.006148258689790964	0.15915768	No
**220563_s_at**	SHANK1	SH3 and multiple ankyrin repeat domains 1	14015	-0.00630180025473237	0.15844466	No
**215697_at**	RIMBP2	RIMS binding protein 2	14040	-0.006485729478299618	0.1573534	No
**220187_at**	STEAP4	STEAP family member 4	14046	-0.006555491127073765	0.15716413	No
**220704_at**	IKZF1	IKAROS family zinc finger 1 (Ikaros)	14059	-0.006634428631514311	0.1566433	No
**37022_at**	PRELP	proline/arginine-rich end leucine-rich repeat protein	14063	-0.0066811395809054375	0.15654983	No
**206802_at**	PAX5	paired box gene 5 (B-cell lineage specific activator)	14121	-0.007068458944559097	0.15389717	No
**214367_at**	RASGRP2	RAS guanyl releasing protein 2 (calcium and DAG-regulated)	14127	-0.007106251548975706	0.1537119	No
**218600_at**	LIMD2	LIM domain containing 2	14133	-0.0071358331479132175	0.15352687	No
**220354_at**	MCF2L	MCF.2 cell line derived transforming sequence-like	14144	-0.007303472142666578	0.15310584	No
**216736_at**	TM6SF2	transmembrane 6 superfamily member 2	14174	-0.007556362077593803	0.1517852	No
**210769_at**	CNGB1	cyclic nucleotide gated channel beta 1	14186	-0.0076764607802033424	0.15131944	No
**205977_s_at**	EPHA1	EPH receptor A1	14200	-0.007786486763507128	0.1507596	No
**204836_at**	GLDC	glycine dehydrogenase (decarboxylating)	14223	-0.008015912026166916	0.14977443	No
**208229_at**	FGFR2	ffer syndrome, Jackson-Weiss syndrome)	14246	-0.008168014697730541	0.14879037	No
**205784_x_at**	ARVCF	armadillo repeat gene deletes in velocardiofacial syndrome	14255	-0.008247883059084415	0.14847115	No
**91826_at**	EPS8L1	EPS8-like 1	14311	-0.008645705878734589	0.1459249	No
**211393_at**	PATZ1	POZ (BTB) and AT hook containing zinc finger 1	14373	-0.009283497929573059	0.14309864	No
**206815_at**	SPAG8	sperm associated antigen 8	14383	-0.009391753003001213	0.14274032	No
**216461_at**	C9ORF10 6	Chromosome 9 open reading frame 106	14421	-0.009750786237418652	0.14105617	No

**211217_s_at**	KCNQ1	potassium voltage-gated channel, KQT-like subfamily, member 1	14436	-0.009871860034763813	0.14046414	No
**203331_s_at**	INPP5D	inositol polyphosphate-5-phosphatase, 145kDa	14460	-0.010138281621038914	0.13944705	No
**208561_at**	ABCC9	ATP-binding cassette, sub-family C (CFTR/MRP), member 9	14496	-0.010512139648199081	0.13786335	No
**220628_s_at**	SDK2	sidekick homolog 2 (chicken)	14535	-0.010862245224416256	0.13613988	No
**211259_s_at**	BMP7	bone morphogenetic protein 7 (osteogenic protein 1)	14536	-0.010865644551813602	0.13621935	No
**207869_s_at**	CACNA1 G	calcium channel, voltage-dependent, alpha 1G subunit	14568	-0.011082066223025322	0.1348296	No
**204926_at**	INHBA	inhibin, beta A (activin A, activin AB alpha polypeptide)	14570	-0.011092150583863258	0.13486327	No
**210881_s_at**	IGF2	insulin-like growth factor 2 (somatomedin A)	14585	-0.011279028840363026	0.13428155	No
**202936_s_at**	SOX9	SRY (sex determining region Y)-box 9 (campomelic dysplasia, autosomal sex-reversal)	14605	-0.011483950540423393	0.13346407	No
**206901_at**	C19ORF5 7	chromosome 19 open reading frame 57	14632	-0.01181674376130104	0.13231692	No
**208978_at**	CRIP2	cysteine-rich protein 2	14662	-0.01208801381289959	0.13102943	No
**215638_at**	ERBB3	v-erb-b2 erythroblastic leukemia viral oncogene homolog 3 (avian)	14695	-0.012305566109716892	0.12960118	No
**205117_at**	FGF1	fibroblast growth factor 1 (acidic)	14705	-0.01241402979940176	0.12926497	No
**219535_at**	HUNK	hormonally upregulated Neu-associated kinase	14729	-0.012644654139876366	0.1282662	No
**205533_s_at**	CDH6	cadherin 6, type 2, K-cadherin (fetal kidney)	14744	-0.01274685375392437	0.1276952	No
**203070_at**	SEMA3B	sema domain, immunoglobulin domain (Ig), short basic domain, secreted, (semaphorin) 3B	14758	-0.012885785661637783	0.12717266	No
**216898_s_at**	COL4A3	collagen, type IV, alpha 3 (Goodpasture antigen)	14793	-0.013214098289608955	0.12565617	No
**211422_at**	TRPM3	transient receptor potential cation channel, subfamily M, member 3	14814	-0.013377570547163486	0.12480511	No
**213947_s_at**	NUP210	nucleoporin 210kDa	14817	-0.013429706916213036	0.12480844	No
**222172_at**	NPAS3	neuronal PAS domain protein 3	14828	-0.013556758873164654	0.12443314	No
**210248_at**	WNT7A	wingless-type MMTV integration site family, member 7A	14849	-0.01379227265715599	0.123585105	No
**214934_at**	ATP9B	ATPase, Class II, type 9B	14871	-0.01401227805763483	0.12269124	No
**207034_s_at**	GLI2	GLI-Kruppel family member GLI2	14874	-0.014065327122807503	0.122699216	No
**210245_at**	ABCC8	ATP-binding cassette, sub-family C (CFTR/MRP), member 8	14883	-0.014141222462058067	0.12242308	No
**205758_at**	CD8A	CD8a molecule /// CD8a molecule	14968	-0.014807576313614845	0.118545994	No
**208058_s_at**	MGAT3	mannosyl (beta-1,4-)-glycoprotein beta-1,4-N-acetylglucosaminyltransferase	14996	-0.015015484765172005	0.11737479	No

**208084_at**	ITGB6	integrin, beta 6	15004	-0.015107526443898678	0.11715317	No
**210888_s_at**	ITIH1	inter-alpha (globulin) inhibitor H1	15018	-0.015246054157614708	0.116647884	No
**209841_s_at**	LRRN3	leucine rich repeat neuronal 3	15054	-0.015623630955815315	0.115101576	No
**215177_s_at**	ITGA6	integrin, alpha 6	15184	-0.016984574496746063	0.10910538	No
**220102_at**	FOXL2	forkhead box L2	15237	-0.01752251386642456	0.10676639	No
**206718_at**	LMO1	LIM domain only 1 (rhombotin 1)	15242	-0.017616305500268936	0.10670544	No
**210654_at**	TNFRSF10D	tumor necrosis factor receptor superfamily, member 10d, decoy with truncated death domain	15260	-0.017946476116776466	0.10603014	No
**206377_at**	FOXF2	forkhead box F2	15274	-0.01813117414712906	0.10554595	No
**41220_at**	Sep-09	septin 9	15283	-0.018195172771811485	0.105299465	No
**210090_at**	ARC	activity-regulated cytoskeleton-associated protein	15292	-0.018272479996085167	0.105053544	No
**205222_at**	EHHADH	enoyl-Coenzyme A, hydratase/3-hydroxyacyl Coenzyme A dehydrogenase	15318	-0.018542427569627762	0.10400303	No
**209840_s_at**	LRRN3	leucine rich repeat neuronal 3	15389	-0.01941380277276039	0.10082386	No
**210846_x_at**	TRIM14	tripartite motif-containing 14	15414	-0.01957448571920395	0.09982834	No
**49051_g_at**	DTX3	deltex 3 homolog (Drosophila)	15466	-0.02014196291565895	0.09755595	No
**204503_at**	EVPL	envoplakin	15539	-0.0211216527968645 1	0.09429438	No
**213430_at**	RUFY3	RUN and FYVE domain containing 3	15552	-0.02123330347239971	0.09388033	No
**215431_at**	SNTB1	syntrophin, beta 1 (dystrophin-associated protein A1, 59kDa, basic component 1)	15587	-0.021715575829148293	0.09242602	No
**216630_at**	SEC14L1	SEC14-like 1 (S. cerevisiae)	15654	-0.02259627915918827	0.0894599	No
**214303_x_at**	MUC5AC	mucin 5AC, oligomeric mucus/gel-forming	15668	-0.022761259227991104	0.08900958	No
**208240_s_at**	FGF1	fibroblast growth factor 1 (acidic)	15678	-0.02288532257080078	0.08874995	No
**201350_at**	FLOT2	flotillin 2	15687	-0.022978011518716812	0.088538446	No
**206857_s_at**	FKBP1B	FK506 binding protein 1B, 12.6 kDa	15700	-0.023104103282094002	0.08813808	No
**209686_at**	S100B	S100 calcium binding protein, beta (neural)	15711	-0.02331898733973503	0.08783418	No
**205363_at**	BBOX1	butyrobetaine (gamma), 2-oxoglutarate dioxygenase (gamma-butyrobetaine hydroxylase) 1	15718	-0.023401379585266113	0.087720655	No
**201565_s_at**	ID2	inhibitor of DNA binding 2, dominant negative helix-loop-helix protein	15728	-0.02347574196755886	0.087465346	No
**219755_at**	CBX8	chromobox homolog 8 (Pc class homolog, Drosophila)	15766	-0.02403545007109642	0.08588567	No
**219407_s_at**	LAMC3	laminin, gamma 3	15874	-0.02526233345270157	0.0809938	No
**219550_at**	ROBO3	roundabout, axon guidance receptor, homolog 3 (Drosophila)	15884	-0.025369198992848396	0.080752335	No

**208057_s_at**	GLI2	GLI-Kruppel family member GLI2	15913	-0.02591477707028389	0.0796134	No
**210263_at**	KCNF1	potassium voltage-gated channel, subfamily F, member 1	15925	-0.026103463023900986	0.07928242	No
**212096_s_at**	MTUS1	mitochondrial tumor suppressor 1	15976	-0.02672864869236946	0.07710565	No
**208512_s_at**	MLLT4	myeloid/lymphoid or mixed-lineage leukemia (trithorax homolog, Drosophila); translocated to, 4	16001	-0.027029355987906456	0.07616465	No
**208921_s_at**	SRI	sorcin	16017	-0.02723103202879429	0.07565213	No
**218660_at**	DYSF	dysferlin, limb girdle muscular dystrophy 2B (autosomal recessive)	16046	-0.027623793110251427	0.0745257	No
**214538_x_at**	RGS6	regulator of G-protein signalling 6	16054	-0.02772645093500614	0.074396364	No
**207143_at**	CDK6	cyclin-dependent kinase 6	16082	-0.028105702251195908	0.0733209	No
**206712_at**	GRTP1	growth hormone regulated TBC protein 1	16109	-0.02845410630106926	0.072295435	No
**206564_at**	OPRL1	opiate receptor-like 1	16164	-0.029213951900601387	0.069947064	No
**206258_at**	ST8SIA5	ST8 alpha-N-acetyl-neuraminide alpha-2,8-sialyltransferase 5	16171	-0.029369382187724113	0.06987719	No
**209186_at**	ATP2A2	ATPase, Ca++ transporting, cardiac muscle, slow twitch 2	16193	-0.029662229120731354	0.06909778	No
**209734_at**	NCKAP1L	NCK-associated protein 1-like	16212	-0.029826613143086433	0.06846191	No
**203332_s_at**	INPP5D	inositol polyphosphate-5-phosphatase, 145kDa	16264	-0.030525315552949905	0.066265464	No
**206206_at**	CD180	CD180 molecule	16267	-0.030560022220015526	0.066394076	No
**219634_at**	CHST11	carbohydrate (chondroitin 4) sulfotransferase 11	16325	-0.03125624731183052	0.06391831	No
**207583_at**	ABCD2	ATP-binding cassette, sub-family D (ALD), member 2	16340	-0.03142266720533371	0.06348389	No
**212862_at**	CDS2	CDP-diacylglycerol synthase (phosphatidate cytidylyltransferase) 2	16384	-0.032091300934553146	0.061678454	No
**212095_s_at**	MTUS1	mitochondrial tumor suppressor 1	16390	-0.03212795406579971	0.061676204	No
**206338_at**	ELAVL3	ELAV (embryonic lethal, abnormal vision, Drosophila)-like 3 (Hu antigen C)	16403	-0.032305728644132614	0.061343133	No
**210162_s_at**	NFATC1	nuclear factor of activated T-cells, cytoplasmic, calcineurin-dependent 1	16450	-0.03291287273168564	0.059401374	No
**216617_s_at**	MAG	myelin associated glycoprotein	16499	-0.03344804421067238	0.057368636	No
**220299_at**	SPATA6	spermatogenesis associated 6	16528	-0.033907677978277206	0.056288164	No
**207558_s_at**	PITX2	paired-like homeodomain transcription factor 2	16533	-0.033973727375268936	0.056346856	No
**209695_at**	PTP4A3	protein tyrosine phosphatase type IVA, member 3	16539	-0.03401905298233032	0.056358434	No
**205127_at**	PTGS1	prostaglandin-endoperoxide synthase 1 (prostaglandin G/H synthase and cyclooxygenase)	16545	-0.03404879942536354	0.05637023	No
**213946_s_at**	OBSL1	obscurin-like 1	16562	-0.03419476002454758	0.055861197	No

**205926_at**	IL27RA	interleukin 27 receptor, alpha	16667	-0.03589439392089844	0.051189426	No
**220493_at**	DMRT1	doublesex and mab-3 related transcription factor 1	16671	-0.03595338761806488	0.05131004	No
**219474_at**	C3ORF52	chromosome 3 open reading frame 52	16700	-0.036290112882852554	0.05024699	No
**206194_at**	HOXC4	homeobox C4	16705	-0.036315832287073135	0.050322812	No
**217182_at**	MUC5AC	mucin 5AC, oligomeric mucus/gel-forming	16715	-0.036519162356853485	0.050162897	No
**206816_s_at**	SPAG8	sperm associated antigen 8	16739	-0.036820683628320694	0.049340952	No
**210948_s_at**	LEF1	lymphoid enhancer-binding factor 1	16765	-0.037264540791511536	0.048427366	No
**212825_at**	PAXIP1	PAX interacting (with transcription-activation domain) protein 1	16844	-0.03819118067622185	0.045005966	No
**219123_at**	ZNF232	zinc finger protein 232	16861	-0.038373980671167374	0.044527497	No
**205418_at**	FES	feline sarcoma oncogene	16925	-0.039330627769231796	0.041826107	No
**38149_at**	ARHGAP 25	Rho GTPase activating protein 25	16943	-0.039642464369535446	0.041309472	No
**213435_at**	SATB2	SATB family member 2	16994	-0.04047943279147148	0.03923327	No
**209703_x_at**	METTL7 A	methyltransferase like 7A	17004	-0.04066823795437813	0.039103698	No
**207074_s_at**	SLC18A1	solute carrier family 18 (vesicular monoamine), member 1	17011	-0.0408400259912014	0.039117716	No
**202595_s_at**	LEPROTL 1	leptin receptor overlapping transcript-like 1	17013	-0.04084492102265358	0.039369	No
**218686_s_at**	RHBDF1	rhomboid 5 homolog 1 (Drosophila)	17028	-0.04109835624694824	0.039005347	No
**215591_at**	SATB2	SATB family member 2	17101	-0.042416565120220184	0.03589952	No
**221128_at**	ADAM19	ADAM metallopeptidase domain 19 (meltrin beta)	17122	-0.04275713115930557	0.03526333	No
**221693_s_at**	MRPS18A	mitochondrial ribosomal protein S18A /// mitochondrial ribosomal protein S18A	17139	-0.04305204749107361	0.034819074	No
**207704_s_at**	GAS7	growth arrest-specific 7	17150	-0.04325054958462715	0.034660943	No
**208291_s_at**	TH	tyrosine hydroxylase	17153	-0.04327628016471863	0.03488256	No
**205558_at**	TRAF6	TNF receptor-associated factor 6	17159	-0.04331259801983833	0.034962106	No
**205001_s_at**	DDX3Y	DEAD (Asp-Glu-Ala-Asp) box polypeptide 3, Y-linked	17195	-0.04392777010798454	0.0336228	No
**211399_at**	FGFR2	ffer syndrome, Jackson-Weiss syndrome)	17198	-0.04395699501037598	0.0338494	No
**209769_s_at**	GP1BB	glycoprotein Ib (platelet), beta polypeptide	17214	-0.044238585978746414	0.03346127	No
**215319_at**	ATP8B3	ATPase, Class I, type 8B, member 3	17221	-0.04435522109270096	0.033500995	No
**215001_s_at**	GLUL	glutamate-ammonia ligase (glutamine synthetase)	17251	-0.04479121044278145	0.032452676	No
**204736_s_at**	CSPG4	chondroitin sulfate proteoglycan 4 (melanoma-associated)	17256	-0.0448906272649765	0.03259121	No
**206120_at**	CD33	CD33 molecule	17263	-0.044992439448833466	0.0326356	No
**205000_at**	DDX3Y	DEAD (Asp-Glu-Ala-Asp) box polypeptide 3, Y-linked	17281	-0.045306604355573654	0.032160386	No

**219625_s_at**	COL4A3BP	collagen, type IV, alpha 3 (Goodpasture antigen) binding protein	17309	-0.04586035758256912	0.031214777	No
**202935_s_at**	SOX9	SRY (sex determining region Y)-box 9 (campomelic dysplasia, autosomal sex-reversal)	17311	-0.045873865485191345	0.03150284	No
**208372_s_at**	LIMK1	LIM domain kinase 1	17330	-0.046111930161714554	0.030986072	No
**204707_s_at**	MAPK4	mitogen-activated protein kinase 4	17372	-0.04696308821439743	0.029384296	No
**213395_at**	MLC1	megalencephalic leukoencephalopathy with subcortical cysts 1	17427	-0.0479620099067688	0.027173039	No
**204200_s_at**	PDGFB	platelet-derived growth factor beta polypeptide (simian sarcoma viral (v-sis) oncogene homolog)	17573	-0.050849977880716324	0.0206654	No
**211300_s_at**	TP53	tumor protein p53 (Li-Fraumeni syndrome)	17617	-0.05165997892618179	0.019003084	No
**206219_s_at**	VAV1	vav 1 oncogene	17635	-0.05205920711159706	0.01857726	No
**207345_at**	FST	follistatin	17646	-0.05224913731217384	0.018484943	No
**202479_s_at**	TRIB2	tribbles homolog 2 (Drosophila)	17760	-0.054241959005594254	0.013520354	No
**210511_s_at**	INHBA	inhibin, beta A (activin A, activin AB alpha polypeptide)	17876	-0.05675263702869415	0.0084792385	No
**207425_s_at**	Sep-09	septin 9	17900	-0.05720384418964386	0.0078063705	No
**218872_at**	TESC	tescalcin	18014	-0.059365205466747284	0.0028792522	No
**222240_s_at**	ISYNA1	myo-inositol 1-phosphate synthase A1	18018	-0.05951191857457161	0.0031721662	No
**200648_s_at**	GLUL	glutamate-ammonia ligase (glutamine synthetase)	18064	-0.060373105108737946	0.0014786851	No
**222272_x_at**	SCIN	scinderin	18076	-0.06068722531199455	0.0014006342	No
**222062_at**	IL27RA	interleukin 27 receptor, alpha	18088	-0.06091685593128204	0.0013242628	No
**218959_at**	HOXC10	homeobox C10	18095	-0.06101223826408386	0.0014858143	No
**219372_at**	IFT81	intraflagellar transport 81 homolog (Chlamydomonas)	18096	-0.061014484614133835	0.0019320528	No
**220298_s_at**	SPATA6	spermatogenesis associated 6	18129	-0.06182490661740303	8,66E+02	No
**206765_at**	KCNJ2	potassium inwardly-rectifying channel, subfamily J, member 2	18178	-0.062881238758564	-9,51E+03	No
**204882_at**	ARHGAP25	Rho GTPase activating protein 25	18254	-0.06508447974920273	-0.0040338733	No
**210602_s_at**	CDH6	cadherin 6, type 2, K-cadherin (fetal kidney)	18304	-0.06602354347705841	-0.005875809	No
**206893_at**	SALL1	sal-like 1 (Drosophila)	18306	-0.06607918441295624	-0.0054399744	No
**210152_at**	LILRB4	leukocyte immunoglobulin-like receptor, subfamily B (with TM and ITIM domains), member 4	18327	-0.06657163798809052	-0.0059019946	No
**204192_at**	CD37	CD37 molecule	18365	-0.0675717443227768	-0.0071632667	No
**210170_at**	PDLIM3	PDZ and LIM domain 3	18376	-0.06779727339744568	-0.007141872	No
**212873_at**	HMHA1	histocompatibility (minor) HA-1	18423	-0.06908021867275238	-0.008819117	No
**211067_s_at**	GAS7	growth arrest-specific 7 /// growth arrest-specific 7	18429	-0.06924574822187424	-0.0085499035	No

**219656_at**	PCDH12	protocadherin 12	18491	-0.07093678414821625	-0.010925247	No
**210270_at**	RGS6	regulator of G-protein signalling 6	18498	-0.07109648734331131	-0.010689943	No
**206578_at**	NKX2-5	NK2 transcription factor related, locus 5 (Drosophila)	18522	-0.07172869145870209	-0.011256581	No
**40560_at**	TBX2	T-box 2	18640	-0.07469414919614792	-0.016261369	No
**202478_at**	TRIB2	tribbles homolog 2 (Drosophila)	18655	-0.07531750202178955	-0.016374756	No
**221967_at**	NXPH4	neurexophilin 4	18667	-0.07560074329376221	-0.016343733	No
**204487_s_at**	KCNQ1	potassium voltage-gated channel, KQT-like subfamily, member 1	18671	-0.07571398466825485	-0.015932323	No
**213437_at**	RUFY3	RUN and FYVE domain containing 3	18691	-0.07651538401842117	-0.016274173	No
**218385_at**	MRPS18A	mitochondrial ribosomal protein S18A	18729	-0.0773368775844574	-0.017464027	No
**219359_at**	ATHL1	ATH1, acid trehalase-like 1 (yeast)	18738	-0.07763466238975525	-0.017275795	No
**206847_s_at**	HOXA7	homeobox A7	18762	-0.07838444411754608	-0.017793756	No
**211448_s_at**	RGS6	regulator of G-protein signalling 6	18766	-0.0785321444272995	-0.017361734	No
**204776_at**	THBS4	thrombospondin 4	18775	-0.07885324954986572	-0.01716459	No
**212776_s_at**	OBSL1	obscurin-like 1	18856	-0.0815117359161377	-0.020364048	No
**212813_at**	JAM3	junctional adhesion molecule 3	18859	-0.08164804428815842	-0.019861793	No
**218885_s_at**	GALNT12	UDP-N-acetyl-alpha-D-galactosamine:polypeptide N-acetylgalactosaminyltransferase 12 (GalNAc-T12)	18886	-0.08221110701560974	-0.020494102	No
**216733_s_at**	GATM	glycine amidinotransferase (L-arginine:glycine amidinotransferase)	18909	-0.08268314599990845	-0.02093318	No
**207104_x_at**	LILRB1	leukocyte immunoglobulin-like receptor, subfamily B (with TM and ITIM domains), member 1	18932	-0.08321784436702728	-0.021368345	No
**218870_at**	ARHGAP 15	Rho GTPase activating protein 15	18963	-0.08392686396837234	-0.022177886	No
**202421_at**	IGSF3	immunoglobulin superfamily, member 3	18964	-0.08396394550800323	-0.021563802	No
**209765_at**	ADAM19	ADAM metallopeptidase domain 19 (meltrin beta)	18977	-0.08442201465368271	-0.02151571	No
**206896_s_at**	GNG7	guanine nucleotide binding protein (G protein), gamma 7	19041	-0.08636459708213806	-0.023873111	No
**204724_s_at**	COL9A3	collagen, type IX, alpha 3	19054	-0.08670848608016968	-0.023808297	No
**209466_x_at**	PTN	pleiotrophin (heparin binding growth factor 8, neurite growth-promoting factor 1)	19117	-0.08817065507173538	-0.026105043	No
**219797_at**	MGAT4A	mannosyl (alpha-1,3-)-glycoprotein beta-1,4-N-acetylglucosaminyltransferase, isozyme A	19134	-0.08862249553203583	-0.02621601	No
**211200_s_at**	EFCAB2	EF-hand calcium binding domain 2	19179	-0.08993273973464966	-0.027645858	No
**205932_s_at**	MSX1	msh homeobox homolog 1 (Drosophila)	19196	-0.0904756411910057	-0.02774327	No
**205586_x_at**	VGF	VGF nerve growth factor inducible	19206	-0.0907052606344223	-0.027506892	No

**203147_s_at**	TRIM14	tripartite motif-containing 14	19210	-0.09092284739017487	-0.026984248	No
**213417_at**	TBX2	T-box 2	19249	-0.0918646901845932	-0.028115295	No
**211336_x_at**	LILRB1	leukocyte immunoglobulin-like receptor, subfamily B (with TM and ITIM domains), member 1	19261	-0.0922267809510231	-0.027962677	No
**221581_s_at**	LAT2	linker for activation of T cells family, member 2	19314	-0.09407646954059601	-0.029741779	No
**201656_at**	ITGA6	integrin, alpha 6	19328	-0.09447818249464035	-0.029667584	No
**218898_at**	FAM57A	family with sequence similarity 57, member A	19334	-0.09466541558504105	-0.029212462	No
**203071_at**	SEMA3B	sema domain, immunoglobulin domain (Ig), short basic domain, secreted, (semaphorin) 3B	19346	-0.09514255821704865	-0.029038517	No
**216867_s_at**	PDGFA	platelet-derived growth factor alpha polypeptide	19364	-0.0960480198264122	-0.029142622	No
**211737_x_at**	PTN	omoting factor 1)	19374	-0.09634647518396378	-0.028864983	No
**205304_s_at**	KCNJ8	potassium inwardly-rectifying channel, subfamily J, member 8	19394	-0.09717865288257599	-0.02905571	No
**205532_s_at**	CDH6	cadherin 6, type 2, K-cadherin (fetal kidney)	19421	-0.0985725000500679	-0.029568357	No
**207060_at**	EN2	engrailed homolog 2	19500	-0.10193899273872375	-0.032523528	No
**203441_s_at**	CDH2	cadherin 2, type 1, N-cadherin (neuronal)	19564	-0.10409697145223618	-0.03475124	No
**209185_s_at**	IRS2	insulin receptor substrate 2	19636	-0.10712805390357971	-0.037336346	No
**206574_s_at**	PTP4A3	protein tyrosine phosphatase type IVA, member 3	19656	-0.10809318721294403	-0.037447244	No
**208527_x_at**	HIST1H2 BE	histone 1, H2be	19693	-0.10942599922418594	-0.038354963	No
**203440_at**	CDH2	cadherin 2, type 1, N-cadherin (neuronal)	19742	-0.11136201024055481	-0.039817866	No
**201746_at**	TP53	tumor protein p53 (Li-Fraumeni syndrome)	19756	-0.11191345751285553	-0.039616156	No
**219815_at**	GAL3ST4	galactose-3-O-sulfotransferase 4	19764	-0.11214873939752579	-0.039128054	No
**202082_s_at**	SEC14L1	SEC14-like 1 (S. cerevisiae)	19790	-0.11312928050756454	-0.039486796	No
**210345_s_at**	DNAH9	dynein, axonemal, heavy polypeptide 9	19799	-0.11341086775064468	-0.039036907	No
**207327_at**	EYA4	eyes absent homolog 4 (Drosophila)	19829	-0.11456075310707092	-0.03957496	No
**205858_at**	NGFR	nerve growth factor receptor (TNFR superfamily, member 16)	19846	-0.11545581370592117	-0.039489675	No
**205270_s_at**	LCP2	lymphocyte cytosolic protein 2 (SH2 domain containing leukocyte protein of 76kDa)	19864	-0.11587949842214584	-0.03944874	No
**205303_at**	KCNJ8	potassium inwardly-rectifying channel, subfamily J, member 8	19876	-0.11651334911584854	-0.0391185	No
**204201_s_at**	PTPN13	protein tyrosine phosphatase, non-receptor type 13 (APO-1/CD95 (Fas)-associated phosphatase)	19905	-0.11784041672945023	-0.039585117	No
**219403_s_at**	HPSE	heparanase	19917	-0.118352971971035	-0.039241422	No
**204336_s_at**	RGS19	regulator of G-protein signalling 19	19937	-0.11912371218204498	-0.03927165	No

**218847_at**	IGF2BP2	insulin-like growth factor 2 mRNA binding protein 2	19959	-0.12046508491039276	-0.039386958	No
**218326_s_at**	LGR4	leucine-rich repeat-containing G protein-coupled receptor 4	19973	-0.12095335870981216	-0.039119132	No
**202083_s_at**	SEC14L1	SEC14-like 1 (S. cerevisiae)	20097	-0.12645989656448364	-0.044029996	No
**209474_s_at**	ENTPD1	ectonucleoside triphosphate diphosphohydrolase 1	20102	-0.1267687976360321	-0.043292634	No
**206071_s_at**	EPHA3	EPH receptor A3	20108	-0.12693871557712555	-0.042601474	No
**207691_x_at**	ENTPD1	ectonucleoside triphosphate diphosphohydrolase 1	20115	-0.12747140228748322	-0.04195386	No
**205406_s_at**	SPA17	sperm autoantigenic protein 17	20133	-0.12831518054008484	-0.04182198	No
**212775_at**	OBSL1	obscurin-like 1	20157	-0.1295546442270279	-0.041965697	No
**217165_x_at**	MT1F	metallothionein 1F (functional)	20210	-0.13202565908432007	-0.043467253	No
**219683_at**	FZD3	frizzled homolog 3 (Drosophila)	20237	-0.13350097835063934	-0.043724444	No
**53991_at**	DENND2 A	DENN/MADD domain containing 2A	20254	-0.13418114185333252	-0.04350221	No
**208808_s_at**	HMGB2	high-mobility group box 2	20293	-0.13581162691116333	-0.044311848	No
**221886_at**	DENND2 A	DENN/MADD domain containing 2A	20337	-0.13823507726192474	-0.045340985	No
**204948_s_at**	FST	follistatin	20368	-0.14013490080833435	-0.04573944	No
**213629_x_at**	MT1F	metallothionein 1F (functional)	20378	-0.14057587087154388	-0.04513832	No
**220291_at**	GDPD2	glycerophosphodiester phosphodiesterase domain containing 2	20381	-0.14072678983211517	-0.044203985	No
**201069_at**	MMP2	matrix metallopeptidase 2 (gelatinase A, 72kDa gelatinase, 72kDa type IV collagenase)	20384	-0.1408800333738327	-0.043268528	No
**203178_at**	GATM	glycine amidinotransferase (L-arginine:glycine amidinotransferase)	20435	-0.14344793558120728	-0.044591654	No
**214755_at**	UAP1L1	UDP-N-acteylglucosamine pyrophosphorylase 1-like 1	20455	-0.14471569657325745	-0.04443471	No
**219522_at**	FJX1	four jointed box 1 (Drosophila)	20468	-0.14583736658096313	-0.04393745	No
**216336_x_at**	MT1M	metallothionein 1M	20487	-0.14678172767162323	-0.04371795	No
**213138_at**	ARID5A	AT rich interactive domain 5A (MRF1-like)	20492	-0.14702863991260529	-0.042832416	No
**209443_at**	SERPINA 5	serpin peptidase inhibitor, clade A (alpha-1 antiproteinase, antitrypsin), member 5	20507	-0.14772899448871613	-0.042416207	No
**221885_at**	DENND2 A	DENN/MADD domain containing 2A	20554	-0.150554358959198	-0.04349758	No
**209184_s_at**	IRS2	insulin receptor substrate 2	20589	-0.15302003920078278	-0.04399158	No
**211792_s_at**	CDKN2C	cyclin-dependent kinase inhibitor 2C (p18, inhibits CDK4)	20669	-0.15916968882083893	-0.04657563	No
**207959_s_at**	DNAH9	dynein, axonemal, heavy polypeptide 9	20695	-0.1612715721130371	-0.046582274	No
**204357_s_at**	LIMK1	LIM domain kinase 1	20761	-0.16617998480796814	-0.04845082	No
**204345_at**	COL16A1	collagen, type XVI, alpha 1	20771	-0.16740724444389343	-0.04765347	No

**209465_x_at**	PTN	pleiotrophin (heparin binding growth factor 8, neurite growth-promoting factor 1)	20791	-0.16926904022693634	-0.047316954	No
**205186_at**	DNALI1	dynein, axonemal, light intermediate polypeptide 1	20797	-0.16966691613197327	-0.046313293	No
**202877_s_at**	CD93	CD93 molecule /// CD93 molecule	20926	-0.179648295044899	-0.05107238	No
**216967_at**	GAP43	growth associated protein 43	20954	-0.18246300518512726	-0.051018927	No
**203144_s_at**	KIAA0040	KIAA0040	20973	-0.18435053527355194	-0.050524663	No
**215248_at**	GRB10	growth factor receptor-bound protein 10	21031	-0.19003556668758392	-0.05183918	No
**215706_x_at**	ZYX	zyxin	21032	-0.19037024676799774	-0.05044688	No
**205918_at**	SLC4A3	solute carrier family 4, anion exchanger, member 3	21034	-0.19062155485153198	-0.049100183	No
**213056_at**	FRMD4B	FERM domain containing 4B	21035	-0.1908007115125656	-0.047704734	No
**207107_at**	RPE65	retinal pigment epithelium-specific protein 65kDa	21103	-0.20029199123382568	-0.04941869	No
**204159_at**	CDKN2C	cyclin-dependent kinase inhibitor 2C (p18, inhibits CDK4)	21206	-0.2111043483018875	-0.052714147	No
**207144_s_at**	CITED1	Cbp/p300-interacting transactivator, with Glu/Asp-rich carboxy-terminal domain, 1	21276	-0.22063599526882172	-0.054374203	No
**202350_s_at**	MATN2	matrilin 2	21300	-0.22470766305923462	-0.053822007	No
**205128_x_at**	PTGS1	prostaglandin-endoperoxide synthase 1 (prostaglandin G/H synthase and cyclooxygenase)	21315	-0.2269819676876068	-0.052826174	No
**209410_s_at**	GRB10	growth factor receptor-bound protein 10	21351	-0.23378048837184906	-0.052776963	No
**215813_s_at**	PTGS1	prostaglandin-endoperoxide synthase 1 (prostaglandin G/H synthase and cyclooxygenase)	21373	-0.23628070950508118	-0.052045234	No
**203799_at**	CD302	CD302 molecule	21423	-0.24472856521606445	-0.052580185	No
**212558_at**	SPRY1	sprouty homolog 1, antagonist of FGF signaling (Drosophila)	21448	-0.2492828518152237	-0.0518957	No
**204677_at**	CDH5	cadherin 5, type 2, VE-cadherin (vascular epithelium)	21508	-0.25913000106811523	-0.052799772	No
**218731_s_at**	VWA1	von Willebrand factor A domain containing 1	21538	-0.26351574063301086	-0.05224842	No
**205030_at**	FABP7	fatty acid binding protein 7, brain	21545	-0.26499876379966736	-0.05059498	No
**220559_at**	EN1	engrailed homolog 1	21546	-0.26526159048080444	-0.04865495	No
**219331_s_at**	KLHDC8A	kelch domain containing 8A	21554	-0.26660335063934326	-0.047037225	No
**205269_at**	LCP2	lymphocyte cytosolic protein 2 (SH2 domain containing leukocyte protein of 76kDa)	21564	-0.26835620403289795	-0.045501567	No
**206432_at**	HAS2	hyaluronan synthase 2	21671	-0.29473453760147095	-0.048375163	No
**216963_s_at**	GAP43	growth associated protein 43	21699	-0.29965510964393616	-0.047464605	No
**203143_s_at**	KIAA0040	KIAA0040	21715	-0.30481067299842834	-0.045947004	No

**203148_s_at**	TRIM14	tripartite motif-containing 14	21717	-0.30513760447502136	-0.04376278	No
**205374_at**	SLN	sarcolipin	21727	-0.3076360523700714	-0.041939843	No
**204471_at**	GAP43	growth associated protein 43	21728	-0.3077133595943451	-0.039689336	No
**206070_s_at**	EPHA3	EPH receptor A3	21749	-0.3153459131717682	-0.03833191	No
**209409_at**	GRB10	growth factor receptor-bound protein 10	21783	-0.3254410922527313	-0.037517436	No
**200808_s_at**	ZYX	zyxin	21790	-0.32712322473526	-0.035409644	No
**206580_s_at**	EFEMP2	EGF-containing fibulin-like extracellular matrix protein 2	21812	-0.33359429240226746	-0.0339662	No
**217901_at**	DSG2	Desmoglein 2	21847	-0.3469637930393219	-0.03304176	No
**207261_at**	CNGA3	cyclic nucleotide gated channel alpha 3	21853	-0.34879758954048157	-0.030728003	No
**209651_at**	TGFB1I1	transforming growth factor beta 1 induced transcript 1	21854	-0.34945765137672424	-0.028172191	No
**203698_s_at**	FRZB	frizzled-related protein	21856	-0.34991252422332764	-0.0256605	No
**205463_s_at**	PDGFA	platelet-derived growth factor alpha polypeptide	21866	-0.3517414927482605	-0.023514992	No
**221558_s_at**	LEF1	lymphoid enhancer-binding factor 1	21878	-0.35577279329299927	-0.02143489	No
**209621_s_at**	PDLIM3	PDZ and LIM domain 3	21879	-0.355916291475296	-0.018831844	No
**214913_at**	ADAMTS 3	ADAM metallopeptidase with thrombospondin type 1 motif, 3	21914	-0.36738651990890503	-0.017758042	No
**201896_s_at**	PSRC1	proline/serine-rich coiled-coil 1	21934	-0.3756332993507385	-0.015912248	No
**210999_s_at**	GRB10	growth factor receptor-bound protein 10	21958	-0.387459933757782	-0.01416974	No
**205029_s_at**	FABP7	fatty acid binding protein 7, brain	21982	-0.39802658557891846	-0.01234995	No
**220543_at**	C21ORF6 2	chromosome 21 open reading frame 62	21991	-0.4067578911781311	-0.009754626	No
**217546_at**	MT1M	metallothionein 1M	22000	-0.4110591411590576	-0.007127845	No
**204363_at**	F3	coagulation factor III (thromboplastin, tissue factor)	22007	-0.4159297049045563	-0.004370552	No
**203697_at**	FRZB	frizzled-related protein	22024	-0.42138007283210754	-0.002047848	No
**201792_at**	AEBP1	AE binding protein 1	22044	-0.4351990222930908	2,34E+01	No
**202878_s_at**	CD93	CD93 molecule	22061	-0.4460359215736389	0.0027366166	No
**209356_x_at**	EFEMP2	EGF-containing fibulin-like extracellular matrix protein 2	22118	-0.4807509183883667	0.003595735	No
**204865_at**	CA3	carbonic anhydrase III, muscle specific	22186	-0.5657684803009033	0.004554746	No

Supplementary List 6 - List of the 1416 top ranking genes upregulated in MES CSCs *vs*. PN CSCs and selected based on an Adjusted P value < 0.001 e log2 Fold Change > 1 for Gene Set Enrichment Analysis (GSEA) in the comparison PN *vs*. MES.

PROBE	GENE SYMBOL	GENE TITLE	RANK IN GENE LIST	RANK METRIC SCORE	RUNNING ES	CORE ENRICHMENT
**202237_at**	NNMT	nicotinamide N-methyltransferase	1	10.861.561.298.370.300	0.005434823	Yes
**202238_s_at**	NNMT	nicotinamide N-methyltransferase	2	10.627.878.904.342.600	0.01079961	Yes
**216841_s_at**	SOD2	superoxide dismutase 2, mitochondrial	11	0.878744900226593	0.014851996	Yes
**215446_s_at**	LOX	lysyl oxidase	13	0.8573960065841675	0.019132074	Yes
**215076_s_at**	COL3A1	collagen, type III, alpha 1 (Ehlers-Danlos syndrome type IV, autosomal dominant)	18	0.7898093461990356	0.022927217	Yes
**36711_at**	MAFF	v-maf musculoaponeurotic fibrosarcoma oncogene homolog F (avian)	19	0.783990740776062	0.026884679	Yes
**213060_s_at**	CHI3L2	chitinase 3-like 2 /// chitinase 3-like 2	20	0.7751513719558716	0.03079752	Yes
**201505_at**	LAMB1	laminin, beta 1	24	0.7620312571525574	0.034500368	Yes
**209267_s_at**	SLC39A8	solute carrier family 39 (zinc transporter), member 8	25	0.7552747130393982	0.038312875	Yes
**212097_at**	CAV1	caveolin 1, caveolae protein, 22kDa	26	0.7442374229431152	0.04206967	Yes
**209156_s_at**	COL6A2	collagen, type VI, alpha 2	33	0.7060220241546631	0.04534602	Yes
**202270_at**	GBP1	guanylate binding protein 1, interferon-inducible, 67kDa /// guanylate binding protein 1, interferon-inducible, 67kDa	34	0.7032497525215149	0.048895918	Yes
**201110_s_at**	THBS1	thrombospondin 1	35	0.7013509273529053	0.052436225	Yes
**202269_x_at**	GBP1	guanylate binding protein 1, interferon-inducible, 67kDa /// guanylate binding protein 1, interferon-inducible, 67kDa	36	0.7007303833961487	0.055973403	Yes
**201141_at**	GPNMB	glycoprotein (transmembrane) nmb	37	0.6959453225135803	0.059486426	Yes
**221730_at**	COL5A2	collagen, type V, alpha 2	39	0.6912571787834167	0.062927864	Yes
**201852_x_at**	COL3A1	collagen, type III, alpha 1 (Ehlers-Danlos syndrome type IV, autosomal dominant)	40	0.6894629001617432	0.066408165	Yes
**201666_at**	TIMP1	TIMP metallopeptidase inhibitor 1	41	0.6875158548355103	0.06987863	Yes
**215223_s_at**	SOD2	superoxide dismutase 2, mitochondrial	44	0.6835883259773254	0.07323343	Yes
**204298_s_at**	LOX	lysyl oxidase	46	0.6733514666557312	0.07658449	Yes
**202310_s_at**	COL1A1	collagen, type I, alpha 1	58	0.6329898238182068	0.07925257	Yes
**211161_s_at**	COL3A1	collagen, type III, alpha 1 (Ehlers-Danlos syndrome type IV, autosomal dominant)	59	0.6320534944534302	0.08244308	Yes
**200986_at**	SERPING 1	serpin peptidase inhibitor, clade G (C1 inhibitor), member 1, (angioedema, hereditary)	62	0.6233349442481995	0.08549373	Yes
**206584_at**	LY96	lymphocyte antigen 96	64	0.6226014494895935	0.0885886	Yes

**205173_x_at**	CD58	CD58 molecule	70	0.6126250624656677	0.09144142	Yes
**218002_s_at**	CXCL14	chemokine (C-X-C motif) ligand 14	71	0.6100037693977356	0.09452063	Yes
**221729_at**	COL5A2	collagen, type V, alpha 2	72	0.6085588932037354	0.09759254	Yes
**201058_s_at**	MYL9	myosin, light polypeptide 9, regulatory	76	0.599256694316864	0.100473724	Yes
**216598_s_at**	CCL2	chemokine (C-C motif) ligand 2	78	0.595922589302063	0.10343392	Yes
**215506_s_at**	DIRAS3	DIRAS family, GTP-binding RAS-like 3	79	0.5924184322357178	0.10642436	Yes
**211651_s_at**	LAMB1	laminin, beta 1 /// laminin, beta 1	82	0.5853915214538574	0.10928348	Yes
**204517_at**	PPIC	peptidylprolyl isomerase C (cyclophilin C)	85	0.5832906365394592	0.11213199	Yes
**203570_at**	LOXL1	lysyl oxidase-like 1	90	0.5735485553741455	0.114835486	Yes
**208790_s_at**	PTRF	polymerase I and transcript release factor	92	0.5733436346054077	0.11768171	Yes
**202833_s_at**	SERPINA 1	serpin peptidase inhibitor, clade A (alpha-1 antiproteinase, antitrypsin), member 1	99	0.5631321668624878	0.12023678	Yes
**204490_s_at**	CD44	CD44 molecule (Indian blood group)	100	0.562261700630188	0.123074986	Yes
**212298_at**	NRP1	neuropilin 1	103	0.556877613067627	0.12579018	Yes
**203065_s_at**	CAV1	caveolin 1, caveolae protein, 22kDa	108	0.5499752163887024	0.12837467	Yes
**204846_at**	CP	ceruloplasmin (ferroxidase)	112	0.5432648062705994	0.13097322	Yes
**213338_at**	TMEM15 8	transmembrane protein 158	117	0.5341902375221252	0.13347805	Yes
**203423_at**	RBP1	retinol binding protein 1, cellular	118	0.5324801802635193	0.13616592	Yes
**202638_s_at**	ICAM1	intercellular adhesion molecule 1 (CD54), human rhinovirus receptor	120	0.5293906927108765	0.13879028	Yes
**216942_s_at**	CD58	CD58 molecule	125	0.5247935652732849	0.14124766	Yes
**202430_s_at**	PLSCR1	phospholipid scramblase 1	129	0.5211430191993713	0.14373454	Yes
**208747_s_at**	C1S	complement component 1, s subcomponent	132	0.5182478427886963	0.14625473	Yes
**211958_at**	IGFBP5	insulin-like growth factor binding protein 5	134	0.5150505900382996	0.1488067	Yes
**211744_s_at**	CD58	CD58 molecule /// CD58 molecule	135	0.5123868584632874	0.15139315	Yes
**205499_at**	SRPX2	sushi-repeat-containing protein, X-linked 2	138	0.5088974833488464	0.15386614	Yes
**203910_at**	ARHGAP 29	Rho GTPase activating protein 29	141	0.5045077204704285	0.15631697	Yes
**210809_s_at**	POSTN	periostin, osteoblast specific factor	143	0.5024529695510864	0.15880536	Yes
**200974_at**	ACTA2	actin, alpha 2, smooth muscle, aorta	144	0.5012305378913879	0.16133548	Yes
**202437_s_at**	CYP1B1	cytochrome P450, family 1, subfamily B, polypeptide 1	151	0.4935030937194824	0.16353907	Yes
**202291_s_at**	MGP	matrix Gla protein	152	0.49331021308898926	0.16602923	Yes
**209835_x_at**	CD44	CD44 molecule (Indian blood group)	154	0.4927939474582672	0.16846885	Yes
**202748_at**	GBP2	guanylate binding protein 2, interferon-inducible ///	157	0.49117493629455566	0.17085238	Yes

		guanylate binding protein 2, interferon-inducible				
**201136_at**	PLP2	proteolipid protein 2 (colonic epithelium-enriched)	162	0.48545920848846436	0.17311122	Yes
**209344_at**	TPM4	tropomyosin 4	163	0.4824674129486084	0.17554663	Yes
**212014_x_at**	CD44	CD44 molecule (Indian blood group)	171	0.4788469970226288	0.17762832	Yes
**201438_at**	COL6A3	collagen, type VI, alpha 3	172	0.47728532552719116	0.18003757	Yes
**218880_at**	FOSL2	FOS-like antigen 2	174	0.4763893187046051	0.1823944	Yes
**215719_x_at**	FAS	Fas (TNF receptor superfamily, member 6)	175	0.4749926626682815	0.18479209	Yes
**213503_x_at**	ANXA2	annexin A2	176	0.4740018844604492	0.18718477	Yes
**200600_at**	MSN	moesin	182	0.46992748975753784	0.18931727	Yes
**202796_at**	SYNPO	synaptopodin	184	0.46786177158355713	0.19163105	Yes
**210427_x_at**	ANXA2	annexin A2	186	0.4669896066188812	0.19394042	Yes
**217523_at**	CD44	CD44 molecule (Indian blood group)	187	0.46663156151771545	0.1962959	Yes
**202733_at**	P4HA2	procollagen-proline, 2-oxoglutarate 4-dioxygenase (proline 4-hydroxylase), alpha polypeptide II	188	0.4664992094039917	0.19865072	Yes
**201645_at**	TNC	tenascin C (hexabrachion)	189	0.466433048248291	0.20100519	Yes
**201590_x_at**	ANXA2	annexin A2	193	0.46406906843185425	0.20320398	Yes
**204222_s_at**	GLIPR1	GLI pathogenesis-related 1 (glioma)	195	0.46326392889022827	0.20549454	Yes
**203303_at**	DYNLT3	dynein, light chain, Tctex-type 3	196	0.4628744423389435	0.20783105	Yes
**203305_at**	F13A1	coagulation factor XIII, A1 polypeptide	200	0.46126788854599	0.2100157	Yes
**219869_s_at**	SLC39A8	solute carrier family 39 (zinc transporter), member 8	202	0.4606591463088989	0.2122931	Yes
**213446_s_at**	IQGAP1	IQ motif containing GTPase activating protein 1	209	0.45176368951797485	0.214286	Yes
**202446_s_at**	PLSCR1	phospholipid scramblase 1	215	0.44865989685058594	0.21631116	Yes
**204485_s_at**	TOM1L1	target of myb1-like 1 (chicken)	217	0.4480631947517395	0.21852498	Yes
**200791_s_at**	IQGAP1	IQ motif containing GTPase activating protein 1	220	0.4461022913455963	0.220681	Yes
**202998_s_at**	LOXL2	lysyl oxidase-like 2	227	0.4441116750240326	0.22263527	Yes
**207467_x_at**	CAST	calpastatin	235	0.43745294213294983	0.224508	Yes
**210916_s_at**	CD44	CD44 molecule (Indian blood group)	240	0.4329775273799896	0.22650191	Yes
**214085_x_at**	GLIPR1	GLI pathogenesis-related 1 (glioma)	242	0.43140196800231934	0.22863165	Yes
**211429_s_at**	SERPINA 1	serpin peptidase inhibitor, clade A (alpha-1 antiproteinase, antitrypsin), member 1	245	0.4282582998275757	0.23069757	Yes
**203691_at**	PI3	peptidase inhibitor 3, skin-derived (SKALP) /// peptidase inhibitor 3, skin-derived (SKALP)	252	0.42565086483955383	0.23255865	Yes
**205266_at**	LIF	leukemia inhibitory factor (cholinergic differentiation factor)	254	0.4250584542751312	0.23465636	Yes

**212464_s_at**	FN1	fibronectin 1	257	0.42142003774642944	0.23668778	Yes
**203425_s_at**	IGFBP5	insulin-like growth factor binding protein 5	259	0.4208291172981262	0.23876414	Yes
**208782_at**	FSTL1	follistatin-like 1	261	0.42021313309669495	0.24083738	Yes
**212481_s_at**	TPM4	tropomyosin 4	267	0.4177488684654236	0.2427065	Yes
**210840_s_at**	IQGAP1	IQ motif containing GTPase activating protein 1	268	0.4172048270702362	0.24481249	Yes
**204489_s_at**	CD44	CD44 molecule (Indian blood group)	270	0.4166492819786072	0.24686775	Yes
**204780_s_at**	FAS	Fas (TNF receptor superfamily, member 6)	272	0.41632279753685	0.24892135	Yes
**208789_at**	PTRF	polymerase I and transcript release factor	276	0.41555148363113403	0.25087523	Yes
**205729_at**	OSMR	oncostatin M receptor	286	0.4101821184158325	0.25251445	Yes
**202672_s_at**	ATF3	activating transcription factor 3	288	0.40913787484169006	0.2545318	Yes
**212063_at**	CD44	CD44 molecule (Indian blood group)	289	0.4086081385612488	0.2565944	Yes
**219410_at**	TMEM45 A	transmembrane protein 45A	290	0.40688809752464294	0.2586483	Yes
**202436_s_at**	CYP1B1	cytochrome P450, family 1, subfamily B, polypeptide 1	296	0.4016491770744324	0.26043615	Yes
**203042_at**	LAMP2	lysosomal-associated membrane protein 2	297	0.40160873532295227	0.2624634	Yes
**218424_s_at**	STEAP3	STEAP family member 3	299	0.3984810411930084	0.26442695	Yes
**211959_at**	IGFBP5	insulin-like growth factor binding protein 5	302	0.3967672288417816	0.26633394	Yes
**202948_at**	IL1R1	interleukin 1 receptor, type I	304	0.39582425355911255	0.26828405	Yes
**203021_at**	SLPI	secretory leukocyte peptidase inhibitor	308	0.39505642652511597	0.27013448	Yes
**216252_x_at**	FAS	Fas (TNF receptor superfamily, member 6)	312	0.3920726776123047	0.27196983	Yes
**203665_at**	HMOX1	heme oxygenase (decycling) 1	313	0.3906041383743286	0.27394155	Yes
**211719_x_at**	FN1	fibronectin 1 /// fibronectin 1	314	0.3903660178184509	0.27591205	Yes
**205081_at**	CRIP1	cysteine-rich protein 1 (intestinal)	316	0.3898926079273224	0.27783224	Yes
**201215_at**	PLS3	plastin 3 (T isoform)	318	0.38936856389045715	0.27974978	Yes
**211368_s_at**	CASP1	caspase 1, apoptosis-related cysteine peptidase (interleukin 1, beta, convertase)	319	0.38932448625564575	0.28171504	Yes
**202375_at**	SEC24D	SEC24 related gene family, member D (S. cerevisiae)	320	0.3886062502861023	0.28367665	Yes
**203921_at**	CHST2	carbohydrate (N-acetylglucosamine-6-O) sulfotransferase 2	321	0.38850393891334534	0.28563777	Yes
**210260_s_at**	TNFAIP8	tumor necrosis factor, alpha-induced protein 8	323	0.38776275515556335	0.2875472	Yes
**209515_s_at**	RAB27A	RAB27A, member RAS oncogene family	325	0.3867569863796234	0.28945157	Yes
**203041_s_at**	LAMP2	lysosomal-associated membrane protein 2	334	0.38343456387519836	0.2910037	Yes
**215078_at**	SOD2	superoxide dismutase 2, mitochondrial	337	0.38216471672058105	0.29283696	Yes
**203925_at**	GCLM	glutamate-cysteine ligase, modifier subunit	355	0.3728453814983368	0.29390436	Yes

**216442_x_at**	FN1	fibronectin 1	360	0.3714999258518219	0.29558793	Yes
**203424_s_at**	IGFBP5	insulin-like growth factor binding protein 5	362	0.3708290457725525	0.2974119	Yes
**202625_at**	LYN	v-yes-1 Yamaguchi sarcoma viral related oncogene homolog /// v-yes-1 Yamaguchi sarcoma viral related oncogene homolog	365	0.3678034245967865	0.29917267	Yes
**212724_at**	RND3	Rho family GTPase 3	367	0.36752748489379883	0.30097997	Yes
**201109_s_at**	THBS1	thrombospondin 1	371	0.36620810627937317	0.30268475	Yes
**204221_x_at**	GLIPR1	GLI pathogenesis-related 1 (glioma)	373	0.3656924366950989	0.3044828	Yes
**204518_s_at**	PPIC	peptidylprolyl isomerase C (cyclophilin C)	380	0.36392879486083984	0.30603233	Yes
**206101_at**	ECM2	extracellular matrix protein 2, female organ and adipocyte specific	385	0.36209967732429504	0.30766845	Yes
**205943_at**	TDO2	tryptophan 2,3-dioxygenase	397	0.3574429452419281	0.30894563	Yes
**208296_x_at**	TNFAIP8	tumor necrosis factor, alpha-induced protein 8	406	0.35561761260032654	0.31035733	Yes
**205825_at**	PCSK1	proprotein convertase subtilisin/kexin type 1	409	0.3547059893608093	0.31205198	Yes
**201487_at**	CTSC	cathepsin C	421	0.3505920171737671	0.3132946	Yes
**210495_x_at**	FN1	fibronectin 1	424	0.350460946559906	0.3149678	Yes
**201287_s_at**	SDC1	syndecan 1	434	0.3471185564994812	0.3162887	Yes
**209732_at**	CLEC2B	C-type lectin domain family 2, member B	441	0.34417349100112915	0.3177385	Yes
**200737_at**	PGK1	phosphoglycerate kinase 1	446	0.34244370460510254	0.3192754	Yes
**204259_at**	MMP7	matrix metallopeptidase 7 (matrilysin, uterine)	448	0.3423157334327698	0.32095546	Yes
**210145_at**	PLA2G4A	phospholipase A2, group IVA (cytosolic, calcium-dependent)	451	0.3415105640888214	0.3225835	Yes
**212091_s_at**	COL6A1	collagen, type VI, alpha 1	452	0.34117433428764343	0.32430568	Yes
**208908_s_at**	CAST	calpastatin	453	0.3410177528858185	0.3260271	Yes
**219888_at**	SPAG4	sperm associated antigen 4	455	0.3406703472137451	0.32769883	Yes
**204051_s_at**	SFRP4	secreted frizzled-related protein 4	458	0.33830803632736206	0.32931072	Yes
**214853_s_at**	SHC1	SHC (Src homology 2 domain containing) transforming protein 1	469	0.3336557149887085	0.3305157	Yes
**211367_s_at**	CASP1	caspase 1, apoptosis-related cysteine peptidase (interleukin 1, beta, convertase)	472	0.3322663903236389	0.3320971	Yes
**202555_s_at**	MYLK	myosin, light polypeptide kinase /// myosin, light polypeptide kinase	474	0.33212363719940186	0.3337257	Yes
**206011_at**	CASP1	caspase 1, apoptosis-related cysteine peptidase (interleukin 1, beta, convertase)	475	0.33189988136291504	0.33540106	Yes
**212158_at**	SDC2	syndecan 2 (heparan sulfate proteoglycan 1, cell surface-associated, fibroglycan)	477	0.3313702344894409	0.33702585	Yes

**201302_at**	ANXA4	annexin A4	478	0.33125653862953186	0.33869797	Yes
**202820_at**	AHR	aryl hydrocarbon receptor	481	0.3301985263824463	0.3402689	Yes
**201469_s_at**	SHC1	SHC (Src homology 2 domain containing) transforming protein 1	483	0.3299143314361572	0.34188637	Yes
**204897_at**	PTGER4	prostaglandin E receptor 4 (subtype EP4)	484	0.3296561539173126	0.3435504	Yes
**204748_at**	PTGS2	prostaglandin-endoperoxide synthase 2 (prostaglandin G/H synthase and cyclooxygenase)	485	0.3293932378292084	0.34521315	Yes
**221107_at**	CHRNA9	cholinergic receptor, nicotinic, alpha 9	498	0.3255925476551056	0.34628162	Yes
**204602_at**	DKK1	dickkopf homolog 1 (Xenopus laevis)	503	0.323793888092041	0.34772438	Yes
**217995_at**	SQRDL	sulfide quinone reductase-like (yeast)	505	0.3233276605606079	0.34930855	Yes
**212154_at**	SDC2	syndecan 2 (heparan sulfate proteoglycan 1, cell surface-associated, fibroglycan)	508	0.32232996821403503	0.3508398	Yes
**202901_x_at**	CTSS	cathepsin S	509	0.32194605469703674	0.3524649	Yes
**216005_at**	TNC	Tenascin C (hexabrachion)	517	0.32068192958831787	0.3537482	Yes
**221773_at**	ELK3	ELK3, ETS-domain protein (SRF accessory protein 2)	519	0.32019734382629395	0.3553166	Yes
**202357_s_at**	CFB	complement factor B	523	0.3194219470024109	0.3567852	Yes
**201301_s_at**	ANXA4	annexin A4	524	0.3192821145057678	0.35839692	Yes
**211675_s_at**	MDFIC	MyoD family inhibitor domain containing /// MyoD family inhibitor domain containing	525	0.3188583552837372	0.36000645	Yes
**202311_s_at**	COL1A1	collagen, type I, alpha 1	526	0.31879234313964844	0.36161566	Yes
**204781_s_at**	FAS	Fas (TNF receptor superfamily, member 6)	528	0.3170970380306244	0.3631684	Yes
**213125_at**	OLFML2B	olfactomedin-like 2B	530	0.31671571731567383	0.3647192	Yes
**209875_s_at**	SPP1	secreted phosphoprotein 1 (osteopontin, bone sialoprotein I, early T-lymphocyte activation 1)	531	0.31588834524154663	0.36631376	Yes
**201286_at**	SDC1	syndecan 1	532	0.3153895437717438	0.3679058	Yes
**202087_s_at**	CTSL	cathepsin L	537	0.31377771496772766	0.369298	Yes
**202435_s_at**	CYP1B1	cytochrome P450, family 1, subfamily B, polypeptide 1	539	0.31333208084106445	0.37083173	Yes
**210845_s_at**	PLAUR	plasminogen activator, urokinase receptor	540	0.31307563185691833	0.3724121	Yes
**218736_s_at**	PALMD	palmdelphin	552	0.3079301118850708	0.37343934	Yes
**206825_at**	OXTR	oxytocin receptor	558	0.30678704380989075	0.37474832	Yes
**202180_s_at**	MVP	major vault protein	570	0.3041495680809021	0.37575647	Yes
**205207_at**	IL6	interleukin 6 (interferon, beta 2)	578	0.302767813205719	0.37694934	Yes
**201474_s_at**	ITGA3	integrin, alpha 3 (antigen CD49C, alpha 3 subunit of VLA-3 receptor)	586	0.2990971803665161	0.3781237	Yes

**206336_at**	CXCL6	chemokine (C-X-C motif) ligand 6 (granulocyte chemotactic protein 2)	591	0.298214852809906	0.37943733	Yes
**214974_x_at**	CXCL5	chemokine (C-X-C motif) ligand 5	599	0.29696932435035706	0.38060093	Yes
**201631_s_at**	IER3	immediate early response 3	602	0.29668185114860535	0.38200268	Yes
**220591_s_at**	EFHC2	EF-hand domain (C-terminal) containing 2	604	0.2956761419773102	0.3834473	Yes
**203426_s_at**	IGFBP5	insulin-like growth factor binding protein 5	605	0.2955417037010193	0.38493913	Yes
**207076_s_at**	ASS1	argininosuccinate synthetase 1	633	0.2896791994571686	0.3851075	Yes
**203411_s_at**	LMNA	lamin A/C	642	0.2868761718273163	0.3861722	Yes
**205904_at**	MICA	MHC class I polypeptide-related sequence A	644	0.2865743935108185	0.3875709	Yes
**212586_at**	CAST	calpastatin	646	0.2861398160457611	0.38896734	Yes
**204933_s_at**	TNFRSF1 1B	tumor necrosis factor receptor superfamily, member 11b (osteoprotegerin)	649	0.28607606887817383	0.39031556	Yes
**31845_at**	ELF4	E74-like factor 4 (ets domain transcription factor)	650	0.2853066027164459	0.39175576	Yes
**213988_s_at**	SAT1	spermidine/spermine N1-acetyltransferase 1	655	0.28376561403274536	0.39299646	Yes
**203282_at**	GBE1	glucan (1,4-alpha-), branching enzyme 1 (glycogen branching enzyme, Andersen disease, glycogen storage disease type IV)	657	0.2835060656070709	0.39437965	Yes
**201641_at**	BST2	bone marrow stromal cell antigen 2	661	0.2831474542617798	0.39566514	Yes
**212110_at**	SLC39A1 4	solute carrier family 39 (zinc transporter), member 14	667	0.281805157661438	0.39684805	Yes
**209631_s_at**	GPR37	G protein-coupled receptor 37 (endothelin receptor type B-like)	671	0.28079327940940857	0.39812168	Yes
**202388_at**	RGS2	regulator of G-protein signalling 2, 24kDa	678	0.27876874804496765	0.39924133	Yes
**202688_at**	TNFSF10	tumor necrosis factor (ligand) superfamily, member 10 /// tumor necrosis factor (ligand) superfamily, member 10	685	0.27743664383888245	0.40035424	Yes
**209970_x_at**	CASP1	caspase 1, apoptosis-related cysteine peptidase (interleukin 1, beta, convertase)	686	0.27736103534698486	0.40175432	Yes
**202510_s_at**	TNFAIP2	tumor necrosis factor, alpha-induced protein 2	688	0.27665841579437256	0.40310293	Yes
**210519_s_at**	NQO1	NAD(P)H dehydrogenase, quinone 1	706	0.2716992497444153	0.40365973	Yes
**218196_at**	OSTM1	osteopetrosis associated transmembrane protein 1	712	0.269161581993103	0.4047788	Yes
**204472_at**	GEM	GTP binding protein overexpressed in skeletal muscle	713	0.2687031030654907	0.40613517	Yes
**221760_at**	MAN1A1	Mannosidase, alpha, class 1A, member 1	722	0.267782062292099	0.40710354	Yes
**212501_at**	CEBPB	CCAAT/enhancer binding protein (C/EBP), beta	724	0.2675548195838928	0.40840617	Yes
**200906_s_at**	PALLD	palladin, cytoskeletal associated protein	726	0.267325222492218	0.40970767	Yes

**210986_s_at**	TPM1	tropomyosin 1 (alpha)	727	0.26699298620224	0.41105542	Yes
**209340_at**	UAP1	UDP-N-acteylglucosamine pyrophosphorylase 1	729	0.2665344178676605	0.41235292	Yes
**203510_at**	MET	met proto-oncogene (hepatocyte growth factor receptor)	731	0.266333669424057	0.4136494	Yes
**204332_s_at**	AGA	aspartylglucosaminidase	732	0.26606485247612	0.41499245	Yes
**217356_s_at**	PGK1	phosphoglycerate kinase 1	733	0.2659912407398224	0.41633514	Yes
**204955_at**	SRPX	sushi-repeat-containing protein, X-linked	740	0.2645568549633026	0.41738304	Yes
**214038_at**	CCL8	chemokine (C-C motif) ligand 8	743	0.2636667788028717	0.41861814	Yes
**217728_at**	S100A6	S100 calcium binding protein A6 (calcyclin)	749	0.2624511420726776	0.41970333	Yes
**204158_s_at**	TCIRG1	T-cell, immune regulator 1, ATPase, H+ transporting, lysosomal V0 subunit A3	751	0.2622676491737366	0.4209793	Yes
**221881_s_at**	CLIC4	chloride intracellular channel 4	767	0.26045888662338257	0.42157522	Yes
**201162_at**	IGFBP7	insulin-like growth factor binding protein 7	769	0.2597317099571228	0.4228384	Yes
**209040_s_at**	PSMB8	proteasome (prosome, macropain) subunit, beta type, 8 (large multifunctional peptidase 7)	771	0.2593623101711273	0.42409968	Yes
**221816_s_at**	PHF11	PHD finger protein 11	779	0.25851643085479736	0.42506918	Yes
**202626_s_at**	LYN	v-yes-1 Yamaguchi sarcoma viral related oncogene homolog /// v-yes-1 Yamaguchi sarcoma viral related oncogene homolog	785	0.25707492232322693	0.42612723	Yes
**209739_s_at**	PNPLA4	patatin-like phospholipase domain containing 4	789	0.25636255741119385	0.42727754	Yes
**203455_s_at**	SAT1	spermidine/spermine N1-acetyltransferase 1	799	0.25419488549232483	0.42812937	Yes
**201163_s_at**	IGFBP7	insulin-like growth factor binding protein 7	800	0.25344541668891907	0.42940873	Yes
**205199_at**	CA9	carbonic anhydrase IX	801	0.25335222482681274	0.4306876	Yes
**200656_s_at**	P4HB	procollagen-proline, 2-oxoglutarate 4-dioxygenase (proline 4-hydroxylase), beta polypeptide	806	0.25263822078704834	0.4317712	Yes
**211945_s_at**	ITGB1	integrin, beta 1 (fibronectin receptor, beta polypeptide, antigen CD29 includes MDF2, MSK12)	807	0.25257474184036255	0.43304616	Yes
**200738_s_at**	PGK1	phosphoglycerate kinase 1	808	0.252276748418808	0.43431962	Yes
**202862_at**	FAH	fumarylacetoacetate hydrolase (fumarylacetoacetase)	812	0.25204724073410034	0.43544814	Yes
**201016_at**	EIF1AX	eukaryotic translation initiation factor 1A, X-linked	813	0.25189054012298584	0.43671966	Yes
**214586_at**	GPR37	G protein-coupled receptor 37 (endothelin receptor type B-like)	823	0.2504770755767822	0.43755272	Yes
**219506_at**	C1ORF54	chromosome 1 open reading frame 54	824	0.2503291368484497	0.43881634	Yes
**202086_at**	MX1	protein p78 (mouse)	828	0.2501259446144104	0.43993515	Yes

**202637_s_at**	ICAM1	intercellular adhesion molecule 1 (CD54), human rhinovirus receptor	844	0.24737504124641418	0.44046503	Yes
**203650_at**	PROCR	protein C receptor, endothelial (EPCR)	846	0.24640558660030365	0.44166094	Yes
**201041_s_at**	DUSP1	dual specificity phosphatase 1	853	0.24571162462234497	0.4426137	Yes
**213428_s_at**	COL6A1	collagen, type VI, alpha 1	857	0.2451377958059311	0.44370735	Yes
**218823_s_at**	KCTD9	potassium channel tetramerisation domain containing 9	860	0.24456463754177094	0.44484603	Yes
**204463_s_at**	EDNRA	endothelin receptor type A	863	0.24430687725543976	0.4459834	Yes
**209154_at**	TAX1BP3	Tax1 (human T-cell leukemia virus type I) binding protein 3	864	0.2442103624343872	0.44721615	Yes
**212848_s_at**	C9ORF3	chromosome 9 open reading frame 3	871	0.24288924038410187	0.4481547	Yes
**215464_s_at**	TAX1BP3	Tax1 (human T-cell leukemia virus type I) binding protein 3	873	0.24280719459056854	0.44933242	Yes
**204932_at**	TNFRSF1 1B	tumor necrosis factor receptor superfamily, member 11b (osteoprotegerin)	875	0.2421516627073288	0.45050684	Yes
**209326_at**	SLC35A2	solute carrier family 35 (UDP-galactose transporter), member A2	877	0.24079419672489166	0.4516744	Yes
**203593_at**	CD2AP	CD2-associated protein	878	0.24078933894634247	0.45288986	Yes
**207850_at**	CXCL3	chemokine (C-X-C motif) ligand 3	885	0.2394527792930603	0.45381105	Yes
**205870_at**	BDKRB2	bradykinin receptor B2	893	0.23832841217517853	0.45467862	Yes
**219283_at**	C1GALT1 C1	C1GALT1-specific chaperone 1	896	0.2379733920097351	0.45578405	Yes
**209417_s_at**	IFI35	interferon-induced protein 35	899	0.2376018464565277	0.45688757	Yes
**208944_at**	TGFBR2	transforming growth factor, beta receptor II (70/80kDa)	908	0.23630952835083008	0.45769703	Yes
**210592_s_at**	SAT1	spermidine/spermine N1-acetyltransferase 1	914	0.2360546886920929	0.458649	Yes
**205105_at**	MAN2A1	mannosidase, alpha, class 2A, member 1	922	0.23522759974002838	0.45950094	Yes
**208864_s_at**	TXN	thioredoxin	932	0.23376061022281647	0.46024963	Yes
**204005_s_at**	PAWR	PRKC, apoptosis, WT1, regulator	933	0.2336987406015396	0.4614293	Yes
**210951_x_at**	RAB27A	RAB27A, member RAS oncogene family	940	0.23244047164916992	0.46231508	Yes
**205542_at**	STEAP1	six transmembrane epithelial antigen of the prostate 1	941	0.23221173882484436	0.46348724	Yes
**213640_s_at**	LOX	lysyl oxidase	942	0.23160390555858612	0.46465635	Yes
**202950_at**	CRYZ	crystallin, zeta (quinone reductase)	945	0.23078469932079315	0.46572548	Yes
**203823_at**	RGS3	regulator of G-protein signalling 3	946	0.2306644767522812	0.46688983	Yes
**203903_s_at**	HEPH	hephaestin	960	0.22797080874443054	0.4674176	Yes
**200782_at**	ANXA5	annexin A5	970	0.22652441263198853	0.46812975	Yes
**204430_s_at**	SLC2A5	solute carrier family 2 (facilitated glucose/fructose transporter), member 5	974	0.22592274844646454	0.4691264	Yes
**202531_at**	IRF1	interferon regulatory factor 1	975	0.22559523582458496	0.47026518	Yes
**211366_x_at**	CASP1	caspase 1, apoptosis-related cysteine peptidase	979	0.22538000345230103	0.4712591	Yes

		(interleukin 1, beta, convertase)				
**216064_s_at**	AGA	aspartylglucosaminidase	992	0.2223784178495407	0.47180656	Yes
**207992_s_at**	AMPD3	adenosine monophosphate deaminase (isoform E)	994	0.22194162011146545	0.47287896	Yes
**212472_at**	MICAL2	microtubule associated monoxygenase, calponin and LIM domain containing 2	996	0.2217075377702713	0.47395018	Yes
**202902_s_at**	CTSS	cathepsin S	1000	0.2210456281900406	0.4749222	Yes
**200923_at**	LGALS3BP	lectin, galactoside-binding, soluble, 3 binding protein	1001	0.22102797031402588	0.47603792	Yes
**212086_x_at**	LMNA	lamin A/C	1007	0.2206099033355713	0.47691193	Yes
**200907_s_at**	PALLD	palladin, cytoskeletal associated protein	1011	0.21977832913398743	0.47787756	Yes
**205193_at**	MAFF	v-maf musculoaponeurotic fibrosarcoma oncogene homolog F (avian)	1012	0.21972370147705078	0.47898668	Yes
**203814_s_at**	NQO2	NAD(P)H dehydrogenase, quinone 2	1013	0.21955181658267975	0.48009497	Yes
**41469_at**	PI3	peptidase inhibitor 3, skin-derived (SKALP)	1014	0.21951185166835785	0.48120302	Yes
**214453_s_at**	IFI44	interferon-induced protein 44	1020	0.218451589345932	0.48206612	Yes
**204464_s_at**	EDNRA	endothelin receptor type A	1021	0.21836185455322266	0.48316836	Yes
**205242_at**	CXCL13	chemokine (C-X-C motif) ligand 13 (B-cell chemoattractant)	1028	0.21753297746181488	0.4839789	Yes
**211926_s_at**	MYH9	myosin, heavy polypeptide 9, non-muscle	1029	0.2174818366765976	0.48507673	Yes
**211965_at**	ZFP36L1	zinc finger protein 36, C3H type-like 1	1031	0.21721242368221283	0.48612526	Yes
**204928_s_at**	SLC10A3	solute carrier family 10 (sodium/bile acid cotransporter family), member 3	1032	0.21696311235427856	0.48722044	Yes
**204333_s_at**	AGA	aspartylglucosaminidase	1035	0.21664755046367645	0.48821822	Yes
**203476_at**	TPBG	trophoblast glycoprotein	1045	0.2150317132472992	0.48887235	Yes
**210959_s_at**	SRD5A1	steroid-5-alpha-reductase, alpha polypeptide 1 (3-oxo-5 alpha-steroid delta 4-dehydrogenase alpha 1)	1049	0.21475033462047577	0.4898126	Yes
**218205_s_at**	MKNK2	MAP kinase interacting serine/threonine kinase 2	1054	0.2145136445760727	0.49070376	Yes
**203851_at**	IGFBP6	insulin-like growth factor binding protein 6	1057	0.21328292787075043	0.49168453	Yes
**204004_at**	PAWR	PRKC, apoptosis, WT1, regulator	1061	0.2129514068365097	0.4926157	Yes
**212865_s_at**	COL14A1	collagen, type XIV, alpha 1 (undulin)	1071	0.21165820956230164	0.4932528	Yes
**220975_s_at**	C1QTNF1	C1q and tumor necrosis factor related protein 1 /// C1q and tumor necrosis factor related protein 1	1080	0.21084554493427277	0.49393374	Yes
**212937_s_at**	COL6A1	collagen, type VI, alpha 1	1088	0.20958435535430908	0.49465623	Yes
**219985_at**	HS3ST3A1	heparan sulfate (glucosamine) 3-O-sulfotransferase 3A1	1096	0.20895352959632874	0.49537554	Yes
**201315_x_at**	IFITM2	interferon induced transmembrane protein 2 (1-8D)	1099	0.2088555097579956	0.49633396	Yes

**202687_s_at**	TNFSF10	tumor necrosis factor (ligand) superfamily, member 10 /// tumor necrosis factor (ligand) superfamily, member 10	1102	0.20839254558086395	0.49729005	Yes
**201108_s_at**	THBS1	thrombospondin 1	1104	0.20820096135139465	0.4982931	Yes
**201560_at**	CLIC4	chloride intracellular channel 4	1107	0.20782162249088287	0.4992463	Yes
**203072_at**	MYO1E	myosin IE	1111	0.2076515406370163	0.50015074	Yes
**202016_at**	MEST	mesoderm specific transcript homolog (mouse)	1132	0.20517922937870026	0.500228	Yes
**204070_at**	RARRES3	retinoic acid receptor responder (tazarotene induced) 3	1147	0.20323950052261353	0.500583	Yes
**203233_at**	IL4R	interleukin 4 receptor	1149	0.2031058669090271	0.50156033	Yes
**201471_s_at**	SQSTM1	sequestosome 1	1161	0.2018553763628006	0.5020521	Yes
**209846_s_at**	BTN3A2	butyrophilin, subfamily 3, member A2	1165	0.2014298439025879	0.50292516	Yes
**201559_s_at**	CLIC4	chloride intracellular channel 4	1173	0.2005625069141388	0.5036021	Yes
**212203_x_at**	IFITM3	interferon induced transmembrane protein 3 (1-8U)	1174	0.2005462497472763	0.5046144	Yes
**203407_at**	PPL	periplakin	1178	0.2003227323293686	0.50548184	Yes
**210754_s_at**	LYN	v-yes-1 Yamaguchi sarcoma viral related oncogene homolog	1181	0.19975902140140533	0.5063943	Yes
**219229_at**	SLCO3A1	solute carrier organic anion transporter family, member 3A1	1185	0.1990458071231842	0.5072553	Yes
**202600_s_at**	NRIP1	nuclear receptor interacting protein 1	1188	0.1986156851053238	0.5081621	Yes
**210510_s_at**	NRP1	neuropilin 1	1202	0.19625990092754364	0.5085298	Yes
**221666_s_at**	PYCARD	PYD and CARD domain containing	1209	0.19547392427921295	0.50922894	Yes
**213425_at**	WNT5A	wingless-type MMTV integration site family, member 5A /// wingless-type MMTV integration site family, member 5A	1230	0.19238199293613434	0.5092416	Yes
**204279_at**	PSMB9	proteasome (prosome, macropain) subunit, beta type, 9 (large multifunctional peptidase 2)	1231	0.1923297494649887	0.5102125	Yes
**210751_s_at**	RGN	regucalcin (senescence marker protein-30)	1237	0.1915624439716339	0.51093984	Yes
**219505_at**	CECR1	cat eye syndrome chromosome region, candidate 1	1242	0.19133360683918	0.511714	Yes
**200931_s_at**	VCL	vinculin	1249	0.19057346880435944	0.5123884	Yes
**205140_at**	FPGT	fucose-1-phosphate guanylyltransferase	1256	0.1896439641714096	0.5130582	Yes
**200897_s_at**	PALLD	palladin, cytoskeletal associated protein	1262	0.18926392495632172	0.5137739	Yes
**205990_s_at**	WNT5A	wingless-type MMTV integration site family, member 5A	1264	0.18889759480953217	0.51467955	Yes
**213075_at**	OLFML2A	olfactomedin-like 2A	1266	0.18836985528469086	0.51558244	Yes

**216483_s_at**	C19ORF10	chromosome 19 open reading frame 10	1277	0.18806001543998718	0.51605254	Yes
**202888_s_at**	ANPEP	alanyl (membrane) aminopeptidase (aminopeptidase N, aminopeptidase M, microsomal aminopeptidase, CD13, p150)	1290	0.18630020320415497	0.51641786	Yes
**204214_s_at**	RAB32	RAB32, member RAS oncogene family	1293	0.1862868070602417	0.5172624	Yes
**215664_s_at**	EPHA5	EPH receptor A5	1295	0.1860833764076233	0.5181538	Yes
**214440_at**	NAT1	N-acetyltransferase 1 (arylamine N-acetyltransferase)	1314	0.18398381769657135	0.5182199	Yes
**201467_s_at**	NQO1	NAD(P)H dehydrogenase, quinone 1	1321	0.18343129754066467	0.5188583	Yes
**204834_at**	FGL2	fibrinogen-like 2	1329	0.1823500096797943	0.51944333	Yes
**202307_s_at**	TAP1	transporter 1, ATP-binding cassette, sub-family B (MDR/TAP)	1333	0.1821417212486267	0.52021897	Yes
**218217_at**	SCPEP1	serine carboxypeptidase 1	1334	0.18199490010738373	0.52113765	Yes
**213438_at**	NFASC	neurofascin homolog (chicken)	1338	0.18150465190410614	0.5219101	Yes
**204998_s_at**	ATF5	activating transcription factor 5	1340	0.18138708174228668	0.5227778	Yes
**52255_s_at**	COL5A3	collagen, type V, alpha 3	1341	0.18135099112987518	0.5236932	Yes
**212647_at**	RRAS	related RAS viral (r-ras) oncogene homolog	1342	0.18132968246936798	0.52460855	Yes
**219029_at**	C5ORF28	chromosome 5 open reading frame 28	1360	0.17912527918815613	0.524698	Yes
**219863_at**	HERC5	hect domain and RLD 5	1361	0.17909100651741028	0.52560204	Yes
**204999_s_at**	ATF5	activating transcription factor 5	1363	0.17878568172454834	0.52645665	Yes
**212940_at**	COL6A1	collagen, type VI, alpha 1	1366	0.1785876303911209	0.5272623	Yes
**202201_at**	BLVRB	biliverdin reductase B (flavin reductase (NADPH))	1373	0.17808659374713898	0.5278737	Yes
**203028_s_at**	CYBA	cytochrome b-245, alpha polypeptide	1376	0.17790073156356812	0.52867585	Yes
**201673_s_at**	GYS1	glycogen synthase 1 (muscle)	1381	0.1776486337184906	0.5293809	Yes
**218145_at**	TRIB3	tribbles homolog 3 (Drosophila)	1383	0.17735913395881653	0.53022826	Yes
**221675_s_at**	CHPT1	choline phosphotransferase 1	1385	0.1772233098745346	0.53107494	Yes
**203717_at**	DPP4	dipeptidyl-peptidase 4 (CD26, adenosine deaminase complexing protein 2)	1413	0.17509867250919342	0.5306649	Yes
**211478_s_at**	DPP4	dipeptidyl-peptidase 4 (CD26, adenosine deaminase complexing protein 2)	1415	0.175028994679451	0.5315005	Yes
**220272_at**	BNC2	basonuclin 2	1417	0.1749013215303421	0.53233546	Yes
**218638_s_at**	SPON2	spondin 2, extracellular matrix protein	1424	0.17407061159610748	0.53292656	Yes
**218070_s_at**	GMPPA	GDP-mannose pyrophosphorylase A	1426	0.17403317987918854	0.53375715	Yes
**202052_s_at**	RAI14	retinoic acid induced 14	1428	0.17400670051574707	0.5345876	Yes
**211799_x_at**	HLA-C	major histocompatibility complex, class I, C	1429	0.1738465279340744	0.5354651	Yes

**202609_at**	EPS8	epidermal growth factor receptor pathway substrate 8	1435	0.17349384725093842	0.5361013	Yes
**203843_at**	RPS6KA3	ribosomal protein S6 kinase, 90kDa, polypeptide 3	1437	0.1734631061553955	0.536929	Yes
**211911_x_at**	HLA-B	major histocompatibility complex, class I, B /// major histocompatibility complex, class I, B	1439	0.17333072423934937	0.537756	Yes
**201461_s_at**	MAPKAP K2	mitogen-activated protein kinase-activated protein kinase 2	1442	0.17313632369041443	0.53853416	Yes
**200821_at**	LAMP2	lysosomal-associated membrane protein 2	1443	0.17309288680553436	0.5394079	Yes
**201468_s_at**	NQO1	NAD(P)H dehydrogenase, quinone 1	1444	0.17298759520053864	0.5402811	Yes
**201096_s_at**	ARF4	ADP-ribosylation factor 4	1449	0.17260144650936127	0.54096067	Yes
**202734_at**	TRIP10	thyroid hormone receptor interactor 10	1451	0.17252762615680695	0.54178363	Yes
**204475_at**	MMP1	matrix metallopeptidase 1 (interstitial collagenase)	1465	0.17168064415454865	0.5420273	Yes
**212459_x_at**	SUCLG2	succinate-CoA ligase, GDP-forming, beta subunit	1466	0.17167817056179047	0.5428939	Yes
**205100_at**	GFPT2	glutamine-fructose-6-phosphate transaminase 2	1467	0.17155663669109344	0.5437599	Yes
**217949_s_at**	VKORC1	vitamin K epoxide reductase complex, subunit 1	1470	0.17114731669425964	0.54452795	Yes
**211962_s_at**	ZFP36L1	zinc finger protein 36, C3H type-like 1	1478	0.17011308670043945	0.54505116	Yes
**218163_at**	MCTS1	malignant T cell amplified sequence 1	1480	0.16995328664779663	0.5458612	Yes
**212192_at**	KCTD12	potassium channel tetramerisation domain containing 12	1482	0.16983211040496826	0.5466705	Yes
**214846_s_at**	ALPK3	alpha-kinase 3	1487	0.1694703847169876	0.5473343	Yes
**205812_s_at**	TMED9	transmembrane emp24 protein transport domain containing 9	1490	0.16931109130382538	0.5480931	Yes
**219449_s_at**	TMEM70	transmembrane protein 70	1492	0.1691903918981552	0.54889923	Yes
**203201_at**	PMM2	phosphomannomutase 2	1494	0.16887131333351135	0.5497037	Yes
**210042_s_at**	CTSZ	cathepsin Z	1503	0.16840599477291107	0.5501704	Yes
**209584_x_at**	APOBEC 3C	apolipoprotein B mRNA editing enzyme, catalytic polypeptide-like 3C	1513	0.1670963317155838	0.55058265	Yes
**205168_at**	DDR2	discoidin domain receptor family, member 2	1514	0.16697902977466583	0.5514255	Yes
**215772_x_at**	SUCLG2	succinate-CoA ligase, GDP-forming, beta subunit	1523	0.16608066856861115	0.5518805	Yes
**213372_at**	PAQR3	progestin and adipoQ receptor family member III	1527	0.16582559049129486	0.55257374	Yes
**201883_s_at**	B4GALT1	UDP-Gal:betaGlcNAc beta 1,4- galactosyltransferase, polypeptide 1	1529	0.16573166847229004	0.5533624	Yes
**219304_s_at**	PDGFD	platelet derived growth factor D	1534	0.16533726453781128	0.5540053	Yes
**201200_at**	CREG1	cellular repressor of E1A-stimulated genes 1	1535	0.1652938276529312	0.55483973	Yes
**214835_s_at**	SUCLG2	succinate-CoA ligase, GDP-forming, beta subunit	1538	0.16524432599544525	0.555578	Yes
**218472_s_at**	PELO	pelota homolog (Drosophila)	1550	0.16442328691482544	0.55588084	Yes

**218656_s_at**	LHFP	lipoma HMGIC fusion partner	1555	0.16393426060676575	0.55651665	Yes
**205174_s_at**	QPCT	glutaminyl-peptide cyclotransferase (glutaminyl cyclase)	1565	0.16332855820655823	0.5569098	Yes
**204059_s_at**	ME1	malic enzyme 1, NADP(+)-dependent, cytosolic	1566	0.16316643357276917	0.5577335	Yes
**212887_at**	SEC23A	Sec23 homolog A (S. cerevisiae)	1570	0.16280969977378845	0.55841154	Yes
**214701_s_at**	FN1	fibronectin 1	1573	0.16239358484745026	0.55913544	Yes
**217785_s_at**	YKT6	YKT6 v-SNARE homolog (S. cerevisiae)	1591	0.1609819382429123	0.55913335	Yes
**202599_s_at**	NRIP1	nuclear receptor interacting protein 1	1597	0.16057227551937103	0.55970424	Yes
**205422_s_at**	ITGBL1	integrin, beta-like 1 (with EGF-like repeat domains)	1599	0.16050466895103455	0.5604665	Yes
**209365_s_at**	ECM1	extracellular matrix protein 1	1600	0.16050222516059875	0.56127673	Yes
**211924_s_at**	PLAUR	plasminogen activator, urokinase receptor /// plasminogen activator, urokinase receptor	1608	0.15966731309890747	0.56174725	Yes
**218204_s_at**	FYCO1	FYVE and coiled-coil domain containing 1	1612	0.1592215895652771	0.5624072	Yes
**221739_at**	C19ORF10	chromosome 19 open reading frame 10	1614	0.15899305045604706	0.56316185	Yes
**201810_s_at**	SH3BP5	SH3-domain binding protein 5 (BTK-associated)	1619	0.15870553255081177	0.5637713	Yes
**219561_at**	COPZ2	coatomer protein complex, subunit zeta 2	1620	0.1586531549692154	0.56457216	Yes
**218668_s_at**	RAP2C	RAP2C, member of RAS oncogene family	1639	0.15712003409862518	0.56450266	Yes
**205641_s_at**	TRADD	TNFRSF1A-associated via death domain	1640	0.15707619488239288	0.5652956	Yes
**212174_at**	AK2	adenylate kinase 2	1642	0.15692129731178284	0.56603974	Yes
**217784_at**	YKT6	YKT6 v-SNARE homolog (S. cerevisiae)	1644	0.1568741649389267	0.5667837	Yes
**205452_at**	PIGB	phosphatidylinositol glycan, class B	1645	0.1567411869764328	0.5675749	Yes
**203939_at**	NT5E	5'-nucleotidase, ecto (CD73)	1649	0.1561056524515152	0.5682191	Yes
**208729_x_at**	HLA-B	major histocompatibility complex, class I, B	1663	0.1549016237258911	0.5683781	Yes
**212157_at**	SDC2	syndecan 2 (heparan sulfate proteoglycan 1, cell surface-associated, fibroglycan)	1672	0.15379665791988373	0.568771	Yes
**201443_s_at**	ATP6AP2	ATPase, H+ transporting, lysosomal accessory protein 2	1681	0.15357264876365662	0.56916285	Yes
**203404_at**	ARMCX2	armadillo repeat containing, X-linked 2	1692	0.15317487716674805	0.5694568	Yes
**205409_at**	FOSL2	FOS-like antigen 2	1694	0.1528746634721756	0.5701806	Yes
**204554_at**	PPP1R3D	protein phosphatase 1, regulatory subunit 3D	1695	0.15284988284111023	0.5709522	Yes
**221653_x_at**	APOL2	apolipoprotein L, 2	1697	0.15269915759563446	0.57167506	Yes
**206027_at**	S100A3	S100 calcium binding protein A3	1705	0.15203261375427246	0.572107	Yes
**209047_at**	AQP1	aquaporin 1 (Colton blood group)	1707	0.15190236270427704	0.57282585	Yes
**217599_s_at**	MDFIC	MyoD family inhibitor domain containing	1711	0.15174616873264313	0.5734481	Yes

**201098_at**	COPB2	coatomer protein complex, subunit beta 2 (beta prime)	1713	0.15157733857631683	0.57416534	Yes
**201044_x_at**	DUSP1	dual specificity phosphatase 1	1715	0.15151840448379517	0.5748822	Yes
**206087_x_at**	HFE	hemochromatosis	1735	0.14993061125278473	0.57472855	Yes
**206348_s_at**	PDK3	pyruvate dehydrogenase kinase, isozyme 3	1743	0.14959388971328735	0.5751482	Yes
**201939_at**	PLK2	polo-like kinase 2 (Drosophila)	1746	0.14940042793750763	0.5758065	Yes
**203068_at**	KLHL21	kelch-like 21 (Drosophila)	1749	0.14928844571113586	0.57646424	Yes
**210987_x_at**	TPM1	tropomyosin 1 (alpha)	1755	0.14916706085205078	0.5769776	Yes
**204984_at**	GPC4	glypican 4	1762	0.14833179116249084	0.57743883	Yes
**204105_s_at**	NRCAM	neuronal cell adhesion molecule	1767	0.14807063341140747	0.5779946	Yes
**203745_at**	HCCS	holocytochrome c synthase (cytochrome c heme-lyase)	1796	0.1466253250837326	0.5773929	Yes
**204983_s_at**	GPC4	glypican 4	1799	0.1465141624212265	0.5780366	Yes
**209514_s_at**	RAB27A	RAB27A, member RAS oncogene family	1802	0.1464088410139084	0.5786798	Yes
**209941_at**	RIPK1	receptor (TNFRSF)-interacting serine-threonine kinase 1	1808	0.1462850719690323	0.57917863	Yes
**201042_at**	TGM2	transglutaminase 2 (C polypeptide, protein-glutamine-gamma-glutamyltransferase)	1818	0.14525561034679413	0.5794805	Yes
**204806_x_at**	HLA-F	major histocompatibility complex, class I, F	1819	0.14520053565502167	0.5802135	Yes
**212188_at**	KCTD12	potassium channel tetramerisation domain containing 12 /// potassium channel tetramerisation domain containing 12	1825	0.14472492039203644	0.58070445	Yes
**205505_at**	GCNT1	glucosaminyl (N-acetyl) transferase 1, core 2 (beta-1,6-N-acetylglucosaminyltransferase)	1831	0.14463010430335999	0.5811949	Yes
**218975_at**	COL5A3	collagen, type V, alpha 3	1841	0.1440911740064621	0.58149093	Yes
**203767_s_at**	STS	steroid sulfatase (microsomal), arylsulfatase C, isozyme S	1842	0.14403313398361206	0.582218	Yes
**213506_at**	F2RL1	coagulation factor II (thrombin) receptor-like 1	1846	0.14350001513957977	0.5827986	Yes
**208757_at**	TMED9	transmembrane emp24 protein transport domain containing 9	1855	0.14292097091674805	0.5831367	Yes
**200845_s_at**	PRDX6	peroxiredoxin 6	1857	0.14272058010101318	0.5838092	Yes
**221875_x_at**	HLA-F	major histocompatibility complex, class I, F	1870	0.14204172790050507	0.5839511	Yes
**208967_s_at**	AK2	adenylate kinase 2	1879	0.1417606621980667	0.5842833	Yes
**204479_at**	OSTF1	osteoclast stimulating factor 1	1902	0.14068853855133057	0.5839392	Yes
**204470_at**	CXCL1	chemokine (C-X-C motif) ligand 1 (melanoma growth stimulating activity, alpha)	1906	0.14048458635807037	0.58450454	Yes
**211529_x_at**	HLA-G	HLA-G histocompatibility antigen, class I, G	1907	0.1404208093881607	0.58521336	Yes

**205483_s_at**	ISG15	ISG15 ubiquitin-like modifier	1909	0.14032961428165436	0.58587384	Yes
**210514_x_at**	HLA-G	HLA-G histocompatibility antigen, class I, G	1933	0.1389748603105545	0.5854731	Yes
**216959_x_at**	NRCAM	neuronal cell adhesion molecule	1947	0.13816747069358826	0.58554757	Yes
**202422_s_at**	ACSL4	acyl-CoA synthetase long-chain family member 4	1962	0.13685345649719238	0.5855675	Yes
**200844_s_at**	PRDX6	peroxiredoxin 6	1963	0.13682985305786133	0.5862582	Yes
**213287_s_at**	KRT10	keratin 10 (epidermolytic hyperkeratosis; keratosis palmaris et plantaris)	1967	0.1366204023361206	0.58680403	Yes
**213110_s_at**	COL4A5	collagen, type IV, alpha 5 (Alport syndrome)	1969	0.13647179305553436	0.587445	Yes
**211528_x_at**	HLA-G	HLA-G histocompatibility antigen, class I, G	1975	0.13635732233524323	0.5878937	Yes
**209740_s_at**	PNPLA4	patatin-like phospholipase domain containing 4	1985	0.13587291538715363	0.5881483	Yes
**221156_x_at**	CCPG1	cell cycle progression 1	2001	0.1354265958070755	0.58811307	Yes
**208812_x_at**	HLA-C	major histocompatibility complex, class I, C	2007	0.1352778673171997	0.5885563	Yes
**201749_at**	ECE1	Endothelin converting enzyme 1	2031	0.1340225338935852	0.5881306	Yes
**221196_x_at**	BRCC3	BRCA1/BRCA2-containing complex, subunit 3	2039	0.13383826613426208	0.58847076	Yes
**1729_at**	TRADD	TNFRSF1A-associated via death domain	2049	0.1333545446395874	0.5887126	Yes
**201444_s_at**	ATP6AP2	ATPase, H+ transporting, lysosomal accessory protein 2	2053	0.13314053416252136	0.5892409	Yes
**208829_at**	TAPBP	TAP binding protein (tapasin)	2055	0.13311611115932465	0.5898649	Yes
**216609_at**	TXN	Thioredoxin	2073	0.13200226426124573	0.58971655	Yes
**202994_s_at**	FBLN1	fibulin 1	2086	0.13138388097286224	0.5898047	Yes
**203312_x_at**	ARF6	ADP-ribosylation factor 6	2088	0.13133367896080017	0.5904197	Yes
**214087_s_at**	MYBPC1	myosin binding protein C, slow type	2093	0.13088032603263855	0.5908887	Yes
**205848_at**	GAS2	growth arrest-specific 2	2095	0.13081952929496765	0.5915011	Yes
**221510_s_at**	GLS	glutaminase	2099	0.13066431879997253	0.59201694	Yes
**206295_at**	IL18	interleukin 18 (interferon-gamma-inducing factor)	2101	0.13047900795936584	0.59262764	Yes
**209193_at**	PIM1	pim-1 oncogene /// pim-1 oncogene	2116	0.12991943955421448	0.59261256	Yes
**218853_s_at**	MOSPD1	motile sperm domain containing 1	2120	0.12970218062400818	0.5931235	Yes
**200904_at**	HLA-E	major histocompatibility complex, class I, E	2136	0.12896126508712769	0.59305567	Yes
**204797_s_at**	EML1	echinoderm microtubule associated protein like 1	2140	0.12854410707950592	0.59356076	Yes
**213422_s_at**	MXRA8	matrix-remodelling associated 8	2148	0.1283198893070221	0.593873	Yes
**210993_s_at**	SMAD1	SMAD, mothers against DPP homolog 1 (Drosophila)	2152	0.1281823366880417	0.5943763	Yes
**35820_at**	GM2A	GM2 ganglioside activator	2154	0.12809701263904572	0.594975	Yes
**200654_at**	P4HB	procollagen-proline, 2-oxoglutarate 4-dioxygenase (proline 4-hydroxylase), beta polypeptide	2161	0.1278349608182907	0.59533274	Yes

**208075_s_at**	CCL7	chemokine (C-C motif) ligand 7 /// chemokine (C-C motif) ligand 7	2168	0.1274275779724121	0.59568846	Yes
**206932_at**	CH25H	cholesterol 25-hydroxylase	2177	0.1268654316663742	0.5959455	Yes
**219038_at**	MORC4	MORC family CW-type zinc finger 4	2186	0.1262851059436798	0.5961996	Yes
**204058_at**	ME1	Malic enzyme 1, NADP(+)-dependent, cytosolic	2199	0.12595659494400024	0.5962603	Yes
**214866_at**	PLAUR	plasminogen activator, urokinase receptor	2200	0.12594032287597656	0.596896	Yes
**204429_s_at**	SLC2A5	solute carrier family 2 (facilitated glucose/fructose transporter), member 5	2210	0.12523294985294342	0.59709686	Yes
**212658_at**	LHFPL2	lipoma HMGIC fusion partner-like 2	2217	0.12495478987693787	0.59744006	Yes
**202239_at**	PARP4	poly (ADP-ribose) polymerase family, member 4	2220	0.12481778860092163	0.5979743	Yes
**201097_s_at**	ARF4	ADP-ribosylation factor 4	2236	0.12424879521131516	0.5978826	Yes
**204401_at**	KCNN4	potassium intermediate/small conductance calcium-activated channel, subfamily N, member 4	2249	0.12371709942817688	0.5979321	Yes
**204052_s_at**	SFRP4	secreted frizzled-related protein 4	2262	0.1231844425201416	0.59797883	Yes
**204526_s_at**	TBC1D8	TBC1 domain family, member 8 (with GRAM domain)	2267	0.1228715181350708	0.5984074	Yes
**215101_s_at**	CXCL5	chemokine (C-X-C motif) ligand 5	2270	0.12271863222122192	0.598931	Yes
**219500_at**	CLCF1	cardiotrophin-like cytokine factor 1	2276	0.1226007491350174	0.5993102	Yes
**212713_at**	MFAP4	microfibrillar-associated protein 4	2282	0.12239795178174973	0.5996885	Yes
**212175_s_at**	AK2	adenylate kinase 2	2288	0.12211199849843979	0.6000653	Yes
**209318_x_at**	PLAGL1	pleiomorphic adenoma gene-like 1	2292	0.12187694758176804	0.6005367	Yes
**218066_at**	SLC12A7	solute carrier family 12 (potassium/chloride transporters), member 7	2294	0.12173415720462799	0.6011033	Yes
**221269_s_at**	SH3BGR L3	SH3 domain binding glutamic acid-rich protein like 3 /// SH3 domain binding glutamic acid-rich protein like 3	2305	0.12126387655735016	0.60123616	Yes
**201278_at**	DAB2	Disabled homolog 2, mitogen-responsive phosphoprotein (Drosophila)	2307	0.12099678814411163	0.601799	Yes
**209140_x_at**	HLA-B	major histocompatibility complex, class I, B	2313	0.12089912593364716	0.6021697	Yes
**215313_x_at**	HLA-A	major histocompatibility complex, class I, A	2344	0.1191272884607315	0.6013334	Yes
**209016_s_at**	KRT7	keratin 7	2353	0.1189303770661354	0.60155034	Yes
**219593_at**	SLC15A3	solute carrier family 15, member 3	2358	0.1186329647898674	0.6019575	Yes
**214211_at**	FTH1	ferritin, heavy polypeptide 1	2361	0.11857882887125015	0.6024602	Yes
**206304_at**	MYBPH	myosin binding protein H	2363	0.11847727745771408	0.60301036	Yes
**218530_at**	FHOD1	formin homology 2 domain containing 1	2367	0.11830638349056244	0.60346377	Yes
**39402_at**	IL1B	interleukin 1, beta	2372	0.11815137416124344	0.6038685	Yes

**214446_at**	ELL2	elongation factor, RNA polymerase II, 2	2373	0.11808978766202927	0.6044646	Yes
**203889_at**	SCG5	secretogranin V (7B2 protein)	2382	0.117581807076931	0.6046747	Yes
**202996_at**	POLD4	polymerase (DNA-directed), delta 4	2389	0.11729297041893005	0.6049793	Yes
**212613_at**	BTN3A2	butyrophilin, subfamily 3, member A2	2390	0.11727946251630783	0.60557127	Yes
**205996_s_at**	AK2	adenylate kinase 2	2391	0.11724542081356049	0.6061631	Yes
**202205_at**	VASP	vasodilator-stimulated phosphoprotein	2420	0.11586235463619232	0.60540617	Yes
**203716_s_at**	DPP4	dipeptidyl-peptidase 4 (CD26, adenosine deaminase complexing protein 2)	2432	0.11554054915904999	0.6054622	Yes
**214459_x_at**	HLA-C	major histocompatibility complex, class I, C	2433	0.11552996188402176	0.60604537	Yes
**209109_s_at**	TSPAN6	tetraspanin 6	2438	0.11540723592042923	0.60643625	Yes
**200748_s_at**	FTH1	ferritin, heavy polypeptide 1	2441	0.11502675712108612	0.6069211	Yes
**210151_s_at**	DYRK3	dual-specificity tyrosine-(Y)-phosphorylation regulated kinase 3	2455	0.11443948745727539	0.6068757	Yes
**211062_s_at**	CPZ	carboxypeptidase Z /// carboxypeptidase Z	2456	0.11443282663822174	0.60745335	Yes
**214329_x_at**	TNFSF10	tumor necrosis factor (ligand) superfamily, member 10 /// tumor necrosis factor (ligand) superfamily, member 10	2468	0.11403962224721909	0.60750186	Yes
**219677_at**	SPSB1	splA/ryanodine receptor domain and SOCS box containing 1	2477	0.11376414448022842	0.6076928	Yes
**200905_x_at**	HLA-E	major histocompatibility complex, class I, E	2485	0.1133526936173439	0.60792947	Yes
**205805_s_at**	ROR1	receptor tyrosine kinase-like orphan receptor 1	2488	0.11323650926351547	0.60840523	Yes
**202995_s_at**	FBLN1	fibulin 1	2491	0.11315673589706421	0.6088806	Yes
**205756_s_at**	F8	coagulation factor VIII, procoagulant component (hemophilia A)	2513	0.11226173490285873	0.6084409	Yes
**213164_at**	SLC5A3	solute carrier family 5 (inositol transporters), member 3	2517	0.11217115819454193	0.60886335	Yes
**209206_at**	SEC22B	SEC22 vesicle trafficking protein homolog B (S. cerevisiae)	2532	0.1116860955953598	0.60875624	Yes
**216504_s_at**	SLC39A8	Solute carrier family 39 (zinc transporter), member 8	2536	0.11147988587617874	0.6091752	Yes
**222263_at**	SLC35E1	solute carrier family 35, member E1	2545	0.11110945045948029	0.60935265	Yes
**212737_at**	GM2A	GM2 ganglioside activator	2546	0.11105906218290329	0.6099133	Yes
**200837_at**	BCAP31	B-cell receptor-associated protein 31	2557	0.1106124222278595	0.6099924	Yes
**209405_s_at**	FAM3A	family with sequence similarity 3, member A	2565	0.11027105897665024	0.6102136	Yes
**215485_s_at**	ICAM1	intercellular adhesion molecule 1 (CD54), human rhinovirus receptor	2568	0.11024008691310883	0.6106742	Yes
**201331_s_at**	STAT6	signal transducer and activator of transcription 6, interleukin-4 induced	2571	0.11013887077569962	0.61113435	Yes

**213290_at**	COL6A2	collagen, type VI, alpha 2	2583	0.10965346544981003	0.6111607	Yes
**212089_at**	LMNA	lamin A/C	2587	0.10956915467977524	0.61157	Yes
**201611_s_at**	ICMT	isoprenylcysteine carboxyl methyltransferase	2588	0.10956218838691711	0.6121231	Yes
**206247_at**	MICB	MHC class I polypeptide-related sequence B	2602	0.10884901881217957	0.6120495	Yes
**212590_at**	RRAS2	related RAS viral (r-ras) oncogene homolog 2	2609	0.10865134745836258	0.61231047	Yes
**216235_s_at**	EDNRA	endothelin receptor type A	2619	0.10840295255184174	0.61242634	Yes
**217456_x_at**	HLA-E	major histocompatibility complex, class I, E	2621	0.10821345448493958	0.61292464	Yes
**214152_at**	CCPG1	cell cycle progression 1	2647	0.10712376981973648	0.6122673	Yes
**209546_s_at**	APOL1	apolipoprotein L, 1	2687	0.10540086030960083	0.6109304	Yes
**203746_s_at**	HCCS	holocytochrome c synthase (cytochrome c heme-lyase)	2693	0.10507393628358841	0.6112212	Yes
**208837_at**	TMED3	transmembrane emp24 protein transport domain containing 3	2695	0.10496263206005096	0.6117031	Yes
**221957_at**	PDK3	Pyruvate dehydrogenase kinase, isozyme 3	2700	0.10474395751953125	0.61204016	Yes
**209073_s_at**	NUMB	numb homolog (Drosophila)	2704	0.1045282706618309	0.612424	Yes
**201811_x_at**	SH3BP5	SH3-domain binding protein 5 (BTK-associated)	2731	0.10388379544019699	0.61170244	Yes
**204044_at**	QPRT	quinolinate phosphoribosyltransferase (nicotinate-nucleotide pyrophosphorylase (carboxylating))	2735	0.10367568582296371	0.612082	Yes
**214594_x_at**	ATP8B1	ATPase, Class I, type 8B, member 1	2757	0.10299018770456314	0.6115955	Yes
**214257_s_at**	SEC22B	SEC22 vesicle trafficking protein homolog B (S. cerevisiae)	2779	0.10205946862697601	0.6111043	Yes
**213187_x_at**	FTL	ferritin, light polypeptide	2791	0.10171438753604889	0.6110906	Yes
**221697_at**	MAP1LC 3C	microtubule-associated protein 1 light chain 3 gamma /// microtubule-associated protein 1 light chain 3 gamma	2824	0.1002219095826149	0.61006296	Yes
**213992_at**	COL4A6	collagen, type IV, alpha 6	2849	0.09906280785799026	0.6094129	Yes
**202110_at**	COX7B	cytochrome c oxidase subunit VIIb	2850	0.09905058145523071	0.6099129	Yes
**205891_at**	ADORA2 B	adenosine A2b receptor	2853	0.09897909313440323	0.6103167	Yes
**208788_at**	ELOVL5	ELOVL family member 5, elongation of long chain fatty acids (FEN1/Elo2, SUR4/Elo3-like, yeast)	2857	0.09867806732654572	0.610671	Yes
**201019_s_at**	EIF1AX	eukaryotic translation initiation factor 1A, X-linked	2862	0.09856157004833221	0.6109768	Yes
**211330_s_at**	HFE	hemochromatosis	2863	0.09848003089427948	0.611474	Yes
**209543_s_at**	CD34	CD34 molecule	2878	0.09762822091579437	0.6112959	Yes
**214282_at**	CP	Ceruloplasmin (ferroxidase)	2879	0.0975349172949791	0.6117882	Yes
**208683_at**	CAPN2	calpain 2, (m/II) large subunit	2880	0.09746172279119492	0.6122802	Yes
**218881_s_at**	FOSL2	FOS-like antigen 2	2886	0.09719928354024887	0.6125312	Yes

**212938_at**	COL6A1	collagen, type VI, alpha 1	2889	0.09706426411867142	0.6129253	Yes
**219352_at**	HERC6	hect domain and RLD 6	2899	0.09662723541259766	0.6129818	Yes
**201095_at**	DAP	death-associated protein	2903	0.09649962186813354	0.6133251	Yes
**218398_at**	MRPS30	mitochondrial ribosomal protein S30	2911	0.09630092233419418	0.6134758	Yes
**203738_at**	C5ORF22	chromosome 5 open reading frame 22	2915	0.09613059461116791	0.6138173	Yes
**203710_at**	ITPR1	inositol 1,4,5-triphosphate receptor, type 1	2916	0.09608488529920578	0.6143023	Yes
**209799_at**	PRKAA1	protein kinase, AMP-activated, alpha 1 catalytic subunit	2940	0.09495516121387482	0.6136794	Yes
**205067_at**	IL1B	interleukin 1, beta	2964	0.09418121725320816	0.61305255	Yes
**213126_at**	MED8	mediator of RNA polymerase II transcription, subunit 8 homolog (S. cerevisiae)	2967	0.09413379430770874	0.61343193	Yes
**204867_at**	GCHFR	GTP cyclohydrolase I feedback regulator	2972	0.09399379044771194	0.6137147	Yes
**207542_s_at**	AQP1	aquaporin 1 (Colton blood group)	2973	0.09386689960956573	0.6141885	Yes
**211329_x_at**	HFE	hemochromatosis	2993	0.09319674223661423	0.61374843	Yes
**205263_at**	BCL10	B-cell CLL/lymphoma 10	2999	0.0929415300488472	0.61397797	Yes
**213696_s_at**	MED8	mediator of RNA polymerase II transcription, subunit 8 homolog (S. cerevisiae)	3002	0.09281966090202332	0.6143507	Yes
**208300_at**	PTPRH	protein tyrosine phosphatase, receptor type, H	3031	0.09187536686658859	0.6134726	Yes
**205315_s_at**	SNTB2	syntrophin, beta 2 (dystrophin-associated protein A1, 59kDa, basic component 2)	3037	0.09173069894313812	0.61369604	Yes
**207023_x_at**	KRT10	keratin 10 (epidermolytic hyperkeratosis; keratosis palmaris et plantaris)	3040	0.09169258177280426	0.614063	Yes
**212944_at**	MRPS6	Mitochondrial ribosomal protein S6	3048	0.09154625982046127	0.6141897	Yes
**201263_at**	TARS	threonyl-tRNA synthetase	3092	0.09020108729600906	0.61258435	Yes
**203988_s_at**	FUT8	fucosyltransferase 8 (alpha (1,6) fucosyltransferase)	3097	0.09011471271514893	0.6128475	Yes
**206693_at**	IL7	interleukin 7	3098	0.09010353684425354	0.61330235	Yes
**200075_s_at**	GUK1	guanylate kinase 1 /// guanylate kinase 1	3100	0.0900416374206543	0.613709	Yes
**201953_at**	CIB1	calcium and integrin binding 1 (calmyrin)	3101	0.0899982899427414	0.6141632	Yes
**202273_at**	PDGFRB	platelet-derived growth factor receptor, beta polypeptide	3104	0.08992629498243332	0.6145213	Yes
**204821_at**	BTN3A3	butyrophilin, subfamily 3, member A3	3107	0.08985371887683868	0.6148791	Yes
**202686_s_at**	AXL	AXL receptor tyrosine kinase	3113	0.08957750350236893	0.6150916	Yes
**214647_s_at**	HFE	hemochromatosis	3116	0.08941178023815155	0.6154471	Yes
**218451_at**	CDCP1	CUB domain containing protein 1	3143	0.08876475691795349	0.6146492	No
**203262_s_at**	FAM50A	family with sequence similarity 50, member A	3168	0.0881650522351265	0.6139441	No
**214799_at**	NFASC	Neurofascin homolog (chicken)	3219	0.08690685033798218	0.61198664	No

**217756_x_at**	SERF2	small EDRK-rich factor 2	3224	0.08680178225040436	0.6122331	No
**201634_s_at**	CYB5B	cytochrome b5 type B (outer mitochondrial membrane)	3240	0.0864139050245285	0.6119505	No
**211327_x_at**	HFE	hemochromatosis	3242	0.08635925501585007	0.6123385	No
**38241_at**	BTN3A3	butyrophilin, subfamily 3, member A3	3259	0.08585535734891891	0.6120051	No
**203879_at**	PIK3CD	phosphoinositide-3-kinase, catalytic, delta polypeptide /// phosphoinositide-3-kinase, catalytic, delta polypeptide	3278	0.08541784435510635	0.6115737	No
**202788_at**	MAPKAP K3	mitogen-activated protein kinase-activated protein kinase 3	3282	0.08530319482088089	0.6118605	No
**216092_s_at**	SLC7A8	solute carrier family 7 (cationic amino acid transporter, y+ system), member 8	3287	0.08515498787164688	0.6120987	No
**203236_s_at**	LGALS9	lectin, galactoside-binding, soluble, 9 (galectin 9)	3292	0.08497462421655655	0.6123359	No
**205842_s_at**	JAK2	Janus kinase 2 (a protein tyrosine kinase)	3330	0.08362860977649689	0.6109849	No
**217383_at**	PGK1	Phosphoglycerate kinase 1	3331	0.08362305909395218	0.61140704	No
**208180_s_at**	HIST1H4 H	histone 1, H4h	3339	0.0834837332367897	0.611493	No
**203490_at**	ELF4	E74-like factor 4 (ets domain transcription factor)	3352	0.0831630527973175	0.6113377	No
**203840_at**	BLZF1	basic leucine zipper nuclear factor 1 (JEM-1)	3371	0.08274894207715988	0.61089283	No
**203518_at**	LYST	lysosomal trafficking regulator	3382	0.08254162222146988	0.61083025	No
**211326_x_at**	HFE	hemochromatosis	3386	0.08249762654304504	0.61110294	No
**201266_at**	TXNRD1	thioredoxin reductase 1	3395	0.08221176266670227	0.6111345	No
**213787_s_at**	EBP	emopamil binding protein (sterol isomerase)	3397	0.08219516277313232	0.6115015	No
**213112_s_at**	SQSTM1	sequestosome 1	3406	0.08208836615085602	0.6115325	No
**202752_x_at**	SLC7A8	solute carrier family 7 (cationic amino acid transporter, y+ system), member 8	3410	0.0819641575217247	0.6118025	No
**211920_at**	CFB	complement factor B /// complement factor B	3422	0.08166435360908508	0.6116876	No
**212788_x_at**	FTL	ferritin, light polypeptide	3434	0.08132059872150421	0.6115709	No
**208916_at**	SLC1A5	solute carrier family 1 (neutral amino acid transporter), member 5	3444	0.08093670755624771	0.6115482	No
**221978_at**	HLA-F	major histocompatibility complex, class I, F	3446	0.08081862330436707	0.6119082	No
**201280_s_at**	DAB2	disabled homolog 2, mitogen-responsive phosphoprotein (Drosophila)	3451	0.08059244602918625	0.6121233	No
**201201_at**	CSTB	cystatin B (stefin B)	3458	0.08029408007860184	0.6122411	No
**201460_at**	MAPKAP K2	mitogen-activated protein kinase-activated protein kinase 2	3465	0.0799781009554863	0.6123573	No
**201980_s_at**	RSU1	Ras suppressor protein 1	3468	0.07994658499956131	0.612665	No
**203605_at**	SRP54	signal recognition particle 54kDa	3494	0.07930387556552887	0.61186725	No

**218088_s_at**	RRAGC	Ras-related GTP binding C	3503	0.0790247842669487	0.6118828	No
**210628_x_at**	LTBP4	latent transforming growth factor beta binding protein 4	3504	0.07900875061750412	0.6122816	No
**207438_s_at**	SNUPN	snurportin 1	3505	0.07897774875164032	0.61268026	No
**217826_s_at**	UBE2J1	ubiquitin-conjugating enzyme E2, J1 (UBC6 homolog, yeast)	3509	0.07875155657529831	0.61293405	No
**219687_at**	HHAT	hedgehog acyltransferase	3512	0.07864703983068466	0.6132352	No
**210757_x_at**	DAB2	disabled homolog 2, mitogen-responsive phosphoprotein (Drosophila)	3523	0.07833681255578995	0.6131514	No
**213865_at**	DCBLD2	discoidin, CUB and LCCL domain containing 2	3551	0.07742627710103989	0.6122483	No
**202675_at**	SDHB	succinate dehydrogenase complex, subunit B, iron sulfur (Ip)	3573	0.0768105685710907	0.61162966	No
**202324_s_at**	ACBD3	acyl-Coenzyme A binding domain containing 3	3579	0.0767592042684555	0.61177754	No
**217168_s_at**	HERPUD 1	homocysteine-inducible, endoplasmic reticulum stress-inducible, ubiquitin-like domain member 1	3607	0.07589632272720337	0.6108667	No
**214182_at**	ARF6	ADP-ribosylation factor 6	3647	0.07455266267061234	0.6093741	No
**202974_at**	MPP1	membrane protein, palmitoylated 1, 55kDa	3653	0.074372798204422	0.6095099	No
**217769_s_at**	POMP	proteasome maturation protein	3654	0.07432736456394196	0.6098851	No
**202298_at**	NDUFA1	NADH dehydrogenase (ubiquinone) 1 alpha subcomplex, 1, 7.5kDa	3661	0.07416867464780807	0.60997194	No
**213910_at**	IGFBP7	insulin-like growth factor binding protein 7	3668	0.07402542233467102	0.61005807	No
**38043_at**	FAM3A	family with sequence similarity 3, member A	3678	0.07370570302009583	0.6099988	No
**218834_s_at**	TMEM13 2A	transmembrane protein 132A	3680	0.07365869730710983	0.6103227	No
**206756_at**	CHST7	carbohydrate (N-acetylglucosamine 6-O) sulfotransferase 7	3689	0.0734015479683876	0.61030984	No
**217678_at**	SLC7A11	solute carrier family 7, (cationic amino acid transporter, y+ system) member 11	3698	0.0731629952788353	0.6102958	No
**205153_s_at**	CD40	CD40 molecule, TNF receptor superfamily member 5	3725	0.07239077240228653	0.60941523	No
**216526_x_at**	HLA-C	major histocompatibility complex, class I, C	3759	0.07173697650432587	0.6081959	No
**214660_at**	PELO	Pelota homolog (Drosophila)	3765	0.07158398628234863	0.6083176	No
**216231_s_at**	B2M	beta-2-microglobulin	3781	0.07117097079753876	0.6079581	No
**202768_at**	FOSB	FBJ murine osteosarcoma viral oncogene homolog B	3786	0.07106613367795944	0.6081251	No
**214151_s_at**	CCPG1	cell cycle progression 1	3795	0.07087517529726028	0.60809946	No
**211328_x_at**	HFE	hemochromatosis	3796	0.07087451219558716	0.60845727	No
**218495_at**	UXT	ubiquitously-expressed transcript	3801	0.07078284025192261	0.60862285	No
**201605_x_at**	CNN2	calponin 2	3810	0.0705334022641182	0.6085955	No

**209453_at**	SLC9A1	solute carrier family 9 (sodium/hydrogen exchanger), member 1 (antiporter, Na+/H+, amiloride sensitive)	3815	0.07042687386274338	0.60875934	No
**212224_at**	ALDH1A1	aldehyde dehydrogenase 1 family, member A1	3820	0.07037621736526489	0.6089229	No
**202735_at**	EBP	emopamil binding protein (sterol isomerase)	3823	0.07030560076236725	0.60918194	No
**61734_at**	RCN3	reticulocalbin 3, EF-hand calcium binding domain	3839	0.06965174525976181	0.60881466	No
**202481_at**	DHRS3	dehydrogenase/reductase (SDR family) member 3	3840	0.06964036077260971	0.6091662	No
**213127_s_at**	MED8	mediator of RNA polymerase II transcription, subunit 8 homolog (S. cerevisiae)	3860	0.06906545162200928	0.6086043	No
**216944_s_at**	ITPR1	inositol 1,4,5-triphosphate receptor, type 1	3900	0.06790437549352646	0.60707814	No
**208502_s_at**	PITX1	paired-like homeodomain transcription factor 1	3908	0.06771963089704514	0.6070845	No
**206925_at**	ST8SIA4	ST8 alpha-N-acetyl-neuraminide alpha-2,8-sialyltransferase 4	3919	0.06750459223985672	0.606946	No
**207545_s_at**	NUMB	numb homolog (Drosophila)	3920	0.0674746036529541	0.60728663	No
**218669_at**	RAP2C	RAP2C, member of RAS oncogene family	3928	0.06721650063991547	0.60729045	No
**207439_s_at**	SLC35A2	solute carrier family 35 (UDP-galactose transporter), member A2	3942	0.06691169738769531	0.60700524	No
**219257_s_at**	SPHK1	sphingosine kinase 1	3943	0.06685485690832138	0.6073427	No
**207440_at**	SLC35A2	solute carrier family 35 (UDP-galactose transporter), member A2	3945	0.06680896133184433	0.60763204	No
**209716_at**	CSF1	colony stimulating factor 1 (macrophage)	3951	0.0666527971625328	0.60772884	No
**202997_s_at**	LOXL2	lysyl oxidase-like 2	3962	0.06646708399057388	0.60758513	No
**204587_at**	SLC25A14	solute carrier family 25 (mitochondrial carrier, brain), member 14	3969	0.06628520041704178	0.6076322	No
**221563_at**	DUSP10	dual specificity phosphatase 10	3981	0.06593526154756546	0.6074379	No
**202514_at**	DLG1	discs, large homolog 1 (Drosophila)	4002	0.06554760783910751	0.60681033	No
**217208_s_at**	DLG1	discs, large homolog 1 (Drosophila)	4034	0.06500519812107086	0.60565287	No
**203585_at**	ZNF185	zinc finger protein 185 (LIM domain)	4036	0.06497542560100555	0.6059329	No
**207002_s_at**	PLAGL1	pleiomorphic adenoma gene-like 1	4037	0.06496132165193558	0.60626084	No
**205743_at**	STAC	SH3 and cysteine rich domain	4049	0.06460411846637726	0.6060598	No
**202787_s_at**	MAPKAPK3	mitogen-activated protein kinase-activated protein kinase 3	4054	0.06447380036115646	0.60619354	No
**214927_at**	ITGBL1	Integrin, beta-like 1 (with EGF-like repeat domains)	4062	0.06416531652212143	0.606182	No
**206561_s_at**	AKR1B10	aldo-keto reductase family 1, member B10 (aldose reductase)	4079	0.06369069963693619	0.60573673	No
**211530_x_at**	HLA-G	HLA-G histocompatibility antigen, class I, G	4090	0.06348219513893127	0.60557795	No

**214838_at**	SFT2D2	SFT2 domain containing 2	4095	0.06329885125160217	0.6057058	No
**209906_at**	C3AR1	complement component 3a receptor 1	4097	0.06324827671051025	0.6059771	No
**205503_at**	PTPN14	protein tyrosine phosphatase, non-receptor type 14	4099	0.06314267218112946	0.60624796	No
**204796_at**	EML1	echinoderm microtubule associated protein like 1	4108	0.06278528273105621	0.6061815	No
**201279_s_at**	DAB2	disabled homolog 2, mitogen-responsive phosphoprotein (Drosophila)	4117	0.06266304850578308	0.60611445	No
**217430_x_at**	COL1A1	collagen, type I, alpha 1	4125	0.0623873732984066	0.6060939	No
**222156_x_at**	CCPG1	cell cycle progression 1	4129	0.06228366121649742	0.60626453	No
**201427_s_at**	SEPP1	selenoprotein P, plasma, 1	4133	0.06220865622162819	0.60643476	No
**203200_s_at**	MTRR	5-methyltetrahydrofolate-homocysteine methyltransferase reductase	4152	0.061844415962696075	0.6058844	No
**203054_s_at**	TCTA	T-cell leukemia translocation altered gene	4162	0.06167559325695038	0.6057644	No
**216178_x_at**	ITGB1	integrin, beta 1 (fibronectin receptor, beta polypeptide, antigen CD29 includes MDF2, MSK12)	4170	0.06143248826265335	0.605739	No
**215050_x_at**	MAPKAP K2	mitogen-activated protein kinase-activated protein kinase 2	4174	0.06138215214014053	0.6059051	No
**201107_s_at**	THBS1	thrombospondin 1	4175	0.061346568167209625	0.60621476	No
**204769_s_at**	TAP2	transporter 2, ATP-binding cassette, sub-family B (MDR/TAP)	4189	0.060981035232543945	0.6058996	No
**219412_at**	RAB38	RAB38, member RAS oncogene family	4191	0.06097101792693138	0.60615945	No
**203881_s_at**	DMD	dystrophin (muscular dystrophy, Duchenne and Becker types)	4195	0.06090975180268288	0.6063231	No
**201900_s_at**	AKR1A1	aldo-keto reductase family 1, member A1 (aldehyde reductase)	4228	0.060216594487428665	0.6050936	No
**203781_at**	MRPL33	mitochondrial ribosomal protein L33	4238	0.06005951017141342	0.60496545	No
**219232_s_at**	EGLN3	egl nine homolog 3 (C. elegans)	4248	0.059945207089185715	0.60483676	No
**206116_s_at**	TPM1	tropomyosin 1 (alpha)	4251	0.059865400195121765	0.6050431	No
**214612_x_at**	MAGEA6	melanoma antigen family A, 6	4258	0.05957727134227753	0.6050563	No
**201004_at**	SSR4	signal sequence receptor, delta (translocon-associated protein delta)	4261	0.059537433087825775	0.60526097	No
**202275_at**	G6PD	glucose-6-phosphate dehydrogenase	4263	0.05952858924865723	0.6055136	No
**205324_s_at**	FTSJ1	FtsJ homolog 1 (E. coli)	4265	0.05949079245328903	0.60576594	No
**201891_s_at**	B2M	beta-2-microglobulin	4282	0.05912431329488754	0.6052976	No
**218361_at**	GOLPH3 L	golgi phosphoprotein 3-like	4295	0.058873843401670456	0.60501975	No
**209921_at**	SLC7A11	solute carrier family 7, (cationic amino acid transporter, y+ system) member 11	4311	0.05858634039759636	0.6045966	No

**209207_s_at**	SEC22B	SEC22 vesicle trafficking protein homolog B (S. cerevisiae)	4312	0.058579493314027786	0.6048923	No
**204555_s_at**	PPP1R3D	protein phosphatase 1, regulatory subunit 3D	4321	0.05844045802950859	0.6048039	No
**206347_at**	PDK3	pyruvate dehydrogenase kinase, isozyme 3	4334	0.05815544351935387	0.6045224	No
**219956_at**	GALNT6	UDP-N-acetyl-alpha-D-galactosamine:polypeptide N-acetylgalactosaminyltransferase 6 (GalNAc-T6)	4346	0.05787660554051399	0.60428745	No
**207442_at**	CSF3	colony stimulating factor 3 (granulocyte)	4347	0.0578647144138813	0.6045795	No
**214213_x_at**	LMNA	Lamin A/C	4351	0.057827625423669815	0.6047277	No
**203606_at**	NDUFS6	NADH dehydrogenase (ubiquinone) Fe-S protein 6, 13kDa (NADH-coenzyme Q reductase)	4356	0.0577520914375782	0.6048275	No
**209747_at**	TGFB3	transforming growth factor, beta 3	4364	0.05765688046813011	0.60478306	No
**205978_at**	KL	klotho	4365	0.057638660073280334	0.60507405	No
**220174_at**	LRRC8E	leucine rich repeat containing 8 family, member E	4375	0.05745959281921387	0.6049328	No
**217731_s_at**	ITM2B	integral membrane protein 2B	4377	0.057411856949329376	0.60517466	No
**211199_s_at**	ICOSLG	inducible T-cell co-stimulator ligand	4386	0.05717241019010544	0.6050799	No
**217732_s_at**	ITM2B	integral membrane protein 2B	4387	0.05714334920048714	0.6053683	No
**203700_s_at**	DIO2	deiodinase, iodothyronine, type II	4402	0.05681720748543739	0.6049842	No
**204628_s_at**	ITGB3	integrin, beta 3 (platelet glycoprotein IIIa, antigen CD61)	4421	0.056311894208192825	0.6044059	No
**207426_s_at**	TNFSF4	tumor necrosis factor (ligand) superfamily, member 4 (tax-transcriptionally activated glycoprotein 1, 34kDa)	4424	0.05621671304106712	0.6045938	No
**201750_s_at**	ECE1	endothelin converting enzyme 1	4426	0.05620323494076729	0.60482955	No
**219938_s_at**	PSTPIP2	proline-serine-threonine phosphatase interacting protein 2	4444	0.05591640621423721	0.60429716	No
**220419_s_at**	USP25	ubiquitin specific peptidase 25	4445	0.055863454937934875	0.60457915	No
**215346_at**	CD40	CD40 molecule, TNF receptor superfamily member 5	4455	0.05563119426369667	0.60442865	No
**212457_at**	TFE3	transcription factor binding to IGHM enhancer 3	4481	0.054973602294921875	0.6035081	No
**205244_s_at**	SLC13A3	solute carrier family 13 (sodium-dependent dicarboxylate transporter), member 3	4483	0.054944995790719986	0.60373753	No
**217823_s_at**	UBE2J1	ubiquitin-conjugating enzyme E2, J1 (UBC6 homolog, yeast)	4497	0.05465800687670708	0.60339046	No
**221895_at**	MOSPD2	motile sperm domain containing 2	4505	0.05454869195818901	0.6033303	No
**203308_x_at**	HPS1	Hermansky-Pudlak syndrome 1	4511	0.05446059629321098	0.6033656	No

**205767_at**	EREG	epiregulin	4525	0.05423455685377121	0.6030164	No
**204693_at**	CDC42EP 1	CDC42 effector protein (Rho GTPase binding) 1	4532	0.05400871858000755	0.6030015	No
**203530_s_at**	STX4	syntaxin 4	4543	0.05378833785653114	0.60279375	No
**211332_x_at**	HFE	hemochromatosis	4564	0.053477294743061066	0.60210526	No
**210118_s_at**	IL1A	interleukin 1, alpha	4578	0.05326449126005173	0.60175115	No
**213115_at**	ATG4A	ATG4 autophagy related 4 homolog A (S. cerevisiae)	4597	0.05301300808787346	0.6011562	No
**210362_x_at**	PML	promyelocytic leukemia	4612	0.052673205733299255	0.6007511	No
**202509_s_at**	TNFAIP2	tumor necrosis factor, alpha-induced protein 2	4625	0.05244458094239235	0.6004408	No
**214265_at**	ITGA8	integrin, alpha 8	4627	0.05239090323448181	0.60065734	No
**201312_s_at**	SH3BGR L	SH3 domain binding glutamic acid-rich protein like	4630	0.052344776690006256	0.6008257	No
**206515_at**	CYP4F3	cytochrome P450, family 4, subfamily F, polypeptide 3	4680	0.05147364363074303	0.5987373	No
**210633_x_at**	KRT10	keratin 10 (epidermolytic hyperkeratosis; keratosis palmaris et plantaris)	4690	0.0513596348464489	0.5985653	No
**211057_at**	ROR1	receptor tyrosine kinase-like orphan receptor 1 /// receptor tyrosine kinase-like orphan receptor 1	4693	0.05131056532263756	0.5987284	No
**219582_at**	OGFRL1	opioid growth factor receptor-like 1	4707	0.05092008039355278	0.5983625	No
**201100_s_at**	USP9X	ubiquitin specific peptidase 9, X-linked	4747	0.050083231180906296	0.5967463	No
**206553_at**	OAS2	2'-5'-oligoadenylate synthetase 2, 69/71kDa	4750	0.04999789595603943	0.59690285	No
**219221_at**	ZBTB38	zinc finger and BTB domain containing 38	4771	0.04954876750707626	0.5961945	No
**213765_at**	MFAP5	microfibrillar associated protein 5	4799	0.04903619736433029	0.59514815	No
**206649_s_at**	TFE3	transcription factor binding to IGHM enhancer 3	4818	0.04854581877589226	0.5945306	No
**203032_s_at**	FH	fumarate hydratase	4841	0.04816930368542671	0.5937194	No
**216521_s_at**	BRCC3	BRCA1/BRCA2-containing complex, subunit 3	4862	0.04785769432783127	0.59300256	No
**222314_x_at**	ITPR1	Inositol 1,4,5-triphosphate receptor, type 1	4870	0.04769682139158249	0.59290785	No
**216322_at**	CD58	CD58 molecule	4883	0.04740126430988312	0.5925721	No
**204789_at**	FMNL1	formin-like 1	4884	0.04739799350500107	0.59281135	No
**206550_s_at**	NUP155	nucleoporin 155kDa	4930	0.04663495719432831	0.5908902	No
**213277_at**	ZFP36L1	Zinc finger protein 36, C3H type-like 1	4932	0.04655728489160538	0.5910773	No
**219102_at**	RCN3	reticulocalbin 3, EF-hand calcium binding domain	4941	0.04630504921078682	0.59092766	No
**204875_s_at**	GMDS	GDP-mannose 4,6-dehydratase	4953	0.046108976006507874	0.5906333	No
**205991_s_at**	PRRX1	paired related homeobox 1	4958	0.04604503512382507	0.59067404	No
**215243_s_at**	GJB3	gap junction protein, beta 3, 31kDa (connexin 31)	4972	0.04586764797568321	0.59028256	No
**209266_s_at**	SLC39A8	solute carrier family 39 (zinc transporter), member 8	4981	0.045637886971235275	0.59012955	No
**214678_x_at**	ZFX	zinc finger protein, X-linked	4992	0.045418791472911835	0.5898796	No

**216250_s_at**	LPXN	leupaxin	5060	0.04435745254158974	0.58689266	No
**218241_at**	GOLGA5	golgi autoantigen, golgin subfamily a, 5	5086	0.04395060986280441	0.58591646	No
**203033_x_at**	FH	fumarate hydratase	5096	0.043749820441007614	0.585706	No
**215561_s_at**	IL1R1	interleukin 1 receptor, type I	5155	0.042823318392038345	0.5831427	No
**205660_at**	OASL	2'-5'-oligoadenylate synthetase-like	5161	0.042732857167720795	0.5831188	No
**207334_s_at**	TGFBR2	transforming growth factor, beta receptor II (70/80kDa)	5176	0.042487259954214096	0.58266234	No
**219360_s_at**	TRPM4	transient receptor potential cation channel, subfamily M, member 4	5192	0.042261552065610886	0.58215684	No
**205142_x_at**	ABCD1	ATP-binding cassette, sub-family D (ALD), member 1	5200	0.042172111570835114	0.58203423	No
**204043_at**	TCN2	transcobalamin II; macrocytic anemia	5243	0.0413975790143013	0.5802305	No
**201121_s_at**	PGRMC1	progesterone receptor membrane component 1	5267	0.040873389691114426	0.57933456	No
**203666_at**	CXCL12	chemokine (C-X-C motif) ligand 12 (stromal cell-derived factor 1)	5293	0.04044349119067192	0.57834065	No
**208684_at**	COPA	coatomer protein complex, subunit alpha	5296	0.040422871708869934	0.57844883	No
**217741_s_at**	ZFAND5	zinc finger, AN1-type domain 5	5313	0.040104761719703674	0.57788455	No
**202516_s_at**	DLG1	discs, large homolog 1 (Drosophila)	5345	0.039525505155324936	0.57659847	No
**220049_s_at**	PDCD1LG2	programmed cell death 1 ligand 2	5385	0.03901871293783188	0.57492644	No
**218810_at**	ZC3H12A	zinc finger CCCH-type containing 12A	5406	0.03868772089481354	0.57416326	No
**204442_x_at**	LTBP4	latent transforming growth factor beta binding protein 4	5413	0.03858977183699608	0.5740705	No
**218437_s_at**	LZTFL1	leucine zipper transcription factor-like 1	5415	0.03856944665312767	0.5742173	No
**215209_at**	SEC24D	SEC24 related gene family, member D (S. cerevisiae)	5420	0.038450032472610474	0.5742197	No
**210659_at**	CMKLR1	chemokine-like receptor 1	5428	0.03832940384745598	0.5740777	No
**213937_s_at**	FTSJ1	FtsJ homolog 1 (E. coli)	5429	0.038326334208250046	0.5742712	No
**201120_s_at**	PGRMC1	progesterone receptor membrane component 1	5436	0.038200944662094116	0.5741765	No
**213176_s_at**	LTBP4	latent transforming growth factor beta binding protein 4	5448	0.03808072954416275	0.5738416	No
**32088_at**	BLZF1	basic leucine zipper nuclear factor 1 (JEM-1)	5455	0.038003720343112946	0.57374585	No
**204461_x_at**	RAD1	RAD1 homolog (S. pombe)	5521	0.03718751668930054	0.5708186	No
**201015_s_at**	JUP	junction plakoglobin	5556	0.03664221614599228	0.5693742	No
**57540_at**	RBKS	ribokinase	5559	0.03658587858080864	0.5694631	No
**210166_at**	TLR5	toll-like receptor 5	5561	0.036552976816892624	0.5695996	No
**207038_at**	SLC16A6	solute carrier family 16 (monocarboxylic acid transporters), member 6	5570	0.03649606183171272	0.5694005	No
**211631_x_at**	B4GALT1	UDP-Gal:betaGlcNAc beta 1,4- galactosyltransferase, polypeptide 1 /// UDP-Gal:betaGlcNAc beta 1,4-	5602	0.03600631654262543	0.56809664	No

		galactosyltransferase, polypeptide 1				
**203789_s_at**	SEMA3C	sema domain, immunoglobulin domain (Ig), short basic domain, secreted, (semaphorin) 3C	5660	0.035209253430366516	0.56554276	No
**203417_at**	MFAP2	microfibrillar-associated protein 2	5673	0.03498823568224907	0.5651443	No
**221223_x_at**	CISH	cytokine inducible SH2-containing protein	5675	0.03493989631533623	0.56527275	No
**211863_x_at**	HFE	hemochromatosis	5683	0.034832097589969635	0.5651131	No
**213068_at**	DPT	dermatopontin	5697	0.03463871404528618	0.564665	No
**201612_at**	ALDH9A 1	aldehyde dehydrogenase 9 family, member A1	5707	0.034476086497306824	0.5644077	No
**212473_s_at**	MICAL2	microtubule associated monoxygenase, calponin and LIM domain containing 2	5713	0.03430015593767166	0.56434125	No
**210664_s_at**	TFPI	tissue factor pathway inhibitor (lipoprotein-associated coagulation inhibitor)	5716	0.0342230349779129	0.56441814	No
**201981_at**	PAPPA	pregnancy-associated plasma protein A, pappalysin 1	5742	0.03402267023921013	0.56339186	No
**203335_at**	PHYH	phytanoyl-CoA 2-hydroxylase	5787	0.033400364220142365	0.56145185	No
**215955_x_at**	ARHGAP 26	Rho GTPase activating protein 26	5822	0.033004071563482285	0.5599891	No
**202464_s_at**	PFKFB3	6-phosphofructo-2-kinase/fructose-2,6-biphosphatase 3	5829	0.032938841730356216	0.5598678	No
**209928_s_at**	MSC	musculin (activated B-cell factor-1)	5837	0.03279814496636391	0.5596979	No
**211331_x_at**	HFE	hemochromatosis	5841	0.03273756057024002	0.5597194	No
**204502_at**	SAMHD1	SAM domain and HD domain 1	5844	0.03268521651625633	0.5597885	No
**211866_x_at**	HFE	hemochromatosis	5848	0.03263439983129501	0.5598095	No
**208086_s_at**	DMD	dystrophin (muscular dystrophy, Duchenne and Becker types)	5890	0.032170820981264114	0.55800706	No
**204393_s_at**	ACPP	acid phosphatase, prostate	5897	0.03209609538316727	0.5578816	No
**201609_x_at**	ICMT	isoprenylcysteine carboxyl methyltransferase	5902	0.03199473023414612	0.5578514	No
**201945_at**	FURIN	furin (paired basic amino acid cleaving enzyme)	5966	0.031133875250816345	0.5549894	No
**202727_s_at**	IFNGR1	interferon gamma receptor 1	5971	0.031102431938052177	0.5549547	No
**201994_at**	MORF4L 2	mortality factor 4 like 2	5975	0.0310814306139946	0.5549679	No
**209894_at**	LEPR	leptin receptor	5993	0.03083476796746254	0.55430883	No
**214170_x_at**	FH	fumarate hydratase	6014	0.03056984581053257	0.5535047	No
**213813_x_at**	FTL	Ferritin, light polypeptide	6029	0.030399037525057793	0.5529872	No
**220585_at**	HKDC1	hexokinase domain containing 1	6050	0.030056817457079887	0.55218047	No
**210943_s_at**	LYST	lysosomal trafficking regulator	6075	0.029772983863949776	0.55118066	No
**206153_at**	CYP4F11	cytochrome P450, family 4, subfamily F, polypeptide 11	6082	0.029714442789554596	0.5510431	No

**201787_at**	FBLN1	fibulin 1	6085	0.02968067303299904	0.5510971	No
**207631_at**	NBR2	neighbor of BRCA1 gene 2	6108	0.029392175376415253	0.55019116	No
**221541_at**	CRISPLD 2	cysteine-rich secretory protein LCCL domain containing 2	6109	0.029381614178419113	0.55033946	No
**79005_at**	SLC35E1	solute carrier family 35, member E1	6116	0.029274702072143555	0.5501997	No
**204627_s_at**	ITGB3	integrin, beta 3 (platelet glycoprotein IIIa, antigen CD61)	6119	0.02918844483792782	0.5502512	No
**209770_at**	BTN3A1	butyrophilin, subfamily 3, member A1	6123	0.029173826798796654	0.5502547	No
**214024_s_at**	DGCR6L	DiGeorge syndrome critical region gene 6-like	6125	0.029151862487196922	0.55035394	No
**201185_at**	HTRA1	HtrA serine peptidase 1	6151	0.028831785544753075	0.5493014	No
**201851_at**	SH3GL1	SH3-domain GRB2-like 1	6165	0.028654292225837708	0.54882306	No
**209706_at**	NKX3-1	NK3 transcription factor related, locus 1 (Drosophila)	6193	0.028248583897948265	0.54767174	No
**206200_s_at**	ANXA11	annexin A11	6197	0.028204642236232758	0.5476703	No
**220016_at**	AHNAK	AHNAK nucleoprotein (desmoyokin)	6199	0.028164714574813843	0.5477646	No
**204460_s_at**	RAD1	RAD1 homolog (S. pombe)	6201	0.028081728145480156	0.5478584	No
**219431_at**	ARHGAP 10	Rho GTPase activating protein 10	6202	0.028043173253536224	0.548	No
**218615_s_at**	TMEM39 A	transmembrane protein 39A	6246	0.02746114507317543	0.5460779	No
**201982_s_at**	PAPPA	pregnancy-associated plasma protein A, pappalysin 1	6259	0.027298670262098312	0.54564065	No
**201332_s_at**	STAT6	signal transducer and activator of transcription 6, interleukin-4 induced	6290	0.026903394609689713	0.54433876	No
**204972_at**	OAS2	2'-5'-oligoadenylate synthetase 2, 69/71kDa	6293	0.026890836656093597	0.54437864	No
**202371_at**	TCEAL4	transcription elongation factor A (SII)-like 4	6327	0.026409761980175972	0.54293054	No
**210797_s_at**	OASL	2'-5'-oligoadenylate synthetase-like	6345	0.02625511959195137	0.54224837	No
**213284_at**	ZFP36L1	Zinc finger protein 36, C3H type-like 1	6362	0.026023870334029198	0.541613	No
**209491_s_at**	AMPD3	adenosine monophosphate deaminase (isoform E)	6370	0.025883030146360397	0.5414082	No
**210112_at**	HPS1	Hermansky-Pudlak syndrome 1	6375	0.025817910209298134	0.5413468	No
**211230_s_at**	PIK3CD	phosphoinositide-3-kinase, catalytic, delta polypeptide	6381	0.025721216574311256	0.54123706	No
**211198_s_at**	ICOSLG	inducible T-cell co-stimulator ligand	6387	0.02555791474878788	0.54112643	No
**211107_s_at**	AURKC	aurora kinase C	6405	0.025311525911092758	0.54043955	No
**206086_x_at**	HFE	hemochromatosis	6469	0.024601802229881287	0.5375446	No
**203180_at**	ALDH1A 3	aldehyde dehydrogenase 1 family, member A3	6487	0.024325553327798843	0.5368527	No
**204136_at**	COL7A1	collagen, type VII, alpha 1 (epidermolysis bullosa, dystrophic, dominant and recessive)	6497	0.024209417402744293	0.5365436	No
**205841_at**	JAK2	Janus kinase 2 (a protein tyrosine kinase)	6501	0.024189261719584465	0.5365219	No
**207977_s_at**	DPT	dermatopontin	6510	0.02409396879374981	0.5362602	No

**210025_s_at**	CARD10	caspase recruitment domain family, member 10	6523	0.023932822048664093	0.53580594	No
**203699_s_at**	DIO2	deiodinase, iodothyronine, type II	6529	0.023870334029197693	0.5356868	No
**219475_at**	OSGIN1	oxidative stress induced growth inhibitor 1	6534	0.02380399778485298	0.53561527	No
**210294_at**	TAPBP	TAP binding protein (tapasin)	6603	0.02290387451648712	0.53247213	No
**204415_at**	IFI6	interferon, alpha-inducible protein 6	6634	0.02250148169696331	0.5311481	No
**220300_at**	RGS3	regulator of G-protein signalling 3	6645	0.02238481305539608	0.5307818	No
**201272_at**	AKR1B1	aldo-keto reductase family 1, member B1 (aldose reductase)	6651	0.02234000340104103	0.53065497	No
**219923_at**	TRIM45	tripartite motif-containing 45	6655	0.022288916632533073	0.53062373	No
**220953_s_at**	MTMR12	myotubularin related protein 12	6657	0.022280476987361908	0.5306883	No
**221511_x_at**	CCPG1	cell cycle progression 1	6666	0.02212844230234623	0.5304166	No
**213415_at**	CLIC2	chloride intracellular channel 2	6675	0.022053280845284462	0.5301445	No
**202783_at**	NNT	nicotinamide nucleotide transhydrogenase	6691	0.02178727462887764	0.52953565	No
**218893_at**	ISOC2	isochorismatase domain containing 2	6736	0.021199507638812065	0.52753407	No
**218642_s_at**	CHCHD7	coiled-coil-helix-coiled-coil-helix domain containing 7	6739	0.021172625944018364	0.5275451	No
**214106_s_at**	GMDS	GDP-mannose 4,6-dehydratase	6758	0.02092747576534748	0.5267882	No
**202575_at**	CRABP2	cellular retinoic acid binding protein 2	6760	0.020915253087878227	0.5268458	No
**211197_s_at**	ICOSLG	inducible T-cell co-stimulator ligand	6772	0.020830916240811348	0.5264238	No
**204049_s_at**	PHACTR 2	phosphatase and actin regulator 2	6789	0.020543495193123817	0.52576077	No
**204682_at**	LTBP2	latent transforming growth factor beta binding protein 2	6812	0.020175978541374207	0.5248083	No
**219200_at**	FASTKD3	FAST kinase domains 3	6829	0.019978228956460953	0.5241424	No
**209942_x_at**	MAGEA3	melanoma antigen family A, 3	6856	0.019753672182559967	0.5229961	No
**218574_s_at**	LMCD1	LIM and cysteine-rich domains 1	6877	0.019517751410603523	0.5221362	No
**203309_s_at**	HPS1	Hermansky-Pudlak syndrome 1	6915	0.019152464345097542	0.5204597	No
**202434_s_at**	CYP1B1	cytochrome P450, family 1, subfamily B, polypeptide 1	6918	0.0191250778734684	0.5204604	No
**206614_at**	GDF5	growth differentiation factor 5 (cartilage-derived morphogenetic protein-1)	6924	0.019064389169216156	0.520317	No
**216904_at**	COL6A1	collagen, type VI, alpha 1	6926	0.019028501585125923	0.5203652	No
**209367_at**	STXBP2	syntaxin binding protein 2	6927	0.019004205241799355	0.5204611	No
**205665_at**	TSPAN9	tetraspanin 9	6949	0.018716424703598022	0.5195492	No
**210961_s_at**	ADRA1D	adrenergic, alpha-1D-, receptor	6973	0.018460018560290337	0.5185402	No
**35150_at**	CD40	CD40 molecule, TNF receptor superfamily member 5	7011	0.018079981207847595	0.5168583	No
**211204_at**	ME1	malic enzyme 1, NADP(+)-dependent, cytosolic	7018	0.01801040768623352	0.5166617	No

**210467_x_at**	MAGEA12	melanoma antigen family A, 12	7029	0.017759576439857483	0.5162721	No
**210675_s_at**	PTPRR	protein tyrosine phosphatase, receptor type, R	7044	0.01754307933151722	0.51568973	No
**209979_at**	ADARB1	adenosine deaminase, RNA-specific, B1 (RED1 homolog rat)	7074	0.01721641607582569	0.5143869	No
**204392_at**	CAMK1	calcium/calmodulin-dependent protein kinase I	7092	0.017014235258102417	0.5136581	No
**212173_at**	AK2	adenylate kinase 2	7187	0.016113996505737305	0.5092347	No
**220936_s_at**	H2AFJ	H2A histone family, member J	7192	0.01604708470404148	0.50912404	No
**208456_s_at**	RRAS2	related RAS viral (r-ras) oncogene homolog 2	7210	0.01584412157535553	0.50838935	No
**218850_s_at**	LIMD1	LIM domains containing 1	7226	0.015632428228855133	0.5077494	No
**211003_x_at**	TGM2	transglutaminase 2 (C polypeptide, protein-glutamine-gamma-glutamyltransferase)	7233	0.015548557974398136	0.50754035	No
**203586_s_at**	ARL4D	ADP-ribosylation factor-like 4D	7241	0.015457389876246452	0.5072829	No
**217312_s_at**	COL7A1	collagen, type VII, alpha 1 (epidermolysis bullosa, dystrophic, dominant and recessive)	7251	0.015356935560703278	0.50692916	No
**203672_x_at**	TPMT	thiopurine S-methyltransferase	7272	0.015195641666650772	0.50604737	No
**206636_at**	RASA2	RAS p21 protein activator 2	7287	0.014998085796833038	0.50545216	No
**219423_x_at**	TNFRSF25	tumor necrosis factor receptor superfamily, member 25	7289	0.014991980977356434	0.50547993	No
**211320_s_at**	PTPRU	protein tyrosine phosphatase, receptor type, U	7295	0.014945099130272865	0.5053158	No
**221790_s_at**	LDLRAP1	low density lipoprotein receptor adaptor protein 1	7305	0.014859642833471298	0.50495946	No
**206429_at**	F2RL1	coagulation factor II (thrombin) receptor-like 1	7313	0.014809953048825264	0.50469875	No
**205068_s_at**	ARHGAP26	Rho GTPase activating protein 26	7321	0.014723546802997589	0.5044376	No
**209477_at**	EMD	emerin (Emery-Dreifuss muscular dystrophy)	7322	0.01471291109919548	0.5045119	No
**208116_s_at**	MAN1A1	mannosidase, alpha, class 1A, member 1	7325	0.014699650928378105	0.50449026	No
**211671_s_at**	NR3C1	)	7329	0.014647078700363636	0.5044204	No
**219327_s_at**	GPRC5C	G protein-coupled receptor, family C, group 5, member C	7348	0.01448152121156454	0.50363094	No
**209008_x_at**	KRT8	keratin 8 /// keratin 8	7356	0.014438057318329811	0.5033683	No
**64883_at**	MOSPD2	motile sperm domain containing 2	7406	0.014068687334656715	0.5010912	No
**209175_at**	SEC23IP	SEC23 interacting protein	7432	0.013798338361084461	0.49996275	No
**201633_s_at**	CYB5B	cytochrome b5 type B (outer mitochondrial membrane)	7437	0.013763688504695892	0.49984053	No
**221348_at**	NPPC	natriuretic peptide precursor C	7480	0.013290779665112495	0.49789488	No
**203199_s_at**	MTRR	5-methyltetrahydrofolate-homocysteine methyltransferase reductase	7502	0.013121230527758598	0.49695474	No
**218364_at**	LRRFIP2	leucine rich repeat (in FLII) interacting protein 2	7525	0.012883316725492477	0.49596548	No

**210637_at**	TACR1	tachykinin receptor 1	7530	0.012822726741433144	0.4958385	No
**210960_at**	ADRA1D	adrenergic, alpha-1D-, receptor	7555	0.012620735913515091	0.49475208	No
**203718_at**	NT5E	5'-nucleotidase, ecto (CD73)	7570	0.012472474947571754	0.4941441	No
**214237_x_at**	PAWR	PRKC, apoptosis, WT1, regulator	7607	0.0121694877743721	0.49248034	No
**220603_s_at**	MCTP2	multiple C2 domains, transmembrane 2	7612	0.012125669978559017	0.49234986	No
**213764_s_at**	MFAP5	microfibrillar associated protein 5	7615	0.012103174813091755	0.4923151	No
**220333_at**	PAQR5	progestin and adipoQ receptor family member V	7627	0.011992109939455986	0.4918485	No
**218511_s_at**	PNPO	pyridoxine 5'-phosphate oxidase	7674	0.011519484221935272	0.4897022	No
**202312_s_at**	COL1A1	collagen, type I, alpha 1	7687	0.011405193246901035	0.4891847	No
**217055_x_at**	SLC1A2	Solute carrier family 1 (glial high affinity glutamate transporter), member 2	7704	0.011205950751900673	0.48847452	No
**222245_s_at**	FER1L4	fer-1-like 4 (C. elegans)	7726	0.0109874177724123	0.4875236	No
**201403_s_at**	MGST3	microsomal glutathione S-transferase 3	7760	0.010723244398832321	0.48599628	No
**204045_at**	TCEAL1	transcription elongation factor A (SII)-like 1	7766	0.01068273838609457	0.48581058	No
**208600_s_at**	GPR39	G protein-coupled receptor 39	7779	0.010554437525570393	0.4852888	No
**219026_s_at**	RASAL2	RAS protein activator like 2	7796	0.010384509339928627	0.48457447	No
**215775_at**	THBS1	Thrombospondin 1	7843	0.009911179542541504	0.48242006	No
**213256_at**	Mar-03	membrane-associated ring finger (C3HC4) 3	7848	0.009860201738774776	0.48227814	No
**211841_s_at**	TNFRSF25	tumor necrosis factor receptor superfamily, member 25	7850	0.00984994973987341	0.48227993	No
**219572_at**	CADPS2	Ca2+-dependent activator protein for secretion 2	7873	0.009632524102926254	0.48127425	No
**57082_at**	LDLRAP1	low density lipoprotein receptor adaptor protein 1	7886	0.00953468307852745	0.4807473	No
**209587_at**	PITX1	paired-like homeodomain transcription factor 1	7935	0.00899318978190422	0.47849244	No
**218099_at**	TEX2	testis expressed sequence 2	7976	0.00858827494084835	0.4766189	No
**205832_at**	CPA4	carboxypeptidase A4	7981	0.008539232425391674	0.4764703	No
**200842_s_at**	EPRS	glutamyl-prolyl-tRNA synthetase	7985	0.00848555751144886	0.47636935	No
**33646_g_at**	GM2A	GM2 ganglioside activator	8009	0.008323464542627335	0.47530916	No
**217255_at**	SQSTM1	sequestosome 1	8018	0.008284124545753002	0.4749676	No
**212939_at**	COL6A1	collagen, type VI, alpha 1	8045	0.008115643635392189	0.47376257	No
**217711_at**	TEK	TEK tyrosine kinase, endothelial (venous malformations, multiple cutaneous and mucosal)	8057	0.008035968989133835	0.473276	No
**208181_at**	HIST1H4H	histone 1, H4h	8060	0.008014942519366741	0.47322062	No
**59631_at**	TXNRD3	thioredoxin reductase 3	8064	0.0079941526055336	0.47311717	No
**221584_s_at**	KCNMA1	potassium large conductance calcium-activated channel, subfamily M, alpha member 1	8158	0.007087125908583403	0.46869618	No
**208215_x_at**	DRD4	dopamine receptor D4	8173	0.00696745328605175	0.46806043	No

**203788_s_at**	SEMA3C	sema domain, immunoglobulin domain (Ig), short basic domain, secreted, (semaphorin) 3C	8183	0.006880403961986303	0.46766385	No
**209108_at**	TSPAN6	tetraspanin 6	8200	0.006772067863494158	0.46693128	No
**212562_s_at**	CTSZ	Cathepsin Z	8246	0.006465440150350332	0.4648074	No
**201099_at**	USP9X	ubiquitin specific peptidase 9, X-linked	8259	0.006374880205839872	0.4642645	No
**216259_at**	BMPER	BMP binding endothelial regulator	8288	0.006130510475486517	0.46295363	No
**220968_s_at**	TSPAN9	tetraspanin 9 /// tetraspanin 9	8315	0.005913208704441786	0.46173748	No
**205401_at**	AGPS	alkylglycerone phosphate synthase	8331	0.005821512080729008	0.46104804	No
**201865_x_at**	NR3C1	nuclear receptor subfamily 3, group C, member 1 (glucocorticoid receptor)	8373	0.005325019359588623	0.45911008	No
**215501_s_at**	DUSP10	dual specificity phosphatase 10	8390	0.005155416205525398	0.45836934	No
**210275_s_at**	ZFAND5	zinc finger, AN1-type domain 5	8441	0.004818398505449295	0.45599753	No
**215891_s_at**	GM2A	GM2 ganglioside activator	8454	0.004670027177780867	0.45544603	No
**219716_at**	APOL6	apolipoprotein L, 6	8503	0.004278924781829119	0.45316735	No
**206908_s_at**	CLDN11	claudin 11 (oligodendrocyte transmembrane protein)	8573	0.003591598942875862	0.44987884	No
**202826_at**	SPINT1	serine peptidase inhibitor, Kunitz type 1	8603	0.0033755991607904434	0.44850612	No
**220610_s_at**	LRRFIP2	leucine rich repeat (in FLII) interacting protein 2	8606	0.0033679057378321886	0.44842726	No
**216866_s_at**	COL14A1	collagen, type XIV, alpha 1 (undulin)	8607	0.00336616113781929	0.44844428	No
**214337_at**	COPA	coatomer protein complex, subunit alpha	8618	0.003289359388872981	0.44798166	No
**203671_at**	TPMT	thiopurine S-methyltransferase	8667	0.0028940194752067327	0.44569597	No
**220254_at**	LRP12	low density lipoprotein-related protein 12	8690	0.0027293760795146227	0.44465545	No
**209114_at**	TSPAN1	tetraspanin 1	8706	0.0026198255363851786	0.44394985	No
**207375_s_at**	IL15RA	interleukin 15 receptor, alpha	8720	0.002499279798939824	0.44333947	No
**202515_at**	DLG1	discs, large homolog 1 (Drosophila)	8757	0.0022155195474624634	0.44162542	No
**219790_s_at**	NPR3	natriuretic peptide receptor C/guanylate cyclase C (atrionatriuretic peptide receptor C)	8793	0.0019509200938045979	0.43995798	No
**201430_s_at**	DPYSL3	dihydropyrimidinase-like 3	8798	0.0018896121764555573	0.43977582	No
**219326_s_at**	B3GNT2	UDP-GlcNAc:betaGal beta-1,3-N-acetylglucosaminyltransferase 2	8819	0.0017148341285064816	0.43882605	No
**209727_at**	GM2A	GM2 ganglioside activator	8843	0.0014507316518574953	0.43773115	No
**216392_s_at**	SEC23IP	SEC23 interacting protein	8845	0.0014237264404073358	0.4376904	No
**200841_s_at**	EPRS	glutamyl-prolyl-tRNA synthetase	8861	0.0012674357276409864	0.43697798	No
**206526_at**	RIBC2	RIB43A domain with coiled-coils 2	8865	0.0012332116020843387	0.43684042	No
**207834_at**	FBLN1	fibulin 1	8912	8,53E+11	0.4346403	No

**218182_s_at**	CLDN1	claudin 1	8913	8,52E+11	0.4346446	No
**203768_s_at**	STS	steroid sulfatase (microsomal), arylsulfatase C, isozyme S	8925	7,73E+11	0.43412134	No
**202323_s_at**	ACBD3	acyl-Coenzyme A binding domain containing 3	8975	3,61E+11	0.43177497	No
**220388_at**	FER1L4	fer-1-like 4 (C. elegans)	8977	3,48E+12	0.4317288	No
**209758_s_at**	MFAP5	microfibrillar associated protein 5	9037	-1,43E+12	0.4289021	No
**211124_s_at**	KITLG	KIT ligand	9153	-9,79E+11	0.42339593	No
**209632_at**	PPP2R3A	protein phosphatase 2 (formerly 2A), regulatory subunit B'', alpha	9181	-0.0011878479272127151	0.42210802	No
**221284_s_at**	SRC	v-src sarcoma (Schmidt-Ruppin A-2) viral oncogene homolog (avian)	9199	-0.0013073045993223786	0.42129993	No
**216190_x_at**	ITGB1	integrin, beta 1 (fibronectin receptor, beta polypeptide, antigen CD29 includes MDF2, MSK12)	9214	-0.001402935478836298	0.42063612	No
**213443_at**	TRADD	TNFRSF1A-associated via death domain	9270	-0.0018449326744303107	0.4180097	No
**207133_x_at**	ALPK1	alpha-kinase 1	9297	-0.0020472484175115824	0.41677403	No
**213814_s_at**	SNTB2	Syntrophin, beta 2 (dystrophin-associated protein A1, 59kDa, basic component 2)	9305	-0.0021021063439548016	0.4164492	No
**205373_at**	CTNNA2	catenin (cadherin-associated protein), alpha 2	9312	-0.0021400374826043844	0.41617244	No
**220002_at**	KIF26B	kinesin family member 26B	9324	-0.0022299790289252996	0.41565657	No
**214200_s_at**	COL6A1	Collagen, type VI, alpha 1	9356	-0.0024397780653089285	0.4141833	No
**219448_at**	TMEM70	transmembrane protein 70	9365	-0.0025123462546616793	0.41381258	No
**215618_at**	RSU1	Ras suppressor protein 1	9396	-0.0027374548371881247	0.4123887	No
**202966_at**	CAPN6	calpain 6	9403	-0.0027770560700446367	0.41211522	No
**219101_x_at**	ABHD8	abhydrolase domain containing 8	9407	-0.0028197504580020905	0.41198567	No
**200843_s_at**	EPRS	glutamyl-prolyl-tRNA synthetase	9432	-0.0029607603792101145	0.41085047	No
**210216_x_at**	RAD1	RAD1 homolog (S. pombe)	9508	-0.003483963431790471	0.40727386	No
**207528_s_at**	SLC7A11	solute carrier family 7, (cationic amino acid transporter, y+ system) member 11	9538	-0.003764189314097166	0.4059031	No
**203769_s_at**	STS	steroid sulfatase (microsomal), arylsulfatase C, isozyme S	9572	-0.0040433467365801334	0.4043421	No
**215988_s_at**	DLG1	Discs, large homolog 1 (Drosophila)	9617	-0.004293097648769617	0.40225515	No
**207485_x_at**	BTN3A1	butyrophilin, subfamily 3, member A1	9635	-0.004461195785552263	0.401463	No
**202999_s_at**	LOXL2	lysyl oxidase-like 2	9655	-0.004631041083484888	0.40057585	No
**205314_x_at**	SNTB2	syntrophin, beta 2 (dystrophin-associated protein A1, 59kDa, basic component 2)	9661	-0.0047088065184652805	0.40036	No

**207763_at**	S100A5	S100 calcium binding protein A5	9700	-0.005048978142440319	0.39856443	No
**211855_s_at**	SLC25A14	solute carrier family 25 (mitochondrial carrier, brain), member 14	9705	-0.005105932708829641	0.39839852	No
**204626_s_at**	ITGB3	integrin, beta 3 (platelet glycoprotein IIIa, antigen CD61)	9723	-0.0052210865542292595	0.3976102	No
**207943_x_at**	PLAGL1	pleiomorphic adenoma gene-like 1	9742	-0.005382364150136709	0.39677474	No
**222292_at**	CD40	CD40 molecule, TNF receptor superfamily member 5	9759	-0.005472832825034857	0.3960356	No
**207029_at**	KITLG	KIT ligand	9762	-0.005489609204232693	0.39596748	No
**214702_at**	FN1	fibronectin 1	9804	-0.0057636587880551815	0.39403173	No
**214445_at**	ELL2	elongation factor, RNA polymerase II, 2	9822	-0.005860977806150913	0.39324665	No
**212703_at**	TLN2	talin 2	9827	-0.005889850668609142	0.3930847	No
**206208_at**	CA4	carbonic anhydrase IV	9860	-0.006110446527600288	0.391582	No
**219287_at**	KCNMB4	potassium large conductance calcium-activated channel, subfamily M, beta member 4	9888	-0.006237473338842392	0.3903196	No
**220796_x_at**	SLC35E1	solute carrier family 35, member E1	9913	-0.006409702822566032	0.3892018	No
**207415_at**	PLA2R1	phospholipase A2 receptor 1, 180kDa	9939	-0.006600766908377409	0.38803706	No
**222168_at**	ALDH1A3	Aldehyde dehydrogenase 1 family, member A3	9974	-0.006837963592261076	0.3864422	No
**211056_s_at**	SRD5A1	1 (3-oxo-5 alpha-steroid delta 4-dehydrogenase alpha 1)	10018	-0.0072385892271995544	0.38441807	No
**207017_at**	RAB27B	RAB27B, member RAS oncogene family	10072	-0.0075392452999949455	0.38191625	No
**217438_at**	KCNIP4	Kv channel interacting protein 4	10110	-0.007852989248931408	0.38018274	No
**200930_s_at**	VCL	vinculin	10115	-0.007873965427279472	0.3800308	No
**217194_at**	RASAL2	RAS protein activator like 2	10153	-0.008126048371195793	0.3782987	No
**201311_s_at**	SH3BGRL	SH3 domain binding glutamic acid-rich protein like	10172	-0.008240938186645508	0.37747768	No
**215082_at**	ELOVL5	ELOVL family member 5, elongation of long chain fatty acids (FEN1/Elo2, SUR4/Elo3-like, yeast)	10188	-0.00835458468645811	0.376801	No
**221285_at**	ST8SIA2	ST8 alpha-N-acetyl-neuraminide alpha-2,8-sialyltransferase 2	10200	-0.008497149683535099	0.37631676	No
**211676_s_at**	IFNGR1	interferon gamma receptor 1 /// interferon gamma receptor 1	10236	-0.008680884726345539	0.3746833	No
**210945_at**	COL4A6	collagen, type IV, alpha 6	10327	-0.009355273097753525	0.37041748	No
**212651_at**	RHOBTB1	Rho-related BTB domain containing 1	10349	-0.00951351411640644	0.36945912	No
**211282_x_at**	TNFRSF25	tumor necrosis factor receptor superfamily, member 25	10353	-0.009531890973448753	0.3693635	No
**215879_at**	ITGB1	integrin, beta 1 (fibronectin receptor, beta polypeptide, antigen CD29 includes MDF2, MSK12)	10378	-0.009642497636377811	0.36826202	No
**203311_s_at**	ARF6	ADP-ribosylation factor 6	10407	-0.009790944866836071	0.36696962	No

**216261_at**	ITGB3	integrin, beta 3 (platelet glycoprotein IIIa, antigen CD61)	10434	-0.009926137514412403	0.36577374	No
**201610_at**	ICMT	isoprenylcysteine carboxyl methyltransferase	10435	-0.009927862323820591	0.36582384	No
**220550_at**	FBXO4	F-box protein 4	10445	-0.010023106820881367	0.36544314	No
**221281_at**	SRC	v-src sarcoma (Schmidt-Ruppin A-2) viral oncogene homolog (avian)	10462	-0.010096610523760319	0.36472735	No
**214090_at**	PAWR	PRKC, apoptosis, WT1, regulator	10522	-0.010483972728252411	0.3619528	No
**207071_s_at**	ACO1	aconitase 1, soluble	10523	-0.010485807433724403	0.36200577	No
**220901_at**	GPR157	G protein-coupled receptor 157	10590	-0.010970263741910458	0.35889825	No
**213816_s_at**	MET	met proto-oncogene (hepatocyte growth factor receptor)	10655	-0.011435887776315212	0.35588893	No
**205069_s_at**	ARHGAP 26	Rho GTPase activating protein 26	10658	-0.011458942666649818	0.35585093	No
**211323_s_at**	ITPR1	inositol 1,4,5-triphosphate receptor, type 1	10724	-0.012039968743920326	0.35279673	No
**207999_s_at**	ADARB1	adenosine deaminase, RNA-specific, B1 (RED1 homolog rat)	10805	-0.01265688706189394	0.34902683	No
**210133_at**	CCL11	chemokine (C-C motif) ligand 11	10807	-0.012662727385759354	0.3490428	No
**207510_at**	BDKRB1	bradykinin receptor B1	10836	-0.012815282680094242	0.34776568	No
**207301_at**	EFNA5	ephrin-A5	10867	-0.01303959172219038	0.34639382	No
**216627_s_at**	B4GALT1	UDP-Gal:betaGlcNAc beta 1,4- galactosyltransferase, polypeptide 1	10875	-0.013069703243672848	0.34612432	No
**217037_at**	SLC1A2	Solute carrier family 1 (glial high affinity glutamate transporter), member 2	10876	-0.01306970790028572	0.3461903	No
**215649_s_at**	MVK	mevalonate kinase (mevalonic aciduria)	10881	-0.013116037473082542	0.34606484	No
**201596_x_at**	KRT18	keratin 18	10892	-0.013219942338764668	0.34565234	No
**202784_s_at**	NNT	nicotinamide nucleotide transhydrogenase	10903	-0.013297752477228642	0.34524024	No
**218651_s_at**	LARP6	La ribonucleoprotein domain family, member 6	10925	-0.01344485767185688	0.34430173	No
**214153_at**	ELOVL5	ELOVL family member 5, elongation of long chain fatty acids (FEN1/Elo2, SUR4/Elo3-like, yeast)	11024	-0.014056544750928879	0.33967626	No
**219483_s_at**	PORCN	porcupine homolog (Drosophila)	11048	-0.014185533858835697	0.33864567	No
**206230_at**	LHX1	LIM homeobox 1	11091	-0.01447549182921648	0.33670598	No
**212642_s_at**	HIVEP2	human immunodeficiency virus type I enhancer binding protein 2	11112	-0.014589029364287853	0.33582118	No
**216062_at**	CD44	CD44 molecule (Indian blood group)	11117	-0.014604855328798294	0.3357032	No
**204618_s_at**	GABPB2	GA binding protein transcription factor, beta subunit 2	11128	-0.014653808437287807	0.33529794	No
**209946_at**	VEGFC	vascular endothelial growth factor C	11145	-0.014780386351048946	0.3346058	No

**203904_x_at**	CD82	CD82 molecule	11158	-0.01485761720687151	0.33410573	No
**213518_at**	PRKCI	protein kinase C, iota	11163	-0.014880345202982426	0.33398914	No
**216775_at**	USP53	ubiquitin specific peptidase 53	11181	-0.014990423806011677	0.33325014	No
**221029_s_at**	WNT5B	wingless-type MMTV integration site family, member 5B /// wingless-type MMTV integration site family, member 5B	11217	-0.015210714191198349	0.33164963	No
**203587_at**	ARL4D	ADP-ribosylation factor-like 4D	11246	-0.015346121042966843	0.33038527	No
**217248_s_at**	SLC7A8	solute carrier family 7 (cationic amino acid transporter, y+ system), member 8	11259	-0.015425502322614193	0.32988805	No
**219222_at**	RBKS	ribokinase	11442	-0.01659570075571537	0.32124993	No
**204625_s_at**	ITGB3	integrin, beta 3 (platelet glycoprotein IIIa, antigen CD61)	11453	-0.016655532643198967	0.32085478	No
**215641_at**	SEC24D	SEC24 related gene family, member D (S. cerevisiae)	11461	-0.01677914336323738	0.32060403	No
**220253_s_at**	LRP12	low density lipoprotein-related protein 12	11478	-0.0168768297880888	0.31992245	No
**207852_at**	CXCL5	chemokine (C-X-C motif) ligand 5	11504	-0.017038464546203613	0.31881037	No
**211705_s_at**	SORBS1	sorbin and SH3 domain containing 1 /// sorbin and SH3 domain containing 1	11556	-0.01735314354300499	0.31645393	No
**204344_s_at**	SEC23A	Sec23 homolog A (S. cerevisiae)	11596	-0.017658734694123268	0.31467408	No
**211120_x_at**	ESR2	estrogen receptor 2 (ER beta)	11614	-0.01777360402047634	0.31394914	No
**216974_at**	KITLG	KIT ligand	11634	-0.017827462404966354	0.3131286	No
**209369_at**	ANXA3	annexin A3	11691	-0.01825803890824318	0.3105371	No
**201866_s_at**	NR3C1	nuclear receptor subfamily 3, group C, member 1 (glucocorticoid receptor)	11696	-0.018290000036358833	0.3104377	No
**205490_x_at**	GJB3	gap junction protein, beta 3, 31kDa (connexin 31)	11717	-0.018438076600432396	0.30957234	No
**210194_at**	PLA2R1	phospholipase A2 receptor 1, 180kDa	11731	-0.01855020970106125	0.309043	No
**209677_at**	PRKCI	protein kinase C, iota	11735	-0.01857904903590679	0.308993	No
**210780_at**	ESR2	estrogen receptor 2 (ER beta)	11746	-0.018651222810149193	0.30860794	No
**220306_at**	FAM46C	family with sequence similarity 46, member C	11757	-0.018701130524277687	0.3082231	No
**217675_at**	ZBTB7C	zinc finger and BTB domain containing 7C	11769	-0.018760237842798233	0.30779067	No
**208384_s_at**	MID2	midline 2	11856	-0.019357604905962944	0.30376703	No
**202965_s_at**	CAPN6	calpain 6	11885	-0.019476601853966713	0.30252352	No
**204439_at**	IFI44L	interferon-induced protein 44-like	11888	-0.019485188648104668	0.30252603	No
**215957_at**	UBE2D1	ubiquitin-conjugating enzyme E2D 1 (UBC4/5 homolog, yeast)	11893	-0.019535847008228302	0.30243295	No
**219926_at**	POPDC3	popeye domain containing 3	11913	-0.019628752022981644	0.3016215	No
**217387_at**	CAPN6	calpain 6	11928	-0.019719969481229782	0.30105013	No

**215415_s_at**	LYST	lysosomal trafficking regulator	11948	-0.019888361915946007	0.30024	No
**214590_s_at**	UBE2D1	ubiquitin-conjugating enzyme E2D 1 (UBC4/5 homolog, yeast)	11963	-0.019965890794992447	0.29966986	No
**208174_x_at**	ZRSR2	zinc finger (CCCH type), RNA-binding motif and serine/arginine rich 2	11975	-0.020050669088959694	0.29924393	No
**214981_at**	POSTN	Periostin, osteoblast specific factor	12007	-0.02034749649465084	0.29786104	No
**205472_s_at**	DACH1	dachshund homolog 1 (Drosophila)	12014	-0.02040233463048935	0.2976765	No
**213258_at**	TFPI	tissue factor pathway inhibitor (lipoprotein-associated coagulation inhibitor)	12019	-0.02044552005827427	0.29758802	No
**211118_x_at**	ESR2	estrogen receptor 2 (ER beta)	12020	-0.020445777103304863	0.29769123	No
**210542_s_at**	SLCO3A1	solute carrier organic anion transporter family, member 3A1	12041	-0.02058481238782406	0.29683667	No
**206127_at**	ELK3	ELK3, ETS-domain protein (SRF accessory protein 2)	12050	-0.020647713914513588	0.29655752	No
**201349_at**	SLC9A3R 1	solute carrier family 9 (sodium/hydrogen exchanger), member 3 regulator 1	12077	-0.02077057957649231	0.29541638	No
**214860_at**	SLC9A7	solute carrier family 9 (sodium/hydrogen exchanger), member 7	12098	-0.02089827135205269	0.2945634	No
**208373_s_at**	P2RY6	pyrimidinergic receptor P2Y, G-protein coupled, 6	12102	-0.020914848893880844	0.29452524	No
**204048_s_at**	PHACTR 2	phosphatase and actin regulator 2	12127	-0.021068155765533447	0.29348144	No
**220523_at**	EFHC2	EF-hand domain (C-terminal) containing 2	12153	-0.021236641332507133	0.29239056	No
**214031_s_at**	KRT7	Keratin 7	12185	-0.021473774686455727	0.29101336	No
**209897_s_at**	SLIT2	slit homolog 2 (Drosophila)	12195	-0.021571513265371323	0.29069096	No
**202685_s_at**	AXL	AXL receptor tyrosine kinase	12209	-0.021637901663780212	0.2901772	No
**211473_s_at**	COL4A6	collagen, type IV, alpha 6	12213	-0.021675795316696167	0.29014283	No
**204056_s_at**	MVK	mevalonate kinase (mevalonic aciduria)	12226	-0.021766796708106995	0.28967765	No
**205243_at**	SLC13A3	solute carrier family 13 (sodium-dependent dicarboxylate transporter), member 3	12281	-0.022197186946868896	0.28720188	No
**45714_at**	HCFC1R1	host cell factor C1 regulator 1 (XPO1 dependent)	12286	-0.022229289636015892	0.2871224	No
**208378_x_at**	FGF5	fibroblast growth factor 5	12293	-0.022293366491794586	0.2869474	No
**213876_x_at**	ZRSR2	zinc finger (CCCH type), RNA-binding motif and serine/arginine rich 2	12308	-0.02240813709795475	0.2863896	No
**206399_x_at**	CACNA1 A	calcium channel, voltage-dependent, P/Q type, alpha 1A subunit	12315	-0.022448617964982986	0.28621536	No
**211986_at**	AHNAK	AHNAK nucleoprotein (desmoyokin)	12368	-0.02284310571849346	0.2838387	No
**208428_at**	TAP2	transporter 2, ATP-binding cassette, sub-family B (MDR/TAP)	12405	-0.023122865706682205	0.2822302	No

**210617_at**	PHEX	phosphate regulating endopeptidase homolog, X-linked (hypophosphatemia, vitamin D resistant rickets)	12420	-0.02317897044122219	0.2816763	No
**217911_s_at**	BAG3	BCL2-associated athanogene 3	12436	-0.0232505202293396	0.28107482	No
**216865_at**	COL14A1	collagen, type XIV, alpha 1 (undulin)	12439	-0.023280968889594078	0.2810965	No
**208096_s_at**	COL21A1	collagen, type XXI, alpha 1 /// collagen, type XXI, alpha 1	12444	-0.023303618654608727	0.28102243	No
**206539_s_at**	CYP4F12	cytochrome P450, family 4, subfamily F, polypeptide 12	12454	-0.023419121280312538	0.28070936	No
**210593_at**	SAT1	spermidine/spermine N1-acetyltransferase 1	12554	-0.024153370410203934	0.27608696	No
**201431_s_at**	DPYSL3	dihydropyrimidinase-like 3	12555	-0.024155789986252785	0.27620888	No
**217389_s_at**	ATF5	activating transcription factor 5	12573	-0.02427343837916851	0.27551672	No
**211497_x_at**	NKX3-1	NK3 transcription factor related, locus 1 (Drosophila)	12648	-0.02476596087217331	0.27209547	No
**210557_x_at**	CSF1	colony stimulating factor 1 (macrophage)	12685	-0.02497377246618271	0.2704963	No
**201442_s_at**	ATP6AP2	ATPase, H+ transporting, lysosomal accessory protein 2	12766	-0.025535834953188896	0.2667914	No
**205017_s_at**	MBNL2	muscleblind-like 2 (Drosophila)	12767	-0.0255371555685997	0.26692033	No
**215182_x_at**	MYH9	Myosin, heavy polypeptide 9, non-muscle	12793	-0.02568201720714569	0.26585189	No
**207191_s_at**	ISLR	immunoglobulin superfamily containing leucine-rich repeat	12835	-0.025986533612012863	0.26401824	No
**206112_at**	ANKRD7	ankyrin repeat domain 7	12895	-0.02636416256427765	0.2613239	No
**216321_s_at**	NR3C1	nuclear receptor subfamily 3, group C, member 1 (glucocorticoid receptor)	12906	-0.026409391313791275	0.26097798	No
**210310_s_at**	FGF5	fibroblast growth factor 5	12933	-0.02657688595354557	0.25986615	No
**217252_at**	SQSTM1	sequestosome 1	12974	-0.02683042176067829	0.25808468	No
**211573_x_at**	TGM2	transglutaminase 2 (C polypeptide, protein-glutamine-gamma-glutamyltransferase)	13064	-0.0274642501026392	0.25395823	No
**206084_at**	PTPRR	protein tyrosine phosphatase, receptor type, R	13111	-0.027844280004501343	0.25189433	No
**213621_s_at**	GUK1	Guanylate kinase 1	13117	-0.027887685224413872	0.2517955	No
**220803_at**	STAMBPL1	STAM binding protein-like 1	13128	-0.0279338750988245	0.25145727	No
**201017_at**	EIF1AX	eukaryotic translation initiation factor 1A, X-linked	13151	-0.028066564351320267	0.25054464	No
**206503_x_at**	PML	promyelocytic leukemia	13162	-0.02816769853234291	0.2502076	No
**207652_s_at**	CMKLR1	chemokine-like receptor 1	13242	-0.028806013986468315	0.24656714	No
**207326_at**	BTC	betacellulin	13247	-0.028844207525253296	0.24652106	No
**209505_at**	NR2F1	Nuclear receptor subfamily 2, group F, member 1	13281	-0.02905658259987831	0.24508628	No
**207368_at**	HTR1D	5-hydroxytryptamine (serotonin) receptor 1D	13314	-0.029269838705658913	0.2437005	No
**212530_at**	NEK7	NIMA (never in mitosis gene a)-related kinase 7	13382	-0.029685785993933678	0.24063954	No
**214166_at**	SDHB	succinate dehydrogenase complex, subunit B, iron sulfur (Ip)	13383	-0.02968752011656761	0.2407894	No

**214082_at**	CA5B	carbonic anhydrase VB, mitochondrial	13389	-0.029742568731307983	0.24069992	No
**210136_at**	MBP	myelin basic protein	13415	-0.029936503618955612	0.23965298	No
**218537_at**	HCFC1R1	host cell factor C1 regulator 1 (XPO1 dependent)	13426	-0.0300229974091053	0.2393253	No
**210864_x_at**	HFE	hemochromatosis	13476	-0.030363481491804123	0.23713036	No
**204621_s_at**	NR4A2	nuclear receptor subfamily 4, group A, member 2	13480	-0.03037075698375702	0.23713991	No
**201093_x_at**	SDHA	succinate dehydrogenase complex, subunit A, flavoprotein (Fp)	13520	-0.030688166618347168	0.23542583	No
**209678_s_at**	PRKCI	protein kinase C, iota	13540	-0.030810363590717316	0.23467083	No
**208015_at**	SMAD1	SMAD, mothers against DPP homolog 1 (Drosophila)	13544	-0.030825801193714142	0.23468266	No
**215884_s_at**	UBQLN2	ubiquilin 2	13547	-0.0308542363345623	0.23474257	No
**214917_at**	PRKAA1	protein kinase, AMP-activated, alpha 1 catalytic subunit	13573	-0.031006217002868652	0.23370102	No
**216056_at**	CD44	CD44 molecule (Indian blood group)	13599	-0.031193288043141365	0.23266041	No
**206072_at**	UCN	urocortin	13611	-0.03128107637166977	0.23229116	No
**221283_at**	RUNX2	runt-related transcription factor 2	13654	-0.031551145017147064	0.23043768	No
**211498_s_at**	NKX3-1	NK3 transcription factor related, locus 1 (Drosophila)	13705	-0.0319085568189621	0.22820263	No
**215890_at**	GM2A	GM2 ganglioside activator	13724	-0.032052990049123764	0.22750182	No
**204047_s_at**	PHACTR 2	phosphatase and actin regulator 2	13742	-0.03212316334247589	0.22684929	No
**217201_at**	RASAL2	RAS protein activator like 2	13758	-0.03226688504219055	0.22629333	No
**206569_at**	IL24	interleukin 24	13774	-0.032368823885917664	0.22573788	No
**212960_at**	TBC1D9	TBC1 domain family, member 9	13803	-0.03253284841775894	0.22456028	No
**206673_at**	GPR176	G protein-coupled receptor 176	13833	-0.0326877161860466	0.22333552	No
**216994_s_at**	RUNX2	runt-related transcription factor 2	13867	-0.03297000005841255	0.2219205	No
**211764_s_at**	UBE2D1	ubiquitin-conjugating enzyme E2D 1 (UBC4/5 homolog, yeast) /// ubiquitin-conjugating enzyme E2D 1 (UBC4/5 homolog, yeast)	13878	-0.033034101128578186	0.22160803	No
**221421_s_at**	ADAMTS 12	ADAM metallopeptidase with thrombospondin type 1 motif, 12 /// ADAM metallopeptidase with thrombospondin type 1 motif, 12	13979	-0.03373675420880318	0.21698608	No
**206173_x_at**	GABPB2	GA binding protein transcription factor, beta subunit 2	13988	-0.03382439538836479	0.21677344	No
**213071_at**	DPT	dermatopontin	13998	-0.03390336036682129	0.21651326	No
**222061_at**	CD58	CD58 molecule	14002	-0.033925969153642654	0.21654075	No
**200092_s_at**	RPL37	ribosomal protein L37 /// ribosomal protein L37	14005	-0.033947303891181946	0.21661627	No
**206795_at**	F2RL2	coagulation factor II (thrombin) receptor-like 2	14006	-0.03394754230976105	0.21678764	No

**215240_at**	ITGB3	integrin, beta 3 (platelet glycoprotein IIIa, antigen CD61)	14017	-0.03402048349380493	0.21648014	No
**213450_s_at**	ICOSLG	inducible T-cell co-stimulator ligand	14029	-0.03407031670212746	0.21612497	No
**204896_s_at**	PTGER4	prostaglandin E receptor 4 (subtype EP4)	14067	-0.03431122004985809	0.21452503	No
**217597_x_at**	RAB40B	RAB40B, member RAS oncogene family	14144	-0.03496246039867401	0.2110594	No
**216248_s_at**	NR4A2	nuclear receptor subfamily 4, group A, member 2	14151	-0.034994058310985565	0.21094851	No
**211117_x_at**	ESR2	estrogen receptor 2 (ER beta)	14184	-0.03526211157441139	0.20959298	No
**217176_s_at**	ZFX	zinc finger protein, X-linked	14232	-0.03560589998960495	0.20752037	No
**221906_at**	TXNRD3	thioredoxin reductase 3	14253	-0.035748351365327835	0.20674236	No
**36907_at**	MVK	mevalonate kinase (mevalonic aciduria)	14293	-0.036120302975177765	0.20505571	No
**205601_s_at**	HOXB5	homeobox B5	14304	-0.03620303422212601	0.20475924	No
**210462_at**	BLZF1	basic leucine zipper nuclear factor 1 (JEM-1)	14307	-0.03621210530400276	0.20484617	No
**200613_at**	AP2M1	adaptor-related protein complex 2, mu 1 subunit	14527	-0.037797462195158005	0.19454193	No
**203865_s_at**	ADARB1	adenosine deaminase, RNA-specific, B1 (RED1 homolog rat)	14533	-0.03784146159887314	0.19449334	No
**207929_at**	GRPR	gastrin-releasing peptide receptor	14535	-0.03788842260837555	0.19463667	No
**208048_at**	TACR1	tachykinin receptor 1	14587	-0.038296133279800415	0.19238594	No
**210435_at**	NBR2	neighbor of BRCA1 gene 2	14593	-0.03833802044391632	0.19233985	No
**214378_at**	TFPI	tissue factor pathway inhibitor (lipoprotein-associated coagulation inhibitor)	14615	-0.0385291688144207	0.19152796	No
**215998_at**	SDK1	Sidekick homolog 1 (chicken)	14667	-0.03895578905940056	0.18928055	No
**221667_s_at**	HSPB8	heat shock 22kDa protein 8	14698	-0.03917646408081055	0.18804063	No
**201366_at**	ANXA7	annexin A7	14752	-0.03950696811079979	0.18570016	No
**206525_at**	GABRR1	gamma-aminobutyric acid (GABA) receptor, rho 1	14769	-0.039679691195487976	0.1851337	No
**214207_s_at**	CARD10	Caspase recruitment domain family, member 10	14773	-0.03970643877983093	0.18519036	No
**212531_at**	LCN2	lipocalin 2 (oncogene 24p3)	14847	-0.04018261283636093	0.18189485	No
**211119_at**	ESR2	estrogen receptor 2 (ER beta)	14895	-0.04050453379750252	0.17984696	No
**211579_at**	ITGB3	integrin, beta 3 (platelet glycoprotein IIIa, antigen CD61)	14927	-0.04074396565556526	0.17856702	No
**222353_at**	LIMD1	LIM domains containing 1	14939	-0.04085303097963333	0.1782461	No
**214819_at**	IQSEC2	IQ motif and Sec7 domain 2	14941	-0.040885813534259796	0.17840455	No
**207920_x_at**	ZFX	zinc finger protein, X-linked	14973	-0.04114886373281479	0.17712668	No
**214059_at**	IFI44	Interferon-induced protein 44	14998	-0.041317231953144073	0.17618509	No
**201648_at**	JAK1	Janus kinase 1 (a protein tyrosine kinase)	15019	-0.041444066911935806	0.17543584	No
**220062_s_at**	MAGEC2	melanoma antigen family C, 2	15026	-0.0414867028594017	0.17535773	No

**215362_at**	ST8SIA2	ST8 alpha-N-acetyl-neuraminide alpha-2,8-sialyltransferase 2	15030	-0.04150845855474472	0.17542349	No
**31799_at**	COPB2	Coatomer protein complex, subunit beta 2 (beta prime)	15046	-0.041610460728406906	0.17491469	No
**210026_s_at**	CARD10	caspase recruitment domain family, member 10	15050	-0.041650108993053436	0.17498118	No
**204537_s_at**	GABRE	gamma-aminobutyric acid (GABA) A receptor, epsilon	15234	-0.043260253965854645	0.16642971	No
**208049_s_at**	TACR1	tachykinin receptor 1	15249	-0.04343121126294136	0.16597803	No
**213167_s_at**	MRPS6	Mitochondrial ribosomal protein S6	15251	-0.043442096561193466	0.1661494	No
**216604_s_at**	SLC7A8	solute carrier family 7 (cationic amino acid transporter, y+ system), member 8	15302	-0.04384483024477959	0.1639746	No
**211013_x_at**	PML	promyelocytic leukemia	15305	-0.043867915868759155	0.16410019	No
**204747_at**	IFIT3	interferon-induced protein with tetratricopeptide repeats 3	15329	-0.044046927243471146	0.1632203	No
**214336_s_at**	COPA	coatomer protein complex, subunit alpha	15353	-0.044219717383384705	0.1623413	No
**213023_at**	UTRN	utrophin (homologous to dystrophin)	15357	-0.04427242651581764	0.16242102	No
**211414_at**	GLS	glutaminase	15368	-0.04442644864320755	0.16216604	No
**207366_at**	KCNS1	potassium voltage-gated channel, delayed-rectifier, subfamily S, member 1	15422	-0.04493068903684616	0.15985295	No
**201882_x_at**	B4GALT1	UDP-Gal:betaGlcNAc beta 1,4- galactosyltransferase, polypeptide 1	15435	-0.04502340778708458	0.15950516	No
**208200_at**	IL1A	interleukin 1, alpha	15440	-0.04504206031560898	0.15954083	No
**215278_at**	NNT	Nicotinamide nucleotide transhydrogenase	15479	-0.04533872380852699	0.15794864	No
**203770_s_at**	STS	steroid sulfatase (microsomal), arylsulfatase C, isozyme S	15501	-0.0455460399389267	0.15717217	No
**214018_at**	GRIP1	glutamate receptor interacting protein 1	15553	-0.045971695333719254	0.15496019	No
**220580_at**	BICC1	bicaudal C homolog 1 (Drosophila)	15575	-0.046173132956027985	0.15418687	No
**207129_at**	CA5B	carbonic anhydrase VB, mitochondrial	15586	-0.046256329864263535	0.15394115	No
**209506_s_at**	NR2F1	nuclear receptor subfamily 2, group F, member 1	15620	-0.04648178070783615	0.15259434	No
**207018_s_at**	RAB27B	RAB27B, member RAS oncogene family	15634	-0.046617500483989716	0.15220666	No
**211215_x_at**	DIO2	deiodinase, iodothyronine, type II	15690	-0.04718925803899765	0.14980912	No
**209633_at**	PPP2R3A	protein phosphatase 2 (formerly 2A), regulatory subunit B", alpha	15726	-0.0474235899746418	0.14837122	No
**211354_s_at**	LEPR	leptin receptor	15754	-0.047668859362602234	0.14731795	No
**216051_x_at**	KIAA1217	KIAA1217	15796	-0.048049185425043106	0.14559565	No
**210615_at**	NRP1	neuropilin 1	15811	-0.048196941614151	0.14516804	No
**203902_at**	HEPH	hephaestin	15824	-0.0483085922896862	0.14483681	No

**203640_at**	MBNL2	muscleblind-like 2 (Drosophila)	15870	-0.048664484173059464	0.14292595	No
**221282_x_at**	RUNX2	runt-related transcription factor 2	15884	-0.0488256961107254	0.14254943	No
**220738_s_at**	RPS6KA6	ribosomal protein S6 kinase, 90kDa, polypeptide 6	15885	-0.04883306846022606	0.14279592	No
**220108_at**	GNA14	guanine nucleotide binding protein (G protein), alpha 14	15891	-0.04887447878718376	0.14280303	No
**207938_at**	PI15	peptidase inhibitor 15	15921	-0.04920901358127594	0.14166167	No
**219193_at**	WDR70	WD repeat domain 70	15969	-0.049625739455223083	0.1396598	No
**207099_s_at**	CHM	choroideremia (Rab escort protein 1)	15976	-0.0497024804353714	0.13962317	No
**216643_at**	KCNIP4	Kv channel interacting protein 4	16008	-0.050000958144664764	0.13838996	No
**208006_at**	FOXI1	forkhead box I1	16013	-0.05002749338746071	0.1384508	No
**216042_at**	TNFRSF2 5	tumor necrosis factor receptor superfamily, member 25	16018	-0.05004793405532837	0.13851175	No
**214399_s_at**	KRT8	Keratin 8	16071	-0.05059988051652908	0.1362752	No
**209071_s_at**	RGS5	regulator of G-protein signalling 5	16093	-0.05076620727777481	0.13552508	No
**205724_at**	PKP1	plakophilin 1 (ectodermal dysplasia/skin fragility syndrome)	16136	-0.051143400371074677	0.1337705	No
**211877_s_at**	PCDHGA 11	protocadherin gamma subfamily A, 11	16162	-0.05134011432528496	0.13283159	No
**205600_x_at**	HOXB5	homeobox B5	16191	-0.05161695182323456	0.13175032	No
**206970_at**	CNTN2	contactin 2 (axonal)	16198	-0.05171365290880203	0.13172382	No
**211839_s_at**	CSF1	colony stimulating factor 1 (macrophage)	16215	-0.05188931152224541	0.13121898	No
**216837_at**	EPHA5	EPH receptor A5	16235	-0.052081700414419174	0.13057137	No
**205846_at**	PTPRB	protein tyrosine phosphatase, receptor type, B	16258	-0.05229029804468155	0.12978102	No
**202074_s_at**	OPTN	optineurin	16345	-0.05314652994275093	0.12592795	No
**202105_at**	IGBP1	immunoglobulin (CD79A) binding protein 1	16351	-0.05320111662149429	0.1259569	No
**221932_s_at**	GLRX5	glutaredoxin 5 homolog (S. cerevisiae)	16445	-0.05402882397174835	0.121772826	No
**215787_at**	ACTA2	Actin, alpha 2, smooth muscle, aorta	16465	-0.054206643253564835	0.12113592	No
**221854_at**	PKP1	plakophilin 1 (ectodermal dysplasia/skin fragility syndrome)	16529	-0.05470988154411316	0.11839297	No
**214350_at**	SNTB2	Syntrophin, beta 2 (dystrophin-associated protein A1, 59kDa, basic component 2)	16575	-0.05523407459259033	0.116515264	No
**215878_at**	ITGB1	integrin, beta 1 (fibronectin receptor, beta polypeptide, antigen CD29 includes MDF2, MSK12)	16595	-0.05548442527651787	0.11588481	No
**210819_x_at**	DIO2	deiodinase, iodothyronine, type II	16598	-0.055511582642793655	0.11606918	No
**209860_s_at**	ANXA7	annexin A7	16635	-0.055903851985931396	0.11462616	No
**215459_at**	TAX1BP3	Tax1 (human T-cell leukemia virus type I) binding protein 3	16660	-0.056131668388843536	0.11375937	No

**221030_s_at**	ARHGAP 24	Rho GTPase activating protein 24 /// Rho GTPase activating protein 24	16685	-0.056394293904304504	0.112893894	No
**221013_s_at**	APOL2	apolipoprotein L, 2 /// apolipoprotein L, 2	16730	-0.0568038634955883	0.11107204	No
**216603_at**	SLC7A8	solute carrier family 7 (cationic amino acid transporter, y+ system), member 8	16808	-0.05769948661327362	0.10767326	No
**205389_s_at**	ANK1	ankyrin 1, erythrocytic /// ankyrin 1, erythrocytic	16832	-0.05791117995977402	0.10686337	No
**221874_at**	KIAA132 4	KIAA1324	16950	-0.05914630368351936	0.10155499	No
**207749_s_at**	PPP2R3A	protein phosphatase 2 (formerly 2A), regulatory subunit B", alpha	16989	-0.059532713145017624	0.100034446	No
**221619_s_at**	MTCH1	mitochondrial carrier homolog 1 (C. elegans)	17055	-0.06021531671285629	0.09722344	No
**204622_x_at**	NR4A2	nuclear receptor subfamily 4, group A, member 2	17056	-0.060216452926397324	0.0975274	No
**206301_at**	TEC	tec protein tyrosine kinase	17062	-0.060268957167863846	0.09759202	No
**213807_x_at**	MET	met proto-oncogene (hepatocyte growth factor receptor)	17075	-0.060402762144804	0.097321846	No
**209070_s_at**	RGS5	regulator of G-protein signalling 5	17093	-0.06069033592939377	0.09681352	No
**211428_at**	SERPINA 1	serpin peptidase inhibitor, clade A (alpha-1 antiproteinase, antitrypsin), member 1	17101	-0.06077354773879051	0.09678484	No
**209615_s_at**	PAK1	p21/Cdc42/Rac1-activated kinase 1 (STE20 homolog, yeast)	17157	-0.06127350777387619	0.094458394	No
**215087_at**	C15ORF3 9	chromosome 15 open reading frame 39	17203	-0.061688195914030075	0.09261327	No
**216183_at**	TGM2	transglutaminase 2 (C polypeptide, protein-glutamine-gamma-glutamyltransferase)	17222	-0.061893437057733536	0.0920631	No
**211356_x_at**	LEPR	leptin receptor	17238	-0.06211818754673004	0.09165782	No
**221583_s_at**	KCNMA1	potassium large conductance calcium-activated channel, subfamily M, alpha member 1	17392	-0.06402315944433212	0.08464885	No
**215333_x_at**	GSTM1	glutathione S-transferase M1	17412	-0.06424674391746521	0.08406263	No
**220225_at**	IRX4	iroquois homeobox protein 4	17417	-0.06428496539592743	0.084195435	No
**211355_x_at**	LEPR	leptin receptor	17496	-0.06529289484024048	0.08078706	No
**205391_x_at**	ANK1	ankyrin 1, erythrocytic /// ankyrin 1, erythrocytic	17546	-0.06601104885339737	0.078772075	No
**215405_at**	MYO1E	Myosin IE	17548	-0.06602846831083298	0.079057455	No
**206409_at**	TIAM1	T-cell lymphoma invasion and metastasis 1	17551	-0.06613162159919739	0.07929543	No
**206209_s_at**	CA4	carbonic anhydrase IV	17555	-0.06619144976139069	0.07948579	No
**221592_at**	TBC1D8	TBC1 domain family, member 8 (with GRAM domain)	17582	-0.06657955050468445	0.07857588	No
**207082_at**	CSF1	colony stimulating factor 1 (macrophage)	17664	-0.06742604076862335	0.075034514	No
**203333_at**	KIFAP3	kinesin-associated protein 3	17679	-0.06761740893125534	0.074704915	No

**215408_at**	MYO1E	Myosin IE	17758	-0.06894396245479584	0.071314976	No
**216639_at**	SRPX2	sushi-repeat-containing protein, X-linked 2	17787	-0.06940552592277527	0.0703235	No
**214921_at**	KCNMA1	potassium large conductance calcium-activated channel, subfamily M, alpha member 1	17828	-0.0700097382068634	0.06875999	No
**207379_at**	EDIL3	EGF-like repeats and discoidin I-like domains 3	17840	-0.07020572572946548	0.06858723	No
**218631_at**	AVPI1	arginine vasopressin-induced 1	17920	-0.07132795453071594	0.0651614	No
**210665_at**	TFPI	tissue factor pathway inhibitor (lipoprotein-associated coagulation inhibitor)	17968	-0.07191572338342667	0.06327206	No
**204494_s_at**	C15ORF39	chromosome 15 open reading frame 39	17981	-0.07207275927066803	0.0630608	No
**221840_at**	PTPRE	protein tyrosine phosphatase, receptor type, E	18009	-0.0723325908l840515	0.062132016	No
**214783_s_at**	ANXA11	annexin A11	18050	-0.07286202907562256	0.060582906	No
**212172_at**	AK2	adenylate kinase 2	18067	-0.07320472598075867	0.060185675	No
**220518_at**	ABI3BP	ABI gene family, member 3 (NESH) binding protein	18123	-0.07417598366737366	0.05792436	No
**213324_at**	SRC	v-src sarcoma (Schmidt-Ruppin A-2) viral oncogene homolog (avian)	18130	-0.0743161588907241 8	0.05801196	No
**204495_s_at**	C15ORF39	chromosome 15 open reading frame 39	18205	-0.07534315437078476	0.054846015	No
**211599_x_at**	MET	met proto-oncogene (hepatocyte growth factor receptor) /// met proto-oncogene (hepatocyte growth factor receptor)	18217	-0.07550634443759918	0.05470001	No
**205782_at**	FGF7	fibroblast growth factor 7 (keratinocyte growth factor)	18227	-0.07566623389720917	0.054650657	No
**207835_at**	FBLN1	fibulin 1	18263	-0.07623298466205597	0.05335818	No
**213791_at**	PENK	proenkephalin	18293	-0.07670389115810394	0.052355614	No
**205321_at**	EIF2S3	eukaryotic translation initiation factor 2, subunit 3 gamma, 52kDa	18387	-0.07835376262664795	0.048294336	No
**206142_at**	ZNF135	zinc finger protein 135	18444	-0.07951163500547409	0.046012033	No
**200980_s_at**	PDHA1	pyruvate dehydrogenase (lipoamide) alpha 1	18489	-0.0802755281329155	0.04430866	No
**204770_at**	TAP2	transporter 2, ATP-binding cassette, sub-family B (MDR/TAP)	18512	-0.08066903054714203	0.04366157	No
**201700_at**	CCND3	cyclin D3	18583	-0.08212662488222122	0.04072155	No
**215081_at**	KIAA1024	KIAA1024 protein	18598	-0.08231676369905472	0.04046616	No
**206644_at**	NR0B1	nuclear receptor subfamily 0, group B, member 1	18634	-0.08282949030399323	0.039206978	No
**211589_at**	PML	promyelocytic leukemia	18672	-0.08358155190944672	0.03785575	No
**222282_at**	PAPD4	PAP associated domain containing 4	18692	-0.08390072733163834	0.03736874	No
**205491_s_at**	GJB3	gap junction protein, beta 3, 31kDa (connexin 31)	18754	-0.0849413350224495	0.034874234	No
**204550_x_at**	GSTM1	glutathione S-transferase M1	18773	-0.08547161519527435	0.034443077	No

**218466_at**	TBC1D17	TBC1 domain family, member 17	18805	-0.08594813942909241	0.03339133	No
**207255_at**	LEPR	leptin receptor	18825	-0.0862715095281601	0.032916285	No
**219167_at**	RASL12	RAS-like, family 12	18836	-0.08647270500659943	0.03287356	No
**201329_s_at**	ETS2	v-ets erythroblastosis virus E26 oncogene homolog 2 (avian)	18840	-0.08652237057685852	0.033166543	No
**207660_at**	DMD	dystrophin (muscular dystrophy, Duchenne and Becker types)	18906	-0.08773399889469147	0.030494446	No
**205390_s_at**	ANK1	ankyrin 1, erythrocytic /// ankyrin 1, erythrocytic	18941	-0.08832307159900665	0.02931092	No
**204814_at**	CADPS	Ca2+-dependent secretion activator	18996	-0.089299775660038	0.027173873	No
**217824_at**	UBE2J1	ubiquitin-conjugating enzyme E2, J1 (UBC6 homolog, yeast)	18999	-0.08942443132400513	0.027529428	No
**215932_at**	MAGEC2	melanoma antigen family C, 2	19063	-0.09083344787359238	0.02496882	No
**220737_at**	RPS6KA6	ribosomal protein S6 kinase, 90kDa, polypeptide 6	19082	-0.09128625690937042	0.024567014	No
**209687_at**	CXCL12	chemokine (C-X-C motif) ligand 12 (stromal cell-derived factor 1)	19107	-0.09178564697504044	0.023880191	No
**201018_at**	EIF1AX	eukaryotic translation initiation factor 1A, X-linked	19114	-0.0918782576918602	0.024056442	No
**207087_x_at**	ANK1	ankyrin 1, erythrocytic	19142	-0.09261743724346161	0.023230052	No
**207648_at**	DRP2	dystrophin related protein 2	19172	-0.09313526749610901	0.02231043	No
**204686_at**	IRS1	insulin receptor substrate 1	19175	-0.09318052977323532	0.022684947	No
**213500_at**	COPB2	Coatomer protein complex, subunit beta 2 (beta prime)	19176	-0.09320803731679916	0.023155445	No
**206990_at**	TNR	tenascin R (restrictin, janusin)	19203	-0.09382806718349457	0.022383088	No
**220041_at**	PIGZ	phosphatidylinositol glycan, class Z	19222	-0.0943652093410492	0.021996822	No
**220778_x_at**	SEMA6B	sema domain, transmembrane domain (TM), and cytoplasmic domain, (semaphorin) 6B	19230	-0.09446588158607483	0.022138214	No
**209249_s_at**	GHITM	growth hormone inducible transmembrane protein	19239	-0.09470434486865997	0.022232886	No
**205618_at**	PRRG1	proline rich Gla (G-carboxyglutamic acid) 1	19241	-0.09475785493850708	0.022663286	No
**214888_at**	CAPN2	calpain 2, (m/II) large subunit	19273	-0.09567064046859741	0.021660618	No
**219631_at**	LRP12	low density lipoprotein-related protein 12	19322	-0.09678627550601959	0.019848896	No
**213201_s_at**	TNNT1	troponin T type 1 (skeletal, slow)	19331	-0.09699692577123642	0.019955141	No
**204412_s_at**	NEFH	neurofilament, heavy polypeptide 200kDa	19341	-0.0972348004579544	0.020014664	No
**203786_s_at**	TPD52L1	tumor protein D52-like 1	19379	-0.09794141352176666	0.018735923	No
**210311_at**	FGF5	fibroblast growth factor 5	19410	-0.09869006276130676	0.017796418	No
**208353_x_at**	ANK1	ankyrin 1, erythrocytic	19420	-0.09881626814603806	0.017863924	No
**209676_at**	TFPI	tissue factor pathway inhibitor (lipoprotein-associated coagulation inhibitor)	19428	-0.09902141988277435	0.018028311	No

**209248_at**	GHITM	growth hormone inducible transmembrane protein	19449	-0.09959448128938675	0.017572597	No
**219789_at**	NPR3	natriuretic peptide receptor C/guanylate cyclase C (atrionatriuretic peptide receptor C)	19463	-0.09990260750055313	0.017453896	No
**202017_at**	EPHX1	epoxide hydrolase 1, microsomal (xenobiotic)	19512	-0.10129313915967941	0.015664926	No
**217825_s_at**	UBE2J1	ubiquitin-conjugating enzyme E2, J1 (UBC6 homolog, yeast)	19553	-0.10211841762065887	0.014263501	No
**214474_at**	PRKAB2	protein kinase, AMP-activated, beta 2 non-catalytic subunit	19559	-0.10224782675504684	0.014540019	No
**201117_s_at**	CPE	carboxypeptidase E	19570	-0.10240734368562698	0.01457773	No
**219421_at**	TTC33	tetratricopeptide repeat domain 33	19677	-0.10559847950935364	0.010030984	No
**205018_s_at**	MBNL2	muscleblind-like 2 (Drosophila)	19706	-0.106841541826725	0.009228472	No
**210300_at**	REM1	RAS (RAD and GEM)-like GTP-binding 1	19814	-0.11026871204376221	0.004657377	No
**204716_at**	CCDC6	coiled-coil domain containing 6	19885	-0.11271631717681885	0.0018717725	No
**204675_at**	SRD5A1	steroid-5-alpha-reductase, alpha polypeptide 1 (3-oxo-5 alpha-steroid delta 4-dehydrogenase alpha 1)	19895	-0.11308272182941437	0.0020112933	No
**206767_at**	RBMS3	RNA binding motif, single stranded interacting protein	19910	-0.11370080709457397	0.0019143213	No
**208352_x_at**	ANK1	ankyrin 1, erythrocytic	19944	-0.11482202261686325	9,12E+03	No
**205562_at**	RPP38	ribonuclease P/MRP 38kDa subunit	19953	-0.11505100876092911	0.0011098594	No
**220038_at**	SGK3	serum/glucocorticoid regulated kinase family, member 3	19980	-0.11597439646720886	4,49E+02	No
**205626_s_at**	CALB1	calbindin 1, 28kDa	20022	-0.11744040995836258	-9,23E+02	No
**201328_at**	ETS2	v-ets erythroblastosis virus E26 oncogene homolog 2 (avian)	20041	-0.11817789822816849	-0.0011887731	No
**220777_at**	KIF13A	kinesin family member 13A	20227	-0.12578432261943817	-0.009419506	No
**204161_s_at**	ENPP4	ectonucleotide pyrophosphatase/phosphodiesterase 4 (putative function)	20290	-0.12839394807815552	-0.011742593	No
**212641_at**	HIVEP2	human immunodeficiency virus type I enhancer binding protein 2	20418	-0.13437095284461975	-0.017150473	No
**210372_s_at**	TPD52L1	tumor protein D52-like 1	20454	-0.13644611835479736	-0.018139005	No
**202747_s_at**	ITM2A	integral membrane protein 2A	20456	-0.13646721839904785	-0.017498063	No
**205625_s_at**	CALB1	calbindin 1, 28kDa	20608	-0.14383816719055176	-0.024008296	No
**213135_at**	TIAM1	T-cell lymphoma invasion and metastasis 1	20635	-0.14475950598716736	-0.024523558	No
**218608_at**	ATP13A2	ATPase type 13A2	20653	-0.14584164321422577	-0.024602057	No
**217853_at**	TNS3	tensin 3	20665	-0.14655247330665588	-0.02438943	No
**204729_s_at**	STX1A	syntaxin 1A (brain)	20713	-0.1498134434223175	-0.025885556	No
**218573_at**	MAGEH1	melanoma antigen family H, 1	20729	-0.1506660431623459	-0.025843857	No

**203158_s_at**	GLS	glutaminase	20732	-0.15078853070735931	-0.025178544	No
**205273_s_at**	PITRM1	pitrilysin metallopeptidase 1	20763	-0.15227970480918884	-0.025847537	No
**203157_s_at**	GLS	glutaminase	20801	-0.15417584776878357	-0.026842415	No
**203837_at**	MAP3K5	mitogen-activated protein kinase kinase kinase 5	20805	-0.15429745614528656	-0.026207315	No
**218087_s_at**	SORBS1	sorbin and SH3 domain containing 1	20818	-0.15519514679908752	-0.025998984	No
**204339_s_at**	RGS4	regulator of G-protein signalling 4	20854	-0.157202810049057	-0.026882738	No
**218292_s_at**	PRKAG2	protein kinase, AMP-activated, gamma 2 non-catalytic subunit	20899	-0.16014905273914337	-0.028182924	No
**204337_at**	RGS4	regulator of G-protein signalling 4	20926	-0.161294624209404	-0.02861472	No
**203159_at**	GLS	glutaminase	20972	-0.1643132120370865	-0.029941808	No
**203390_s_at**	KIF3C	kinesin family member 3C	20981	-0.1647927463054657	-0.029493341	No
**220663_at**	IL1RAPL 1	interleukin 1 receptor accessory protein-like 1	20982	-0.1648615002632141	-0.028661145	No
**204612_at**	PKIA	protein kinase (cAMP-dependent, catalytic) inhibitor alpha	20991	-0.16546028852462769	-0.028209308	No
**210770_s_at**	CACNA1 A	calcium channel, voltage-dependent, P/Q type, alpha 1A subunit	21032	-0.16826923191547394	-0.029276814	No
**204338_s_at**	RGS4	regulator of G-protein signalling 4	21052	-0.17000176012516022	-0.029329201	No
**210461_s_at**	ABLIM1	actin binding LIM protein 1	21070	-0.17111872136592865	-0.029280104	No
**221728_x_at**	XIST	X (inactive)-specific transcript	21072	-0.17116066813468933	-0.028464034	No
**201034_at**	ADD3	adducin 3 (gamma)	21107	-0.17370256781578064	-0.029216578	No
**205471_s_at**	DACH1	dachshund homolog 1 (Drosophila)	21114	-0.17409920692443848	-0.028625287	No
**206702_at**	TEK	TEK tyrosine kinase, endothelial (venous malformations, multiple cutaneous and mucosal)	21119	-0.17481917142868042	-0.027934518	No
**209726_at**	CA11	carbonic anhydrase XI	21151	-0.17761538922786713	-0.028523542	No
**212956_at**	TBC1D9	TBC1 domain family, member 9	21156	-0.1779506802558899	-0.027816966	No
**206275_s_at**	MICAL2	microtubule associated monoxygenase, calponin and LIM domain containing 2	21181	-0.1806897521018982	-0.028055014	No
**203836_s_at**	MAP3K5	mitogen-activated protein kinase kinase kinase 5	21202	-0.18258097767829895	-0.028091824	No
**201753_s_at**	ADD3	adducin 3 (gamma)	21215	-0.18371309340000153	-0.02773954	No
**206973_at**	PPFIA2	protein tyrosine phosphatase, receptor type, f polypeptide (PTPRF), interacting protein (liprin), alpha 2	21227	-0.18481957912445068	-0.027333748	No
**209684_at**	RIN2	Ras and Rab interactor 2	21261	-0.18751776218414307	-0.027968632	No
**202073_at**	OPTN	optineurin	21268	-0.18779447674751282	-0.02730821	No
**204547_at**	RAB40B	RAB40B, member RAS oncogene family	21270	-0.18784497678279877	-0.026407922	No
**214933_at**	CACNA1 A	calcium channel, voltage-dependent, P/Q type, alpha 1A subunit	21318	-0.1923600733280182	-0.027689278	No

**209074_s_at**	FAM107A	family with sequence similarity 107, member A	21320	-0.1926003098487854	-0.026764985	No
**219389_at**	SUSD4	sushi domain containing 4	21338	-0.19394929707050323	-0.026600642	No
**201116_s_at**	CPE	carboxypeptidase E	21353	-0.19601711630821228	-0.026282094	No
**205830_at**	CLGN	calmegin	21395	-0.2002772092819214	-0.027235951	No
**204160_s_at**	ENPP4	ectonucleotide pyrophosphatase/phosphodiesterase 4 (putative function)	21403	-0.20104484260082245	-0.026556566	No
**218353_at**	RGS5	regulator of G-protein signalling 5	21408	-0.2014036327600479	-0.025731603	No
**219799_s_at**	DHRS9	dehydrogenase/reductase (SDR family) member 9	21416	-0.2022528052330017	-0.02504612	No
**214218_s_at**	XIST	X (inactive)-specific transcript	21441	-0.2047070860862732	-0.025162932	No
**213035_at**	ANKRD28	ankyrin repeat domain 28	21488	-0.21028152108192444	-0.0263059	No
**203389_at**	KIF3C	kinesin family member 3C	21512	-0.2138569951057434	-0.026328605	No
**201752_s_at**	ADD3	adducin 3 (gamma)	21558	-0.2209016978740692	-0.027370041	No
**211819_s_at**	SORBS1	sorbin and SH3 domain containing 1	21560	-0.22106978297233582	-0.02630204	No
**204424_s_at**	LMO3	LIM domain only 3 (rhombotin-like 2)	21567	-0.22219814360141754	-0.025467953	No
**205882_x_at**	ADD3	adducin 3 (gamma)	21576	-0.22355996072292328	-0.024722837	No
**204454_at**	LDOC1	leucine zipper, down-regulated in cancer 1	21603	-0.22635743021965027	-0.024826208	No
**206356_s_at**	GNAL	guanine nucleotide binding protein (G protein), alpha activating activity polypeptide, olfactory type	21626	-0.22894150018692017	-0.024724843	No
**206331_at**	CALCRL	calcitonin receptor-like	21649	-0.23210301995277405	-0.024607519	No
**202746_at**	ITM2A	integral membrane protein 2A	21655	-0.23281921446323395	-0.023671897	No
**207547_s_at**	FAM107A	family with sequence similarity 107, member A	21674	-0.23494555056095123	-0.023348534	No
**205952_at**	KCNK3	potassium channel, subfamily K, member 3	21737	-0.2493169903755188	-0.02506122	No
**206117_at**	TPM1	tropomyosin 1 (alpha)	21776	-0.25767651200294495	-0.025581567	No
**206645_s_at**	NR0B1	nuclear receptor subfamily 0, group B, member 1	21793	-0.2614171802997589	-0.025028735	No
**208389_s_at**	SLC1A2	solute carrier family 1 (glial high affinity glutamate transporter), member 2	21810	-0.2644945979118347	-0.02446037	No
**33767_at**	NEFH	neurofilament, heavy polypeptide 200kDa	21816	-0.2654890716075897	-0.023359835	No
**207323_s_at**	MBP	myelin basic protein	21819	-0.2661219835281372	-0.022112338	No
**216594_x_at**	AKR1C1	aldo-keto reductase family 1, member C1 (dihydrodiol dehydrogenase 1; 20-alpha (3-alpha)-hydroxysteroid dehydrogenase)	21882	-0.28260666131973267	-0.023656981	No
**200965_s_at**	ABLIM1	actin binding LIM protein 1	21918	-0.2941366732120514	-0.023849515	No
**209755_at**	NMNAT2	nicotinamide nucleotide adenylyltransferase 2	21926	-0.2952931523323059	-0.022694381	No
**219305_x_at**	FBXO2	F-box protein 2	21962	-0.30613768100738525	-0.022826334	No
**206355_at**	GNAL	guanine nucleotide binding protein (G protein), alpha	21983	-0.31346482038497925	-0.022202464	No

		activating activity polypeptide, olfactory type				
**214589_at**	FGF12	fibroblast growth factor 12	21989	-0.3163500726222992	-0.020845192	No
**204151_x_at**	AKR1C1	aldo-keto reductase family 1, member C1 (dihydrodiol dehydrogenase 1; 20-alpha (3-alpha)-hydroxysteroid dehydrogenase)	21993	-0.31690549850463867	-0.01938927	No
**209072_at**	MBP	myelin basic protein	22012	-0.32353675365448	-0.018618714	No
**205290_s_at**	BMP2	bone morphogenetic protein 2	22021	-0.32746750116348267	-0.017349089	No
**203798_s_at**	VSNL1	visinin-like 1	22029	-0.33013638854026794	-0.01601807	No
**205289_at**	BMP2	bone morphogenetic protein 2	22037	-0.3341241180896759	-0.014666922	No
**209981_at**	CSDC2	cold shock domain containing C2, RNA binding	22075	-0.354461669921875	-0.01465079	No
**203797_at**	VSNL1	visinin-like 1	22081	-0.35708820819854736	-0.013087878	No
**219368_at**	NAP1L2	nucleosome assembly protein 1-like 2	22084	-0.3581567108631134	-0.0113758035	No
**206448_at**	ZNF365	zinc finger protein 365	22089	-0.36116212606430054	-0.009744404	No
**202507_s_at**	SNAP25	synaptosomal-associated protein, 25kDa	22123	-0.3845044672489166	-0.00938493	No
**207501_s_at**	FGF12	fibroblast growth factor 12	22156	-0.4153258502483368	-0.008821952	No
**205827_at**	CCK	cholecystokinin	22159	-0.41719669103622437	-0.0068118544	No
**210815_s_at**	CALCRL	calcitonin receptor-like	22176	-0.43791863322257996	-0.0053680707	No
**202508_s_at**	SNAP25	synaptosomal-associated protein, 25kDa	22201	-0.47124162316322327	-0.0041394583	No
**203001_s_at**	STMN2	stathmin-like 2	22219	-0.5103008151054382	-0.0023782235	No
**209160_at**	AKR1C3	aldo-keto reductase family 1, member C3 (3-alpha hydroxysteroid dehydrogenase, type II)	22232	-0.5234576463699341	-3,11E+02	No
**203000_at**	STMN2	stathmin-like 2	22238	-0.526969313621521	0.0021094838	No

Supplementary Methods

Bioinformatics analysis of microarray data

Quality control of hybridization and outlier detection was done by Image Quality, MAplots, Boxplot and Density Plot, by means of R package *arrayQualityMetrics* (19106121). Array normalization was executed by the RMA algorithm (12925520) using the R package *affy* (14960456). Divisive clustering algorithms were used to obtain dendrograms, in which the biologic samples were clustered on the basis of the differentially expressed genes. The hierarchical clustering algorithms employed were (i) distances (Euclidian, correlation), (ii) linkage (complete, single, mcquitty, ward, and centroid). The differentially expressed genes (DEG) were obtained based on (i) *t* test moderated empirical Bayes, (ii) *P* value [false discovery rate (FDR) adjusted 0.05], (iii) cut-off (1 log2 Fold Change, FC) as implemented in the R package *limma* (25605792).

NanoPro Assay

Standard pI Ladder 1 was added to the ampholyte pre-mix. Lysates were then separated for 40 min at 21000 μW in individual capillaries. After separation the proteins in the lysate were immobilized to the capillary wall by subjecting them to UV exposure for a period of 80 s. Anti-beta-2-Microglobulin antibody was used as loading control (Abcam). Secondary anti-rabbit-HRP-conjugated antibodies were loaded into the capillary for 1 h. A luminol-peroxidase 1:1 mix was flowed through the capillaries and chemiluminescence was detected at 30, 60, 120, 240, and 480s.

MRI sequences and analysis

DCE-MR imaging

DCE-MR imaging was performed during the injection of gadobutrol (Gadavist; Bayer Schering Pharma, Berlin, Germany) by using a dynamic gradient-echo T1-weighted sequence (TR/TE = 50/3 ms, flip angle = 35°, matrix = 170×170, in-plane voxel size = 0.11 mm^2, section thickness = 0.75 mm). Ninety dynamic scans were obtained. DCE-MR imaging was preceded by a saturation recovery sequence for T1 mapping and followed by a contrast-enhanced T1 sequence for anatomic reference.

DCE-MRI data analysis

DCE-MR imaging data were analyzed by using the software nordicICE (NordicNeuroLab, Bergen, Norway). The modified pharmacokinetic model of Tofts was used for the generation of V_p and K^{trans} maps after deconvolution with an averaged arterial input function. Quantitative values were then obtained from both the region of maximal abnormality (hotspots) and histogram analysis. Volumes of interest were drawn on contrast T1-weighted images, previously coregistered to the DCE-MRI parametric maps using FSL (FMRIB software; Oxford Center for Functional MR Imaging of the Brain, Oxford, England). All the values of the plasma volume (V_p) and contrast transfer coefficient (K^{trans}) parameters were normalized over the values measured on the contralateral hemisphere. Histograms in DCE maps for the various segmented areas were generated using MATLAB and analysed to provide further quantitative insight. The main parameters were obtained from the histograms: mean, median, standard deviation, skewness, kurtosis, 10th, 25th, 75th 90th and 95th percentile.

Primary and secondary antibodies used in the study

mouse anti-MASH1 (ASCL1)	BD Pharmingen
mouse anti-EGFR	Dako
mouse anti-CD31	Dako
mouse anti-GFAP	Chemicon
rabbit anti-GFAP	Dako
mouse anti-Tuj1	Covance
rabbit anti-Olig2	Millipore
rabbit anti-Tuj1	Covance
mouse anti-MAP2	Millipore
rabbit anti-PDGFRα	Thermo Scientific
rabbit anti-YKL40	Quidel
rabbit anti-SGK	Abcam
rabbit anti-$pSGK_{S422}$	Santa Cruz Biotechnology
rabbit anti-Calnexin	Genetex
rabbit anti-NDRG1 clone D6C2	Cell Signaling
rabbit anti-pNDRG1	Cell Signaling
rabbit anti-Akt clone C67E7	Cell Signaling
mouse anti-$pAkt_{S473}$ D9E	Cell Signaling
rabbit anti-ERK	Cell Signaling
rabbit anti-$pERK1/2_{T202/Y204}$	Cell Signaling
mouse anti-Ki67 (clone MIB-1)	Dako
Alexa Fluor®546 goat anti-mouse IgG (H+L)	Invitrogen
Alexa Fluor®546 goat anti-rabbit IgG (H+L)	Invitrogen
Alexa Fluor®488 goat anti-mouse IgG (H+L)	Invitrogen
Alexa Fluor®488 goat anti-rabbit IgG (H+L)	Invitrogen
